# Supplementary material for: Efficient and versatile formation of glycosidic bonds via catalytic strain-release glycosylation with glycosyl ortho−2,2-dimethoxycarbonylcyclopropylbenzoate donors
Source: Nat Commun. 2023 Jul 7;14:4010. doi: 10.1038/s41467-023-39619-7 (PMC10329021; doi:10.1038/s41467-023-39619-7)
Supplement: Supplementary file 1 — Supplementary Information [file 41467_2023_39619_MOESM1_ESM.pdf]

Supplementary Information for

Efficient and Versatile Formation of Glycosidic Bonds via Catalytic Strain-Release Glycosylation with Glycosyl *ortho*-2,2-Dimethoxycarbonylcyclopropylbenzoate Donors

Han Ding,[1] Jian Lyu,[1] Xiao-Lin Zhang,[1] Xiong Xiao,[2]* and Xue-Wei Liu[1]*

[1]School of Chemistry, Chemical Engineering and Biotechnology, Nanyang Technological University, 21 Nanyang Link, Singapore 637371.

[2]School of Chemistry and Chemical Engineering, Northwestern Polytechnical University (NPU) Xi'an 710072, P. R. of China.

xiongxiao@nwpu.edu.cn; xuewei@ntu.edu.sg

Table of contents

**Supplementary Note**	S6
**Supplementary Methods**	S7
**Section 1. Synthesis of CCBzOH and glycosyl donors.**	S7
Synthesis of compound **S3**	S7
Synthesis of compound **S4**	S7
Synthesis of compound **S5**	S8
Synthesis of compound **S6**	S9
Synthesis of **CCBzOH**	S9
Synthesis of donor **1a**	S10
Synthesis of donor **1b**	S12
Synthesis of donor **1c**	S13
Synthesis of donor **1d**	S14
Synthesis of donor **1e**	S15
Synthesis of donor **1f**	S16
Synthesis of donor **1g**	S16
Synthesis of donor **1h**	S17
**Section 2. Reaction development of strain-release glycosylation.**	S19
Supplementary Table 1. Reaction development	S19
General procedure for the reaction development	S19
**Section 3. Strain-release glycosylation with 1a as the donor.**	S21
Synthesis of compound **3a**.	S21
Synthesis of compound **3b**	S21
Synthesis of compound **3c**	S22
Synthesis of compound **3d**	S22
Synthesis of compound **3e**	S23
Synthesis of compound **3f**	S24
Synthesis of compound **3g**	S24
Synthesis of compound **3h**	S25
Synthesis of compound **3i**	S25
Synthesis of compound **3j**	S26
Synthesis of compound **3k**	S27
Synthesis of compound **3l**	S27
Synthesis of compound **3m**	S28
Synthesis of compound **3n**	S28
Synthesis of compound **3o**	S29
Synthesis of compound **3p**	S30
Synthesis of compound **3q**	S30
Synthesis of compound **3r**	S31
**Section 4. Strain-release glycosylation with 1b-h as the donors.**	S32
Synthesis of compound **3ba**	S32
Synthesis of compound **3bb**	S32

Synthesis of compound **3bc**	S33
Synthesis of compound **3ca**	S33
Synthesis of compound **3cb**	S34
Synthesis of compound **3cc**	S35
Synthesis of compound **3da**	S35
Synthesis of compound **3db**	S36
Synthesis of compound **3dc**	S37
Synthesis of compound **3ea**	S37
Synthesis of compound **3eb**	S38
Synthesis of compound **3ec**	S39
Synthesis of compound **3fa**	S39
Synthesis of compound **3fb**	S40
Synthesis of compound **3fc**	S41
Synthesis of compound **3ga**	S41
Synthesis of compound **3gb**	S42
Synthesis of compound **3gc**	S43
Synthesis of compound **3ha**	S43
Synthesis of compound **3hb**	S44
Synthesis of compound **3hc**	S45
**Section 5. Divergent synthesis of chitooligosaccharides.**	S47
Synthesis of compound **7**	S47
Synthesis of compound **9**	S47
Synthesis of compound **10**	S48
Synthesis of compound **11**	S49
Synthesis of compound **13**	S50
Synthesis of compound **14**	S51
Synthesis of compound **15**	S51
Synthesis of compound **16**	S53
Synthesis of compound **17**	S54
Synthesis of compound **19**	S55
Synthesis of compound **21**	S56
Synthesis of compound **22**	S57
Synthesis of compound **23**	S58
Synthesis of compound **24**	S59
**Section 6. NMR spectra.**	S61
^{1}H spectrum of **S4**	S61
^{1}H and ^{13}C spectra for **CCBzOH**	S62
^{1}H and ^{13}C spectra for **1a**	S63
^{1}H and ^{13}C spectra for **1b**	S64
^{1}H and ^{13}C spectra for **1c**	S65
^{1}H and ^{13}C spectra for **1d**	S66
^{1}H and ^{13}C spectra for **1e**	S67
^{1}H and ^{13}C spectra for **1f**	S68

^{1}H and ^{13}C spectra for **1g**	S69
^{1}H and ^{13}C spectra for **1h**	S70
^{1}H and ^{13}C spectra for **4**	S71
^{1}H spectrum for **3a**	S72
^{1}H spectrum for **3b**	S73
^{1}H spectrum for **3c**	S74
^{1}H spectrum for **3d**	S75
^{1}H spectrum for **3e**	S76
^{1}H spectrum for **3f**	S77
^{1}H spectrum for **3g**	S78
^{1}H spectrum for **3h**	S79
^{1}H spectrum for **3i**	S80
^{1}H spectrum for **3j**	S81
^{1}H spectrum for **3k**	S82
^{1}H spectrum for **3l**	S83
^{1}H spectrum for **3m**	S84
^{1}H and ^{13}C spectra for **3n**	S85
^{1}H spectrum for **3o**	S86
^{1}H spectrum for **3p**	S87
^{1}H spectrum for **3q**	S88
^{1}H and ^{13}C spectra for **3r**	S89
^{1}H spectrum for **3ba**	S90
^{1}H spectrum for **3bb**	S91
^{1}H and ^{13}C spectra for **3bc**	S92
^{1}H spectrum for **3ca**	S93
^{1}H and ^{13}C spectra for **3cb**	S94
^{1}H and ^{13}C spectra for **3cc**	S95
^{1}H spectrum for **3da**	S96
^{1}H and ^{13}C spectra for **3db**	S97
^{1}H and ^{13}C spectra for **3dc**	S98
^{1}H and ^{13}C spectra for **3ea**	S99
^{1}H and ^{13}C spectra for **3eb**	S100
^{1}H and ^{13}C spectra for **3ec**	S101
^{1}H spectrum for **3fa**	S102
^{1}H and ^{13}C spectra for **3fb**	S103
^{1}H and ^{13}C spectra for **3fc**	S104
^{1}H spectrum for **3ga**	S105
^{1}H and ^{13}C spectra for **3gb**	S106
^{1}H and ^{13}C spectra for **3gc**	S107
^{1}H and ^{13}C spectra for **3ha**	S108
^{1}H and ^{13}C spectra for **3hb**	S109
^{1}H and ^{13}C spectra for **3hc**	S110
^{1}H and ^{13}C spectra for **7**	S111

^{1}H spectrum for **9**	S112
^{1}H spectrum for **10**	S113
^{1}H, ^{13}C, ^{1}H-^{1}H COSY and ^{1}H-^{13}C HSQC spectra for **11**	S114
^{1}H, ^{13}C, ^{1}H-^{1}H COSY and ^{1}H-^{13}C HSQC spectra for **13**	S116
^{1}H and ^{13}C spectra for **14**	S118
^{1}H and ^{13}C spectra for **15**	S119
^{1}H and ^{13}C spectra for **16**	S120
^{1}H, ^{13}C, ^{1}H-^{1}H COSY and ^{1}H-^{13}C HSQC spectra for **17**	S121
^{1}H and ^{13}C spectra for **19**	S123
^{1}H, ^{13}C, ^{1}H-^{1}H COSY and ^{1}H-^{13}C HSQC spectra for **21**	S124
^{1}H, ^{13}C, ^{1}H-^{1}H COSY and ^{1}H-^{13}C HSQC spectra for **22**	S126
^{1}H and ^{13}C spectra for **23**	S128
^{1}H, ^{13}C, ^{1}H-^{1}H COSY and ^{1}H-^{13}C HSQC spectra for **24**	S129
**Section 7. Supplementary References.**	S131

Supplementary Note

All reactions were carried out under argon or nitrogen atmosphere with magnetic stirring unless otherwise indicated. All commercially obtained reagents were used as received, except where specified otherwise. $Sc(OTf)_3$ was purchased from Alfa and used without further purification. Tetrahydrofuran (THF) and toluene were distilled immediately before use from sodium-benzophenone ketyl. Dichloromethane (CH_2Cl_2), pyridine and acetonitrile were refluxed over calcium hydride and distilled before use. Anhydrous *N*,*N*-dimethylformamide (DMF) was purchased from Sigma-Aldrich and used without further purification. Flash column chromatography was performed on Silica Gel H (300–400 mesh, Qingdao, China). Analytical thin layer chromatography was performed on Silicycle SiliaPlate glass-backed plates coated with silica gel (60 mesh pore size, F-254 indicator) and visualized by exposure to ultraviolet light and/or staining with 7% sulfuric acid in methanol. Optical rotations were determined with a JASCO P-1020 digital polarimeter. All NMR spectra were recorded with Bruker BBFO-400 (400 MHz) NMR spectrometer at ambient temperature using $CDCl_3$, CD_2Cl_2 or D_2O as solvents. The NMR spectra were calibrated by using residual undeuterated chloroform (δ_H = 7.26 ppm), $CDCl_3$ (δ_C = 77.16 ppm), residual undeuterated dichloromethane (δ_H = 5.32 ppm), CD_2Cl_2 (δ_C = 53.84 ppm) and undeuterated H_2O (δ_H = 4.79 ppm) as internal references. The following abbreviations are used to designate multiplicities: s = singlet, d = doublet, t = triplet, q = quartet, m = multiplet, brs = broad singlet, COSY = ^1H-^1H correlation spectroscopy, HSQC = heteronuclear single-quantum correlation spectroscopy.

Supplementary Methods

Section 1. Synthesis of CCBzOH and glycosyl donors.

1,3-Dimethyl 2-[(2-methylphenyl)methylene]propanedioate (S3)

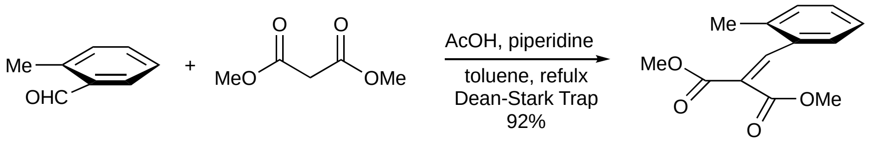

Supplementary Fig. 1 | Synthesis of S3.

In a 500 mL round bottom flask, to a solution of commercially available *ortho*-tolualdehyde **S1** (16.3 mL, 140 mmol, 1.3 equiv) and dimethyl malonate (12.4 mL, 107 mmol, 1.0 equiv) in toluene (200 mL) were added sequentially piperidine (2.1 mL, 21.4 mmol, 0.2 equiv) and AcOH (0.92 mL, 16.05 mmol, 0.15 equiv). The flask was equipped with a Dean-Stark trap which was filled with toluene. The reaction apparatus was immersed in an oil bath which was set to 150 °C. The mixture was stirred at 150 °C for 4 h before the mixture was cooled to room temperature and then concentrated *in vauco*. The residue was purified by silica gel column chromatography (hexane:EtOAc = 12:1 to 9:1) to afford the titled compound **S3** (23.05 g, 98.4 mmol, 92%) as a colorless oil which can solidify when storing in the refrigerator.

Tips: *With the reaction goes, the color of the reaction mixture turns from colorless to orange. A slightly excess of ortho-tolualdehyde is necessary to ensure the full consumption of the dimethyl malonate. When carrying out the high temperature experiment, an aluminum foil or cotton is suggested to maintain the temperature.*

ortho-2,2-Dimethoxycarbonylcyclopropyltoluene (S4)

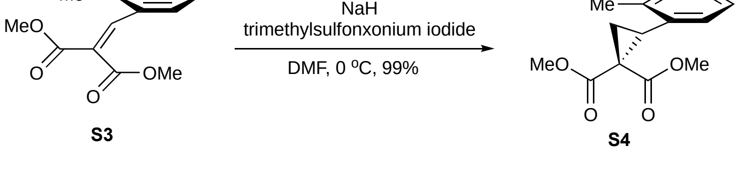

Supplementary Fig. 2 | Synthesis of S4.

A dry 500 mL round bottom flask was charged with trimethylsulfonxonium iodide (TMSI, 24.9 g, 108.2 mmol, 1.1 equiv). Anhydrous DMF (150 mL) was added and the

mixture was cooled by an ice-bath. NaH (60% in mineral, 4.33 g, 108.2 mmol, 1.1 equiv) was added in portions and the mixture was stirred at 0 °C for 1 h before a solution of **S3** obtained from last step in anhydrous DMF (50 mL) was added into the reaction mixture dropwise through a constant pressure dropping funnel. The mixture was stirred under the ice bath for another 1 h before the reaction was quenched with sat. NH_4Cl solution. The mixture was concentrated to around 50 mL before it was diluted with EtOAc. The organic phase was washed sequentially with 1 M HCl solution and sat. $NaHCO_3$ solution. The organic layers were combined, dried over Na_2SO_4, filtered and concentrated *in vauco*. The residue was purified by silica gel column chromatography (hexane:EtOAc = 9:1) to afford the titled compound **S4** (24.3 g, 98.0 mmol, 99%) as a colorless oil. ^{1}H NMR (400 MHz, $CDCl_3$) δ 7.20 – 7.07 (m, 3H), 7.03 (d, J = 7.4 Hz, 1H), 3.81 (s, 3H), 3.29 (s, 3H), 3.18 (t, J = 8.7 Hz, 1H), 2.36 (s, 3H), 2.32 (dd, J = 8.3, 5.1 Hz, 1H), 1.72 (dd, J = 9.2, 5.1 Hz, 1H). The data are identical with the literature report.[1]

Tips: *At the initial stage of the transformation, TMSI is a well-dispersed solid precipitated at the bottom of the flask. After the addition of the NaH and stirring for a while, the reaction mixture turned to a cloudy solution, indicating the formation of NaI and required ylide. When a nearly clear solution is formed, the addition of alkene substrate can be proceeded. The anhydrous DMF is essential to the successful synthesis of* ***S4****, and the undry DMF will cause the decomposition of the products, which is indicated by the formation of a very polar spot on TLC. The quenching step is similarly important. Without the addition of* NH_4Cl *solution, the color of the mixture will turn from colorless to dark purple to black during the concentration of the mixture, indicating the product has decomposed.*

***ortho*-2,2-Dimethoxycarbonylcyclopropylbenzyl bromide (S5)**

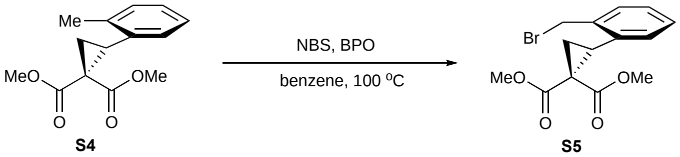

Supplementary Fig. 3 | Synthesis of S5.

To a solution of the mixture of compound **S4** obtained from last step in benzene (150 mL) were added benzoyl peroxide (BPO, 70% in water, 5.09 g, 14.7 mmol, 0.15 equiv) and *N*-bromosuccinimide (NBS, 26.2 g, 147 mmol, 1.5 equiv). The mixture was heated by the oil bath at 100 ºC for 1 h before it was concentrated *in vauco*. The residue was purified by silica gel column chromatography (hexane:EtOAc = 9:1) to afford a mixture of the desired monobrominated **S5** and the dibrominated side product as a light yellow oil, which was used directly for the next step without further purification.

Tips: *The reaction time and the equivalent of NBS significantly matter the ratio of monobromination and dibromination. During the reaction, the color changes from colorless to orange. Typically, after the NBS was fully dissolved in the mixture, the reaction completes. This process generally takes 1 h. Meanwhile, it is not necessary to separate the* ***S5*** *and dibrominated side product. The dibrominated side product will not participate the next oxidation step.*

***ortho*-2,2-Dimethoxycarbonylcyclopropylbenzaldehyde (S6)**

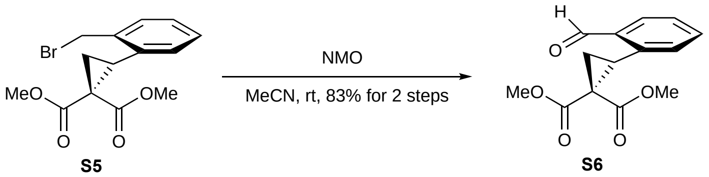

Supplementary Fig. 4 | Synthesis of S6.

To a solution of the mixture from last step in MeCN (150 mL) was added 4-methylmorpholine *N*-oxide (34.4 g, 294 mmol, 3.0 equiv) portionwise. The mixture was stirred at room temperature for 1 h before it was concentrated *in vauco*. The residue was purified by silica gel column chromatography (hexane:EtOAc = 6:1 to 4:1) to afford the titled compound **S5** (21.3 g, 81.3 mmol, 83% over 2 steps) as a colorless oil.

Tips: *At this stage, the aldehyde and dibrominated side product from last step is very easy to separate.*

***ortho*-2,2-Dimethoxycarbonylcyclopropylbenzoic acid (CCBzOH)**

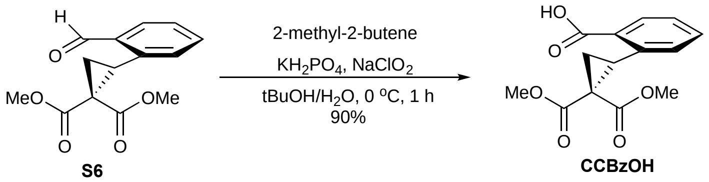

Supplementary Fig. 5 | Synthesis of CCBzOH.

To a solution of **S6** (9.28 g, 35.4 mmol, 1.0 equiv), KH_2PO_4 (16.4 g, 120 mmol, 3.4 equiv) in a mixed solvent of H_2O/*t*BuOH (v/v = 9:8, 340 mL) at 0 °C were sequentially added 2-methyl-2-butene (40 mL) and $NaClO_2$ (18.0 g, 200 mmol, 5.6 equiv). The mixture was stirred at this temperature for 1 h before the mixture was allowed to warm-up to room temperature. The volatile was removed under reduced pressure and the residue was acidified by 1 M HCl solution to form a white precipitate. The aqueous layer was treated with EtOAc. Two phases were separated, and the aqueous phase was extracted with EtOAc. The organic layers were combined, dried over anhydrous Na_2SO_4, filtered and concentrated *in vauco* to afford a yellowish solid, which was washed with hexane to obtain the pure **CCBzOH** (8.9 g, 31.9 mmol, 90%) as a white solid. ^{1}H NMR (400 MHz, $CDCl_3$) δ 8.06 (dd, J = 7.8, 1.4 Hz, 1H), 7.51 (td, J = 7.6, 1.5 Hz, 1H), 7.41 – 7.34 (m, 1H), 7.29 (d, J = 7.7 Hz, 1H), 4.85 (brs, 1H), 3.86 – 3.77 (m, 4H), 3.34 (s, 3H), 2.21 (dd, J = 8.5, 5.1 Hz, 1H), 1.90 (dd, J = 9.0, 5.1 Hz, 1H); ^{13}C NMR (100 MHz, $CDCl_3$) δ 171.4, 170.4, 167.6, 137.3, 133.0, 131.5, 130.5, 129.8, 127.9, 52.9, 52.4, 36.2, 33.4, 19.91; HRMS (ESI) *m/z* Calcd for $C_{14}H_{14}O_6Na$ $[M + Na]^+$ 301.0688, found 301.0683.

Tips: *The acidification is important to transfer the salt to benzoic acid, which can be extracted from the aqueous solution by EtOAc. After work-up, a yellowish solid is generally obtained, which could be easily purified by washing portion wise with hexane.*

2′,3′,4′,6′-Tetra-*O*-benzoyl-D-glucopyranosyl *ortho*-2,2-dimethoxycarbony-lcyclopropylbenzoate (1a)

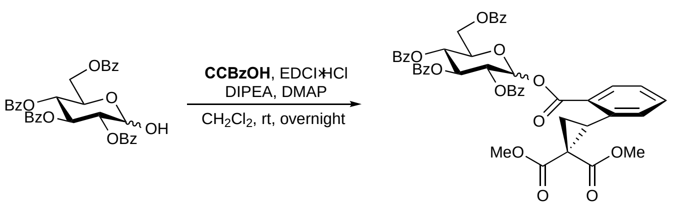

Supplementary Fig. 6 | Synthesis of 1a.

To a solution of 2,3,4,6-tetra-*O*-benzoyl-D-glucopyranose[2] (1.19 g, 2.0 mmol, 1.0 equiv), **CCBzOH** (668 mg, 2.4 mmol, 1.2 equiv) and DMAP (24 mg, 0.2 mmol, 0.1 equiv) in anhydrous CH_2Cl_2 (10 mL) were added sequentially EDC·HCl (575 g, 3.0 mmol, 1.5 equiv) and DIPEA (0.7 mL, 4.0 mmol, 2.0 equiv). The mixture was stirred at room temperature overnight. The resulting mixture was washed sequentially with 1 M HCl solution and sat. $NaHCO_3$ solution, the organic layers were combined, dried over anhydrous Na_2SO_4, filtered and concentrated *in vauco*. The crude product was purified by silica gel column chromatography (toluene: EtOAc = 20:1) to afford donor **1a** (1.25 g, 1.6 mmol, 80%, α_1:α_2:β_1:β_2 = 1.0:1.3:1.3:1.5) as a white foam. ^{1}H NMR (400 MHz, $CDCl_3$) δ 8.14 (ddd, J = 7.6, 6.0, 1.5 Hz, 2.8H), 8.10 – 8.01 (m, 12.9H), 7.97 – 7.84 (m, 34.5H), 7.59 – 7.26 (m, 82.8H), 7.21 (d, J = 7.7 Hz, 3.2H), 6.84 (d, J = 3.8 Hz, 1H), 6.82 (d, J = 3.7 Hz, 1.3H), 6.40 (d, J = 8.1 Hz, 1.3H), 6.35 – 6.26 (m, 4H), 6.05 (td, J = 9.5, 6.2 Hz, 2.9H), 5.97 – 5.78 (m, 8.5H), 5.71 (ddd, J = 10.2, 5.3, 3.7 Hz, 2.4H), 4.73 – 4.63 (m, 6.9H), 4.60 (dt, J = 10.2, 3.6 Hz, 1.2H), 4.55 – 4.38 (m, 8.4H), 3.87 – 3.68 (m, 22.6H), 3.30 (s, 3.9H), 3.25 (s, 3.9H), 3.02 (s, 3H), 2.80 (s, 4.4H), 2.21 – 2.08 (m, 5.5H), 1.87 – 1.72 (m, 9.2H); ^{13}C NMR (100 MHz, $CDCl_3$) δ 170.4, 170.3, 170.1, 167.5, 167.22, 167.19, 166.3, 166.2, 166.1, 165.82, 165.77, 165.5, 165.4, 165.32, 165.28, 165.25, 165.2, 164.1, 163.9, 163.79, 137.78, 137.6, 137.54, 137.51, 133.63, 133.59, 133.50, 133.47, 133.4, 133.31, 133.25, 133.2, 133.1, 133.0, 132.9, 132.8, 131.6, 131.1, 130.8, 130.6, 130.3, 129.97, 129.95, 129.92, 129.89, 129.8, 129.73, 129.67, 129.6, 129.4, 129.1, 129.0, 128.9, 128.83, 128.79, 128.6, 128.52, 128.48, 128.46, 128.0, 127.9, 92.6, 92.4, 90.0, 73.21, 73.16, 73.04, 73.02, 71.2, 70.9, 70.8, 70.7, 70.6, 70.5, 70.3, 69.4, 69.3, 68.9, 68.8, 63.0, 62.9, 62.5, 53.0, 52.93, 52.89, 52.85, 52.5, 52.2, 52.0,

51.7, 36.6, 36.4, 36.3, 35.7, 32.6, 32.4, 32.2, 19.80, 19.75, 19.5; HRMS (ESI) *m/z* Calcd for $C_{48}H_{40}O_{15}Na$ [M + Na]$^+$ 879.2265, found 879.2263.

Tips: *In most cases, the glycosyl donors are easy to separate by toluene and EtOAc eluent system. Alternatively, if the product is difficult to separate from the unreacted substrate. It is suggested to add a small amount of* Ac_2O *to facilitate the acetylation of the unreacted anomeric hemiacetal. With such small modification from standard operation, the anomeric acetate is easier to remove by silica gel column chromatography by hexane and EtOAc eluent system.*

2′,3′,4′,6′-Tetra-*O*-benzoyl-D-galactopyranosyl *ortho*-2,2-dimethoxycarbonylcyclopropylbenzoate (1b)

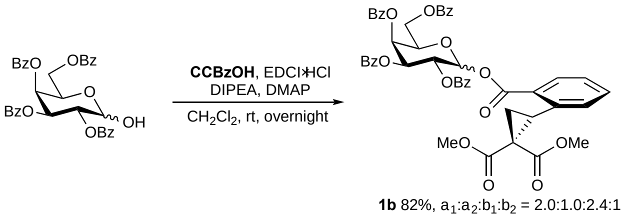

Supplementary Fig. 7 | Synthesis of 1b.

Following the procedure for **1a**, 2,3,4,6-tetra-*O*-benzoyl-D-galactopyranose[3] (596.6 mg, 1.0 mmol, 1.0 equiv) was transformed into **1b** (701.3 mg, 0.82 mmol, 82%, α_1:α_2:β_1:β_2 = 2.0:1.0:2.4:1.0) as a white foam after purification by silica gel column chromatography (toluene:EtOAc = 20:1). ^{1}H NMR (400 MHz, CD_2Cl_2) δ 8.28 – 7.75 (m, 47.8H), 7.75 – 7.21 (m, 83.7H), 6.92 (dd, *J* = 10.0, 3.7 Hz, 1.5H), 6.78 (d, *J* = 12.4 Hz, 0.7H), 6.45 (d, *J* = 8.2 Hz, 1H), 6.34 (d, *J* = 8.3 Hz, 1.2H), 6.24 – 6.01 (m, 10.2H), 5.91 – 5.73 (m, 4.1H), 5.56 (dd, *J* = 8.8, 3.3 Hz, 0.7H), 5.02 – 4.42 (m, 14.8H), 3.87 – 3.63 (m, 25.8H), 3.31 (s, 2.9H), 3.25 (s, 3H), 3.00 (s, 1.7H), 2.81 (s, 3.6H), 2.28 – 2.08 (m, 6H), 1.85 – 1.73 (m, 5.5H); ^{13}C NMR (100 MHz, CD_2Cl_2) δ170.53, 170.50, 170.43, 170.35, 170.0, 169.5, 169.4, 167.6, 167.4, 167.3, 166.3, 166.23, 166.21, 166.1, 166.01, 165.97, 165.94, 165.90, 165.8, 165.7, 164.9, 164.5, 164.4, 164.3, 164.2, 138.2, 138.04, 137.96, 137.9, 137.3, 134.6, 134.1, 133.94, 133.86, 133.84, 133.79, 133.74, 133.68, 133.63, 133.59, 133.5, 133.4, 133.3, 133.2, 133.1, 133.0, 131.7, 131.3, 131.2, 131.1,

130.8, 130.7, 130.4, 130.30, 130.26, 130.22, 130.20, 130.13, 130.08, 130.04, 129.99, 129.94, 129.90, 129.88, 129.8, 129.7, 129.60, 129.57, 129.55, 129.48, 129.46, 129.44, 129.39, 129.32, 129.28, 129.26, 129.1, 128.94, 128.88, 128.86, 128.83, 128.81, 128.78, 128.76, 128.2, 128.1, 127.9, 126.4, 126.0, 122.5, 100.4, 100.1, 93.3, 93.1, 91.0, 90.9, 84.8, 84.7, 81.3, 79.0, 78.0, 77.83, 77.78, 72.83, 72.76, 72.2, 72.1, 70.8, 69.8, 69.7, 69.6, 69.20, 69.15, 69.1, 68.6, 68.3, 68.1, 64.0, 62.4, 62.3, 62.3, 62.2, 53.2, 53.12, 53.09, 53.02, 52.99, 52.5, 52.4, 52.3, 52.1, 51.8, 48.1, 36.9, 36.7, 36.58, 36.55, 36.2, 34.2, 32.6, 32.5, 32.3, 19.9, 19.8, 19.7; HRMS (ESI) *m/z* Calcd for $C_{48}H_{40}O_{15}Na$ $[M + Na]^+$ 879.2265, found 879.2263.

2′,3′,4′,6′-Tetra-*O*-benzoyl-D-mannopyranosyl *ortho*-2,2-dimethoxycarbonylcyclopropylbenzoate (1c)

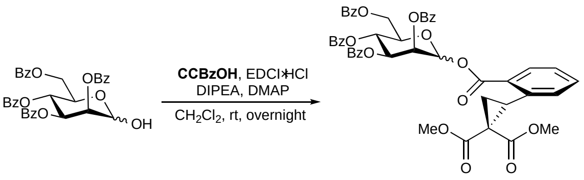

Supplementary Fig. 8 | Synthesis of 1c.

Following the procedure for **1a**, 2,3,4,6-tetra-*O*-benzoyl-D-mannopyranose[4] (477 mg, 0.8 mmol, 1.0 equiv) was transformed into **1c** (694 mg, 0.704 mmol, 88%, $\alpha_1:\alpha_2:\beta_1:\beta_2$ = 8.9:8.9:1.2:1.0) as a white foam after purification by silica gel column chromatography (hexane:EtOAc = 3:1 to 2.5:1). ^1H NMR (400 MHz, $CDCl_3$) δ 8.22 – 7.04 (m, 66.3H), 6.61 (d, J = 15.1 Hz, 2H), 6.46 (d, J = 6.8 Hz, 0.2H), 6.41 – 5.89 (m, 7H), 5.83 (dd, J = 9.4, 5.3 Hz, 0.3H), 5.64 – 5.50 (m, 0.2H); 4.87 – 4.72 (m, 2.5H), 4.63 (dt, J = 20.3, 10.3 Hz, 2.5H), 4.56 – 4.46 (m, 2.2H), 4.44 – 4.33 (m, 0.3H), 4.17 – 4.06 (m, 0.2H), 3.97 – 3.64 (m, 12.2H), 3.41 (s, 3H), 3.36 – 3.26 (m, 3.7H), 3.20 (s, 0.4H), 2.86 – 2.74 (m, 0.2H), 2.31 – 2.11 (m, 2.9H), 1.98 – 1.76 (m, 3.7H); ^{13}C NMR (100 MHz, $CDCl_3$) δ170.3, 170.22, 170.15, 169.9, 169.2, 169.1, 167.34, 167.29, 167.16, 166.17, 166.1, 165.8, 165.7, 165.6, 165.39, 165.37, 165.2, 165.1, 163.7, 163.5, 163.2, 137.7, 137.4, 137.30, 134.31, 133.7, 133.6, 133.54, 133.49, 133.4, 133.22, 133.15, 133.12, 133.08, 132.91, 132.85, 131.1, 130.8, 130.54, 130.51, 130.04, 130.00, 129.93,

129.88, 129.84, 129.80, 129.7, 129.6, 129.5, 129.4, 129.3, 129.2, 128.99, 128.97, 128.93, 128.90, 128.85, 128.7, 128.62, 128.57, 128.53, 128.50, 128.4, 128.3, 128.04, 128.01, 127.7, 126.0, 125.9, 122.1, 91.8, 91. 6, 90.8, 73.32, 73.26, 71.9, 71.6, 71.5, 71.0, 70.2, 70.1, 69.7, 69.6, 69.4, 66.52, 66.49, 66.4, 66.3, 62.8, 62.4, 60.4, 53.04, 52.99, 52.96, 52.90, 52.85, 52.50, 52.45, 52.3, 52.1, 47.7, 36.24, 36.19, 35.8, 32.6, 32.4, 32.3, 19.8, 19.5, 14.3, 14.2; HRMS (ESI) *m/z* Calcd for $C_{48}H_{40}O_{15}Na$ $[M + Na]^+$ 879.2265, found 879.2263.

3′,4′,6′-Tri-*O*-acetyl-2′-deoxy-2′-phthalimido-D-glucopyranosyl *ortho*-2,2-dimethoxycarbonylcyclopropylbenzoate (1d)

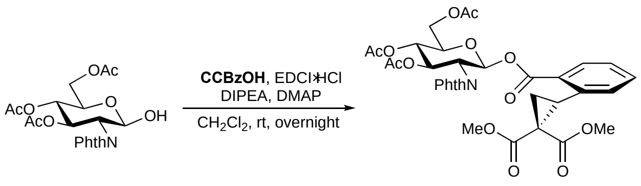

1d 80%, b1:b2 = 1.4:1

Supplementary Fig. 9 | Synthesis of 1d.

Following the procedure for **1a**, 3,4,6-tri-*O*-acetyl-2-deoxy-2-phthalimido-D-glucopyranose[5] (218 mg, 0.5 mmol, 1.0 equiv) was transformed into **1d** (278.7 mg, 0.4 mmol, 80%, β_1:β_2 = 1.4:1.0) as a white foam after purification by silica gel column chromatography (hexane:EtOAc:$CHCl_3$ = 2:2:1). ^1H NMR (400 MHz, $CDCl_3$) δ 7.86 (dd, J = 7.9, 1.4 Hz, 1.4H), 7.83 – 7.73 (m, 6H), 7.72 – 7.61 (m, 5H), 7.42 – 7.33 (m, 2.5H), 7.29 – 7.20 (m, 3.3H), 7.19 – 7.10 (m, 2.5H), 6.73 – 6.65 (m, 2.4H), 6.05 – 5.92 (m, 2.4H), 5.29 – 5.17 (m, 2.4H), 4.71 – 4.57 (m, 2.4H), 4.43 – 4.31 (m, 2.5H), 4.17 – 4.05 (m, 5H), 3.83 (s, 4.1H), 3.77 (s, 3H), 3.69 (t, J = 8.6 Hz, 1.4H), 3.61 (t, J = 8.7 Hz, 1H), 3.27 (s, 3H), 2.58 (s, 4.2H), 2.12 – 1.95 (m, 18.5H), 1.89 – 1.83 (m, 7.6H), 1.77 – 1.66 (m, 2.6H); ^{13}C NMR (100 MHz, $CDCl_3$) δ 170.72, 170.67, 170.2, 170.11, 170.07, 170.0, 169.6, 167.53, 167.45, 167.3, 166.5, 163.9, 163.2, 137.7, 137.4, 134.5, 134.1, 132.9, 132.7, 131.2, 130.9, 130.8, 130.0, 129.8, 129.5, 129.2, 127.8, 127.7, 123.8, 123.4, 90.0, 89.9, 72.52, 72.50, 70.6, 70.5, 68.7, 61.8, 61.7, 53.7, 53.4, 52.8, 52.7, 52.3, 51.2,

36.2, 35.4, 32.3, 32.0, 20.8, 20.68, 20.65, 20.5, 19.7, 19.6; HRMS (ESI) *m/z* Calcd for $C_{34}H_{33}O_{15}NNa$ [M + Na]$^+$ 718.1748, found 718.1738.

2′,3′,4′-Tri-*O*-benzoyl-D-xylopyranosyl *ortho*-2,2-dimethoxycarbonylcyclopropylbenzoate (1e)

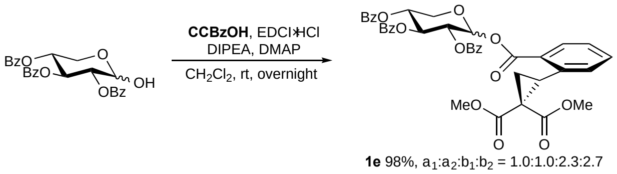

Supplementary Fig. 10 | Synthesis of 1e.

Following the procedure for **1a**, 2,3,4-tri-*O*-benzoyl-D-xylopyranose[2] (231 mg, 0.5 mmol, 1.0 equiv) was transformed into **1e** (354 mg, 0.49 mmol, 98%, α_1:α_2:β_1:β_2 = 1.0:1.0:2.3:2.7) as a white foam after purification by silica gel column chromatography (hexane:EtOAc = 3:1). ^{1}H NMR (400 MHz, $CDCl_3$) δ 8.16 – 8.08 (m, 1H), 8.07 – 7.87 (m, 19.6H), 7.73 – 7.65 (m, 0.7H), 7.60 – 7.16 (m, 37.2H), 6.77 (d, *J* = 3.6 Hz, 0.4H), 6.74 (d, *J* = 3.7 Hz, 0.4H), 6.40 (d, *J* = 4.5 Hz, 0.8H), 6.36 (d, *J* = 4.7 Hz, 1H), 6.28 – 6.21 (m, 0.8H), 5.89 – 5.80 (m, 2H), 5.69 – 5.61 (m, 1.9H), 5.61 – 5.50 (m, 2.3H), 5.42 – 5.33 (m, 2H), 4.61 – 4.51 (m, 1.9H), 4.36 – 4.25 (m, 0.8H), 4.16 – 3.94 (m, 3.3H), 3.89 – 3.65 (m, 15.7H), 3.34 – 3.29 (m, 3.7H), 3.04 – 2.98 (m, 4.3H), 2.87 – 2.75 (m, 0.7H), 2.27 – 2.11 (m, 3.6H), 1.89 – 1.81 (m, 1.8H), 1.80 – 1.71 (m, 1.7H); ^{13}C NMR (100 MHz, $CDCl_3$) δ 170.3, 170.2, 170.1, 170.0, 169.9, 169.21, 169.15, 167.3, 167.2, 166.0, 165.64, 165.61, 165.59, 165.5, 165.4, 165.2, 165.1, 164.16, 164.15, 164.1, 164.0, 137.5, 137.4, 137.2, 134.3, 133.61, 133.57, 133.53, 133.49, 132.9, 132.8, 132.7, 131.2, 130.87, 130.85, 130.4, 130.3, 130.2, 130.14, 130.05, 130.01, 129.97, 129.95, 129.92, 129.88, 129.8, 129.7, 129.5, 129.4, 129.23, 129.21, 129.10, 129.06, 129.04, 129.00, 128.98, 128.9, 128.8, 128.6, 128.53, 128.51, 128.48, 127.98, 127.95, 127.73, 127.67, 126.02, 125.98, 122.1, 92.3, 92.0, 90.4, 90.1, 70.5, 70.3, 70.2, 69.7, 69.6, 69.2, 69.1, 68.8, 68.6, 68.3, 62.0, 61.9, 61.5, 61.3, 53.0, 52.93, 52.91, 52.88, 52.85, 52.3, 52.2, 52.00, 51.97, 36.4, 36.24, 36.19, 34.0, 32.6, 32.5, 32.2, 32.1, 19.8, 19.7, 19.5; HRMS (ESI) *m/z* Calcd for $C_{40}H_{34}O_{13}Na$ [M + Na]$^+$ 745.1897, found 745.1907.

2′,3′,4′-Tri-*O*-benzoyl-L-rhamnopyranosyl *ortho*-2,2-dimethoxycarbonylcyclopropylbenzoate (1f)

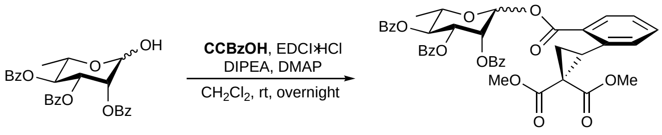

Supplementary Fig. 11 | Synthesis of 1f.

Following the procedure for **1a**, 2,3,4-tri-*O*-benzoyl-L-rhamnopyranose[6] (238 mg, 0.5 mmol, 1.0 equiv) was transformed into **1f** (343 mg, 0.465 mmol, 93%, α_1:α_2:β_1:β_2 = 5.0:4.5:1.0:1.7) as a white foam after purification by silica gel column chromatography (hexane:EtOAc = 3:1). ^{1}H NMR (400 MHz, $CDCl_3$) δ 8.23 – 8.05 (m, 7.4H), 8.04 – 7.93 (m, 5.4H), 7.93 – 7.88 (m, 0.5H), 7.88 – 7.72 (m, 6.1H), 7.72 – 7.60 (m, 3.2H), 7.60 – 7.20 (m, 31.1H), 7.14 (dt, J = 15.1, 7.5 Hz, 0.8H), 6.57 – 6.49 (m, 1.9H), 6.39 – 6.33 (m, 0.5H), 6.11 – 6.02 (m, 0.6H), 6.00 – 5.84 (m, 4H), 5.84 – 5.68 (m, 3.3H), 5.59 – 5.50 (m, 0.3 H), 4.44 – 4.32 (m, 2.0H), 4.14 – 4.03 (m, 0.7H), 3.96 – 3.76 (m, 11.6H), 3.75 – 3.70 (m, 0.4 H), 3.68 (s, 0.9H), 3.40 (s, 3H), 3.36 (s, 2.7H), 3.30 (s, 1.6H), 2.87 – 2.75 (m, 0.3H), 2.24 – 2.14 (m, 2.9H), 1.94 – 1.81 (m, 2.9H), 1.51 – 1.31 (m, 8H); ^{13}C NMR (100 MHz, $CDCl_3$) δ 170.3, 170.1, 169.22, 169.16, 167.4, 167.3, 165.79, 165.75, 165.71, 165.65, 165.5, 165.4, 165.3, 163.8, 163.6, 163.4, 137.8, 137.6, 137.3, 134.4, 133.7, 133.63, 133.59, 133.5, 133.4, 133.0, 132.9, 132.8, 132.7, 131.1, 130.9, 130.8, 130.7, 130.5, 130.1, 130.04, 129.88, 129.85, 129.81, 129.79, 129.6, 129.5, 129.28, 129.25, 129.23, 129.19, 129.11, 129.08, 128.9, 128.8, 128.7, 128.60, 128.56, 128.4, 128.00, 127.97, 127.7, 126.0, 122.1, 91.64, 91.56, 90.7, 78.6, 71.8, 71.43, 71.40, 70.0, 69.91, 69.85, 69.7, 69.6, 69.3, 53.04, 53.01, 52.99, 52.94, 52.85, 52.6, 52.5, 52.3, 52.2, 47.8, 36.33, 36.26, 36.2, 36.11, 34.06, 32.6, 32.5, 32.3, 19.8, 19.6, 17.9, 17.8, 17.7; HRMS (ESI) *m/z* Calcd for $C_{41}H_{36}O_{13}Na$ [M + Na]$^+$ 759.2054, found 759.2044.

2′,3′,5′-Tri-*O*-acetyl-D-ribofuranosyl *ortho*-2,2-dimethoxycarbonylcyclopropybenzoate (1g)

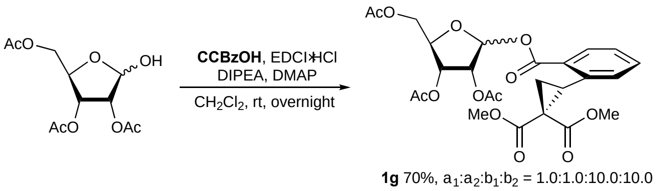

Supplementary Fig. 12 | Synthesis of 1g.

Following the procedure for **1a**, 2,3,5-tri-*O*-acetyl-D-ribofluranose[7] (793 mg, 2.9 mmol, 1.0 equiv) was transformed into **1g** (1.08 g, 2.03 mmol, 70%, α_1:α_2:β_1:β_2 = 1.0:1.0:10.0:10.0) as a white foam after purification by silica gel column chromatography (hexane:EtOAc = 2:1). ^{1}H NMR (400 MHz, $CDCl_3$) δ 8.05 – 7.97 (m, 0.3H), 7.91 (d, J = 7.8 Hz, 2H), 7.52 – 7.43 (m, 2.3H), 7.38 – 7.30 (m, 2.3H), 7.28 – 7.22 (m, 4.8H), 6.69 – 6.65 (m, 0.2H), 6.41 (s, 1H), 6.36 (s, 1H), 5.51 (d, J = 4.8 Hz, 1H), 5.50 – 5.43 (m, 3H), 5.37 – 5.30 (m, 0.5H), 4.57 – 4.52 (m, 0.3H), 4.45 – 4.32 (m, 4.4H), 4.20 – 4.11 (m, 2H), 3.81 – 3.75 (d, J = 4.0 Hz, 8.2H), 3.31 – 3.28 (m, 6H), 2.19 – 2.12 (m, 8.9H), 2.12 – 2.04 (m, 8H), 2.01 (s, 0.3H), 1.99 (s, 0.3H), 1.96 (s, 3H), 1.90 (s, 3H), 1.87 – 1.76 (m, 3.6H); ^{13}C NMR (100 MHz, $CDCl_3$) δ 170.59, 170.55, 170.23, 170.16, 169.8, 169.5, 169.4, 167.30, 167.25, 164.5, 164.2, 137.4, 136.8, 132.83, 132.76, 131.0, 130.7, 130.4, 130.1, 129.7, 127.7, 99.2, 98.9, 79.3, 74.4, 74.2, 70.6, 70.5, 63.7, 63.6, 53.0, 52.9, 52.32, 52.30, 36.4, 36.1, 32.5, 32.1, 20.7, 20.61, 20.56, 19.8, 19.7; HRMS (ESI) *m/z* Calcd for $C_{25}H_{28}O_{13}Na$ $[M + Na]^+$ 559.1428, found 559.1425.

2′,3′,5′-Tri-*O*-benzoyl-4-(2,3,4,6-tetra-*O*-benzoyl-β-D-galactopyranosyl)-D-glucopyranosyl *ortho*-2,2-dimethoxycarbonylcyclopropylbenzoate (1h)

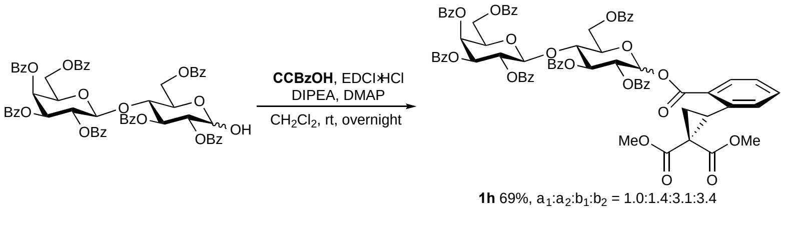

Supplementary Fig. 13 | Synthesis of 1h.

Following the procedure for **1a**, 2,3,5-tri-*O*-benzoyl-4-(2,3,4,6-tetra-*O*-benzoyl-β-D-galactopyranosyl)-D-glucopyranose[8] (504 mg, 0.5 mmol, 1.0 equiv) was transformed into **1h** (460.5 mg, 0.345 mmol, 69%, α_1:α_2:β_1:β_2 = 1.0:1.4:3.1:3.4) as a white foam

after purification by silica gel column chromatography (hexane:EtOAc = 2:1). ^{1}H NMR (400 MHz, $CDCl_3$) δ 8.17 – 7.84 (m, 42.3H), 7.74 (dd, *J* = 6.1, 3.7 Hz, 6.8H), 7.68 – 7.11 (m, 80.6H), 6.74 (d, *J* = 3.8 Hz, 0.3H), 6.69 (d, *J* = 3.8 Hz, 0.4H), 6.25 (d, *J* = 8.2 Hz, 1H), 6.14 (d, *J* = 8.1 Hz, 1H), 6.02 – 5.90 (m, 2H), 5.87 – 5.71 (m, 8.5H), 5.70 – 5.59 (m, 0.8H), 5.48 – 5.33 (m, 3H), 5.00 – 4.88 (m, 3H), 4.71 – 4.49 (m, 6.3H), 4.49 – 4.35 (m, 3.7H), 4.34 – 4.26 (m, 0.4H), 4.17 – 4.03 (m, 2.5H), 3.96 – 3.87 (m, 3.4H), 3.87 – 3.64 (m, 19H), 3.17 (s, 3H), 2.98 – 2.93 (m, 2.5H), 2.76 (s, 3.3H), 2.16 – 2.02 (m, 4H), 1.84 – 1.70 (m, 3.7H); ^{13}C NMR (100 MHz, $CDCl_3$) *δ* 170.3, 170.2, 170.1, 170.0, 167.4, 167.19, 167.15, 166.9, 166.0, 165.9, 165.8, 165.7, 165.6, 165.51, 165.48, 165.42, 165.39, 165.32, 165.29, 165.0, 164.92, 164.88, 164.2, 163.8, 163.7, 163.5, 101.4, 101.2, 101.1, 92.5, 92.2, 89.8, 75.8, 75.5, 73.88, 73.85, 73.0, 72.1, 71.9, 71.5, 71.2, 71.1, 70.7, 70.5, 70.3, 69.99, 69.95, 67.6, 62.3, 62.1, 61.1, 52.9, 52.82, 52.76, 52.3, 51.9, 51.8, 51.7, 36.6, 36.3, 36.2, 35.55, 32.58, 32.4, 32.1, 19.7, 19.3; HRMS (ESI) *m/z* Calcd for $C_{75}H_{62}O_{23}Na$ $[M + Na]^+$ 1353.3580, found 1353.3580.

Section 2. Reaction development of strain-release glycosylation.

Supplementary Table 1 | Reaction development.[a]

reaction development and control experiment

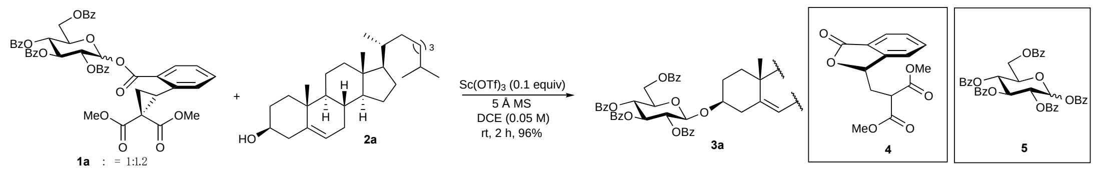

Entry	Derivation from the standard condition	Yields of **3a** (%)	Yields of recovered donor (%)	Yields of **4** (%)
1	None	96	-	99
2	No $Sc(OTf)_3$	0	>95	0
3	$Bi(OTf)_3$ as the catalyst	65	40	58
4	$Zn(OTf)_2$ or $Ca(OTf)_2/^{n}Bu_4NPF_6$ or $B(C_6F_5)_3$ as catalyst	0	>95	0
5	TfOH as catalyst	complex mixture	-	NA
6	TMSOTf as catalyst	<5	>95	<5
7	$Ph_3PAuNTf_2$	0	>95	0
8	4 Å MS instead of 5 Å MS	33	68	30
9	CH_2Cl_2 as the solvent	99	-	99
10	$PhCH_3$ or $PhCF_3$ or Et_2O as solvent	96-97	-	>95
11[b]	glucosyl pentabenzoate **5** as donor	trace	>95	NA

[a]Unless otherwise specified, all reactions were performed with 1.2 equiv of **1a**, 1.0 equiv of **2a** (0.05 mmol) in the presence of catalyst (0.1 equiv) and 5 Å MS in corresponding solvent (0.05 M, 1 mL) for 2-5 h at room temperature. The yield for **3a** was based on **2a**, and the yields for **4** and recovered donor were based on the **1a**. [b]CH_2Cl_2 was used as the solvent. DCE = 1,2-dichloroethane, NR = no reaction, NA = not applicable.

General procedure for the reaction development.

A solution of **1a** (25.7 mg, 30 μmol, 1.2 equiv) and cholesterol **2a** (9.7 mg, 25 μmol, 1.0 equiv) in anhydrous solvent (0.5 mL) containing freshly activated molecular sieve was stirred at room temperature for 15 min before the catalyst (2.5 μmol, 0.1 equiv) was added. The mixture was stirred at room temperature for 2-5 h until **2a** was fully consumed or reaction ceased. The reaction was then quenched with triethylamine and the mixture was directly loaded onto silica gel by concentrating the mixture *in vauco*. The residue was further purified by silica gel column chromatography (hexane:EtOAc = 10:1) to give the **3a** as colorless syrup, followed by changing the eluent system (toluene:EtOAc = 20:1) to afford **4** as a colorless oil as well as recover the unreacted glycosyl donor **1a** as a white foam, respectively. The yield for **3a** was based on **2a** and the yields for **4** and recovered donor were based on **1a**. **3a**: ^1H NMR (400 MHz, $CDCl_3$) δ 8.01 (d, J = 7.7 Hz, 2H), 7.96 (d, J = 7.7 Hz, 2H), 7.91 (d, J = 7.7 Hz, 2H), 7.84 (d, J = 7.7 Hz, 2H), 7.56 – 7.46 (m, 3H), 7.46 – 7.26 (m, 9H), 5.90 (t, J = 9.6 Hz, 1H), 5.63 (t, J = 9.7 Hz, 1H), 5.50 (dd, J = 9.8, 7.9 Hz, 1H), 5.23 (d, J = 5.0 Hz, 1H), 4.95 (d, J = 7.9 Hz, 1H), 4.61 (dd, J = 12.0, 3.4 Hz, 1H), 4.53 (dd, J = 12.0, 5.9 Hz, 1H), 4.16 (ddd,

J = 9.6, 5.9, 3.4 Hz, 1H), 3.54 (dt, *J* = 11.0, 5.6 Hz, 1H), 2.22 – 2.10 (m, 2H), 2.04 – 0.72 (m, 57H), 0.66 (s, 3H). The data are identical with the literature report.[9] **4**: ^{1}H NMR (400 MHz, $CDCl_3$) δ 7.87 (d, *J* = 7.6 Hz, 1H), 7.68 (t, *J* = 7.3 Hz, 1H), 7.53 (t, *J* = 7.5 Hz, 1H), 7.50 – 7.43 (m, 1H), 5.52 (dd, *J* = 9.3, 3.2 Hz, 1H), 3.78 (s, 3H), 3.72 – 3.63 (m, 4H), 2.77 (ddd, *J* = 14.8, 9.6, 3.2 Hz, 1H), 2.17 (ddd, *J* = 14.5, 9.2, 4.9 Hz, 1H); ^{13}C NMR (100 MHz, $CDCl_3$) δ 169.9, 169.2, 169.1, 148.8, 134.3, 129.6, 126.0, 125.9, 122.1, 78.6, 53.0, 52.9, 47.7, 34.0; HRMS (ESI) *m/z* Calcd for $C_{14}H_{14}O_6Na$ [M + Na]$^+$ 301.0688, found 301.0687.

Section 3. Strain-release glycosylation with 1a as the donor.

Acceptors **2a-d**, **m-r** were commercially available. Acceptors **2e,**[10] **2f,**[10] **2g,**[11] **2h,**[10] **2i,**[12] **2j,**[13] **2k,**[14] **2l,**[15] and **2s**[8] were prepared according to the reported literatures.

(3β)-Cholest-5-en-3-yl 2′,3′,4′,6′-tetra-*O*-benzoyl-β-D-glucopyranoside (3a)

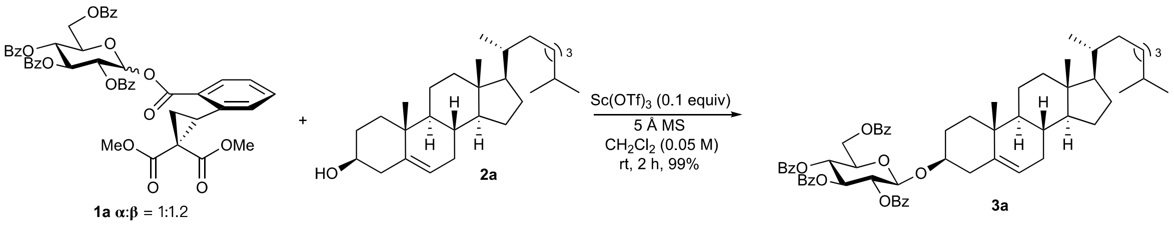

Supplementary Fig. 14 | Synthesis of 3a.

A solution of **1a** (25.7 mg, 30 μmol, 1.2 equiv) and **2a** (9.7 mg, 25 μmol, 1.0 equiv) in anhydrous CH_2Cl_2 (0.5 mL) containing freshly activated 5 Å molecular sieve was stirred at room temperature for 15 min before $Sc(OTf)_3$ (1.25 mg, 2.5 μmol, 0.1 equiv) was added. The mixture was stirred at room temperature for 2 h before the reaction was quenched by triethylamine. The mixture was directly loaded onto silica gel by concentrating the mixture *in vauco* and the residue was further purified by silica gel column chromatography (hexane:EtOAc = 10:1) to afford **3a** (26.0 mg, 25 μmol, 99%) as colorless syrup.

4-Pentenyl 2′,3′,4′,6′-tetra-*O*-benzoyl-β-D-glucopyranoside (3b)

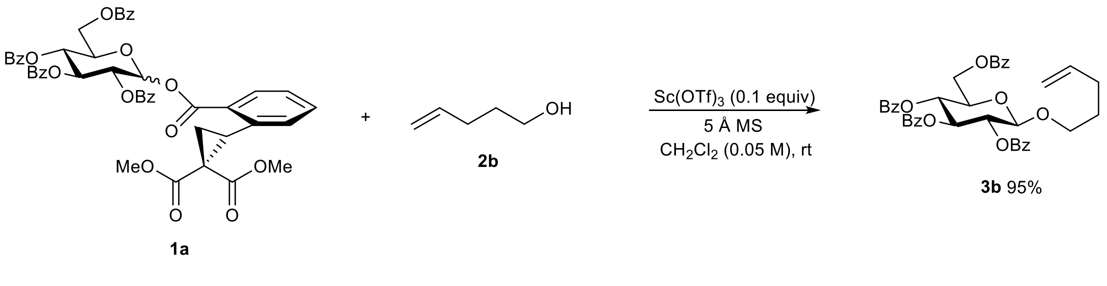

Supplementary Fig. 15 | Synthesis of 3b.

Following the procedure for **3a**, **2b** (5.16 μL, 50 μmol, 1.0 equiv) was transformed into **3b** (31.5 mg, 47.5 μmol, 95%) as a colorless syrup after purification by silica gel column chromatography (hexane:EtOAc = 9:1). ^{1}H NMR (400 MHz, $CDCl_3$) δ 8.05 –

7.99 (m, 2H), 7.99 – 7.94 (m, 2H), 7.93 – 7.87 (m, 2H), 7.86 – 7.81 (m, 2H), 7.57 – 7.46 (m, 3H), 7.45 – 7.26 (m, 9H), 5.91 (t, *J* = 9.6 Hz, 1H), 5.73 – 5.59 (m, 2H), 5.53 (dd, *J* = 9.8, 7.8 Hz, 1H), 4.88 – 4.78 (m, 3H), 4.64 (dd, *J* = 12.1, 3.3 Hz, 1H), 4.51 (dd, *J* = 12.1, 5.2 Hz, 1H), 4.16 (ddd, *J* = 9.9, 5.3, 3.3 Hz, 1H), 3.93 (dt, *J* = 9.7, 6.2 Hz, 1H), 3.56 (dt, *J* = 9.7, 6.6 Hz, 1H), 2.07 – 1.89 (m, 2H), 1.73 – 1.60 (m, 2H). The data are identical with the literature.[9]

D-Menthyl 2,3,4,6-tetra-*O*-benzoyl-β-D-glucopyranoside (3c)

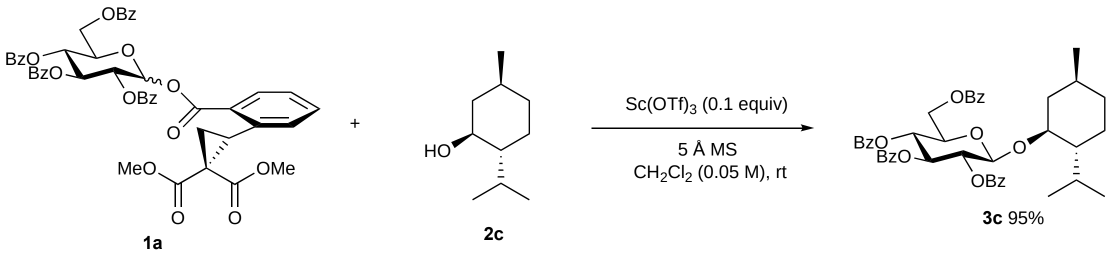

Supplementary Fig. 16 | Synthesis of 3c.

Following the procedure for **3a**, **2c** (7.8 mg, 50 mmol, 1.0 equiv) was transformed into **3c** (34.8 mg, 47.5 μmol, 95%) as a colorless syrup after purification by silica gel column chromatography (hexane:EtOAc = 7:1). ^{1}H NMR (400 MHz, $CDCl_3$) δ 8.02 (d, *J* = 7.0 Hz, 2H), 7.95 (d, *J* = 7.0 Hz, 2H), 7.92 (d, *J* = 8.2 Hz, 2H), 7.83 (d, *J* = 8.0 Hz, 2H), 7.56 – 7.47 (m, 3H), 7.44 – 7.32 (m, 7H), 7.31 – 7.26 (m, 2H), 5.92 (t, *J* = 9.7 Hz, 1H), 5.62 – 5.53 (m, 2H), 4.89 (d, *J* = 7.9 Hz, 1H), 4.63 (dd, *J* = 12.0, 3.1 Hz, 1H), 4.50 (dd, *J* = 12.0, 6.8 Hz, 1H), 4.24 – 4.15 (m, 1H), 3.39 – 3.29 (m, 1H), 2.22 (d, *J* = 12.7 Hz, 1H), 1.94 – 1.82 (m, 1H), 1.67 – 1.47 (m, 3H), 1.21 – 1.05 (m, 2H), 0.91 – 0.79 (m, 2H), 0.78 (d, *J* = 6.4 Hz, 3H), 0.47 (d, *J* = 7.0 Hz, 3H), 0.40 (d, *J* = 6.9 Hz, 3H). The data are identical with the literature.[16]

1-Adamantyl 2′,3′,4′,6′-tetra-*O*-benzoyl-β-D-glucopyranoside (3d)

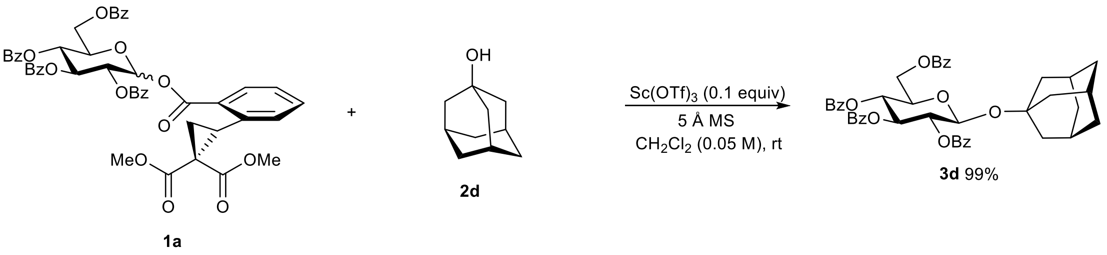

Supplementary Fig. 17 | Synthesis of 3d.

Following the procedure for **3a**, **2d** (7.6 mg, 50 µmol, 1.0 equiv) was transformed into **3d** (36.3 mg, 49.5 µmol, 99%) as a white foam after purification by silica gel column chromatography (hexane:EtOAc = 7:1). ^{1}H NMR (400 MHz, CDCl$_3$) δ 8.05 – 7.99 (m, 2H), 7.98 – 7.94 (m, 2H), 7.94 – 7.90 (m, 2H), 7.87 – 7.80 (m, 2H), 7.57 – 7.47 (m, 3H), 7.45 – 7.32 (m, 7H), 7.29 (d, J = 7.8 Hz, 2H), 5.93 (t, J = 9.6 Hz, 1H), 5.56 (t, J = 9.7 Hz, 1H), 5.50 (dd, J = 9.8, 7.9 Hz, 1H), 5.13 (d, J = 7.9 Hz, 1H), 4.59 (dd, J = 11.9, 3.1 Hz, 1H), 4.49 (dd, J = 11.9, 7.1 Hz, 1H), 4.19 (ddd, J = 10.1, 7.1, 3.1 Hz, 1H), 2.02 (s, 3H), 1.83 (d, J = 11.7 Hz, 3H), 1.65 (d, J = 11.3 Hz, 4H), 1.60 – 1.45 (m, 6H). The data are identical with the literature.[17]

Methyl 6-*O*-(2,3,4,6-tetra-*O*-benzoyl-β-D-glucopyranosyl)-2,3,4-tri-*O*-benzyl-α-D-glucopyranoside (3e)

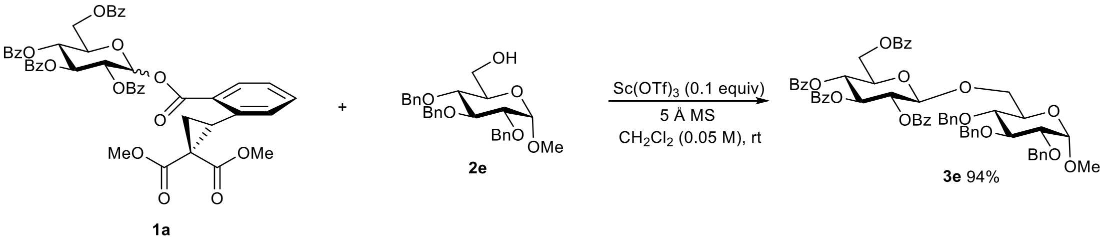

Supplementary Fig. 18 | Synthesis of 3e.

Following the procedure for **3a**, **2e** (23.2 mg, 50 µmol, 1.0 equiv) was transformed into **3e** (49.0 mg, 47 µmol, 94%) as a colorless syrup after purification by silica gel column chromatography (toluene:EtOAc = 20:1). ^{1}H NMR (400 MHz, CDCl$_3$) δ 8.00 (d, J = 7.7 Hz, 2H), 7.90 (d, J = 7.8 Hz, 4H), 7.92 – 7.89 (m, 2H), 7.50 (dd, J = 13.1, 7.0 Hz, 2H), 7.43 – 7.21 (m, 23H), 7.06 (d, J = 6.5 Hz, 2H), 5.90 (t, J = 9.6 Hz, 1H), 5.68 (t, J = 9.7 Hz, 1H), 5.60 (t, J = 8.7 Hz, 1H), 4.90 (d, J = 10.9 Hz, 1H), 4.83 (d, J = 7.8 Hz, 1H), 4.74 (d, J = 12.0 Hz, 1H), 4.69 (d, J = 10.9 Hz, 1H), 4.66 – 4.58 (m, 2H), 4.58 –

4.48 (m, 3H), 4.30 (d, J = 11.1 Hz, 1H), 4.16 (d, J = 9.7 Hz, 1H), 4.11 (dt, J = 9.3, 4.4 Hz, 1H), 3.89 (t, J = 9.2 Hz, 1H), 3.74 (t, J = 9.2 Hz, 2H), 3.44 (dd, J = 9.7, 3.5 Hz, 1H), 3.39 (t, J = 9.2 Hz, 1H), 3.22 (s, 3H). The data are identical with the literature.[8]

Methyl 6-*O*-(2,3,4,6-tetra-*O*-benzoyl-β-D-glucopyranosyl)-2,3,4-tri-*O*-benzoyl-α-D-glucopyranoside (3f)

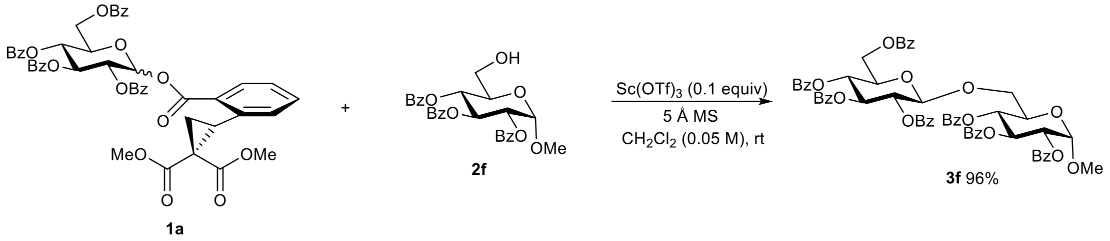

Supplementary Fig. 19 | Synthesis of 3f.

Following the procedure for **3a**, **2f** (25.3 mg, 50 μmol, 1.0 equiv) was transformed into **3f** (52.0 mg, 48 μmol, 96%) as a colorless syrup after purification by silica gel column chromatography (toluene:EtOAc = 20:1). ^{1}H NMR (400 MHz, $CDCl_3$) δ 8.03 – 7.78 (m, 14H), 7.59 – 7.45 (m, 5H), 7.45 – 7.31 (m, 12H), 7.30 – 7.24 (m, 5H), 6.08 (t, J = 9.8 Hz, 1H), 5.94 (t, J = 9.6 Hz, 1H), 5.67 (t, J = 9.7 Hz, 1H), 5.58 (t, J = 8.8 Hz, 1H), 5.33 (t, J = 9.8 Hz, 1H), 5.11 (dd, J = 10.2, 3.6 Hz, 1H), 5.04 – 4.92 (m, 2H), 4.62 (dd, J = 12.4, 3.1 Hz, 1H), 4.46 (dd, J = 12.2, 5.0 Hz, 1H), 4.24 (dd, J = 10.3, 7.6 Hz, 1H), 4.20 – 4.08 (m, 2H), 3.80 (dd, J = 11.4, 7.7 Hz, 1H), 3.12 (s, 3H). The data are identical with the literature.[8]

Phenyl 6-*O*-(2,3,4,6-tetra-*O*-benzoyl-β-D-glucopyranosyl)-2,3,4-tri-*O*-benzoyl-1-thio-β-D-glucopyranoside (3g)

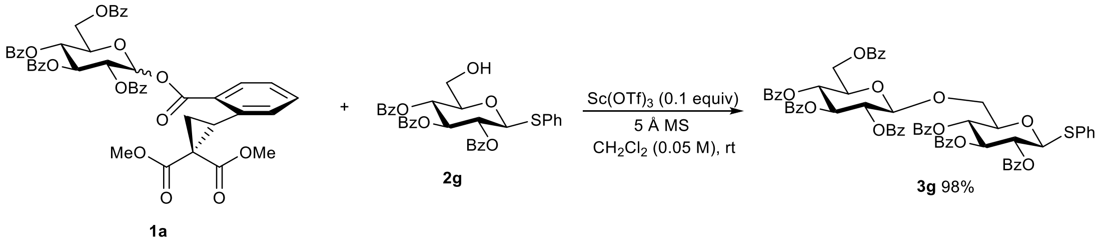

Supplementary Fig. 20 | Synthesis of 3g.

Following the procedure for **3a**, **2g** (29.2 mg, 50 μmol, 1.0 equiv) was transformed into **3g** (57.1 mg, 49 μmol, 98%) as a colorless syrup after purification by silica gel column chromatography (toluene:EtOAc = 20:1). ^{1}H NMR (400 MHz, $CDCl_3$) δ 8.06 (d, J = 7.0 Hz, 2H), 7.98 – 7.90 (m, 6H), 7.88 – 7.81 (m, 4H), 7.76 (d, J = 8.2 Hz, 2H), 7.60 – 7.32 (m, 23H), 7.31 – 7.26 (m, 4H), 5.89 – 5.79 (m, 2H), 5.62 (t, J = 9.7 Hz, 1H), 5.51 (dd, J = 9.8, 7.8 Hz, 1H), 5.37 (t, J = 9.7 Hz, 1H), 5.28 (t, J = 9.7 Hz, 1H), 4.98 (d, J = 7.8 Hz, 1H), 4.93 (d, J = 10.0 Hz, 1H), 4.62 (dd, J = 12.1, 3.1 Hz, 1H), 4.42 (dd, J = 12.2, 5.2 Hz, 1H), 4.10 – 3.93 (m, 4H). The data are identical with the literature.[18]

6-*O*-(2,3,4,6-Tetra-*O*-benzoyl-β-D-glucopyranosyl)-1,2:3,4-di-*O*-isopropylidene-α-D-galactopyranose (3h)

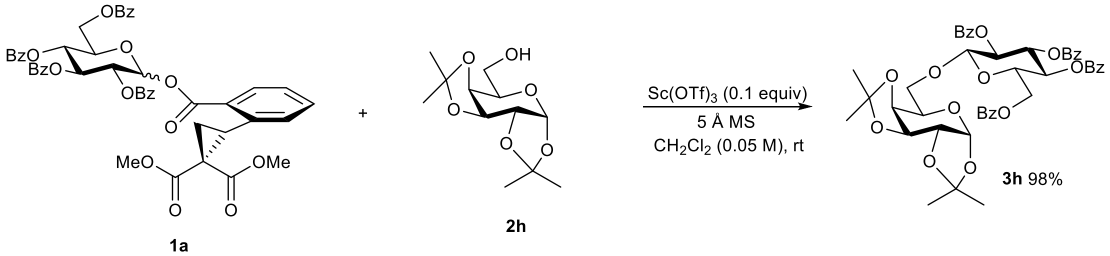

Supplementary Fig. 21 | Synthesis of 3h.

Following the procedure for **3a**, **2h** (16.0 mg, 60 μmol, 1.0 equiv) was transformed into **3h** (49.3 mg, 58.8 μmol, 98%) as a colorless syrup after purification by silica gel column chromatography (toluene:EtOAc = 15:1). ^{1}H NMR (400 MHz, $CDCl_3$) δ 8.03 (d, J = 7.0 Hz, 2H), 7.97 (d, J = 7.0 Hz, 2H), 7.90 (d, J = 7.2 Hz, 2H), 7.83 (d, J = 7.0 Hz, 2H), 7.55 – 7.45 (m, 3H), 7.45 – 7.27 (m, 9H), 5.90 (t, J = 9.6 Hz, 1H), 5.68 (t, J = 9.7 Hz, 1H), 5.54 (dd, J = 9.7, 7.8 Hz, 1H), 5.42 (d, J = 5.0 Hz, 1H), 5.04 (d, J = 7.8 Hz, 1H), 4.64 (dd, J = 12.1, 3.2 Hz, 1H), 4.49 (dd, J = 12.1, 5.3 Hz, 1H), 4.43 (dd, J = 7.9, 2.4 Hz, 1H), 4.24 – 4.14 (m, 2H), 4.10 (dd, J = 7.9, 1.7 Hz, 1H), 4.02 (dd, J = 10.5, 3.7 Hz, 1H), 3.93 – 3.81 (m, 2H), 1.37 (s, 3H), 1.25 – 1.18 (m, 9H). The data are identical with the literature.[8]

Methyl 5-*O*-(2,3,4,6-tetra-*O*-benzoyl-β-D-glucopyranosyl)-2,3-*O*-isopropylidene-β-D-ribofuranoside (3i)

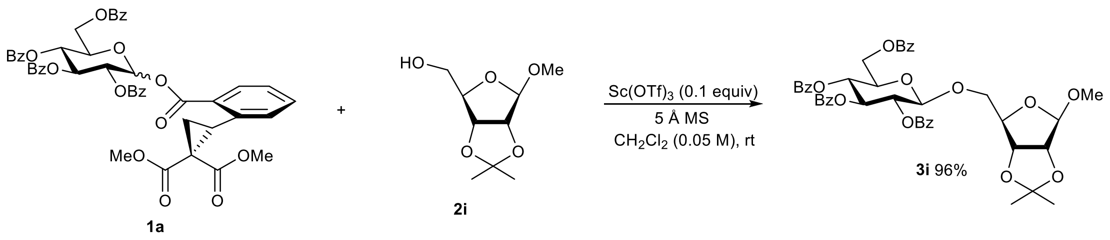

Supplementary Fig. 22 | Synthesis of 3i.

Following the procedure for **3a**, **2i** (10.2 mg, 50 μmol, 1.0 equiv) was transformed into **3i** (37.4 mg, 47 μmol, 96%) as a colorless syrup after purification by silica gel column chromatography (toluene:EtOAc = 15:1). ^{1}H NMR (400 MHz, $CDCl_3$) δ 8.03 (d, J = 7.8 Hz, 2H), 7.96 (d, J = 7.7 Hz, 2H), 7.90 (d, J = 7.8 Hz, 2H), 7.83 (d, J = 7.8 Hz, 2H), 7.57 – 7.47 (m, 3H), 7.44 – 7.26 (m, 9H), 5.90 (t, J = 9.6 Hz, 1H), 5.68 (t, J = 9.7 Hz, 1H), 5.55 (dd, J = 9.6, 7.9 Hz, 1H), 4.93 (d, J = 7.9 Hz, 1H), 4.87 (s, 1H), 4.70 – 4.58 (m, 2H), 4.55 – 4.46 (m, 2H), 4.27 (t, J = 7.2 Hz, 1H), 4.20 – 4.13 (m, 1H), 3.85 (t, J = 9.3 Hz, 1H), 3.71 – 3.65 (m, 1H), 3.18 (s, 3H), 1.37 (s, 3H), 1.16 (s, 3H). The data are identical with the literature.[19]

Methyl 3-*O*-(2,3,4,6-tetra-*O*-benzoyl-β-D-glucopyranosyl)-2-*O*-benzyl-4,6-*O*-benzylidene-α-D-glucopyranoside (3j)

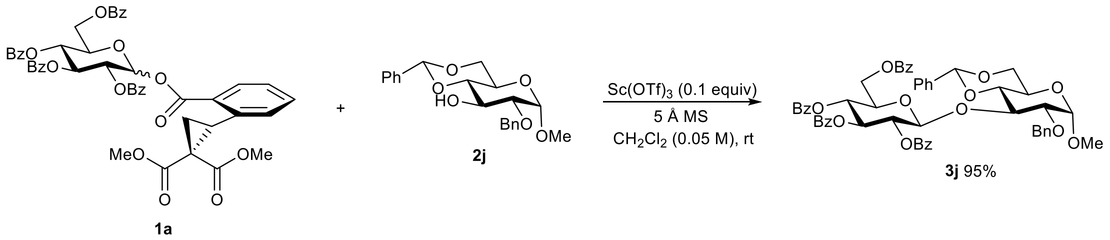

Supplementary Fig. 23 | Synthesis of 3j.

Following the procedure for **3a**, **2j** (18.6 mg, 50 μmol, 1.0 equiv) was transformed into **3j** (45.2 mg, 47.5 μmol, 95%) as a colorless syrup after purification by silica gel column chromatography (toluene:EtOAc = 20:1). ^{1}H NMR (400 MHz, $CDCl_3$) δ 8.00 – 7.92 (m, 4H), 7.86 (d, J = 7.0 Hz, 2H), 7.80 (d, J = 8.4 Hz, 2H), 7.53 – 7.44 (m, 5H), 7.43 – 7.38 (m, 1H), 7.37 – 7.27 (m, 8H), 7.26 – 7.20 (m, 6H), 7.11 (dd, J = 7.2, 2.4 Hz, 2H), 5.88 (t, J = 9.6 Hz, 1H), 5.74 – 5.63 (m, 2H), 5.56 (s, 1H), 5.25 (d, J = 7.9 Hz, 1H), 4.59 (d, J = 12.5 Hz, 1H), 4.50 (dd, J = 12.1, 3.5 Hz, 1H), 3.96 (ddd, J = 9.8, 4.8, 3.5

Hz, 1H), 3.77 (td, J = 9.8, 4.4 Hz, 1H), 3.69 (t, J = 10.1 Hz, 1H), 3.61 (t, J = 9.3 Hz, 1H), 3.43 (dd, J = 9.2, 3.8 Hz, 1H), 3.27 (s, 3H). The data are identical with the literature.[20]

Methyl 2-*O*-(2,3,4,6-tetra-*O*-benzoyl-β-D-glucopyranosyl)-3,4,6-tri-*O*-benzyl-α-D-mannopyranoside (3k)

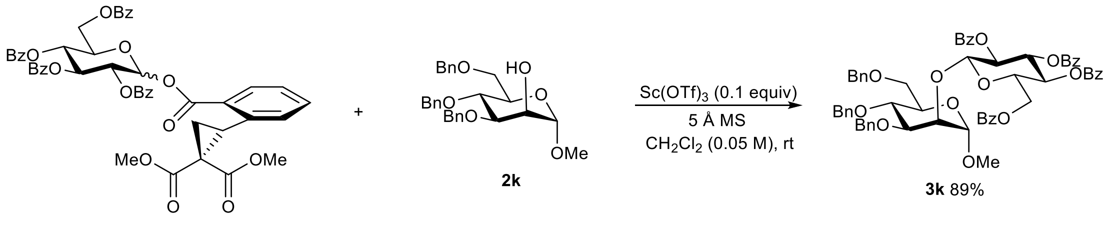

Supplementary Fig. 24 | Synthesis of 3k.

Following the procedure for **3a**, **2k** (23 mg, 50 μmol, 1.0 equiv) was transformed into **3k** (46.6 mg, 44.5 μmol, 89%) as a colorless syrup after purification by silica gel column chromatography (toluene:EtOAc = 20:1). ^{1}H NMR (400 MHz, $CDCl_3$) δ 7.98 (d, J = 8.4 Hz, 2H), 7.96 – 7.88 (m, 4H), 7.85 (d, J = 8.7 Hz, 2H), 7.54 – 7.47 (m, 4H), 7.37 – 7.18 (m, 22H), 7.16 – 7.10 (m, 2H), 5.91 (t, J = 9.7 Hz, 1H), 5.75 – 5.61 (m, 2H), 5.03 (d, J = 7.9 Hz, 1H), 4.82 – 4.72 (m, 2H), 4.70 – 4.63 (m, 2H), 4.56 – 4.47 (m, 2H), 4.39 (d, J = 11.0 Hz, 1H), 4.30 – 4.13 (m, 4H), 3.87 (q, J = 3.5 Hz, 1H), 3.68 – 3.60 (m, 2H), 3.55 (d, J = 10.8 Hz, 1H), 3.30 (dd, J = 10.4, 4.2 Hz, 1H), 3.22 (s, 3H). The data are identical with the literature.[21]

Methyl 4-*O*-(2,3,4,6-tetra-*O*-benzoyl-β-D-glucopyranosyl)-2,3-di-*O*-benzyl-6-*O*-methyl-α-D-glucopyranosyl urinate (3l)

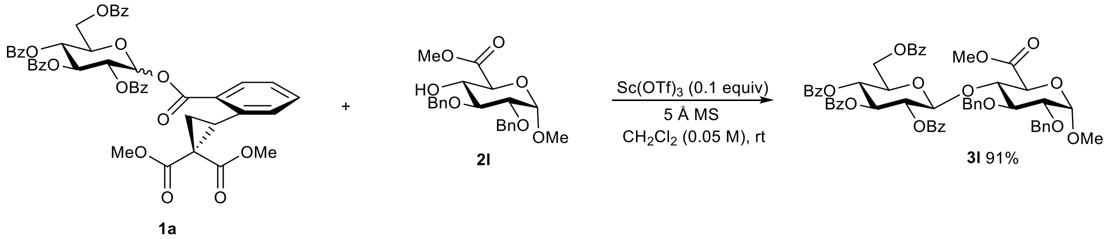

Supplementary Fig. 25 | Synthesis of 3l.

Following the procedure for **3a**, **2l** (20.1 mg, 50 μmol, 1.0 equiv) was transformed into **3l** (44.6 mg, 45.5 μmol, 91%) as a colorless syrup after purification by silica gel column chromatography (toluene:EtOAc = 20:1). ^{1}H NMR (400 MHz, $CDCl_3$) δ 7.98 – 7.89 (m, 4H), 7.84 (d, J = 7.0 Hz, 2H), 7.79 (d, J = 7.2 Hz, 2H), 7.54 – 7.43 (m, 3H), 7.41 – 7.16 (m, 19H), 5.83 (t, J = 9.7 Hz, 1H), 5.62 (t, J = 9.7 Hz, 1H), 5.45 (dd, J = 9.8, 7.9 Hz, 1H), 5.16 – 5.03 (m, 2H), 4.86 (d, J = 11.4 Hz, 1H), 4.72 (d, J = 12.2 Hz, 1H), 4.55 (d, J = 12.2 Hz, 1H), 4.51 (d, J = 3.5 Hz, 1H), 4.39 (dd, J = 12.1, 3.4 Hz, 1H), 4.27 (dd, J = 12.2, 4.7 Hz, 1H), 4.10 (dd, J = 9.8, 8.6 Hz, 1H), 4.04 – 3.92 (m, 3H), 3.47 (dd, J = 9.5, 3.5 Hz, 1H), 3.44 (s, 3H), 3.33 (s, 3H). The data are identical with the literature.[8]

Phthalimidyl 2,3,4,6-tetra-*O*-benzoyl-β-D-glucopyranoside (3m)

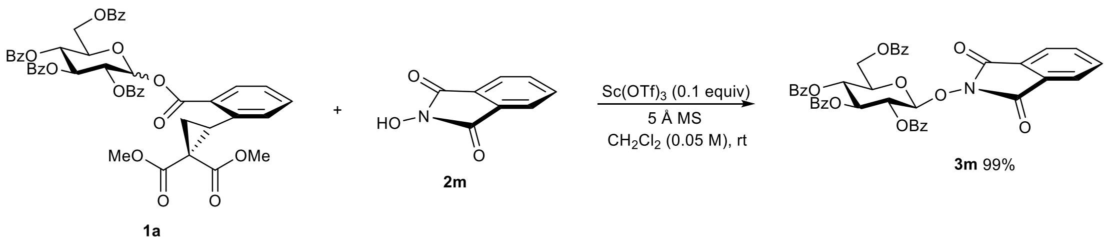

Supplementary Fig. 26 | Synthesis of 3m.

Following the procedure for **3a**, **2m** (9.8 mg, 60 μmol, 1.2 equiv) was transformed into **3m** (36.9 mg, 49.5 μmol, 99%) as a white foam after purification by silica gel column chromatography (hexane:EtOAc = 2:1 to toluene:EtOAc = 10:1). ^{1}H NMR (400 MHz, $CDCl_3$) δ 8.09 (d, J = 7.0 Hz, 2H), 7.94 – 7.84 (m, 6H), 7.79 (dd, J = 5.5, 3.1 Hz, 2H), 7.72 (dd, J = 5.5, 3.1 Hz, 2H), 7.58 – 7.39 (m, 6H), 7.37 – 7.22 (m, 6H), 5.98 (t, J = 9.2 Hz, 1H), 5.90 – 5.77 (m, 2H), 5.54 (d, J = 7.7 Hz, 1H), 4.67 – 4.57 (m, 2H), 4.24 (dt, J = 9.8, 4.9 Hz, 1H). The data are identical with the literature.[22]

***ortho*-Iodobenzoyl 2,3,4,6-tetra-*O*-benzoyl-β-D-glucopyranoside (3n)**

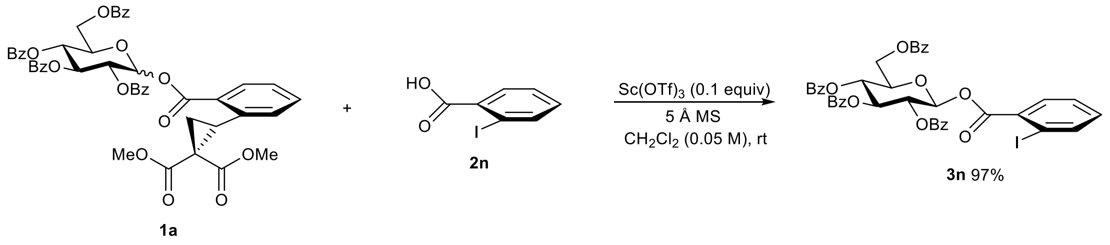

Supplementary Fig. 27 | Synthesis of 3n.

Following the procedure for **3a**, **2n** (18.6 mg, 75 μmol, 1.5 equiv) was transformed into **3n** (40.1 mg, 48.5 μmol, 97%) as a colorless syrup after purification by silica gel column chromatography (hexane:EtOAc = 5:1). $[\alpha]_D^{23}$ = +14.3 (*c* = 1.0, $CHCl_3$); ^1H NMR (400 MHz, $CDCl_3$) *δ* 8.06 (d, *J* = 7.0 Hz, 2H), 7.98 – 7.89 (m, 6H), 7.86 (d, *J* = 7.0 Hz, 2H), 7.57 – 7.27 (m, 14H), 7.13 (td, *J* = 7.7, 1.7 Hz, 1H), 6.33 (d, *J* = 8.1 Hz, 1H, H-1), 6.04 (t, *J* = 9.5 Hz, 1H), 5.89 – 5.77 (m, 2H), 4.68 (dd, *J* = 12.3, 2.9 Hz, 1H, H-5), 4.53 (dd, *J* = 12.3, 5.0 Hz, 1H, H-5′), 4.40 (ddd, *J* = 9.9, 5.0, 2.9 Hz, 1H, H-4); ^{13}C NMR (100 MHz, $CDCl_3$) *δ* 166.2, 165.8, 165.3, 163.8, 142.0, 133.7, 133.5, 133.2, 132.1, 130.0, 130.0, 129.9, 129.7, 128.9, 128.8, 128.6, 128.5, 128.2, 95.2, 93.1, 73.4, 73.0, 71.0, 69.2, 62.8; HRMS (ESI) *m/z* Calcd for $C_{41}H_{31}O_{11}NaI$ $[M + Na]^+$ 849.0809, found 849.0809.

Phenyl 2,3,4,6-tetra-*O*-benzoyl-β-D-glucopyranoside (3o)

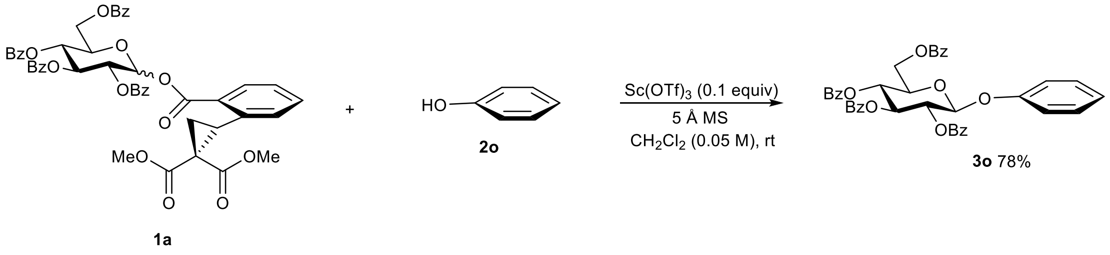

Supplementary Fig. 28 | Synthesis of 3o.

Following the procedure for **3a**, **2o** (11.3 mg, 0.12 mmol, 1.5 equiv) was transformed into **3o** (43.1 mg, 64 μmol, 78%) as a colorless syrup after purification by silica gel column chromatography (hexane:EtOAc = 4:1). ^1H NMR (400 MHz, $CDCl_3$) *δ* 8.04 (d, *J* = 7.0 Hz, 2H), 8.00 – 7.91 (m, 4H), 7.87 (d, *J* = 8.3 Hz, 2H), 7.61 – 7.55 (m, 1H), 7.55 – 7.49 (m, 2H), 7.48 – 7.28 (m, 9H), 7.21 – 7.14 (m, 2H), 7.06 – 6.98 (m, 3H), 6.00 (t, *J* = 9.5 Hz, 1H), 5.82 (dd, *J* = 9.6, 7.8 Hz, 1H), 5.72 (t, *J* = 9.6 Hz, 1H), 5.41 (d, *J* = 7.8 Hz, 1H), 4.69 (dd, *J* = 12.1, 3.0 Hz, 1H), 4.54 (dd, *J* = 12.0, 6.6 Hz, 1H), 4.34 (ddd, *J* = 9.8, 6.6, 3.0 Hz, 1H). The data are identical with the literature.[23]

***N*-(2,3,4,6-Tetra-*O*-benzoyl-β-D-glucopyranosyl)-*para*-methylbenzenesulfonamide (3p)**

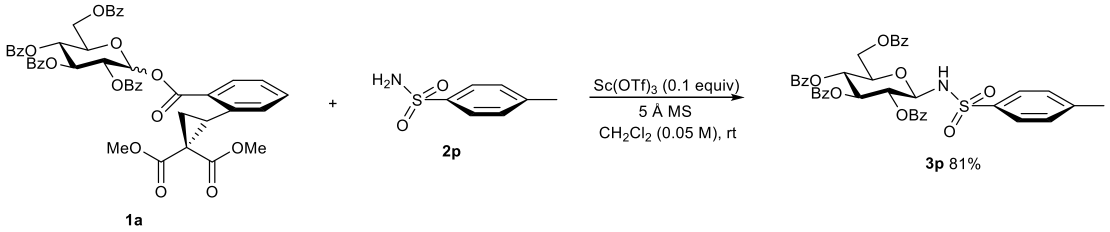

Supplementary Fig. 29 | Synthesis of 3p.

Following the procedure for **3a**, **2p** (10.3 mg, 60 μmol, 1.5 equiv) was transformed into **3p** (30.4 mg, 40.5 μmol, 81%) as a colorless syrup after purification by silica gel column chromatography (toluene:EtOAc = 12:1). ^{1}H NMR (400 MHz, $CDCl_3$) δ 8.01 (d, J = 7.0 Hz, 2H), 7.88 (d, J = 7.0 Hz, 2H), 7.84 (d, J = 8.3 Hz, 2H), 7.80 (d, J = 7.0 Hz, 2H), 7.66 (d, J = 8.4 Hz, 2H), 7.58 – 7.47 (m, 4H), 7.44 – 7.32 (m, 8H), 6.94 (d, J = 8.2 Hz, 2H), 5.97 (t, J = 9.6 Hz, 1H), 5.86 (d, J = 9.3 Hz, 1H), 5.61 (t, J = 9.8 Hz, 1H), 5.33 (t, J = 9.5 Hz, 1H), 5.14 (t, J = 9.3 Hz, 1H), 4.45 (dd, J = 12.2, 3.0 Hz, 1H), 4.36 (dd, J = 12.2, 4.9 Hz, 1H), 4.18 (ddd, J = 10.0, 4.9, 3.0 Hz, 1H), 2.17 (s, 3H). The data are identical with the literature.[24]

***para*-Tolyl 2,3,4,6-*O*-benzoyl-1-thio-β-D-glucopyranoside (3q)**

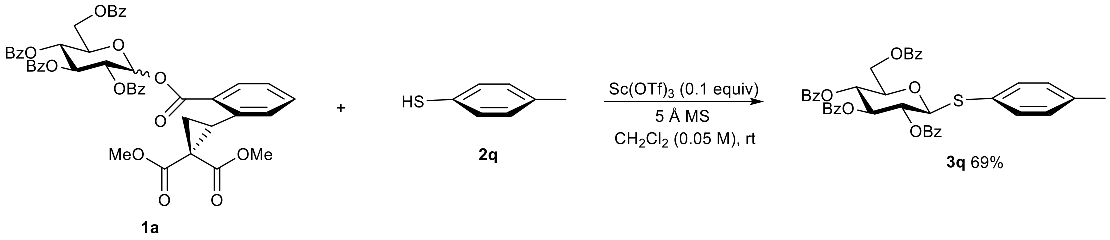

Supplementary Fig. 30 | Synthesis of 3q.

Following the procedure for **3a**, **2q** (7.5 mg, 75 μmol, 1.5 equiv) was transformed into **3q** (24.1 mg, 34.5 μmol, 69%) as a colorless syrup after purification by silica gel column chromatography (toluene:EtOAc = 20:1). ^{1}H NMR (400 MHz, $CDCl_3$) δ 8.05 (d, J = 6.9 Hz, 2H), 7.98 (d, J = 7.0 Hz, 2H), 7.90 (d, J = 7.0 Hz, 2H), 7.80 (d, J = 7.0 Hz, 2H), 7.62 – 7.32 (m, 12H), 7.29 – 7.24 (m, 3H), 6.94 (d, J = 7.9 Hz, 2H), 5.90 (t, J = 9.5 Hz, 1H), 5.60 (t, J = 9.8 Hz, 1H), 5.46 (t, J = 9.7 Hz, 1H), 4.99 (d, J = 10.0 Hz,

1H), 4.69 (dd, J = 12.2, 2.8 Hz, 1H), 4.48 (dd, J = 12.2, 5.7 Hz, 1H), 4.18 (ddd, J = 10.0, 5.7, 2.8 Hz, 1H), 2.28 (s, 3H). The data are identical with the literature.[8]

Octyl 2,3,4,6-tetra-*O*-benzoyl-1-thio-β-D-glucopyranoside (3r)

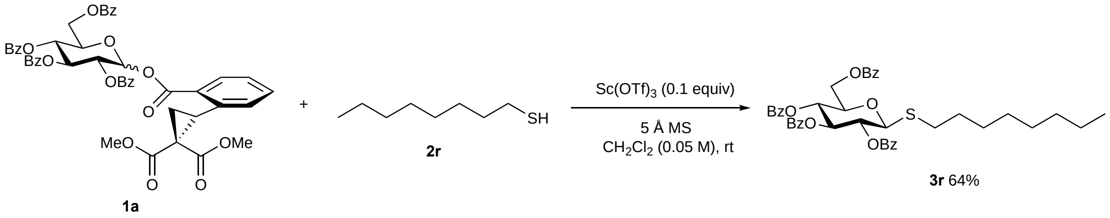

Supplementary Fig. 31 | Synthesis of 3r.

Following the procedure for **3a**, **2r** (11.9 μL, 75 μmol, 1.5 equiv) was transformed into **3q** (23.2 mg, 32.0 μmol, 64%) as a colorless syrup after purification by silica gel column chromatography (hexane:EtOAc = 9:1). $[\alpha]_D^{23}$ = +13.0 (c = 1.0, $CHCl_3$); ^{1}H NMR (400 MHz, $CDCl_3$) δ 8.02 (d, J = 6.9 Hz, 2H), 7.95 (d, J = 6.9 Hz, 2H), 7.90 (d, J = 6.9 Hz, 2H), 7.82 (d, J = 7.0 Hz, 2H), 7.56 – 7.46 (m, 3H), 7.44 – 7.32 (m, 7H), 7.29 – 7.25 (m, 2H), 5.93 (t, J = 9.5 Hz, 1H), 5.67 (t, J = 9.8 Hz, 1H), 5.56 (t, J = 9.7 Hz, 1H), 4.85 (d, J = 10.0 Hz, 1H, H-1), 4.63 (dd, J = 12.2, 3.1 Hz, 1H, H-6), 4.50 (dd, J = 12.2, 5.4 Hz, 1H, H-6′), 4.18 (ddd, J = 10.0, 5.5, 3.1 Hz, 1H, H-5), 2.81 – 2.65 (m, 2H, SCH_2-), 1.61 – 1.54 (m, 2H), 1.30 – 1.17 (m, 10H), 0.86 (t, J = 7.0 Hz, 3H); ^{13}C NMR (100 MHz, $CDCl_3$) δ 166.2, 166.0, 165.4, 165.3, 133.6, 133.41, 133.36, 133.3, 130.01, 129.97, 129.9, 129.8, 129.4, 129.0, 128.9, 128.6, 128.5, 128.4, 84.2, 76.5, 74.3, 70.8, 69.8, 63.5, 31.9, 30.3, 29.8, 29.3, 29.2, 28.9, 22.8, 14.2; HRMS (ESI) *m/z* Calcd for $C_{42}H_{44}O_9SNa$ $[M + Na]^+$ 747.2604, found 747.2625.

Section 4. Strain-release glycosylation with 1b-h as the donors.

1-Adamantyl 2′,3′,4′,6′-tetra-*O*-benzoyl-β-D-galactopyranoside (3ba)

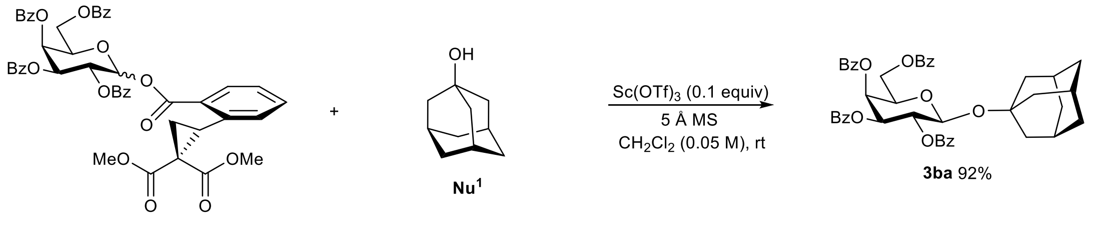

Supplementary Fig. 32 | Synthesis of 3ba.

Following the procedure for **3a**, **Nu¹** (7.6 mg, 50 μmol, 1.0 equiv) was transformed into **3ba** (33.7 mg, 46 μmol, 92%) as a white foam after purification by silica gel column chromatography (hexane:EtOAc = 7:1). ^{1}H NMR (400 MHz, $CDCl_3$) *δ* 8.12 (d, *J* = 7.7 Hz, 2H), 8.04 (d, *J* = 7.8 Hz, 2H), 7.96 (d, *J* = 7.8 Hz, 2H), 7.79 (d, *J* = 7.8 Hz, 2H), 7.65 – 7.34 (m, 10H), 7.26 – 7.20 (m, 2H), 5.97 (d, *J* = 3.5 Hz, 1H), 5.78 (dd, *J* = 10.3, 7.9 Hz, 1H), 5.61 (dd, *J* = 10.3, 3.5 Hz, 1H), 5.10 (d, *J* = 7.9 Hz, 1H), 4.61 (dd, *J* = 11.4, 7.7 Hz, 1H), 4.47 (dd, *J* = 11.5, 5.4 Hz, 1H), 4.33 (t, *J* = 6.6 Hz, 1H), 2.04 (s, 3H), 1.85 (d, *J* = 11.9 Hz, 3H), 1.70 (d, *J* = 13.1 Hz, 4H), 1.54 (q, *J* = 13.0, 12.6 Hz, 6H). The data are identical with the literature.[25]

Phenyl 6-*O*-(2,3,4,6-tetra-*O*-benzoyl-β-D-galactopyranosyl)-2,3,4-tri-*O*-benzoyl-1-thio-β-D-glucopyranoside (3bb)

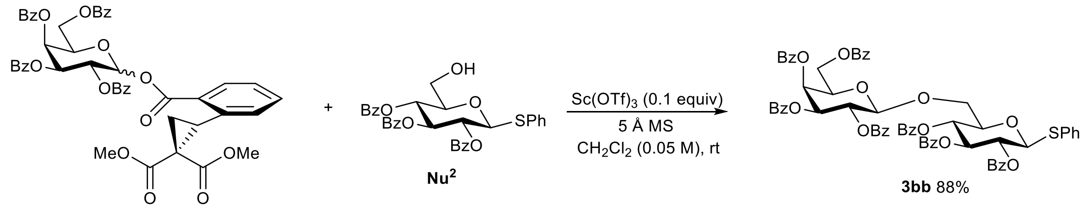

Supplementary Fig. 33 | Synthesis of 3bb.

Following the procedure for **3a**, **Nu²** (29.2 mg, 50 μmol, 1.0 equiv) was transformed into **3bb** (51.1 mg, 44 μmol, 88%) as a colorless syrup after purification by silica gel column chromatography (toluene:EtOAc = 20:1). ^{1}H NMR (400 MHz, $CDCl_3$) *δ* 8.10 – 8.02 (m, 4H), 8.00 – 7.91 (m, 4H), 7.86 – 7.78 (m, 4H), 7.75 (d, *J* = 7.8 Hz, 2H), 7.65

– 7.31 (m, 23H), 7.27 – 7.24 (m, 3H), 5.98 (d, *J* = 3.4 Hz, 1H), 5.85 – 5.76 (m, 2H), 5.56 (dd, *J* = 10.4, 3.3 Hz, 1H), 5.39 – 5.24 (m, 2H), 4.98 (d, *J* = 8.1 Hz, 1H), 4.92 (d, *J* = 10.0 Hz, 1H), 4.59 (dd, *J* = 11.4, 6.5 Hz, 1H), 4.40 (dd, *J* = 11.4, 6.5 Hz, 1H), 4.24 (t, *J* = 6.6 Hz, 1H), 4.10 – 4.02 (m, 2H), 3.98 (dd, *J* = 12.2, 7.7 Hz, 1H). The data are identical with the literature.[26]

***ortho*-Iodobenzoyl 2,3,4,6-tetra-*O*-benzoyl-β-D-galactopyranoside (3bc)**

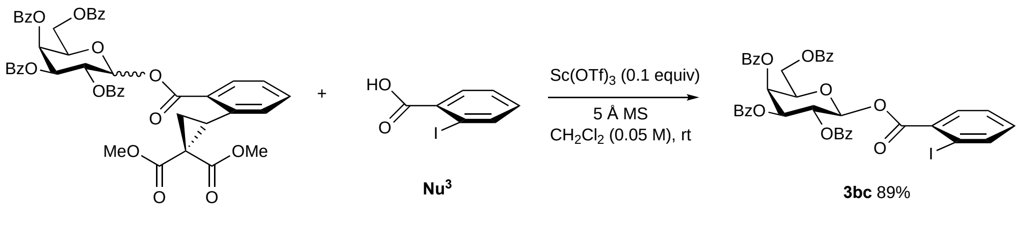

Supplementary Fig. 34 | Synthesis of 3bc.

Following the procedure for **3a**, **Nu^3** (18.6 mg, 75 μmol, 1.5 equiv) was transformed into **3bc** (36.6 mg, 44.5 μmol, 89%) as a colorless syrup after purification by silica gel column chromatography ($CHCl_3$:EtOAc = 100:1 to 30:1). $[\alpha]_D^{23}$ = +109.2 (*c* = 1.0, $CHCl_3$); ^{1}H NMR (400 MHz, $CDCl_3$) δ 8.11 (d, *J* = 7.0 Hz, 2H), 8.03 (d, *J* = 7.0 Hz, 2H), 8.00 – 7.91 (m, 4H), 7.81 (d, *J* = 7.2 Hz, 2H), 7.63 (t, *J* = 7.5 Hz, 1H), 7.55 (t, *J* = 7.4 Hz, 1H), 7.52 – 7.32 (m, 9H), 7.30 – 7.23 (m, 3H), 7.15 (td, *J* = 7.7, 1.7 Hz, 1H), 6.33 (d, *J* = 8.3 Hz, 1H, H-1), 6.14 – 6.03 (m, 2H, H-2&H-4), 5.77 (dd, *J* = 10.3, 3.4 Hz, 1H, H-3), 4.68 (dd, *J* = 11.0, 6.5 Hz, 1H, H-6), 4.58 (td, *J* = 6.4, 1.2 Hz, 1H, H-5), 4.47 (dd, *J* = 11.1, 6.3 Hz, 1H, H-6′); ^{13}C NMR (100 MHz, $CDCl_3$) δ 166.1, 165.6, 165.5, 164.0, 141.9, 133.8, 133.7, 133.5, 133.4, 132.5, 132.0, 130.2, 130.01, 129.97, 129.9, 129.5, 129.1, 129.0, 128.80, 128.78, 128.62, 128.58, 128.5, 128.2, 95.1, 93.4, 72.7, 71.8, 68.9, 68.0, 61.9; HRMS (ESI) *m/z* Calcd for $C_{41}H_{31}O_{11}NaI$ [M + Na]$^+$ 849.0809, found 849.0809.

1-Adamantyl 2′,3′,4′,6′-tetra-*O*-benzoyl-α-D-mannopyranoside (3ca)

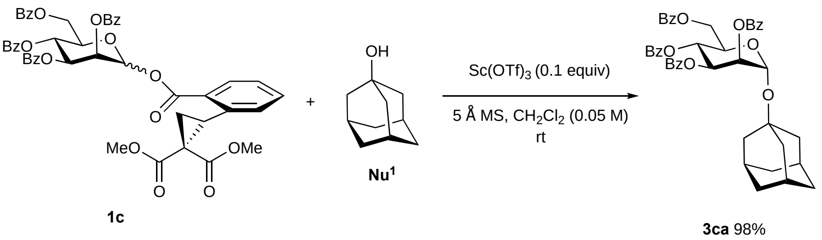

Supplementary Fig. 35 | Synthesis of 3ca.

Following the procedure for **3a**, **Nu**$^{\mathbf{1}}$ (7.6 mg, 50 μmol, 1.0 equiv) was transformed into **3ca** (35.7 mg, 49 μmol, 98%) as a white foam after purification by silica gel column chromatography (hexane:EtOAc = 7:1). ^{1}H NMR (400 MHz, $CDCl_3$) δ 8.14 – 8.06 (m, 4H), 7.99 (d, J = 7.0 Hz, 2H), 7.86 (d, J = 7.0 Hz, 2H), 7.62 – 7.48 (m, 3H), 7.47 – 7.35 (m, 7H), 7.30 – 7.25 (m, 3H), 6.10 – 5.94 (m, 2H), 5.57 – 5.50 (m, 2H), 4.70 – 4.61 (m, 2H), 4.49 (dd, J = 12.5, 6.0 Hz, 1H), 2.17 – 2.10 (m, 3H), 1.92 (d, J = 4.0 Hz, 6H), 1.61 (q, J = 12.5, 12.0 Hz, 6H). The data are identical with the literature.[27]

Phenyl 6-*O*-(2,3,4,6-tetra-*O*-benzoyl-α-D-mannopyranosyl)-2,3,4-tri-*O*-benzoyl-1-thio-β-D-glucopyranoside (3cb)

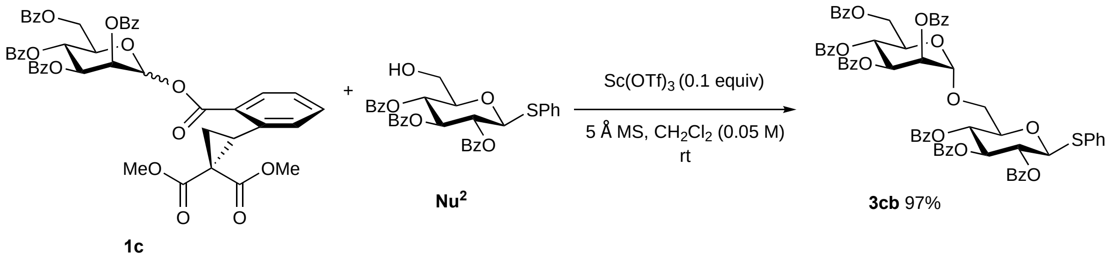

Supplementary Fig. 36 | Synthesis of 3cb.

Following the procedure for **3a**, **Nu**$^{\mathbf{2}}$ (59.2 mg, 50 μmol, 1.0 equiv) was transformed into **3cb** (56.2 mg, 48.5 μmol, 97%) as a colorless syrup after purification by silica gel column chromatography (toluene:EtOAc = 25:1). $[\alpha]_D^{23}$ = +15.0 (c = 1.0, $CHCl_3$); ^{1}H NMR (400 MHz, $CDCl_3$) δ 8.13 (d, J = 7.0 Hz, 2H), 8.04 (d, J = 6.9 Hz, 2H), 7.99 (d, J = 7.0 Hz, 2H), 7.95 (d, J = 7.0 Hz, 2H), 7.90 (d, J = 6.9 Hz, 2H), 7.85 (d, J = 7.0 Hz, 2H), 7.81 (d, J = 7.0 Hz, 2H), 7.63 – 7.24 (m, 28H), 6.13 (t, J = 10.1 Hz, 1H), 6.02 – 5.93 (m, 2H), 5.79 (dd, J = 3.4, 1.7 Hz, 1H, H-2-*manno*), 5.58 – 5.49 (m, 2H), 5.19 – 5.12 (m, 2H, H-1-*manno*&H-1-*gluco*), 4.65 (dd, J = 12.1, 2.4 Hz, 1H, H-6-*manno*), 4.59 (ddd, J = 10.1, 4.5, 2.4 Hz, 1H, H-5-*manno*), 4.41 (dd, J = 12.1, 4.4 Hz, 1H, H-6′-

manno), 4.25 (ddd, *J* = 9.6, 7.1, 1.9 Hz, 1H, H-5-*gluco*), 4.16 (dd, *J* = 10.6, 7.1 Hz, 1H, H-6-*gluco*), 3.82 (dd, *J* = 10.7, 2.0 Hz, 1H, H-6′-*gluco*); ^{13}C NMR (100 MHz, $CDCl_3$) δ 166.1, 165.9, 165.6, 165.5, 165.44, 165.41, 165.2, 133.7, 133.6, 133.43, 133.36, 133.3, 133.2, 131.9, 130.1, 130.01, 129.97, 129.96, 129.93, 129.89, 129.44, 129.35, 129.34, 129.27, 129.0, 128.9, 128.7, 128.63, 128.61, 128.51, 128.46, 128.43, 128.41, 128.3, 97.7, 87.0, 74.3, 70.6, 70.31, 70.26, 69.5, 69.2, 67.2, 66.7, 62.7; HRMS (ESI) *m/z* Calcd for $C_{67}H_{54}O_{17}NaS$ $[M + Na]^+$ 1185.2979, found 1185.2968.

***ortho*-Iodobenzoyl 2,3,4,6-tetra-*O*-benzoyl-α-D-mannopyranoside (3cc)**

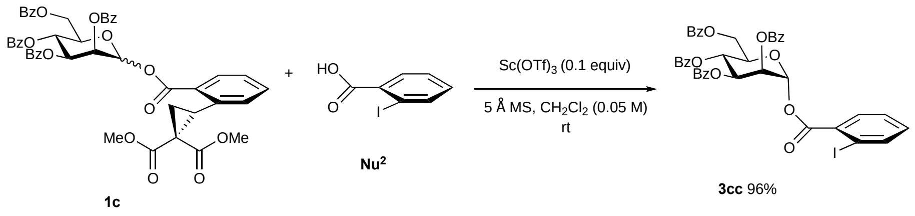

Supplementary Fig. 37 | Synthesis of 3cc.

Following the procedure for **3a**, **Nu³** (18.6 mg, 75 μmol, 1.5 equiv) was transformed into **3cc** (39.8 mg, 48 μmol, 96%) as a colorless syrup after purification by silica gel column chromatography (hexane:EtOAc = 5:1). $[\alpha]_D^{23}$ = –28.4 (*c* = 1.0, $CHCl_3$); ^{1}H NMR (400 MHz, $CDCl_3$) δ 8.14 – 8.06 (m, 5H), 7.99 – 7.93 (m, 3H), 7.85 (d, *J* = 7.0 Hz, 2H), 7.66 – 7.49 (m, 4H), 7.46 – 7.33 (m, 7H), 7.30 – 7.25 (m, 3H), 6.65 (d, *J* = 1.9 Hz, 1H, H-1), 6.26 (t, *J* = 10.1 Hz, 1H, H-4), 6.11 (dd, *J* = 10.2, 3.3 Hz, 1H, H-3), 5.95 (dd, *J* = 3.3, 2.0 Hz, 1H, H-2), 4.74 (dd, *J* = 12.2, 2.6 Hz, 1H, H-6), 4.67 (dt, *J* = 10.1, 3.2 Hz, 1H, H-5), 4.52 (dd, *J* = 12.2, 4.0 Hz, 1H, H-6′); ^{13}C NMR (100 MHz, $CDCl_3$) δ 166.2, 165.7, 165.4, 165.3, 164.1, 141.8, 134.2, 133.8, 133.7, 133.6, 133.5, 133.2, 131.8, 130.1, 129.94, 129.89, 129.1, 128.92, 128.89, 128.8, 128.6, 128.5, 128.4, 94.3, 92.2, 71.6, 70.0, 69.3, 66.3, 62.6; HRMS (ESI) *m/z* Calcd for $C_{41}H_{31}O_{11}NaI$ $[M + Na]^+$ 849.0809, found 849.0809.

1-Adamantyl 3′,4′,6′-tri-*O*-acetyl-2′-deoxy-2′-phthalimido-β-D-glucopyranoside (3da)

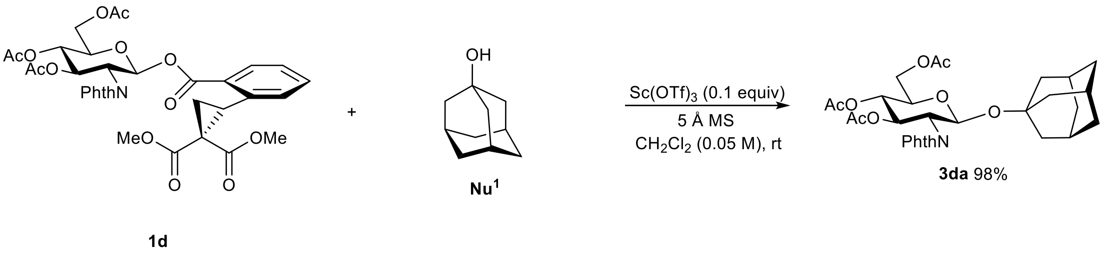

Supplementary Fig. 38 | Synthesis of 3da.

Following the procedure for **3a**, **Nu^1** (7.6 mg, 50 μmol, 1.0 equiv) was transformed into **3da** (27.9 mg, 49 μmol, 98%) as a white foam after purification by silica gel column chromatography (hexane:EtOAc = 3:1). ^{1}H NMR (400 MHz, $CDCl_3$) δ 7.85 (dd, *J* = 5.4, 3.1 Hz, 2H), 7.73 (dd, *J* = 5.5, 3.0 Hz, 2H), 5.84 (dd, *J* = 10.7, 9.0 Hz, 1H), 5.56 (d, *J* = 8.4 Hz, 1H), 5.10 (dd, *J* = 10.1, 9.0 Hz, 1H), 4.35 – 4.24 (m, 2H), 4.11 (dd, *J* = 12.0, 2.5 Hz, 1H), 3.87 (ddd, *J* = 10.2, 5.7, 2.5 Hz, 1H), 2.07 (s, 3H), 2.02 (d, *J* = 4.3 Hz, 6H), 1.84 (s, 3H), 1.72 – 1.67 (m, 3H), 1.56 – 1.45 (m, 9H). The data are identical with the literature.[28]

Phenyl 6-*O*-(3,4,6-tri-*O*-acetyl-2-deoxy-2-phthalimido-β-D-glucopyranosyl)-2,3,4-tri-*O*-benzoyl-1-thio-β-D-glucopyranoside (3db)

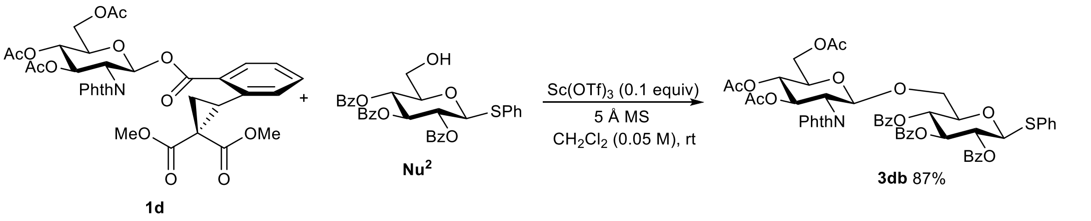

Supplementary Fig. 39 | Synthesis of 3db.

Following the procedure for **3a**, **Nu^2** (29.2 mg, 50 μmol, 1.0 equiv) was transformed into **3db** (46.7 mg, 43.5 μmol, 87%) as a colorless syrup after purification by silica gel column chromatography (toluene:EtOAc = 7:1 to 6:1). $[\alpha]_D^{23}$ = +27.2 (*c* = 1.0, $CHCl_3$); ^{1}H NMR (400 MHz, $CDCl_3$) δ 7.83 (d, *J* = 7.0 Hz, 2H), 7.73 (d, *J* = 9.8 Hz, 2H), 7.64 (d, *J* = 8.4 Hz, 2H), 7.53 (dd, *J* = 5.6, 2.9 Hz, 2H), 7.46 – 7.39 (m, 2H), 7.34 – 7.24 (m, 12H), 7.15 (t, *J* = 7.8 Hz, 2H), 5.75 – 5.63 (m, 2H), 5.45 (d, *J* = 8.5 Hz, 1H, H-1-*glucosamino*), 5.28 –5.14 (m, 2H), 5.07 (dd, *J* = 10.1, 9.1 Hz, 1H), 4.79 (d, *J* = 10.0 Hz, 1H, H-1-*gluco*), 4.29 (dd, *J* = 10.7, 8.5 Hz, 1H, H-2-*glucosamino*), 4.18 (dd, *J* = 12.3,

4.8 Hz, 1H, H-6-*glucosamino*), 4.02 (dd, J = 12.3, 2.3 Hz, 1H, H-6′-*glucosamino*), 3.98 – 3.89 (m, 2H, H-5-*glucosamino*&H-6-*gluco*), 3.77 – 3.66 (m, 2H, H-5-*gluco*&H-6′-*gluco*), 1.99 – 1.93 (m, 6H), 1.79 (s, 3H); ^{13}C NMR (100 MHz, $CDCl_3$) δ 170.8, 170.2, 169.6, 165.8, 165.3, 165.0, 134.1, 133.6, 133.4, 133.3, 132.6, 132.2, 129.90, 129.87, 129.8, 129.3, 129.1, 129.0, 128.8, 128.70, 128.66, 128.6, 128.5, 128.4, 128.3, 128.2, 123.7, 98.5, 85.9, 74.2, 72.0, 71.0, 70.4, 69.6, 69.3, 69.0, 62.0, 54.6, 20.8, 20.7, 20.5; HRMS (ESI) *m/z* Calcd for $C_{53}H_{47}O_{17}NNaS$ [M + Na]$^+$ 1024.2462, found 1024.2476.

ortho-Iodobenzoyl 3,4,6-tri-_O_-acetyl-2-deoxy-2-phthalimido-β-D-glucopyranoside (3dc)

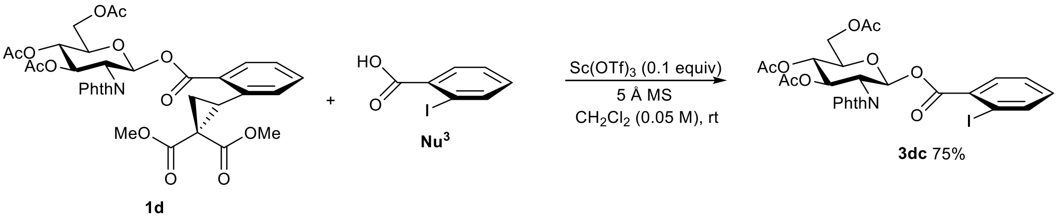

Supplementary Fig. 40 | Synthesis of 3dc.

Following the procedure for **3a**, **Nu³** (18.6 mg, 75 μmol, 1.5 equiv) was transformed into **3dc** (24.9 mg, 37.5 μmol, 75%) as a colorless syrup after purification by silica gel column chromatography (hexane:EtOAc = 2:1). $[\alpha]_D^{23}$ = +29.5 (c = 1.0, $CHCl_3$); ^{1}H NMR (400 MHz, $CDCl_3$) δ 7.91 (dd, J = 7.9, 1.2 Hz, 1H), 7.84 (dd, J = 5.5, 3.1 Hz, 2H), 7.79 (dd, J = 7.8, 1.7 Hz, 1H), 7.71 (dd, J = 5.5, 3.1 Hz, 2H), 7.35 (td, J = 7.6, 1.2 Hz, 1H), 7.11 (td, J = 7.7, 1.7 Hz, 1H), 6.74 (d, J = 8.9 Hz, 1H, H-1), 5.98 (dd, J = 10.6, 9.1 Hz, 1H), 5.27 (dd, J = 10.2, 9.1 Hz, 1H), 4.64 (dd, J = 10.6, 8.8 Hz, 1H, H-2), 4.41 (dd, J = 12.5, 4.4 Hz, 1H, H-6), 4.18 (dd, J = 12.4, 2.2 Hz, 1H, H-6′), 4.11 (ddd, J = 10.2, 4.4, 2.2 Hz, 1H, H-5), 2.11 (s, 3H), 2.05 (s, 3H), 1.88 (s, 3H); ^{13}C NMR (100 MHz, $CDCl_3$) δ 170.8, 170.2, 169.7, 167.6, 163.6, 141.9, 134.6, 133.6, 132.6, 131.9, 131.4, 128.2, 124.0, 94.8, 90.8, 72.9, 70.6, 68.5, 61.7, 53.7, 20.9, 20.8, 20.6; HRMS (ESI) *m/z* Calcd for $C_{27}H_{24}O_{11}NNaI$ [M + Na]$^+$ 688.0292, found 688.0281.

1-Adamantyl 2′,3′,4′-tri-_O_-benzoyl-β-D-xylopyranoside (3ea)

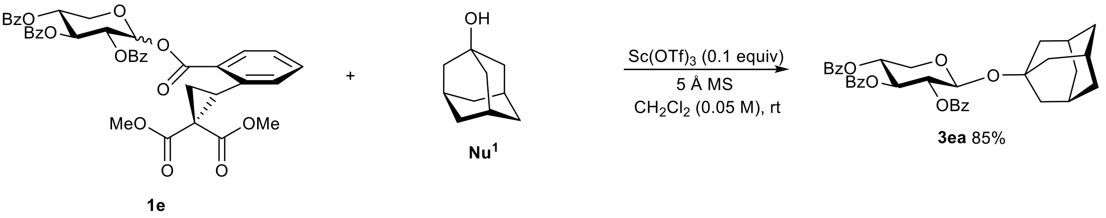

Supplementary Fig. 41 | Synthesis of 3ea.

Following the procedure for **3a**, **Nu1** (7.6 mg, 50 μmol, 1.0 equiv) was transformed into **3ea** (25.3 mg, 42.5 μmol, 85%) as a white foam after purification by silica gel column chromatography (hexane:EtOAc = 7:1). $[\alpha]_D^{23}$ = –9.6 (c = 1.0, $CHCl_3$); ^{1}H NMR (400 MHz, $CDCl_3$) δ 8.04 – 7.94 (m, 6H), 7.56 – 7.46 (m, 3H), 7.41 – 7.32 (m, 6H), 5.78 (t, J = 7.8 Hz, 1H, H-3′), 5.37 – 5.26 (m, 2H, H-2′&H-4′), 5.14 (d, J = 6.0 Hz, 1H, H-1′), 4.45 (dd, J = 12.0, 4.6 Hz, 1H, H-5′), 3.65 (dd, J = 12.0, 7.8 Hz, 1H, H-5″), 2.17 – 2.08 (m, 3H), 1.89 – 1.80 (m, 3H), 1.72 (dt, J = 11.5, 3.2 Hz, 3H), 1.57 (dd, J = 12.4, 8.8 Hz, 6H); ^{13}C NMR (100 MHz, $CDCl_3$) δ 165.7, 165.2, 133.44, 133.36, 133.2, 130.02, 130.00, 129.9, 129.7, 129.5, 129.4, 128.6, 128.5, 93.7, 75.6, 71.4, 71.2, 69.7, 61.5, 42.6, 36.3, 30.7.0; HRMS (ESI) m/z Calcd for $C_{36}H_{36}O_8Na$ $[M + Na]^+$ 619.2308, found 619.2305.

Phenyl 6-*O*-(2,3,4-tri-*O*-benzoyl-β-D-xylopyranosyl)-2,3,4-tri-*O*-benzoyl-1-thio-β-D-glucopyranoside (3eb)

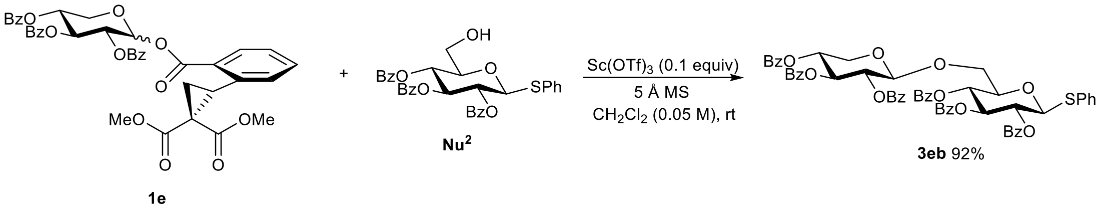

Supplementary Fig. 42 | Synthesis of 3eb.

Following the procedure for **3a**, **Nu2** (29.2 mg, 50 μmol, 1.0 equiv) was transformed into **3eb** (47.3 mg, 46 μmol, 92%) as a colorless syrup after purification by silica gel column chromatography (toluene:EtOAc = 30:1). $[\alpha]_D^{23}$ = –5.5 (c = 1.0, $CHCl_3$); ^{1}H NMR (400 MHz, $CDCl_3$) δ 8.05 – 7.95 (m, 8H), 7.89 (d, J = 7.0 Hz, 2H), 7.75 (d, J = 8.2 Hz, 2H), 7.56 – 7.25 (m, 26H), 5.85 (t, J = 9.5 Hz, 1H), 5.72 (t, J = 6.6 Hz, 1H, H-

3-*xylo*), 5.50 – 5.40 (m, 2H), 5.37 (dd, J = 6.6, 4.9 Hz, 1H, H-2-*xylo*), 5.23 (td, J = 6.3, 3.9 Hz, 1H, H-4-*xylo*), 4.99 – 4.91 (m, 2H, H-1-*gluco*&H-1-*xylo*), 4.40 (dd, J = 12.3, 4.0 Hz, 1H, H-5-*xylo*), 4.10 – 4.00 (m, 2H, H-5-*gluco*&H-6-*gluco*), 3.94 – 3.86 (m, 1H, H-6′-*gluco*), 3.68 (dd, J = 12.3, 6.2 Hz, 1H, H-5′-*gluco*); ^{13}C NMR (100 MHz, $CDCl_3$) δ 165.9, 165.7, 165. 5, 165.3, 165.23, 165.16, 133.6, 133.5, 133.4, 133.3, 131.6, 130.1, 130.02, 129.98, 129.97, 129.9, 129.44, 129.40, 129.38, 129.3, 129.19, 129.16, 129.1, 128.9, 128.7, 128.6, 128.5, 128.44, 128.35, 125.4, 100.1, 86.4, 78.3, 74.4, 70.8, 69.9, 69.4, 69.0, 68.0, 60.9; HRMS (ESI) *m/z* Calcd for $C_{59}H_{48}O_{15}NaS$ [M + Na]$^+$ 1051.2612, found 1051.2607.

***ortho*-Iodobenzoyl 2,3,4-tri-*O*-benzoyl-β-D-xylopyranoside (3ec)**

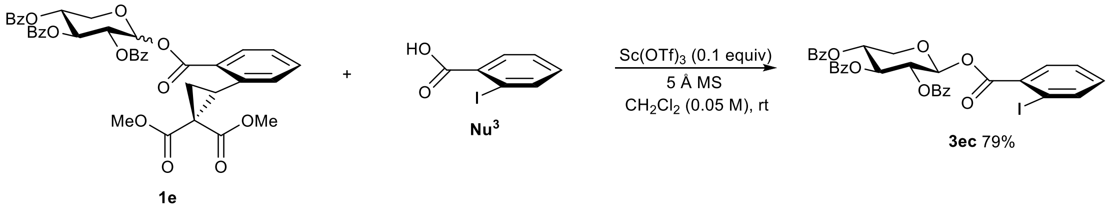

Supplementary Fig. 43 | Synthesis of 3ec.

Following the procedure for **3a**, **Nu^3** (18.6 mg, 75 μmol, 1.5 equiv) was transformed into **3ec** (27.5 mg, 39.5 μmol, 79%) as a colorless syrup after purification by silica gel column chromatography (hexane:EtOAc = 5:1). $[\alpha]_D^{23}$ = –7.9 (c = 1.0, $CHCl_3$); ^{1}H NMR (400 MHz, $CDCl_3$) δ 8.06 – 7.94 (m, 7H), 7.85 (dd, J = 7.8, 1.7 Hz, 1H), 7.59 – 7.50 (m, 3H), 7.41 – 7.28 (m, 7H), 7.17 (td, J = 7.6, 1.7 Hz, 1H), 6.40 (d, J = 4.4 Hz, 1H, H-1), 5.82 (t, J = 5.9 Hz, 1H, H-3), 5.60 (dd, J = 5.9, 4.5 Hz, 1H, H-2), 5.37 (td, J = 5.6, 3.6 Hz, 1H, H-4), 4.58 (dd, J = 12.7, 3.6 Hz, 1H, H-5), 4.02 (dd, J = 12.7, 5.4 Hz, 1H, H-5′); ^{13}C NMR (100 MHz, $CDCl_3$) δ 165.7, 165.2, 165.1, 164.3, 141.8, 133.7, 133.6, 133.5, 133.4, 131.6, 130.2, 130.12, 130.06, 129.2, 129.1, 129.0, 128.59, 128.58, 128.56, 128.1, 94.8, 92.7, 68.9, 68.5, 68.1, 62.1; HRMS (ESI) *m/z* Calcd for $C_{33}H_{25}O_9NaI$ [M + Na]$^+$ 715.0441, found 715.0437.

1-Adamantyl 2′,3′,4′-tri-*O*-benzoyl-α-L-rhamnopyranoside (3fa)

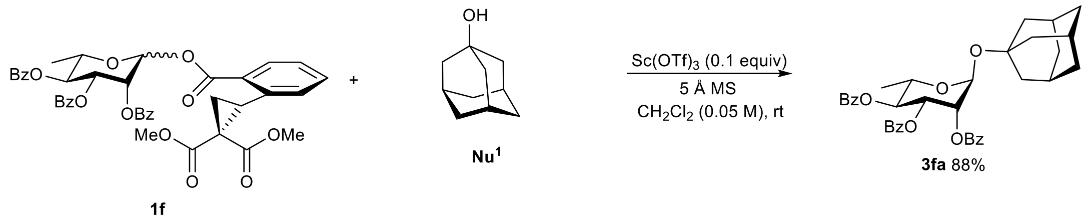

Supplementary Fig. 44 | Synthesis of 3fa.

Following the procedure for **3a**, **Nu1** (7.6 mg, 50 μmol, 1.0 equiv) was transformed into **3fa** (26.9 mg, 44 μmol, 88%) as a white foam after purification by silica gel column chromatography (hexane:EtOAc = 9:1). ^{1}H NMR (400 MHz, $CDCl_3$) δ 8.12 (d, J = 6.9 Hz, 2H), 8.00 (d, J = 7.0 Hz, 2H), 7.84 (d, J = 6.9 Hz, 2H), 7.60 (t, J = 7.4 Hz, 1H), 7.55 – 7.46 (m, 3H), 7.45 – 7.35 (m, 3H), 7.30 – 7.23 (m, 3H), 5.90 (dd, J = 10.1, 3.4 Hz, 1H), 5.66 (t, J = 10.0 Hz, 1H), 5.47 (dd, J = 3.4, 1.9 Hz, 1H), 5.43 (d, J = 1.9 Hz, 1H), 4.38 (dq, J = 9.6, 6.3 Hz, 1H), 2.23 – 2.16 (m, 3H), 1.96 – 1.86 (m, 6H), 1.66 (d, J = 3.2 Hz, 6H), 1.32 (d, J = 6.3 Hz, 3H). The data are identical with the literature.[28]

Phenyl 6-*O*-(2,3,4-tri-*O*-benzoyl-α-L-rhamnopyranosyl)-2,3,4-tri-*O*-benzoyl-1-thio-β-D-glucopyranoside (3fb)

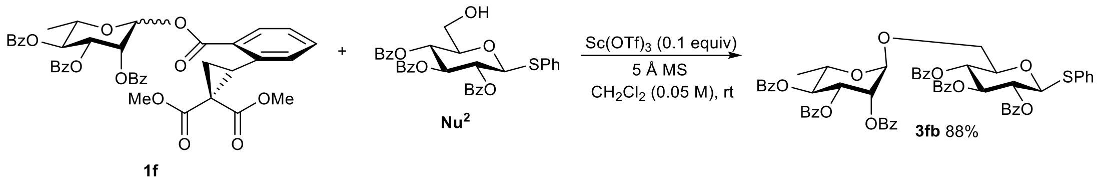

Supplementary Fig. 45 | Synthesis of 3fb.

Following the procedure for **3a**, **Nu2** (29.2 mg, 50 μmol, 1.0 equiv) was transformed into **3fb** (45.8 mg, 44 μmol, 88%) as a colorless syrup after purification by silica gel column chromatography (toluene:EtOAc = 50:1). $[\alpha]_D^{23}$ = +55.2 (c = 1.0, $CHCl_3$); ^{1}H NMR (400 MHz, $CDCl_3$) δ 8.09 (d, J = 9.7 Hz, 2H), 8.02 – 7.95 (m, 6H), 7.86 – 7.80 (m, 4H), 7.61 (t, J = 7.5 Hz, 1H), 7.56 – 7.34 (m, 17H), 7.31 – 7.23 (m, 6H), 5.95 (t, J = 9.5 Hz, 1H), 5.81 (dd, J = 10.1, 3.5 Hz, 1H, H-3-*rhamno*), 5.75 (dd, J = 3.5, 1.7 Hz, 1H, H-2-*rhamno*), 5.64 (t, J = 9.9 Hz, 1H, H-4-*rhanmo*), 5.57 – 5.48 (m, 2H), 5.14 – 5.08 (m, 2H, H-1-*gluco*&H-1-*rhamno*), 4.24 – 4.12 (m, 2H, H-5-*rhamno*&H-5-*gluco*), 3.99 (dd, J = 11.7, 2.3 Hz, 1H, H-6-*gluco*), 3.89 (dd, J = 11.7, 6.6 Hz, 1H, H-6′-*gluco*),

1.27 (d, J = 6.2 Hz, 3H); ^{13}C NMR (100 MHz, $CDCl_3$) δ 165.92, 165.90, 165.5, 165.4, 165.2, 133.7, 133.6, 133.4, 133.3, 133.2, 131.9, 130.04, 130.02, 129.90, 129.88, 129.8, 129.6, 129.42, 129.38, 129.3, 129.1, 128.92, 128.85, 128.72, 128.69, 128.6, 128.5, 128.40, 128.37, 98.4, 86.5, 78.1, 74.4, 71.9, 70.7, 70.6, 70.1, 69.7, 67.3, 67.1, 17.7; HRMS (ESI) *m/z* Calcd for $C_{60}H_{50}O_{15}NaS$ [M + Na]$^+$ 1065.2763, found 1065.2768.

***ortho*-Iodobenzoyl 2,3,4-tri-*O*-benzoyl-α-L-rhamnopyranoside (3fc)**

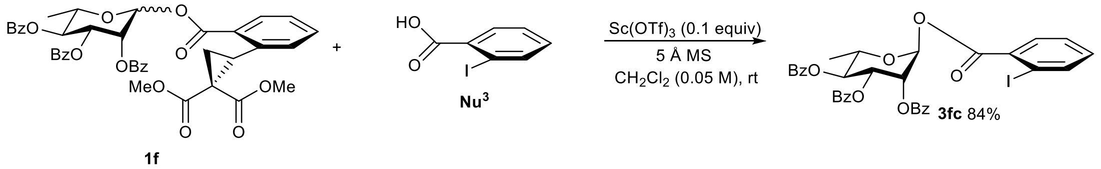

Supplementary Fig. 46 | Synthesis of 3fc.

Following the procedure for **3a**, **Nu³** (18.6 mg, 75 μmol, 1.0 equiv) was transformed into **3fc** (29.6 mg, 42 μmol, 84%) as a colorless syrup after purification by silica gel column chromatography (hexane:EtOAc = 7:1). $[\alpha]_D^{23}$ = +69.2 (c = 1.0, $CHCl_3$); ^{1}H NMR (400 MHz, $CDCl_3$) δ 8.13 (d, J = 6.9 Hz, 2H), 8.09 (dd, J = 8.0, 1.1 Hz, 1H), 8.00 – 7.94 (m, 3H), 7.83 (d, J = 7.0 Hz, 2H), 7.64 (t, J = 7.5 Hz, 1H), 7.56 – 7.49 (m, 4H), 7.44 (t, J = 7.5 Hz, 1H), 7.39 (t, J = 7.8 Hz, 2H), 7.30 – 7.23 (m, 4H), 6.56 (d, J = 1.9 Hz, 1H, H-1), 6.03 (dd, J = 10.2, 3.5 Hz, 1H, H-3), 5.90 (dd, J = 3.5, 1.9 Hz, 1H, H-2), 5.79 (t, J = 10.0 Hz, 1H, H-4), 4.42 (dq, J = 9.7, 6.2 Hz, 1H, H-5), 1.42 (d, J = 6.2 Hz, 3H, H-6); ^{13}C NMR (100 MHz, $CDCl_3$) δ 165.8, 165.7, 165.5, 164.2, 141.9, 134.3, 133.8, 133.6, 133.5, 133.4, 131.9, 130.2, 129.89, 129.86, 129.23, 129.20, 129.1, 128.8, 128.6, 128.5, 128.3, 94.3, 92.2, 71.3, 69.9, 69.8, 69.7, 17.8; HRMS (ESI) *m/z* Calcd for $C_{34}H_{27}O_9NaI$ [M + Na]$^+$ 729.0597, found 729.0601.

1-Adamantyl 2′,3′,5′-tri-*O*-acetyl-β-D-ribofuranoside (3ga)

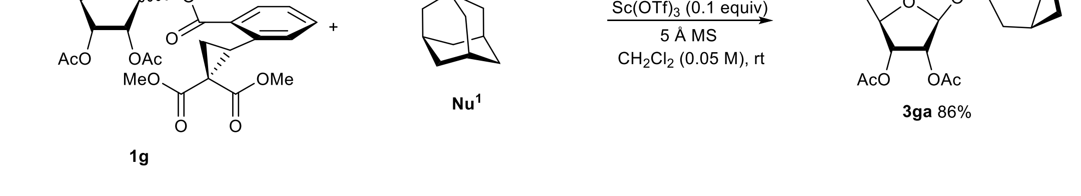

Supplementary Fig. 47 | Synthesis of 3ga.

Following the procedure for **3a**, **Nu¹** (55 mg, 0.359 mmol, 1.0 equiv) was transformed into **3ga** (151.3 mg, 0.309 mmol, 86%) as a white foam after purification by silica gel column chromatography (hexane:EtOAc = 4:1). ^{1}H NMR (400 MHz, $CDCl_3$) δ 5.39 – 5.32 (m, 2H), 5.09 (dd, J = 4.8, 1.3 Hz, 1H), 4.33 (dd, J = 11.2, 3.9 Hz, 1H), 4.24 – 4.10 (m, 2H), 2.11 (dd, J = 16.0, 4.0 Hz, 11H), 2.05 (s, 3H), 1.82 – 1.75 (m, 3H), 1.71 (dd, J = 10.5, 3.2 Hz, 7H), 1.67 – 1.53 (m, 11H). The data are identical with the literature.[28]

Phenyl 6-*O*-(2,3,5-tri-*O*-acetyl-β-D-ribofuranosyl)-2,3,4-tri-*O*-benzoyl-1-thio-β-D-glucopyranoside (3gb)

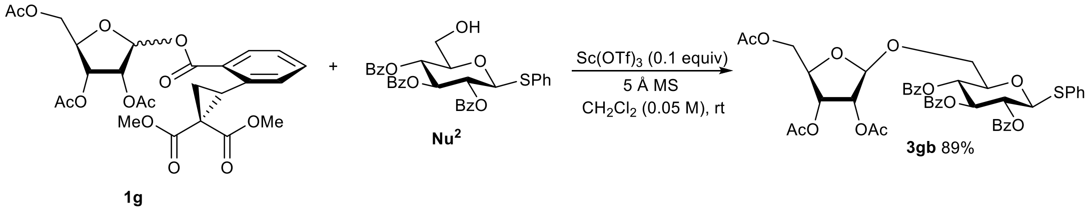

Supplementary Fig. 48 | Synthesis of 3gb.

Following the procedure for **3a**, **Nu²** (29.2 mg, 50 μmol, 1.0 equiv) was transformed into **3gb** (37.4 mg, 44.5 μmol, 89%) as a colorless syrup after purification by silica gel column chromatography (toluene:EtOAc = 9:1). $[\alpha]_D^{23}$ = +1.0 (c = 1.0, $CHCl_3$); ^{1}H NMR (400 MHz, $CDCl_3$) δ 7.99 – 7.94 (m, 2H), 7.94 – 7.89 (m, 2H), 7.82 – 7.76 (m, 2H), 7.54 – 7.47 (m, 4H), 7.44 – 7.31 (m, 8H), 7.28 – 7.23 (m, 2H), 5.88 (t, J = 9.5 Hz, 1H), 5.52 – 5.40 (m, 2H), 5.37 – 5.27 (m, 2H, H-2-*ribo*&H-3-*ribo*), 5.09 (s, 1H, H-1-*ribo*), 5.04 (d, J = 10.0 Hz, 1H, H-1-*gluco*), 4.32 – 4.23 (m, 2H, H-5-*ribo*&H-5-*gluco*), 4.08 (dd, J = 12.9, 7.1 Hz, 1H, H-5′-*ribo*), 4.02 (ddd, J = 9.7, 7.1, 2.2 Hz, 1H, H-4-*ribo*), 3.92 (dd, J = 11.5, 2.2 Hz, 1H, H-6-*gluco*), 3.74 – 3.67 (m, 1H, H-6′-*gluco*), 2.08 (s, 3H), 2.05 (s, 3H), 1.96 (s, 3H); ^{13}C NMR (100 MHz, $CDCl_3$) δ 170.6, 169.7, 169.6, 165.9, 165.4, 165.2, 133.6, 133.4, 133.3, 132.7, 132.3, 130.00, 129.95, 129.8, 129.3, 129.2, 128.91, 128.86, 128.6, 128.5, 128.44, 128.38, 106.0, 86.3, 78.8, 77.9, 74.8, 74.3, 71.5, 70.7, 69.6, 67.4, 64.5, 20.8, 20.7, 20.6; HRMS (ESI) m/z Calcd for $C_{44}H_{42}O_{15}NaS$ $[M + Na]^+$ 865.2142, found 865.2154.

ortho-Iodobenzoyl 2,3,5-tri-*O*-acetyl-β-D-ribofuranoside (3gc)

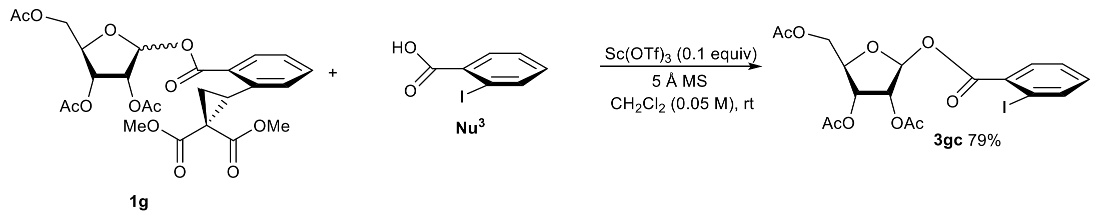

Supplementary Fig. 49 | Synthesis of 3gc.

Following the procedure for **3a**, **Nu**$^{\mathbf{3}}$ (87 mg, 0.352 mmol, 1.0 equiv) was transformed into **3gc** (93.2 mg, 0.278 mmol, 79%) as a colorless syrup after purification by silica gel column chromatography (toluene:EtOAc = 9:1). $[\alpha]_D^{23}$ = –7.2 (*c* = 1.0, $CHCl_3$); ^1H NMR (400 MHz, $CDCl_3$) δ 8.00 (d, *J* = 7.9 Hz, 1H), 7.83 (dd, *J* = 7.8, 1.8 Hz, 1H), 7.42 (t, *J* = 7.6 Hz, 1H), 7.17 (td, *J* = 7.6, 1.8 Hz, 1H), 6.39 (s, 1H, H-1), 5.58 – 5.54 (m, 1H, H-2), 5.50 (dd, *J* = 7.2, 4.8 Hz, 1H, H-3), 4.44 (ddd, *J* = 7.2, 5.0, 3.4 Hz, 1H, H-4), 4.37 (dd, *J* = 12.2, 3.5 Hz, 1H, H-5), 4.16 (dd, *J* = 12.2, 5.0 Hz, 1H, H-5′), 2.15 (s, 3H), 2.07 (s, 3H), 1.92 (s, 3H); ^{13}C NMR (100 MHz, $CDCl_3$) δ 170.6, 169.8, 169.5, 164.3, 141.8, 133.8, 133.5, 131.6, 128.1, 99.3, 94.4, 79.6, 74.2, 70.5, 63.4, 20.7, 20.61, 20.55; HRMS (ESI) *m/z* Calcd for $C_{18}H_{19}O_9NaI$ $[M + Na]^+$ 528.9971, found 528.9962.

1-Adamantyl 2′,3′,5′-tri-*O*-benzoyl-4′-*O*-(2,3,4,6-tetra-*O*-benzoyl-β-D-galactopyranosyl)-β-D-glucopyranoside (3ha)

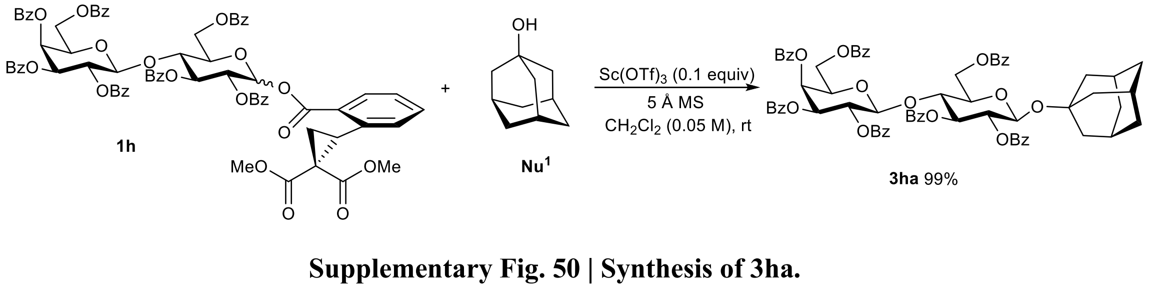

Supplementary Fig. 50 | Synthesis of 3ha.

Following the procedure for **3a**, **Nu**$^{\mathbf{1}}$ (7.6 mg, 50 μmol, 1.0 equiv) was transformed into **3ha** (59.7 mg, 49.5 μmol, 99%) as a white foam after purification by silica gel column chromatography (hexane:EtOAc = 3:1). $[\alpha]_D^{23}$ = +40.5 (*c* = 1.0, $CHCl_3$); ^1H NMR (400 MHz, $CDCl_3$) δ 8.06 – 7.89 (m, 12H), 7.74 (d, *J* = 6.9 Hz, 2H), 7.66 – 7.45 (m, 8H),

7.44 – 7.27 (m, 9H), 7.21 (t, *J* = 7.8 Hz, 2H), 7.13 (t, *J* = 7.7 Hz, 2H), 5.81 (t, *J* = 9.4 Hz, 1H), 5.78 – 5.70 (m, 2H), 5.48 – 5.37 (m, 2H), 5.00 (d, *J* = 8.0 Hz, 1H, H-1-*gluco*), 4.87 (d, *J* = 7.9 Hz, 1H, H-1-*galacto*), 4.57 (dd, *J* = 11.8, 2.0 Hz, 1H, H-6-*galacto*), 4.46 (dd, *J* = 11.7, 6.3 Hz, 1H, H-6′-*galacto*), 4.15 (t, *J* = 9.5 Hz, 1H), 3.94 (t, *J* = 6.6 Hz, 1H, H-5-*galacto*), 3.87 (ddd, *J* = 9.9, 6.4, 2.0 Hz, 1H, H-5-*gluco*), 3.75 (dd, *J* = 11.4, 6.5 Hz, 1H, H-6-*gluco*), 3.64 (dd, *J* = 11.4, 6.8 Hz, 1H, H-6′-*gluco*), 1.99 (s, 3H), 1.76 (d, *J* = 9.4 Hz, 3H), 1.60 (d, *J* = 9.3 Hz, 3H), 1.53 (d, *J* = 12.5 Hz, 3H), 1.45 (d, *J* = 10.9 Hz, 3H); ^{13}C NMR (100 MHz, $CDCl_3$) δ 165.9, 165.7, 165.6, 165.5, 165.3, 165.1, 164.9, 133.7, 133.5, 133.4, 133.3, 133.2, 133.1, 130.1, 129.89, 129.86, 129.81, 129.78, 129.76, 129.71, 129.66, 129.5, 129.0, 128.83, 128.76, 128.7, 128.6, 128.5, 128.44, 128.36, 128.3, 101.2, 94.2, 75.8, 73.5, 72.9, 72.0, 71.9, 71.5, 70.1, 67.7, 63.2, 61.3, 42.4, 36.1, 30.6; HRMS (ESI) *m/z* Calcd for $C_{71}H_{64}O_{18}Na$ $[M + Na]^+$ 1227.3990, found 1227.3995.

Phenyl 6-*O*-[2,3,5-tri-*O*-benzoyl-4-*O*-(2,3,4,6-tetra-*O*-benzoyl-β-D-galactopyranosyl)-β-D-glucopyransyl]-2,3,4-tri-*O*-benzoyl-1-thio-β-D-glucopyranoside (3hb)

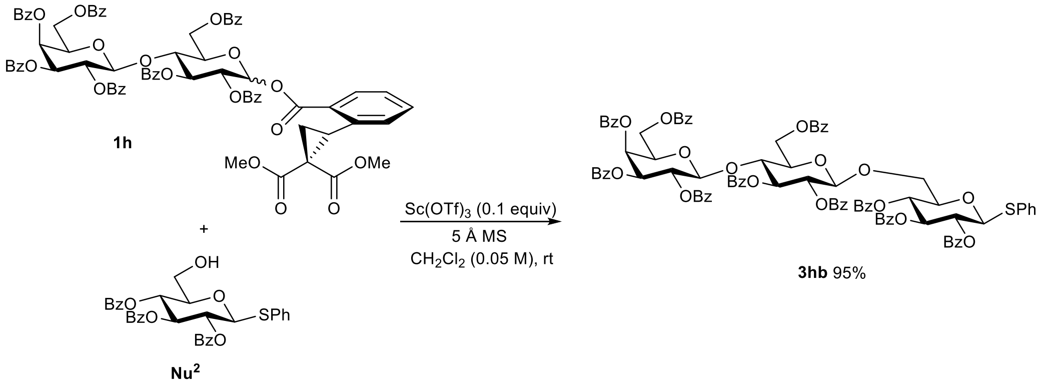

Supplementary Fig. 51 | Synthesis of 3hb.

Following the procedure for **3a**, **Nu²** (29.2 mg, 50 μmol, 1.0 equiv) was transformed into **3hb** (77.4 mg, 47.5 μmol, 95%) as a colorless syrup after purification by silica gel column chromatography (hexane:EtOAc = 15:1). $[\alpha]_D^{23}$ = +31.4 (*c* = 1.0, $CHCl_3$); ^{1}H NMR (400 MHz, Chloroform-*d*) δ 8.07 – 8.00 (m, 8H), 7.98 – 7.92 (m, 6H), 7.83 – 7.79 (m, 2H), 7.78 – 7.72 (m, 4H), 7.66 – 7.32 (m, 31H), 7.30 (d, *J* = 7.7 Hz, 2H), 7.23 – 7.14 (m, 5H), 5.84 – 5.69 (m, 4H), 5.48 – 5.33 (m, 3H), 5.23 (t, *J* = 9.8 Hz, 1H), 4.93

(d, *J* = 9.9 Hz, 1H), 4.87 (d, *J* = 7.8 Hz, 1H), 4.78 (d, *J* = 7.9 Hz, 1H), 4.56 (dd, *J* = 12.2, 1.9 Hz, 1H, H-6-*galacto*), 4.46 (dd, *J* = 12.2, 4.4 Hz, 1H, H-6′-*galacto*), 4.21 (t, *J* = 9.4 Hz, 1H), 3.99 (dt, *J* = 9.6, 4.4 Hz, 1H, H-5-*gluco*), 3.90 (dd, *J* = 11.3, 5.6 Hz, 3H, H-5-*galacto*&H-6-*gluco*&H-6-*gluco′*), 3.75 – 3.64 (m, 3H, H-5-*gluco′*&H-6′-*gluco*&H-6′-*gluco′*); ^{13}C NMR (100 MHz, CDCl3) *δ* 165.9, 165.8, 165.7, 165.51, 165.49, 165.4, 165.3, 165.1, 165.0, 133.7, 133.5, 133.4, 133.3, 133.0, 131.6, 130.1, 130.0, 129.93, 129.87, 129.84, 129.77, 129.6, 129.53, 129.46, 129.4, 129.3, 129.1, 129.04, 128.97, 128.9, 128.8, 128.73, 128.69, 128.66, 128.6, 128.47, 128.45, 128.4, 128.3, 125.4, 101.1, 100.8, 85.8, 79.2, 78.9, 76.1, 74.1, 73.1, 73.0, 71.8, 71.5, 70.7, 70.0, 69.5, 68.0, 67.7, 62.3, 61.2; HRMS (ESI) *m/z* Calcd for $C_{94}H_{76}O_{25}NaS$ [M + Na]$^+$ 1659.4289, found 1659.4291.

ortho-Iodobenzoyl 2,3,5-tri-_O_-benzoyl-4-_O_-(2,3,4,6-tetra-_O_-benzoyl-β-D-galactopyranosyl)-β-D-glucopyranoside (3hc)

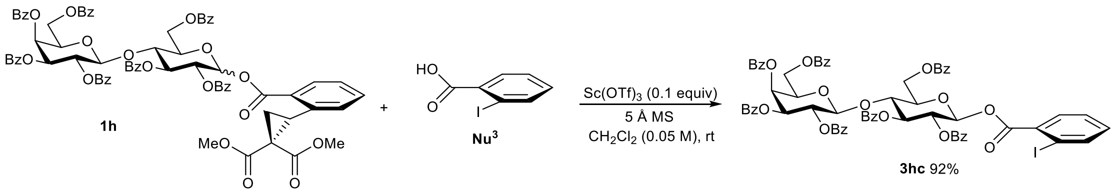

Supplementary Fig. 52 | Synthesis of 3hc.

Following the procedure for **3a**, **Nu³** (18.6 mg, 75 µmol, 1.5 equiv) was transformed into **3hc** (60.1 mg, 46 µmol, 92%) as a colorless syrup after purification by silica gel column chromatography (toluene:EtOAc = 20:1). $[\alpha]_D^{23}$ = +53.9 (*c* = 1.0, CHCl3); ^{1}H NMR (400 MHz, CDCl3) *δ* 8.06 – 7.98 (m, 8H), 7.98 – 7.90 (m, 5H), 7.85 (dd, *J* = 7.9, 1.7 Hz, 1H), 7.75 (d, *J* = 6.9 Hz, 2H), 7.66 – 7.53 (m, 3H), 7.54 – 7.46 (m, 5H), 7.46 – 7.31 (m, 12H), 7.25 – 7.16 (m, 5H), 7.11 (td, *J* = 7.7, 1.7 Hz, 1H), 6.18 (d, *J* = 8.1 Hz, 1H, H-1-*gluco*), 5.94 (t, *J* = 9.3 Hz, 1H), 5.82 – 5.72 (m, 3H), 5.40 (dd, *J* = 10.4, 3.4 Hz, 1H, H-3-*galacto*), 4.91 (d, *J* = 7.9 Hz, 1H, H-1-*galacto*), 4.62 (dd, *J* = 12.5, 2.0 Hz, 1H, H-6-*galacto*), 4.54 (dd, *J* = 12.4, 4.1 Hz, 1H, H-6′-*galacto*), 4.38 (t, *J* = 9.4 Hz, 1H), 4.06 (ddd, *J* = 9.9, 4.1, 2.0 Hz, 1H, H-5-*gluco*), 3.91 (t, *J* = 6.7 Hz, 1H, H-5-*galacto*), 3.82 – 3.67 (m, 2H, H-6-*gluco*&H-6′-*gluco*); ^{13}C NMR (100 MHz, CDCl3) *δ*

165.9, 165.7, 165.6, 165.40, 165.38, 165.3, 165.0, 163.8, 141.9, 133.7, 133.6, 133.5, 133.4, 132.3, 132.0, 130.1, 130.0, 129.9, 129.83, 129.80, 129.6, 129.5, 129.2, 129.1, 129.0, 128.9, 128.73, 128.69, 128.6, 128.41, 128.38, 128.2, 101.2, 95.1, 93.0, 79.3, 75.7, 74.1, 73.0, 71.9, 71.5, 70.8, 70.0, 67.6, 62.2, 61.1.; HRMS (ESI) *m/z* Calcd for $C_{68}H_{53}O_{19}NaI$ $[M + Na]^+$ 1323.2123, found 1323.2134.

Section 5. Divergent synthesis of chitooligosaccharides.

3′,6′-Di-*O*-benzyl-4′-*O*-acetyl-2′-deoxy-2′-phthalimido-β-D-glucopyranosyl *ortho*-2,2-dimethoxycarbonylcyclopropylbenzoate (7)

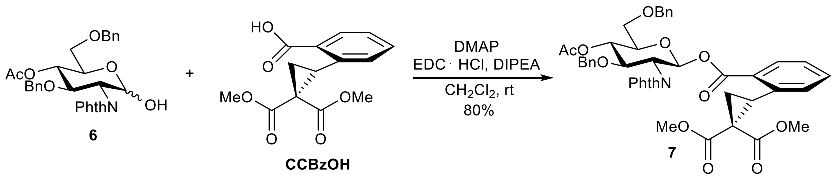

Supplementary Fig. 53 | Synthesis of 7.

Following the procedure for **1a**, 3,5-di-*O*-benzyl-4-*O*-acetyl-2-deoxy-2-phthalimido-D-glucopyranose **6**[29] (3.54 g, 6.66 mmol, 1.0 equiv) was transformed into **7** (4.21 g, 5.33 mmol, 80%, β_1:β_2 = 1.4:1.0) as a white foam after purification by silica gel column chromatography (hexane:EtOAc = 2:1 to 3:2). ^{1}H NMR (400 MHz, $CDCl_3$) *δ* 7.88 (dd, *J* = 7.8, 1.5 Hz, 1H), 7.79 (dd, *J* = 7.9, 1.4 Hz, 0.6H), 7.73 – 7.59 (m, 6.5H), 7.41 – 7.22 (m, 11.9H), 7.17 – 7.12 (m, 1.6H), 7.04 – 6.99 (m, 3.2H), 6.98 – 6.87 (m, 4.9H), 6.51 (d, *J* = 8.5 Hz, 1.6H), 5.34 – 5.26 (m, 1.8H), 4.74 – 4.49 (m, 8.2H), 4.37 (d, *J* = 12.1 Hz, 1.6H), 4.01 – 3.92 (m, 1.6H), 3.81 (s, 3H), 3.79 – 3.70 (m, 2.9H), 3.70 – 3.58 (m, 3.9H), 3.26 (s, 1.8H), 2.64 (s, 3H), 2.09 – 2.00 (m, 2.3H), 1.98 – 1.93 (m, 4.8H), 1.75 (dd, *J* = 9.0, 5.1 Hz, 1H), 1.70 (dd, *J* = 9.0, 5.1 Hz, 0.7H); ^{13}C NMR (100 MHz, $CDCl_3$) *δ* 170.3, 170.2, 169.71, 169.67, 167.8, 167.5, 166.7, 164.2, 163.6, 137.9, 137.8, 137.5, 137.3, 134.1, 133.8, 132.7, 132.6, 131.9, 131. 5, 131.2, 131.0, 130.2, 129.62, 129.57, 129.4, 128.5, 128.4, 128.22, 128.20, 128.1, 128.0, 127.80, 127.75, 127.7, 127.58, 127.56, 123.6, 123.3, 90.5, 74.2, 74.1, 74.0, 73.71, 73.69, 72.20, 72.15, 69.3, 69.2, 54.8, 54.3, 52.8, 52.7, 52.4, 51.3, 36.3, 35.7, 32.3, 32.1, 21.0, 19.74, 19.68.; HRMS (ESI) *m/z* Calcd for $C_{44}H_{41}NO_{13}Na$ $[M + Na]^+$ 814.2476, found 814.2496.

Benzyl 3,6-di-*O*-benzyl-4-*O*-(3,6-di-*O*-benzyl-4-*O*-acetyl-2-deoxy-2-phthalimido-β-D-glucopyranosyl)-2-deoxy-2-phthalimido-β-D-glucopyranoside (9)

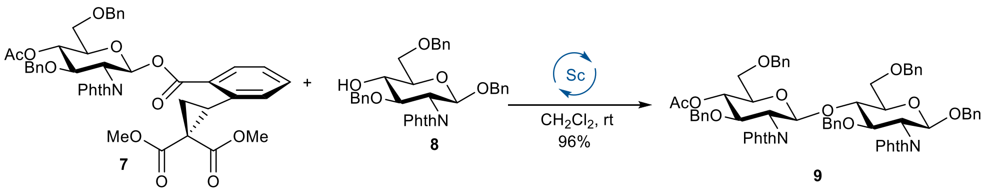

Supplementary Fig. 54 | Synthesis of 9.

Following the procedure for **3a**, **8**[30] (39.4 mg, 68 µmol, 1.0 equiv) was glycosylated with **7** (64.6 mg, 82 µmol, 1.2 equiv) to deliver **9** (86.3 mg, 65 µmol, 96%) as a white foam after purification by silica gel column chromatography (hexane:EtOAc = 2:1 to toluene:EtOAc = 9:1 to ether:hexane = 3:2). ^{1}H NMR (400 MHz, $CDCl_3$) *δ* 7.88 – 7.57 (m, 8H), 7.40 – 7.30 (m, 8H), 7.30 – 7.26 (m, 2H), 7.07 – 6.88 (m, 13H), 6.84 – 6.79 (m, 3H), 5.33 (d, *J* = 8.4 Hz, 1H), 5.15 (t, *J* = 9.4 Hz, 1H), 4.95 (d, *J* = 6.7 Hz, 1H), 4.82 (d, *J* = 12.5 Hz, 1H), 4.68 (d, *J* = 12.3 Hz, 1H), 4.60 (d, *J* = 12.1 Hz, 1H), 4.57 – 4.43 (m, 6H), 4.38 – 4.26 (m, 3H), 4.17 (d, *J* = 6.4 Hz, 3H), 3.60 – 3.50 (m, 3H), 3.48 – 3.77 (m, 2H), 3.31 (d, *J* = 7.8 Hz, 1H), 1.92 (s, 3H). The data are identical with the literature.[30]

Benzyl 3,6-di-*O*-benzyl-4-*O*-(3,6-di-*O*-benzyl-2-deoxy-2-phthalimido-β-D-glucopyranosyl)-2-deoxy-2-phthalimido-β-D-glucopyranoside (10)

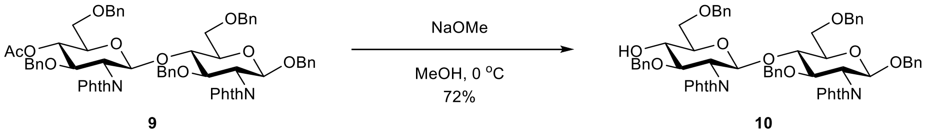

Supplementary Fig. 55 | Synthesis of 10.

To a solution of **9** (563 mg, 0.51 mmol, 1.0 equiv) in anhydrous MeOH/THF (v/v = 1:1. 20 mL) was added NaOMe (1M solution in MeOH, 1 mL, 1 mmol, 1.96 equiv). The mixture was stirred at 0 °C until all starting material was consumed. The reaction was then quenched with AcOH, concentrated *in vauco* and the residue was purified by silica gel column chromatography to give the titled compound **10** (379 mg, 0.367 mmol, 72%) as a white foam. ^{1}H NMR (400 MHz, $CDCl_3$) *δ* 7.95 – 7.56 (m, 7H), 7.40 – 7.27 (m, 9H), 7.07 – 6.92 (m, 12H), 6.87 – 6.78 (m, 3H), 5.31 (d, *J* = 8.2 Hz, 1H), 4.95 (d, *J* = 7.9 Hz, 1H), 4.83 – 4.73 (m, 2H), 4.69 (d, *J* = 12.4 Hz, 1H), 4.58 – 4.43 (m, 6H), 4.37

(d, J = 12.3 Hz, 1H), 4.26 (dd, J = 10.8, 8.2 Hz, 1H), 4.22 – 4.09 (m, 4H), 3.86 – 3.78 (m, 1H), 3.71 (dd, J = 10.0, 4.4 Hz, 1H), 3.60 – 3.50 (m, 2H), 3.44 (dd, J = 11.1, 3.9 Hz, 1H), 3.38 (ddd, J = 10.1, 6.1, 4.4 Hz, 1H), 3.34 – 3.26 (m, 1H), 3.10 (d, J = 2.2 Hz, 1H). The data are identical with the literature.[30]

Benzyl 3,6-di-*O*-benzyl-4-*O*-[3,6-di-*O*-benzyl-4-*O*-(3,6-di-*O*-benzyl-4-*O*-acetyl-2-deoxy-2-phthalimido-β-D-glucopyrabosyl)-2-deoxy-2-phthalimido-β-D-glucopyranosyl]-2-deoxy-2-phthalimido-β-D-glucopyranoside (11)

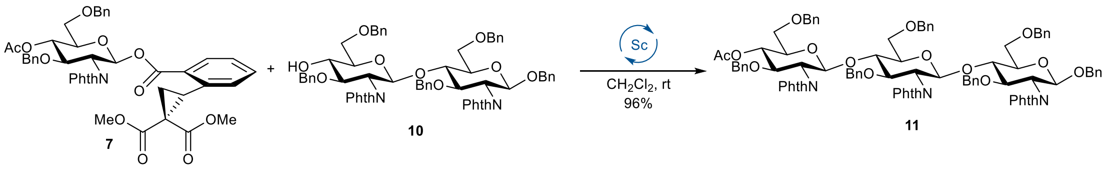

Supplementary Fig. 56 | Synthesis of 11.

Following the procedure for **3a**, **10** (379 mg, 0.36 mmol, 1.0 equiv) was glycosylated with **7** (345 mg, 0.433 mmol, 1.2 equiv) to deliver **11** (518 mg, 0.274 mmol, 96%) as a white foam after purification by silica gel column chromatography (hexane:EtOAc = 2:1 to toluene:EtOAc = 8:1). $[\alpha]_D^{22}$ = +19.5 (c = 1.0, $CHCl_3$); ^1H NMR (400 MHz, $CDCl_3$) δ 7.92 – 7.60 (m, 11H), 7.31 – 7.26 (m, 8H), 7.23 – 7.18 (m, 3H), 7.16 – 7.10 (m, 1H), 7.07 – 6.81 (m, 21H), 6.70 – 6.61 (m, 3H), 5.32 (d, J = 8.3 Hz, 1H, H-1), 5.14 (t, J = 9.4 Hz, 1H), 5.09 (d, J = 7.6 Hz, 1H, H-1′), 4.90 (d, J = 8.0 Hz, 1H, H-1″), 4.87 (d, J = 12.4 Hz, 1H), 4.71 (d, J = 12.8 Hz, 1H), 4.65 (d, J = 12.4 Hz, 1H), 4.60 (d, J = 12.1 Hz, 1H), 4.54 (d, J = 12.2 Hz, 1H), 4.50 – 4.24 (m, 11H), 4.21 – 4.02 (m, 6H), 3.58 – 3.46 (m, 3H), 3.45 – 3.30 (m, 3H), 3.29 – 3.19 (m, 1H), 3.12 (dd, J = 11.3, 3.1 Hz, 1H), 2.94 – 2.85 (m, 1H), 1.89 (s, 3H); ^{13}C NMR (100 MHz, $CDCl_3$) δ 169.8, 168.4, 167.7, 138.9, 138.8, 138.6, 138.5, 138.3, 137.9, 137.3, 134.2, 133.8, 133.5, 131.8, 128.5, 128.4, 128.3, 128.22, 128.16, 128.08, 128.05, 128.0, 127.84, 127.78, 127.7, 127.6, 127.5, 127.4, 127.3, 127.2, 127.0, 126.8, 123.7, 123.2, 97.2, 96.8, 76.6, 76.2, 75.6, 74.7, 74.6, 74.5, 74.4, 74.1, 73.7, 73.3, 73.0, 72.7, 72.5, 70.6, 69.6, 68.3, 67.1, 56.8, 56.4, 55.9, 21.0; HRMS (ESI) m/z Calcd for $C_{93}H_{85}N_3O_{20}Na$ $[M + Na]^+$ 1586.5619, found 1586.5653.

Benzyl 3,6-di-*O*-benzyl-4-*O*-[3,6-di-*O*-benzyl-4-*O*-(3,6-di-*O*-benzyl-2-deoxy-2-acetamido-β-D-glucopyranosyl)-2-deoxy-2-acetamido-β-D-glucopyranosyl]-2-deoxy-2-acetamido-β-D-glucopyranoside (13)

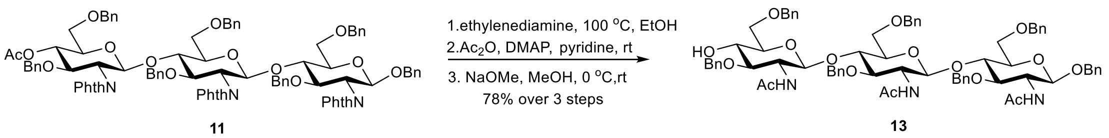

Supplementary Fig. 57 | Synthesis of 13.

To a suspension of **11** (383 mg, 0.245 mmol, 1.0 equiv) in EtOH (10 mL) was added ethylenediamine (0.245 mL, 3.675 mmol, 15.0 equiv). The mixture was heated to 100 ^{o}C, and the mixture was stirred at this temperature overnight before it was cooled up to rt. The mixture was then concentrated *in vauco*. The residue was dissolved in anhydrous pyridine (4.0 mL). To the solution were sequentially added Ac_2O (2.0 mL) and DMAP (30 mg, 0.245 mmol, 1.0 equiv). The mixture was stirred at room temperature overnight before it was concentrated *in vauco* to give a crude product, which was purified by silica gel column chromatography (EtOAc:CH_2Cl_2 = 2:1) to give **12** (218 mg, 0.206 mmol, 84%) as a white foam. Following the procedure for **10**, the compound **12** (273 mg, 0.21 mmol, equiv) was transformed into **13** (245.4 mg, 0.195 mmol, 93%) as a white foam after purification by silica gel column chromatography (EtOAc:CH_2Cl_2 = 2:1). $[\alpha]_D^{23}$ = –19.3 (c = 1.0, $CHCl_3$); ^{1}H NMR (400 MHz, $CDCl_3$) δ 7.40 – 7.17 (m, 37H), 6.27 (d, J = 9.0 Hz, 1H, AcNH″), 5.86 (d, J = 9.2 Hz, 1H, AcNH′), 4.88 – 4.76 (m, 3H), 4.63 (d, J = 8.0 Hz, 1H, H-1) 4.68 – 4.39 (m, 12H), 4.29 (d, J = 7.6 Hz, 1H, H-1′), 4.27 – 4.21 (m, 1H, H-2″), 4.08 (d, J = 8.2, 1H, H-1″) 4.08 – 4.01 (m, 1H, H-2′), 4.01 – 3.91(m, 2H), 3.83 – 3.61 (m, 8H, H-3′&H-3″), 3.57 – 3.49 (m, 2H), 3.48 – 3.37 (m, 2H), 3.30 – 3.02 (m, 3H), 1.95 (s, 3H), 1.79 (s, 3H), 1.67 (s, 3H); ^{13}C NMR (100 MHz, $CDCl_3$) δ 170.8, 170.7, 170.3, 139.0, 138.7, 138.6, 138.5, 138.1, 137.8, 137.5, 128.72, 128.69, 128.63, 128.56, 128.5, 128.44, 128.41, 128.36, 128.3, 128.2, 128.14, 128.10, 128.03, 127.97, 127.89, 127.85, 127.68, 127.67, 127.65, 127.5, 100.7, 99.7, 99.3, 81.0, 79.7, 78.8, 75.2, 74.7, 74.12, 74.08, 74.0, 73.8, 73.7, 73.6, 73.0, 72.7, 72.1, 71.0, 70.3, 70.0, 69.2, 54.5, 53.4, 51.3, 23.6, 23.4, 23.3.; HRMS (ESI) *m/z* Calcd for $C_{73}H_{84}N_3O_{16}$ [M + H]$^+$ 1258.5852, found 1258.5859.

Phenyl 3,4,6-tri-*O*-benzyl-2-deoxy-2-benzyloxymethanamido-1-seleno-α-D-glucopyranoside (14)

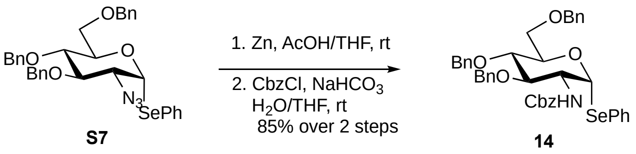

Supplementary Fig. 58 | Synthesis of 14.

To a solution of **S7**[31] (614 mg, 1.0 mmol, 1.0 equiv) in a mixture of THF and AcOH (v/v = 9:1, 10 mL) was added zinc dust (654 mg, 10.0 mmol, 10.0 equiv). The mixture was stirred at room temperature for 5 h before the solid was filtered off. The volatile was concentrated *in vauco*. The residue was dissolved in EtOAc and the organic layer was washed with sat. $NaHCO_3$ solution. The organic phase was dried over Na_2SO_4, filtered and concentrated *in vauco*. The crude mixture was dissolved in THF and water (v/v = 1:1, 10 mL). To the solution were added $NaHCO_3$ (336 mg, 4.0 mmol, 4.0 equiv) and benzyl chloroformate (CbzCl, 0.28 mL, 2.0 mmol, 2.0 equiv). The mixture was stirred at room temperature for 2 h before the volatile was removed. The aqueous phase was extracted with EtOAc, the organic phase was dried over Na_2SO_4, filtered and concentrated *in vauco*. The residue was purified by silica gel column chromatography (hexane:EtOAc = 6:1) to give the titled compound **14** (614.3 mg, 0.85 mmol, 85%) as a white foam. $[\alpha]_D^{22}$ = +17.2 (c = 1.0, $CHCl_3$); ^{1}H NMR (400 MHz, $CDCl_3$) δ 7.57 – 7.50 (m, 2H), 7.39 – 7.17 (m, 25H), 5.88 (d, J = 4.7 Hz, 1H, H-1), 5.13 (d, J = 12.2 Hz, 1H), 5.03 (d, J = 12.3 Hz, 1H), 4.88 – 4.76 (m, 3H), 4.69 (d, J = 11.2 Hz, 1H, H-1), 4.64 (d, J = 12.0 Hz, 1H), 4.57 (d, J = 10.8 Hz, 1H), 4.49 (d, J = 12.0 Hz, 1H), 4.20 – 4.16 (m, 2H, H-2&CbzNH), 3.88 – 3.77 (m, 2H, H-3&H-6), 3.69 (dd, J = 11.0, 2.1 Hz, 1H, H-6′), 3.54 – 3.46 (m, 1H, H-5); ^{13}C NMR (100 MHz, $CDCl_3$) δ 155.8, 138.1, 136.5, 134.1, 129.3, 128.7, 128.64, 128.60, 128.5, 128.3, 128.2, 128.1, 128.0, 127.9, 127.8, 89.7, 81.4, 78.1, 75.2, 74.6, 73.6, 68.5, 67.1, 55.6.; HRMS (ESI) *m/z* Calcd for $C_{41}H_{41}O_6NNaSe$ $[M + Na]^+$ 746.1991, found 746.1983.

3′,4′,6′-Tri-*O*-benzyl-2′-deoxy-2′-benzyloxymethanamido-D-glucopyranosyl

ortho-2,2-dimethoxycarbonylcyclopropylbenzoate (15)

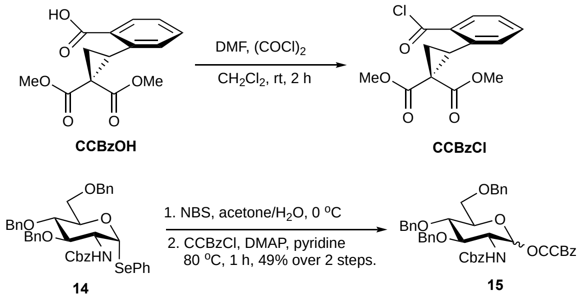

Supplementary Fig. 59 | Synthesis of 15.

CCBzOH (334 mg, 1.2 mmol, 1.0 equiv) was dissolved in anhydrous CH_2Cl_2 (6 mL). To the solution were added 2 drops of DMF and $(COCl)_2$ (0.155 mL, 1.8 mmol, 1.5 equiv) dropwise under the ice bath. The reaction mixture was stirred at room temperature for 2 h. After completion of the reaction, the mixture was concentrated *in vauco* to afford the CCBzCl, which was directly used without further purification.

To a solution of compound **14** (704 mg, 1.0 mmol, 1.0 equiv) in a mixed solvent of acetone and H_2O (v/v = 9:1, 10 mL) was added *N*-bromosuccinimide (NBS, 178 mg, 1.0 mmol, 1.0 equiv) under the ice bath. The reaction mixture was stirred under ice bath for 1 min before the reaction was quenched with sat. $NaHCO_3$ and sat. $Na_2S_2O_3$ solution (***Caution: Extension of the reaction time will cause decomposition of product!***). The aqueous phase was extracted with CH_2Cl_2, the organic phase was combined, washed with brine, dried over Na_2SO_4, filtered and concentrated *in vauco* to afford the hemiacetal. The residue was dissolved in anhydrous pyridine (10 mL) and to the mixture were added DMAP (12.2 mg, 0.1 mmol, 0.1 equiv) and a solution of CCBzCl obtained from last step in CH_2Cl_2 (2 mL). The reaction mixture was sealed and heated to 80 °C and stirred at this temperature for 1 h. The mixture was then concentrated *in vauco*. The residue was dissolved by EtOAc and washed sequentially with 1 M HCl solution and sat. $NaHCO_3$ solution. The organic phases were combined, dried over Na_2SO_4, filtered and concentrated *in vauco*. The residue was purified by silica gel column chromatography (hexane:EtOAc = 2.5:1 followed by toluene:EtOAc = 8:1) to afford the titled compound **15** (413.5 mg, 0.49 mmol, 49% over 2 steps) as a white foam. ^{1}H NMR (400 MHz, $CDCl_3$) δ 8.12 – 7.93 (m, 1.4H), 7.55 – 7.37 (m, 1.8H), 7.36 – 7.00

(m, 36.3H), 5.95 (d, J = 7.7 Hz, 1 H), 5.82 (d, J = 8.5 Hz, 1H), 5.17 – 4.88 (m, 4.6H), 4.85 – 4.75 (m, 3.2H), 4.75 – 4.66 (m, 1.6H), 4.66 – 4.54 (m, 3.2H), 4.54 – 4.44 (m, 1.6H), 3.91 – 3.63 (m, 14.5H), 3.26 (s, 1.4H), 3.06 (s, 3H), 2.22 – 2.16 (m, 1.4H), 1.83 – 1.80 (m, 1.6H); ^{13}C NMR (100 MHz, $CDCl_3$) δ 170.5, 170.4, 167.4, 167.3, 164.8, 164.6, 156.1, 156.0, 138.1, 137.3, 136.8, 136.4, 132.64, 132.58, 131.6, 130.3, 130.0, 128.53, 128.49, 128.47, 128.45, 128.2, 128.10, 128.08, 128.0, 127.92, 127.88, 127.81, 127.78, 127.7, 93.2, 77.9, 77.7, 77.4, 75.7, 74.8, 74.7, 73.62, 73.58, 68.7, 68.5, 66.9, 52.9, 52.8, 52.5, 52.0, 36.5, 36.4, 32.8, 32.4, 20.0, 19.8; HRMS (ESI) *m/z* Calcd for $C_{49}H_{49}O_{12}NNa$ $[M + Na]^+$ 866.3152, found 866.3176.

Benzyl 3,6-di-*O*-benzyl-4-*O*-{3,6-di-*O*-benzyl-4-*O*-[3,6-di-*O*-benzyl-4-(3,4,6-tri-*O*-benzyl-2-deoxy-2-benzyloxymethanamido-β-D-glucopyranosyl)-2-deoxy-2-acetamido-β-D-glucopyranosyl]-2-deoxy-2-acetamido-β-D-glucopyranosyl}-2-deoxy-2-acetamido-β-D-glucopyranoside (16)

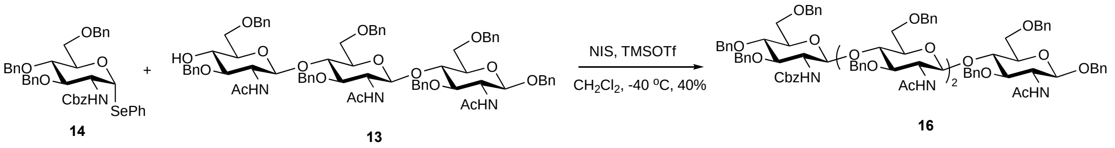

Supplementary Fig. 60 | Synthesis of 16.

To a solution of **13** (212 mg, 0.169 mmol, 1.0 equiv) and **14** (167 mg, 0.231 mmol, 1.37 equiv) in anhydrous CH_2Cl_2 (2 mL) was added freshly activated 4 Å MS (200 mg). The mixture was stirred at room temperature for 20 min before it was cooled to -40 °C. To the mixture was added NIS (76 mg, 0.338 mmol, 2.0 equiv) and TMSOTf (6.1 μL, 33.8 μmol, 0.2 equiv), the mixture was stirred at -40 °C for 2 h before it was quenched by sat. Na_2SO_3 solution and sat. $NaHCO_3$ solution. Two phases were separated and the aqueous phase was extracted with CH_2Cl_2, the organic phases were combined, dried over Na_2SO_4, filtered and concentrated *in vauco*. The residue was purified by silica gel column chromatography (CH_2Cl_2:EtOAc = 1:2) to give the titled compound **16** (124.3 mg, 68 μmol, 40%) as a white foam. ^{1}H NMR (400 MHz, $CDCl_3$) δ 7.34 – 7.19 (m, 59H), 6.47 – 6.27 (m, 1H), 5.87 (s, 1H), 5.13 (d, J = 12.4 Hz, 1H), 5.04 (d, J = 12.2 Hz,

1H), 4.87 – 4.79 (m, 3H), 4.76 – 4.71 (m, 2H), 4.68 – 4.64 (s, 2H), 4.63 – 4.44 (m, 11H), 4.42 – 4.36 (m, 4H), 4.33 – 4.22 (m, 4H), 4.11 – 4.04 (m, 2H), 4.00 – 3.91 (m, 3H), 3.80 – 3.70 (m, 5H), 3.66 – 3.52 (m, 7H), 3.48 – 3.35 (m, 4H), 3.28 – 3.06 (m, 3H), 1.95 (s, 3H), 1.78 (s, 3H), 1.63 (s, 3H); ^{13}C NMR (100 MHz, $CDCl_3$) δ 170.9, 170.8, 170.4, 156.2, 139.3, 138.8, 138.7, 138.24, 138.21, 138.15, 138.1, 137.9, 137.8, 137.5, 136.6, 128.83, 128.75, 128.69, 128.65, 128.6, 128.54, 128.49, 128.45, 128.43, 128.41, 128.36, 128.29, 128.26, 128.21, 128.18, 128.12, 128.05, 128.01, 127.99, 127.95, 127.9, 127.8, 127.7, 127.64, 127.62, 127.57, 127.54, 127.50, 100.6, 99.7, 79.7, 78.5, 75.2, 74.8, 74.5, 74.1, 74.0, 73.7, 73.6, 73.5, 73.0, 72.1, 71.0, 70.3, 70.2, 69.1, 68.8, 67.0, 57.8, 54.4, 53.5, 51.0, 50.7, 23.6, 23.5, 23.3. The data are identical with the literature.[32]

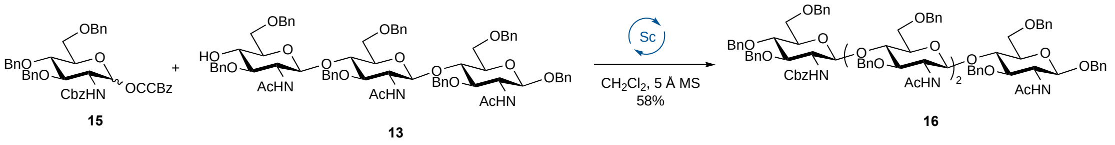

Supplementary Fig. 61 | Alternative synthesis of 16.

Alternatively, the titled tetrasaccharide **16** could be prepared from the glycosylation between acceptor **13** (32 mg, 25 µmol, 1.0 equiv) and glycosyl CCBz **15** (63.3 mg, 75 µmol, 3.0 equiv) in 58% yield (31.2 mg, 14.5 µmol).

4-*O*-{4-*O*-[4-*O*-(2-Deoxy-2-amino-β-D-glucopyranosyl)-2-deoxy-2-acetamido-β-D-glucopyranosyl]-2-deoxy-2-acetamido-β-D-glucopyranosyl}-2-deoxy-2-acetamido-D-glucopyranose (17)

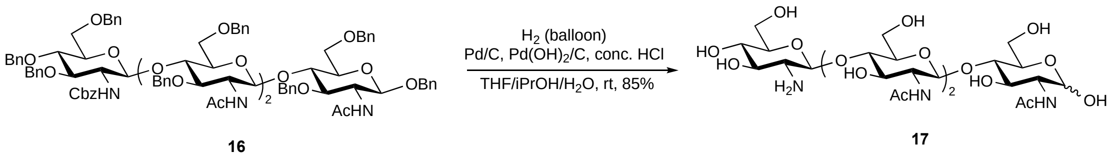

Supplementary Fig. 62 | Synthesis of 17.

To a solution of **16** (40 mg, 22 µmol, 1.0 equiv) in mixed solvent of THF/iPrOH/H_2O (v/v/v = 1:1:1, 6 mL) was added Pd/C (10%, 400 mg, 100 w%) and $Pd(OH)_2/C$ (20%, 200 mg, 100 w%) and a drop of conc. HCl. The mixture was evacuated and backfilled with hydrogen 3 times. The mixture was stirred at room temperature for 48 h before it was filtered with a cotton plug. The residue was concentrated *in vauco* to give the crude

product, which was purified by silica gel column chromatography (iPrOH/20% ammonia/H_2O = 7:2:1) to give the titled compound **17** (14.7 mg, 18.7 μmol, 85%, α:β = 1:2) as a white foam. ^{1}H NMR (400 MHz, D_2O) δ 5.18 (s, 0.4 H), 4.62 – 4.57 (m, 2.4H), 4.48 (d, J = 8.1 Hz, 1H), 3.98 – 3.33 (m, 23.4H), 2.67 (t, J = 8.7 Hz, 1H), 2.05 (d, J = 9.9 Hz, 9H); ^{13}C NMR (100 MHz, D_2O) δ 174.8, 174.7, 174.5, 102.7, 101.3, 94.8, 90.5, 79.7, 79.2, 79.1, 79.0, 78.0, 76.2, 75.6, 74.7, 74.6, 74.5, 72.5, 72.1, 72.0, 70.1, 69.61, 69.58, 69.3, 60.7, 60.1, 60.02, 59.99, 59.97, 56.7, 56.2, 55.3, 55.2, 53.7, 22.3, 22.2, 22.0. The data are identical with the literature.[32]

3′,6′-Di-*O*-benzyl-4′-*O*-*tert*-butyldimethylsilyl-2′-deoxy-2′-phthalimido-β-D-glucopyranosyl *ortho*-2,2-dimethoxycarbonylcyclopropylbenzoate (19)

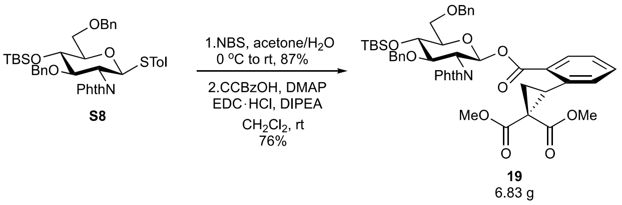

Supplementary Fig. 63 | Synthesis of 19.

To a solution of **S8**[33] (11.24 g, 15.83 mmol, 1.0 equiv,) in a mixture of acetone/H_2O (v/v = 6:1, 140 mL) was added NBS (4.23 g, 23.75 mmol, 1.5 equiv) under an ice bath. The reaction was warmed up to room temperature and was stirred at this temperature for another 2 h before the reaction was quenched with sat. Na_2SO_3 solution. Two phases were separated and the organic phase was washed with sat. $NaHCO_3$, dried over Na_2SO_4, filtered and concentrated *in vauco*. The residue was purified by silica gel column chromatography (7.77 g, 13.77 mmol, 87%) as a white foam. Following the procedure for **1a**, 3,5-di-*O*-benzyl-4-*O*-*tert*-butyldimethylsilyl-2-deoxy-2-phthalimido-D-glucopyranoside (6.3 g, 10.43 mmol, 1.0 equiv) was transformed into **19** (6.83 g, 7.93 mmol, 76%, β_1:β_2 = 1.4:1.0) as a white foam after purification by silica gel column chromatography (hexane:EtOAc = 4:1 to 3:1). ^{1}H NMR (400 MHz, $CDCl_3$) δ 7.86 (dd, J = 7.9, 1.5 Hz, 1.6H), 7.75 (dd, J = 7.8, 1.4 Hz, 1H), 7.66 – 7.53 (m, 10.8H), 7.40 – 7.28 (m, 13.5H), 7.25 – 7.18 (m, 3.4H), 7.13 (t, J = 6.6 Hz, 2.7H), 7.03 – 6.96 (m, 5.3H), 6.95 – 6.87 (m, 5.5H), 6.86 – 6.79 (m, 2.7H), 6.53 – 6.47 (m, 2.5H), 4.88 – 4.76 (m,

2.6H), 4.73 – 4.60 (m, 2.6H), 4.57 – 4.50 (m, 4.2H), 4.50 – 4.41 (m, 3.6H), 4.39 – 4.29 (m, 2.6H), 3.96 – 3.62 (m, 21.8H), 3.58 (t, J = 8.7 Hz, 1H), 3.23 (s, 3H), 2.61 (s, 4.8H), 2.04 – 1.99 (m, 2.6H), 1.75 (dd, J = 9.0, 5.1 Hz, 1.7H), 1.69 (dd, J = 9.0, 5.1 Hz, 1H), 0.90 – 0.86 (m, 23.8H), 0.14 – –0.04 (m, 15.8H); ^{13}C NMR (100 MHz, $CDCl_3$) δ 170.2, 168.0, 166.8, 164.1, 163.7, 138.43, 138.38, 137.5, 137.4, 133.9, 133.6, 132.5, 132.4, 131.9, 131.5, 131.1, 130.8, 130.3, 129.9, 129.6, 129.3, 128.5, 128.4, 128.1, 127.9, 127.8, 127.69, 127.65, 127.6, 127.24, 127.17, 123.5, 123.2, 90.7, 90.6, 81.0, 75.5, 73.5, 73.4, 72.3, 72.2, 68.7, 55.4, 55.0, 52.8, 52. 5, 51.3, 36.2, 35.8, 32.4, 32.1, 26.1, 19.8, 19.7, 18.14, 18.12, -3.7, -4.4; HRMS (ESI) m/z Calcd for $C_{48}H_{53}NO_{12}NaSi$ $[M + Na]^+$ 886.3235, found 886.3203.

para-Tolyl 3-_O_-benzoyl-6-_O_-benzyl-4-_O_-(3,6-di-_O_-benzyl-4-_O_-_tert_-butyldimethylsilyl-2-deoxy-2-phthalimido-β-D-glucopyranosyl)-2-deoxy-2-phthalimido-1-thio-β-D-glucopyranoside (21)

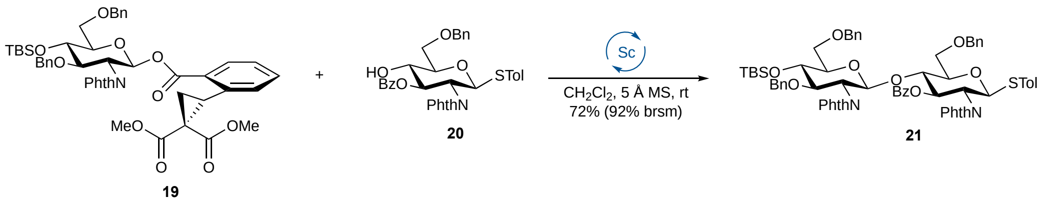

Supplementary Fig. 64 | Synthesis of 21.

Following the procedure for **3a**, **20**[34] (4.07 g, 6.7 mmol, 1.0 equiv) was glycosylated with **19** (6.77 g, 7.83 mmol, 1.17 equiv) to deliver **21** (5.19 g, 4.82 mmol, 72%, 92% brsm) as a white foam after purification by silica gel column chromatography (toluene:EtOAc = 20:1 to hexane:EtOAc = 4:1). $[\alpha]_D^{23}$ = +47.8 (c = 1.0, $CHCl_3$); ^{1}H NMR (400 MHz, $CDCl_3$) δ 7.91 – 7.83 (m, 3H), 7.74 – 7.62 (m, 4H), 7.61 – 7.55 (m, 2H), 7.46 (d, J = 7.4 Hz, 1H), 7.36 – 7.23 (m, 13H), 7.21 – 7.16 (m, 2H), 6.95 – 6.79 (m, 7H), 5.99 (t, J = 9.5 Hz, 1H, H-3), 5.61 (d, J = 10.5 Hz, 1H, H-1), 5.37 – 5.27 (m, 1H, H-1′), 4.67 (d, J = 12.2 Hz, 1H), 4.46 – 4.24 (m, 5H, H-2), 4.22 – 4.13 (m, 2H), 4.04 – 3.97 (m, 2H, H-4&H-2′), 3.69 – 3.63 (m, 1H, H-5), 3.54 – 3.42 (m, 3H, H-6), 3.28 (dd, J = 10.8, 1.9 Hz, 1H), 3.10 – 3.03 (m, 1H, H-6), 2.98 (dd, J = 10.7, 5.2 Hz, 1H, H-6′), 2.23 (s, 3H), 0.78 (s, 9H), -0.04 (s, 3H), -0.12 (s, 3H); ^{13}C NMR (100 MHz, $CDCl_3$) δ

168.0, 167.2, 165.5, 138.7, 138.5, 138.3, 138.2, 134.2, 134.0, 133.7, 133.6, 132.9, 130.0, 129.9, 129.7, 128.4, 128.34, 128.30, 128.0, 127.8, 127.58, 127.56, 127.44, 127.41, 127.3, 127.1, 123.7, 123.6, 97.1, 83.3, 80.9, 78.9, 76.3, 75.3, 74.3, 73.1, 73.1, 73.0, 72.2, 68.2, 56.4, 54.3, 26.0, 21.2, 18.0, -3.7, -4.6; HRMS (ESI) *m/z* Calcd for $C_{69}H_{70}N_2O_{13}NaSi$ $[M + Na]^+$ 1217.4266, found 1217.4274.

***para*-Tolyl 3-*O*-benzoyl-6-*O*-benzyl-4-*O*-(3,6-di-*O*-benzyl-2-deoxy-2-phthalimido-β-D-glucopyranosyl)-2-deoxy-2-phthalimido-1-thio-β-D-glucopyranoside (22)**

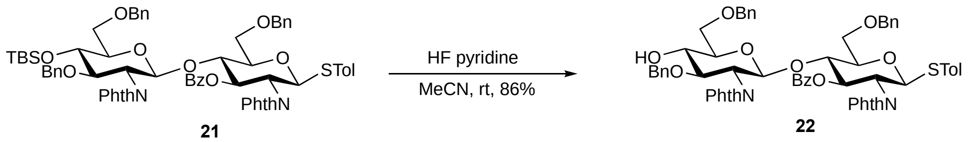

Supplementary Fig. 65 | Synthesis of 22.

To a solution of **21** (2.0 g, 1.87 mmol, 1.0 equiv) in anhydrous MeCN (20 mL) was added 70% HF solution in pyridine (2.4 mL, 18.7 mmol, 10.0 equiv). The mixture was stirred at room temperature overnight. The mixture was poured into water and the aqueous phase was extracted with EtOAc, the organic phase was washed with sat. $NaHCO_3$ solution, dried over Na_2SO_4, filtered and concentrated *in vauco*. The residue was purified by silica gel column chromatography (hexane:EtOAc = 2:1) to give the titled compound **21** (1.56 g, 1.61 mmol, 86%) as a white foam. $[\alpha]_D^{23}$ = +58.2 (c = 1.0, $CHCl_3$); ^1H NMR (400 MHz, $CDCl_3$) δ 7.92 – 7.75 (m, 4H), 7.70 – 7.47 (m, 7H), 7.36 – 7.27 (m, 8H), 7.25 – 7.16 (m, 4H), 7.15 – 7.09 (m, 2H), 6.98 – 6.91 (m, 4H), 6.91 – 6.84 (m, 3H), 5.98 (dd, J = 10.2, 8.7 Hz, 1H, H-3), 5.59 (d, J = 10.5 Hz, 1H, H-1), 5.40 – 5.33 (m, 1H, H-1′), 4.71 (d, J = 12.2 Hz, 1H), 4.42 (d, J = 12.2 Hz, 1H), 4.32 (t, J = 10.3 Hz, 1H, H-2), 4.27 (d, J = 11.8 Hz, 1H), 4.24 – 4.15 (m, 3H), 4.15 – 4.08 (m, 1H, H-4&H-2′), 4.07 – 3.97 (m, 2H), 3.66 – 3.56 (m, 2H, H-5&H-3′), 3.50 – 3.36 (m, 2H, H-6&H-6), 3.23 (dd, J = 9.4, 3.9 Hz, 1H, H-6′), 3.15 (d, J = 1.7 Hz, 1H, -OH), 3.06 (dd, J = 9.3, 7.2 Hz, 1H, H-6′), 3.00 (ddd, J = 9.2, 7.2, 3.9 Hz, 1H, H-4′), 2.24 (s, 3H); ^{13}C NMR (100 MHz, $CDCl_3$) δ 167.9, 167.2, 165.2, 138.5, 138.34, 138.29, 137.4, 134.3, 134.0, 133.9, 133.8, 133.3, 131.9, 131.4, 129.8, 129.7, 129.6, 128.61, 128.57, 128.3, 128.1, 128.0, 127.9, 127.7, 127.5, 127.44, 127.36, 123.7, 123.5, 98.2, 83.2, 78.7, 78.0,

75.9, 75.3, 74.3, 73.8, 73.7, 72.9, 72.1, 71.5, 67.8, 55.8, 54.2, 21.2; HRMS (ESI) *m/z* Calcd for $C_{63}H_{56}N_2O_{13}Na$ [M + Na]$^+$ 1103.3401, found 1103.3450.

3′-*O*-Benzoyl-6′-*O*-benzyl-4′-*O*-(3,6-di-*O*-benzyl-4-*O*-*tert*-butyldimethylsilyl-2-deoxy-2-phthalimido-β-D-glucopyranosyl)-2′-deoxy-2′-phthalimido-β-D-glucopyranosyl *ortho*-2,2-dimethoxycarbonylcyclopropylbenzoate (23)

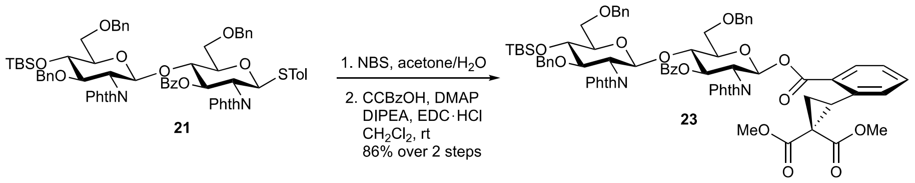

Supplementary Fig. 66 | Synthesis of 23.

To a solution of **21** (2.13 g, 1.78 mmol, 1.0 equiv) in aqueous acetone (v/v = 6:1, 28 mL) was added NBS (423 g, 2.38 mmol, 1.34 equiv). The mixture was stirred at room temperature for 2 h before the reaction was quenched with sat. Na_2SO_3 solution and sat. $NaHCO_3$ solution. The aqueous phase was extracted with CH_2Cl_2, the organic phase was dried over anhydrous Na_2SO_4, filtered and concentrated *in vauco*. The crude product was purified by silica gel column chromatography (hexane:EtOAc = 3:2) to give the hemiacetal, which was coupled with CCBzOH (724 mg, 2.6 mmol, 1.46 equiv) following the procedure for **1a** to give **23** (2.01 g, 1.53 mmol, 86% over 2 steps) as a white foam after purification by silica gel column chromatography (hexane:EtOAc = 3:2). ^{1}H NMR (400 MHz, $CDCl_3$) δ 7.97 – 7.91 (m, 2H), 7.86 (dd, *J* = 7.8, 1.5 Hz, 0.8H), 7.78 – 7.43 (m, 10H), 7.35 (dddd, *J* = 30.8, 13.2, 7.8, 3.7 Hz, 10H), 7.23 (d, *J* = 7.4 Hz, 2H), 7.13 (dd, *J* = 10.8, 7.7 Hz, 1H), 6.95 – 6.80 (m, 5H), 6.65 (dd, *J* = 8.8, 5.3 Hz, 1H), 6.15 (ddd, *J* = 10.5, 9.0, 3.2 Hz, 1H), 5.37 (d, *J* = 7.5 Hz, 1H), 4.77 – 4.62 (m, 2H), 4.50 (d, *J* = 12.4 Hz, 1H), 4.47 – 4.34 (m, 4H), 4.20 (d, *J* = 12.2 Hz, 1H), 4.10 – 3.97 (m, 2H), 3.87 – 3.80 (m, 1H), 3.77 (s, 2H), 3.74 – 3.33 (m, 7H), 3.21 – 3.14 (m, 2H), 3.02 (ddd, *J* = 10.7, 7.7, 5.8 Hz, 1H), 2.61 (s, 2H), 2.00 (ddd, *J* = 17.7, 8.5, 5.1 Hz, 1H), 1.78 – 1.68 (m, 3H), 0.80 (s, 9H), -0.11 (d, *J* = 3.1 Hz, 3H); ^{13}C NMR (100 MHz, $CDCl_3$) δ 170.2, 170.1, 167.7, 167.5, 166.8, 165.48, 165.46, 164.0, 163.5, 138.8, 138.33, 138.28, 138.2, 137.6, 137.4, 134.2, 133.8, 133.0, 132.9, 132.6, 132.5, 131.0, 130.8,

130.3, 130.1, 130.00, 129.96, 129.8, 129.7, 129.3, 129.2, 128.5, 128.4, 128.33, 128.30, 128.0, 127.8, 127.73, 127.68, 127.6, 127.50, 127.46, 127.3, 127.1, 123.7, 123.4, 96.7, 90.33, 90.29, 80.9, 76.2, 75.4, 75.2, 75.1, 73.38, 73.35, 73.11, 73.08, 72.5, 71.8, 71.7, 68.5, 68.0, 67.8, 56.4, 54.4, 54.1, 52.9, 52.7, 52.4, 51.3, 36.2, 35.6, 32.3, 32.1, 26.0, 19.7, 19.5, 18.0, -3.7, -4.5; HRMS (ESI) *m/z* Calcd for $C_{76}H_{76}N_2O_{19}NaSi$ $[M + Na]^+$ 1371.4709, found 1371.4648.

***para*-Tolyl 3-*O*-benzoyl-6-*O*-benzyl-4-*O*-{3,6-di-*O*-benzyl-4-*O*-[3-*O*-benzoyl-6-*O*-benzyl-4-*O*-(3,6-di-*O*-benzyl-4-*O*-*tert*-butyldimethylsilyl-2-deoxy-2-phthalimido-β-D-glucopyranosyl)-2-deoxy-2-phthalimido-β-D-glucopyranosyl]-2-deoxy-2-phthalimido-β-D-glucopyranosyl}-2-deoxy-2-phthalimido-1-thio-β-D-glucopyranoside (24)**

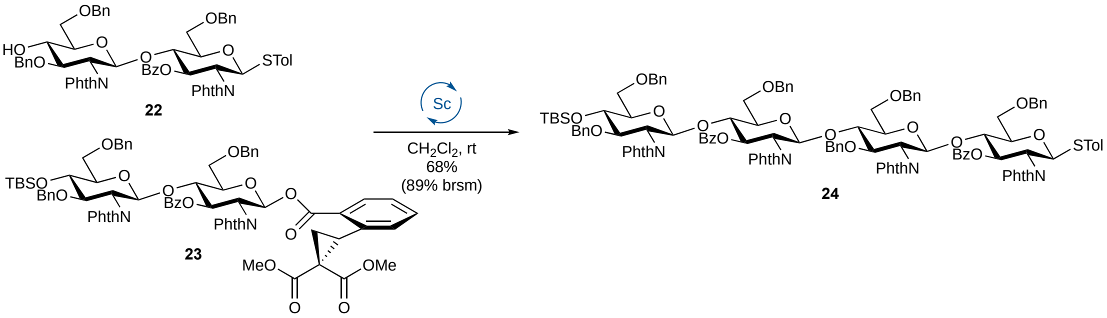

Supplementary Fig. 67 | Synthesis of 24.

Following the procedure for **3a**, **22** (1.076 g, 0.9 mmol, 1.0 equiv) was glycosylated with **23** (1.64 g, 1.19 mmol, 1.3 equiv) to deliver **24** (1.22 g, 0.612 mmol, 68%, 89% brsm) as a white foam after purification by silica gel column chromatography (hexane:EtOAc = 2:1 to toluene:EtOAc = 9:1). $[\alpha]_D^{23}$ = +45.1 (*c* = 1.0, $CHCl_3$); ^1H NMR (400 MHz, $CDCl_3$) *δ* 7.96 – 7.54 (m, 20H), 7.54 – 7.41 (m, 3H), 7.39 – 7.26 (m, 5H), 7.25 – 7.12 (m, 11H), 7.10 – 7.04 (m, 3H), 7.02 – 6.93 (m, 6H), 6.92 – 6.76 (m, 10H), 6.71 – 6.57 (m, 3H), 5.94 (t, *J* = 9.4 Hz, 1H, H-3), 5.80 (dd, *J* = 10.6, 8.8 Hz, 1H, H-3″), 5.56 (d, *J* = 10.5 Hz, 1H, H-1), 5.26 (d, *J* = 7.5 Hz, 1H, H-1′), 5.23 – 5.11 (m, 2H, H-1″&H-1‴), 4.74 – 4.62 (m, 2H), 4.39 – 4.14 (m, 10H), 4.10 – 3.81 (m, 10H), 3.69 (s, 1H), 3.60 (dd, *J* = 9.9, 3.5 Hz, 1H), 3.48 – 3.32 (m, 3H), 3.27 – 3.13 (m, 2H), 3.02 – 2.94 (m, 2H), 2.90 (dd, *J* = 10.6, 5.3 Hz, 1H), 2.83 – 2.75 (m, 2H), 2.21 (s, 3H), 0.76

(s, 9H), -0.05 (s, 3H), -0.14 (s, 3H); ^{13}C NMR (100 MHz, $CDCl_3$) δ 169.23, 169.17, 167.9, 167.4, 167.2, 165.4, 165.0, 148.8, 138.7, 138.5, 138.43, 138.37, 138.32, 138.28, 138.2, 134.4, 134.1, 133.9, 133.7, 133.5, 132.9, 132.7, 131.9, 131.7, 131.4, 130.02, 129.98, 129.7, 129.6, 129.1, 128.6, 128.30, 128.28, 128.17, 128.15, 128.10, 128.06, 128.0, 127.9, 127.7, 127.6, 127.52, 127.48, 127.43, 127.40, 127.30, 127.25, 127.2, 127.1, 126.8, 126.0, 123.7, 123.4, 123.1, 122.1, 97.8, 97.1, 95.9, 83.2, 80.9, 78.7, 78.6, 75.9, 75.3, 75.2, 74.6, 74.5, 74.3, 74.2, 74.0, 72.9, 72.8, 72.5, 72.4, 72.31, 72.26, 68.1, 67.7, 67.4, 66.3, 56.4, 56.3, 55.9, 54.3, 53.1, 53.0, 47.8, 25.9, 21.2, 18.0, -3.8, -4.6; HRMS (ESI) *m/z* Calcd for $C_{125}H_{119}N_4O_{26}SSi$ $[M + H]^+$ 2151.7597, found 2151.7556.

Section 6. NMR spectra.

^{1}H spectrum for **S4**.

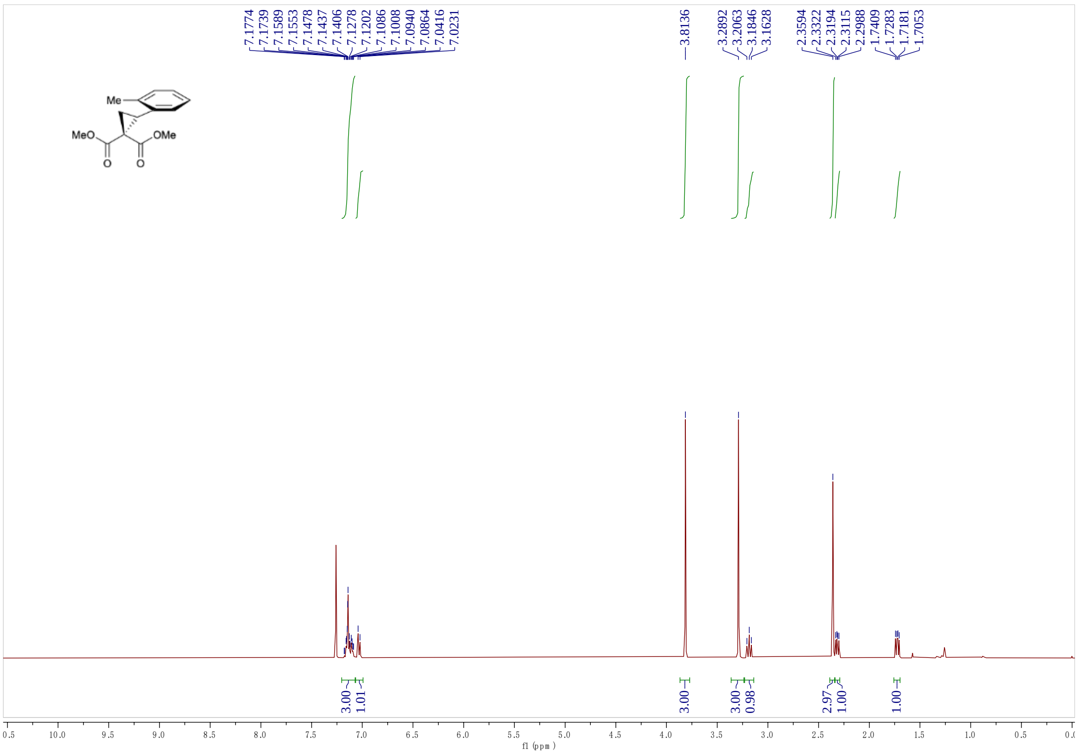

Supplementary Fig. 68 | ^{1}H NMR spectrum of S4 (400 MHz, 25 °C, $CDCl_3$).

^{1}H and ^{13}C spectra for **CCBzOH**.

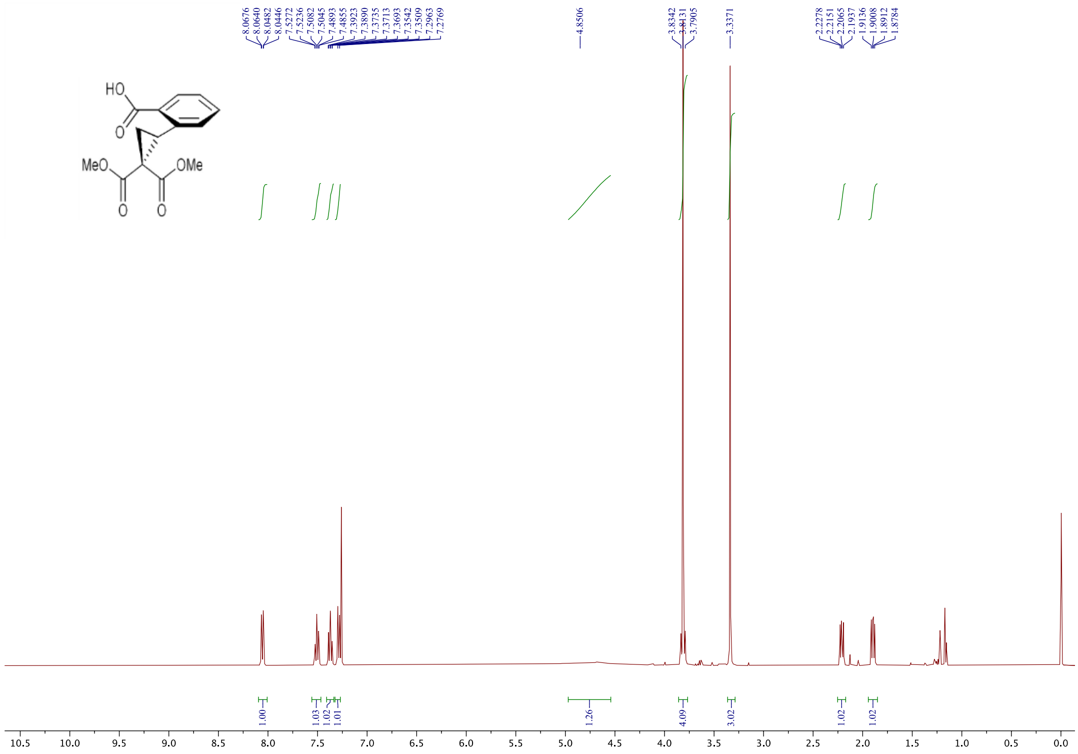

Supplementary Fig. 69 | ^{1}H NMR spectrum of CCBzOH (400 MHz, 25 °C, $CDCl_3$).

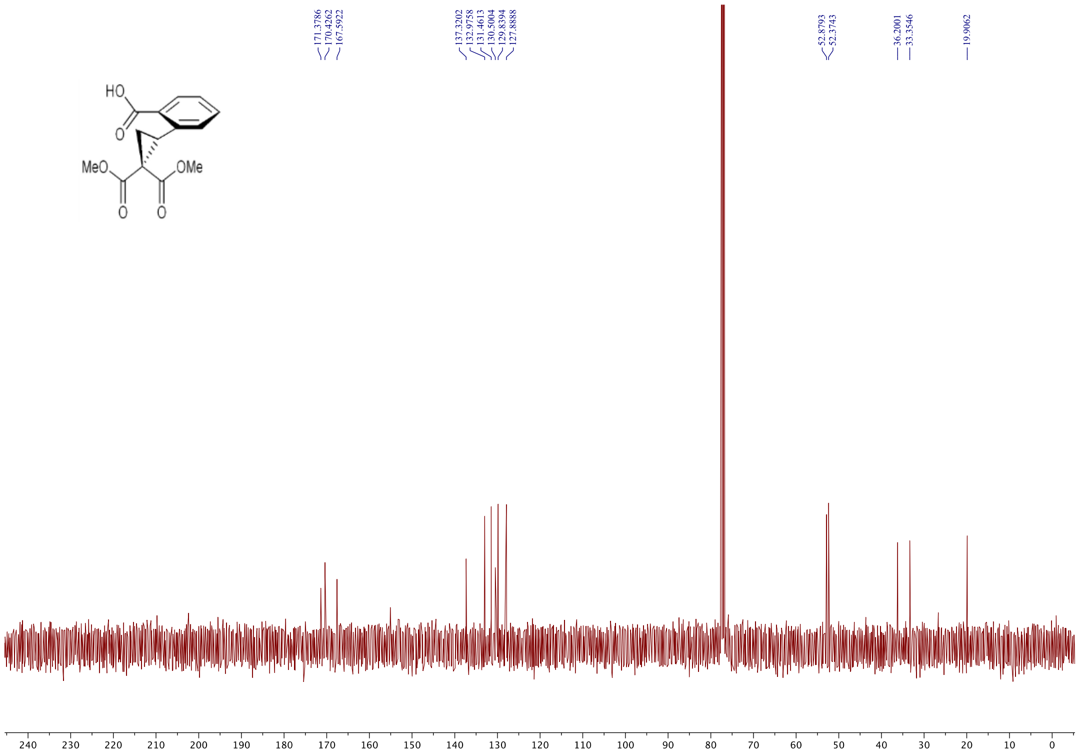

Supplementary Fig. 70 | ^{13}C NMR spectrum of CCBzOH (100 MHz, 25 °C, $CDCl_3$).

^{1}H and ^{13}C spectra for **1a**.

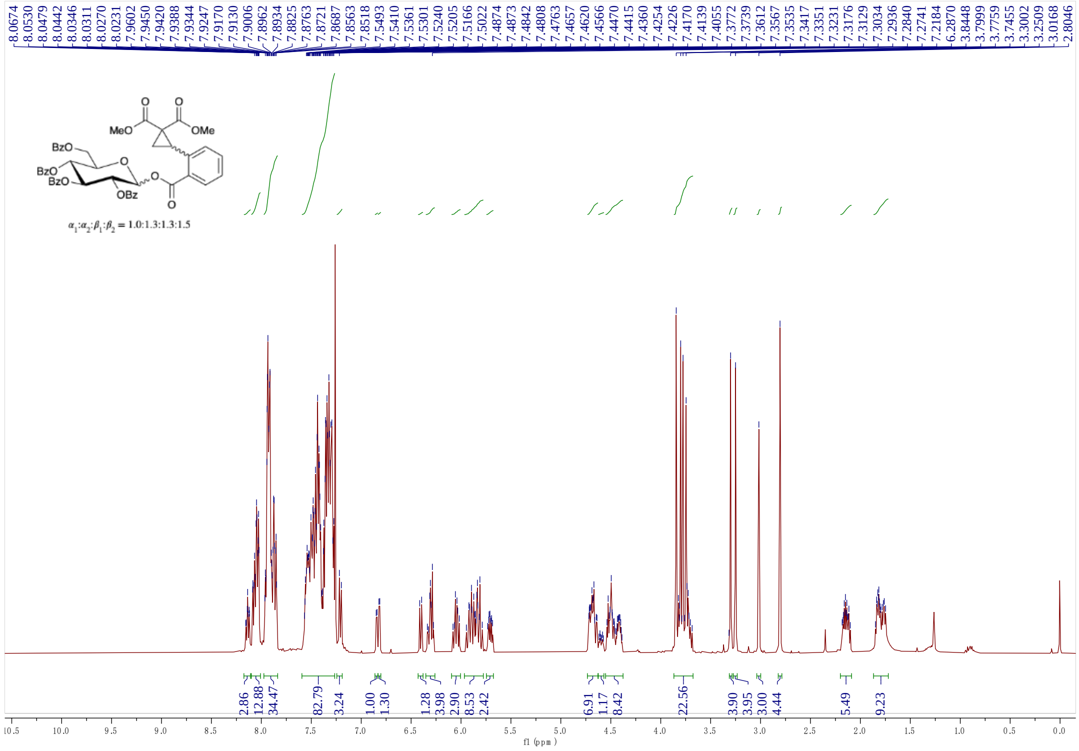

Supplementary Fig. 71 | ^{1}H NMR spectrum of 1a (400 MHz, 25 °C, $CDCl_3$).

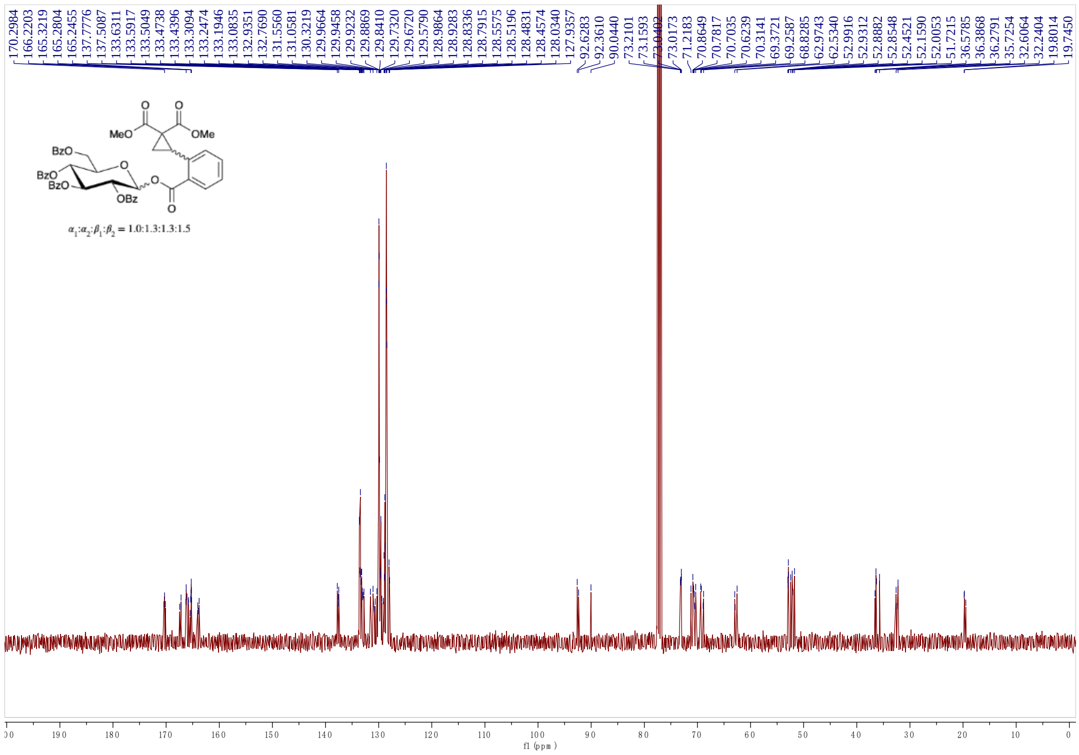

Supplementary Fig. 72 | ^{13}C NMR spectrum of 1a (100 MHz, 25 °C, $CDCl_3$).

^{1}H and ^{13}C spectra for **1b**.

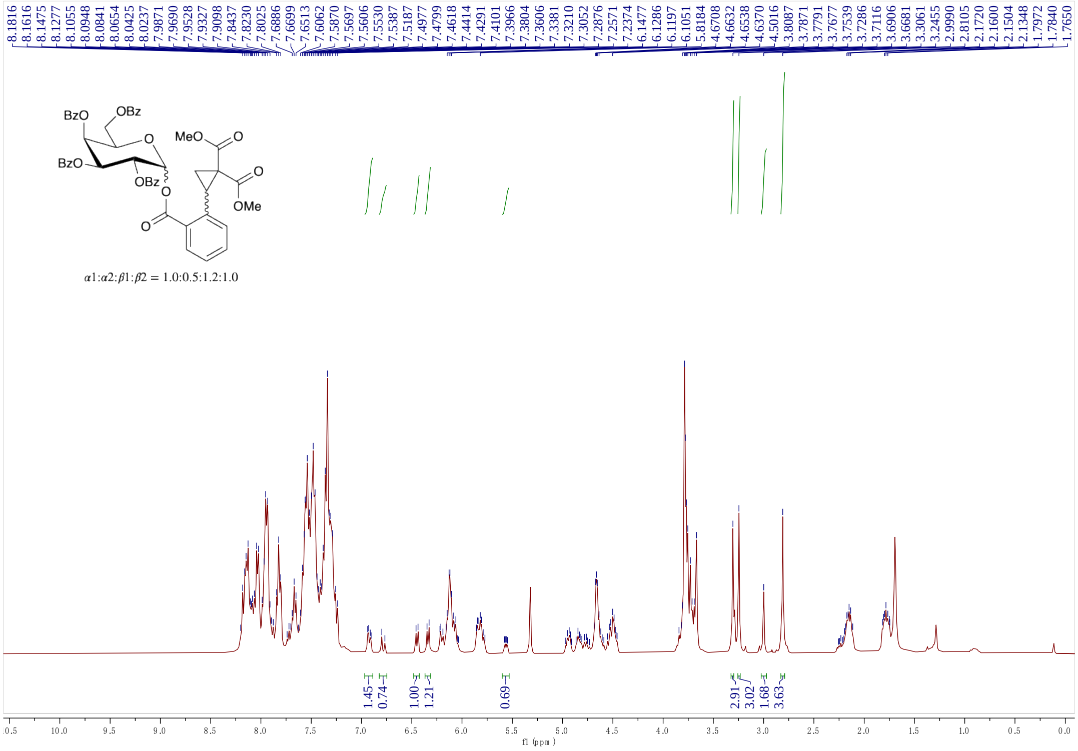

Supplementary Fig. 73 | ^{1}H NMR spectrum of 1b (400 MHz, 25 °C, CD_2Cl_2).

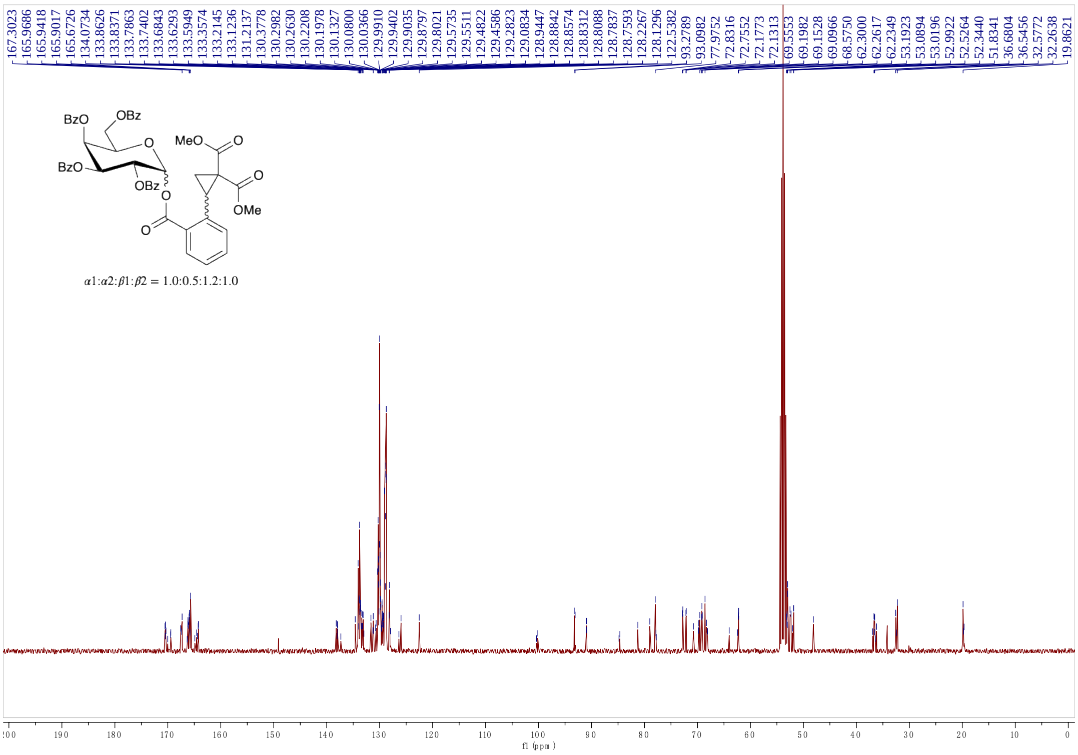

Supplementary Fig. 74 | ^{13}C NMR spectrum of 1b (100 MHz, 25 °C, CD_2Cl_2).

^{1}H and ^{13}C spectra for **1c**.

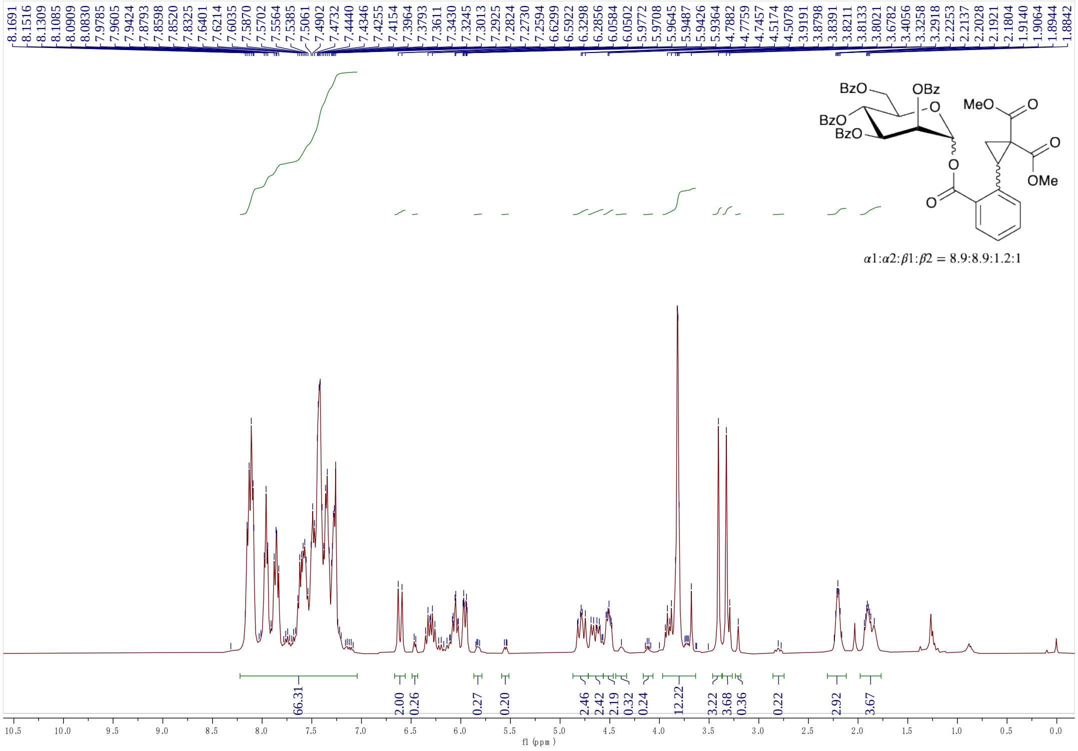

Supplementary Fig. 75 | ^{1}H NMR spectrum of 1c (400 MHz, 25 °C, $CDCl_3$).

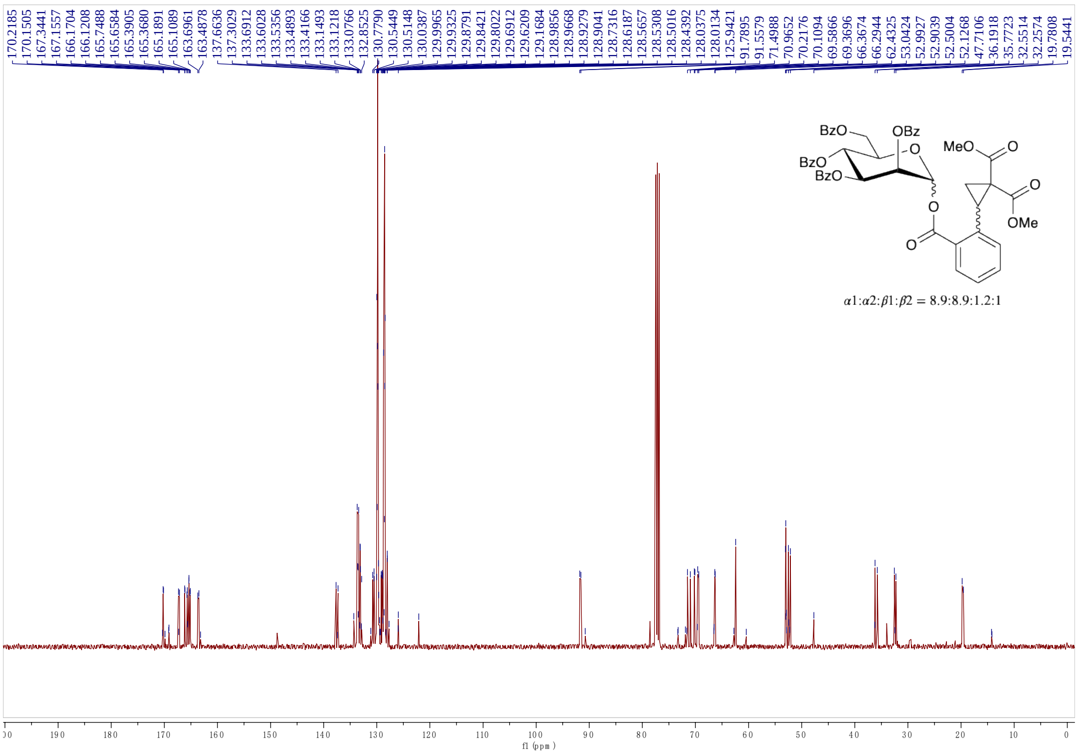

Supplementary Fig. 76 | ^{13}C NMR spectrum of 1c (100 MHz, 25 °C, $CDCl_3$).

^{1}H and ^{13}C spectra for **1d**.

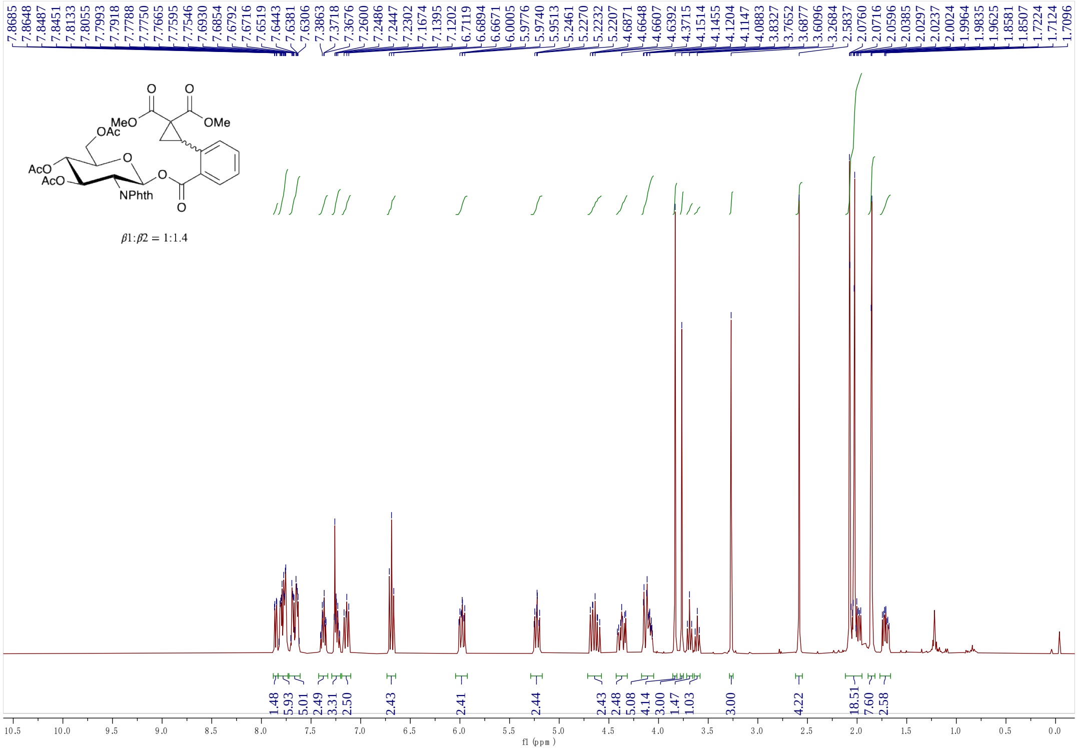

Supplementary Fig. 77 | ^{1}H NMR spectrum of 1d (400 MHz, 25 °C, $CDCl_3$).

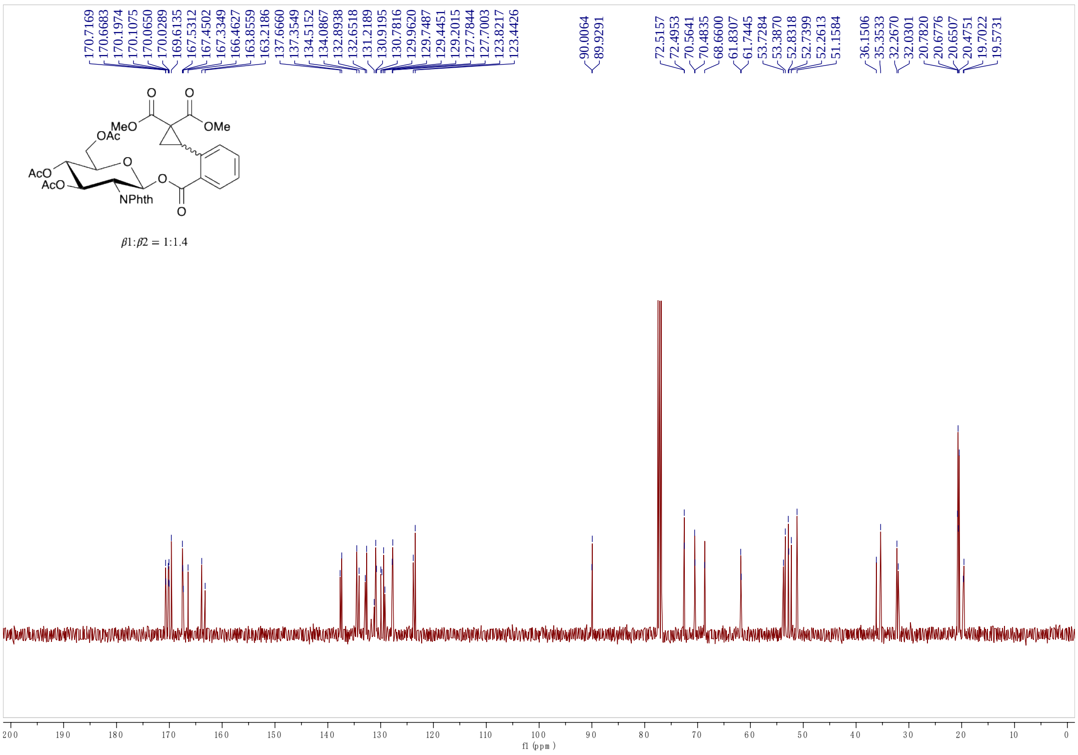

Supplementary Fig. 78 | ^{13}C NMR spectrum of 1d (100 MHz, 25 °C, $CDCl_3$).

^{1}H and ^{13}C spectra for **1e**.

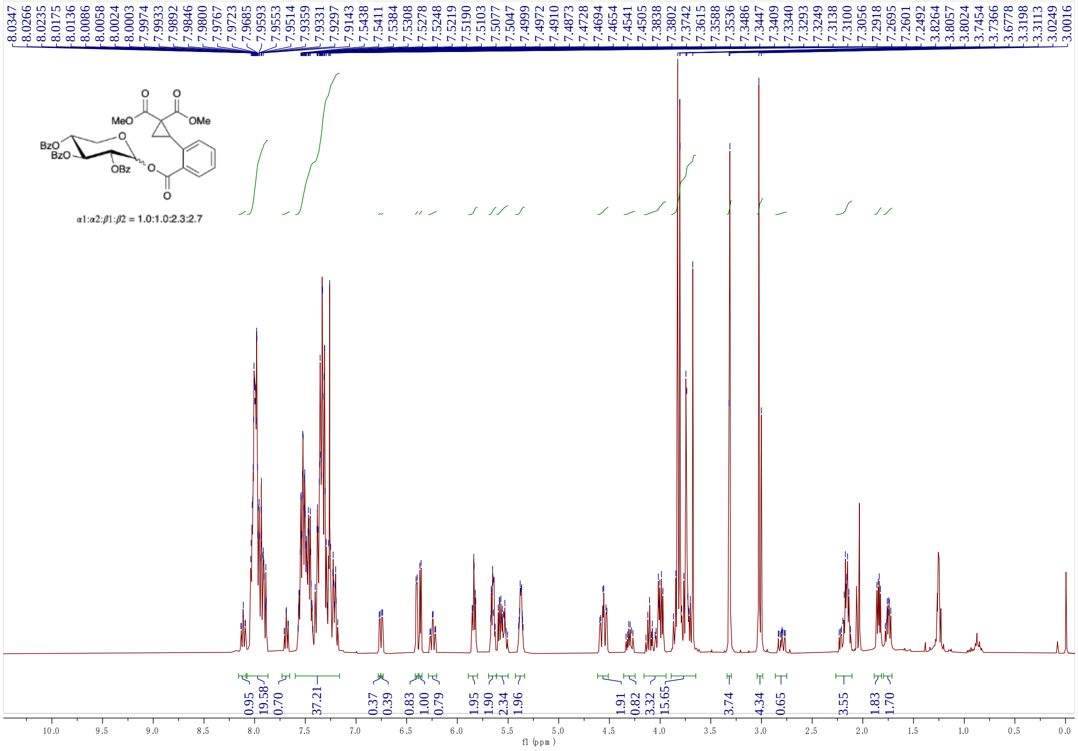

Supplementary Fig. 79 | ^{1}H NMR spectrum of 1e (400 MHz, 25 °C, $CDCl_3$).

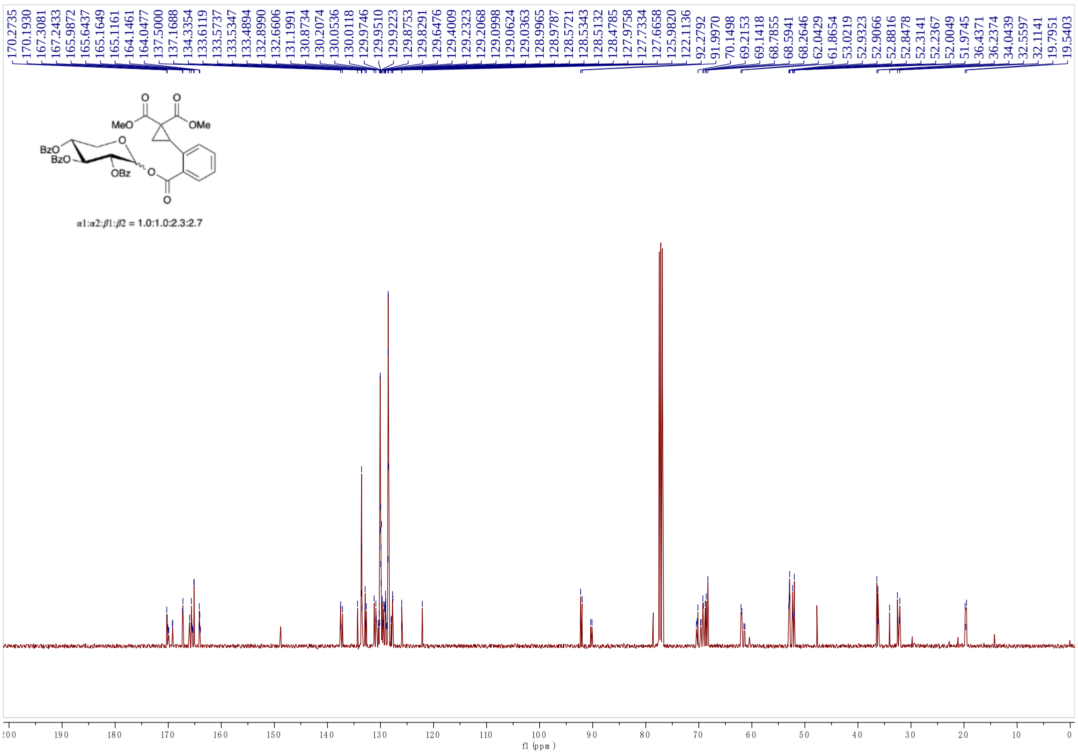

Supplementary Fig. 80 | ^{13}C NMR spectrum of 1e (100 MHz, 25 °C, $CDCl_3$).

^{1}H and ^{13}C spectra for **1f**.

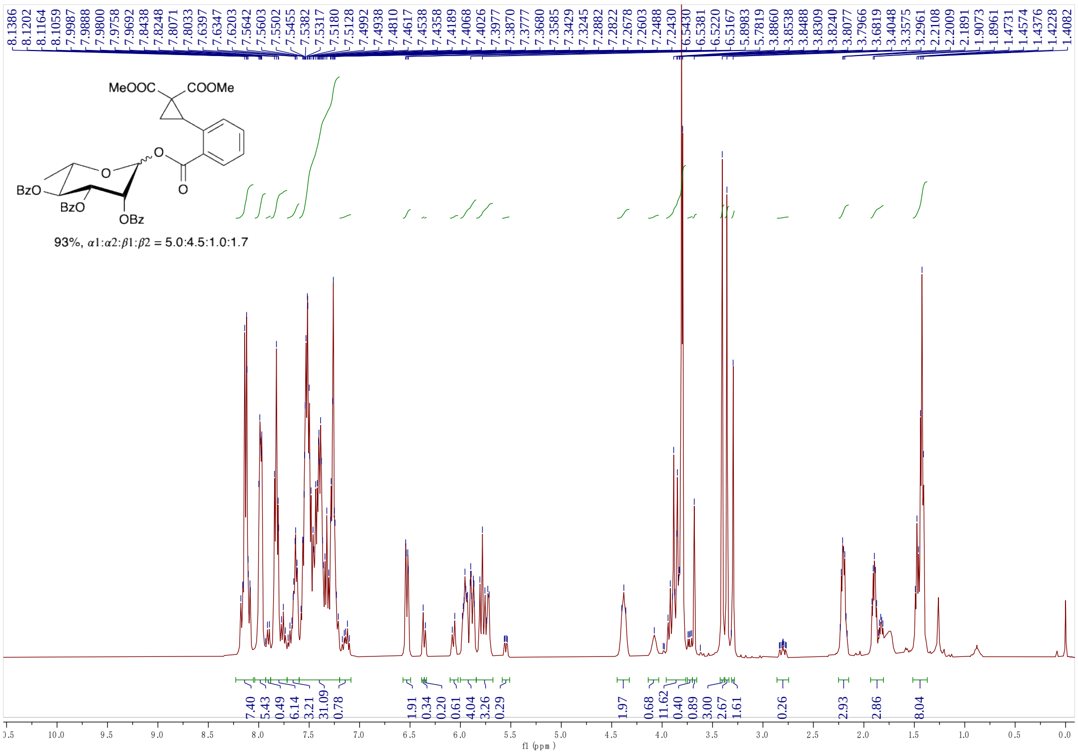

Supplementary Fig. 81 | ^{1}H NMR spectrum of 1f (400 MHz, 25 °C, $CDCl_3$).

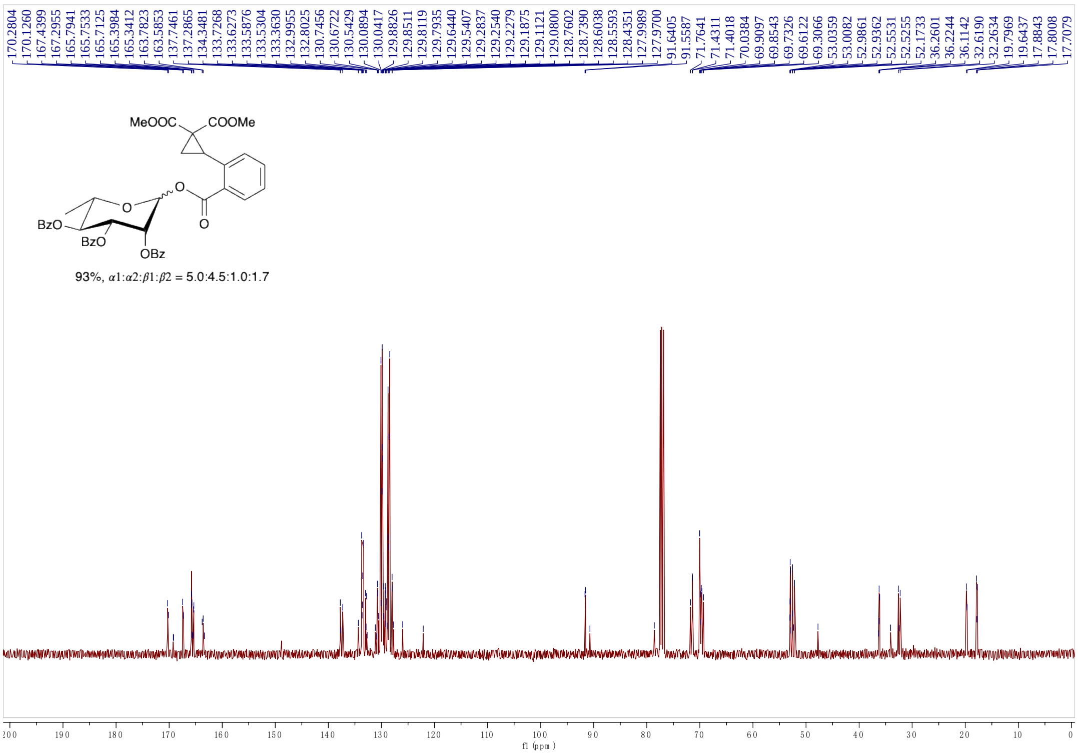

Supplementary Fig. 82 | ^{13}C NMR spectrum of 1f (100 MHz, 25 °C, $CDCl_3$).

^{1}H and ^{13}C spectra for **1g**.

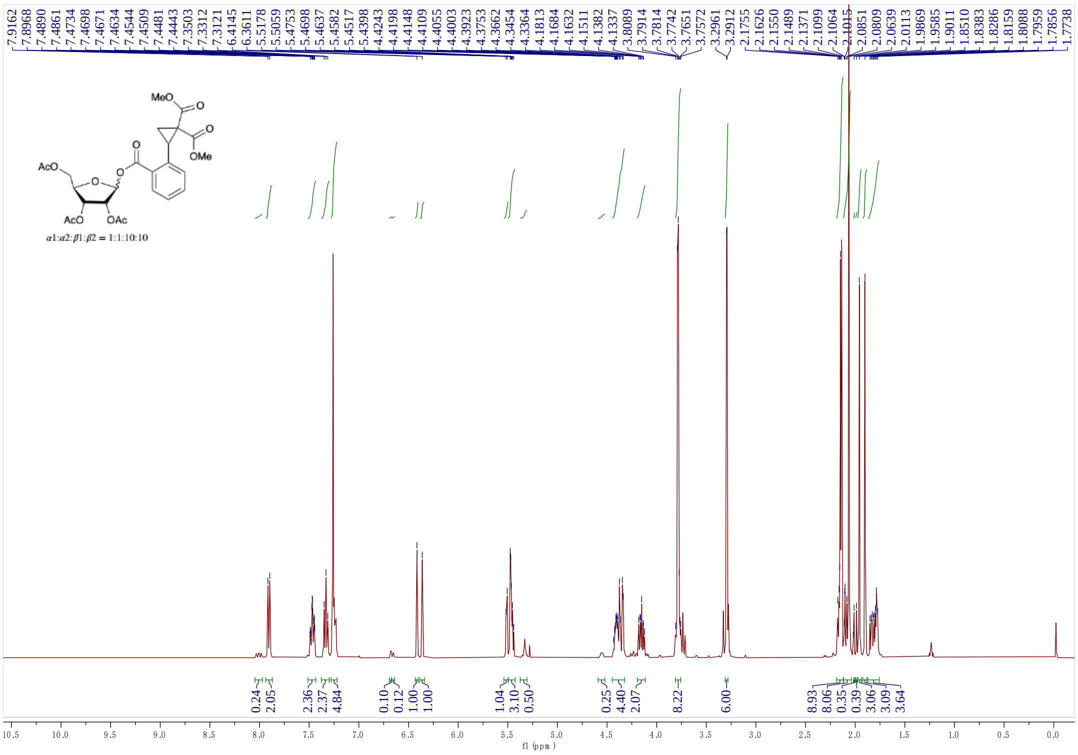

Supplementary Fig. 83 | ^{1}H NMR spectrum of 1g (400 MHz, 25 °C, $CDCl_3$).

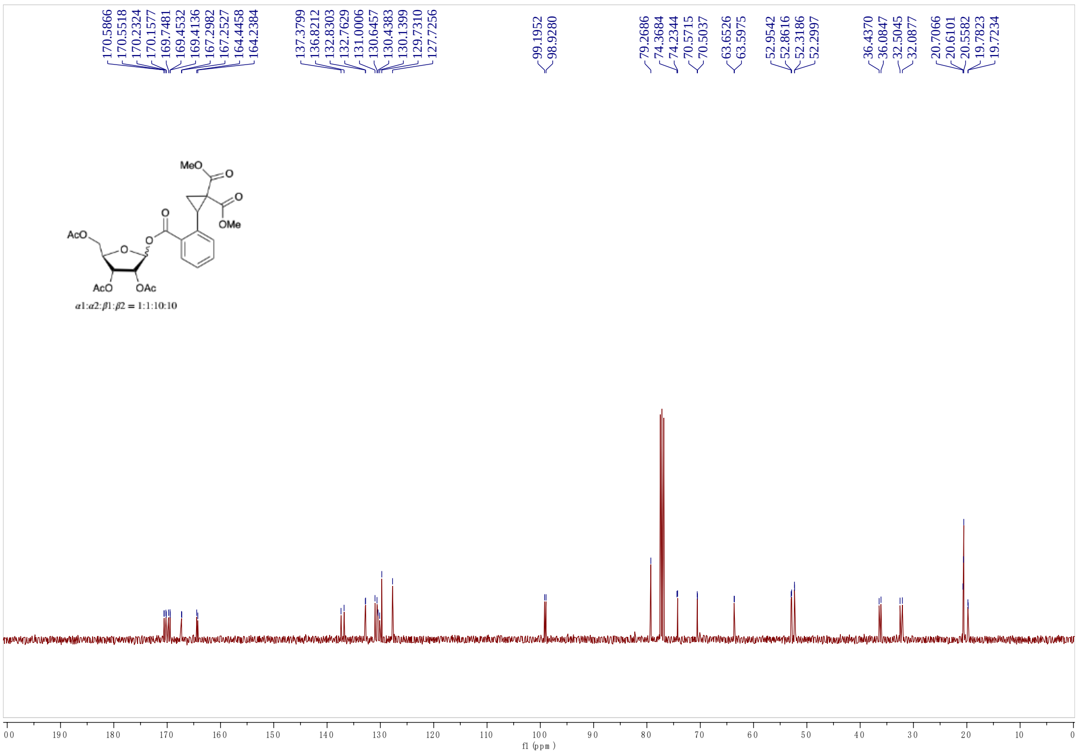

Supplementary Fig. 84 | ^{13}C NMR spectrum of 1g (100 MHz, 25 °C, $CDCl_3$).

^{1}H and ^{13}C spectra for **1h**.

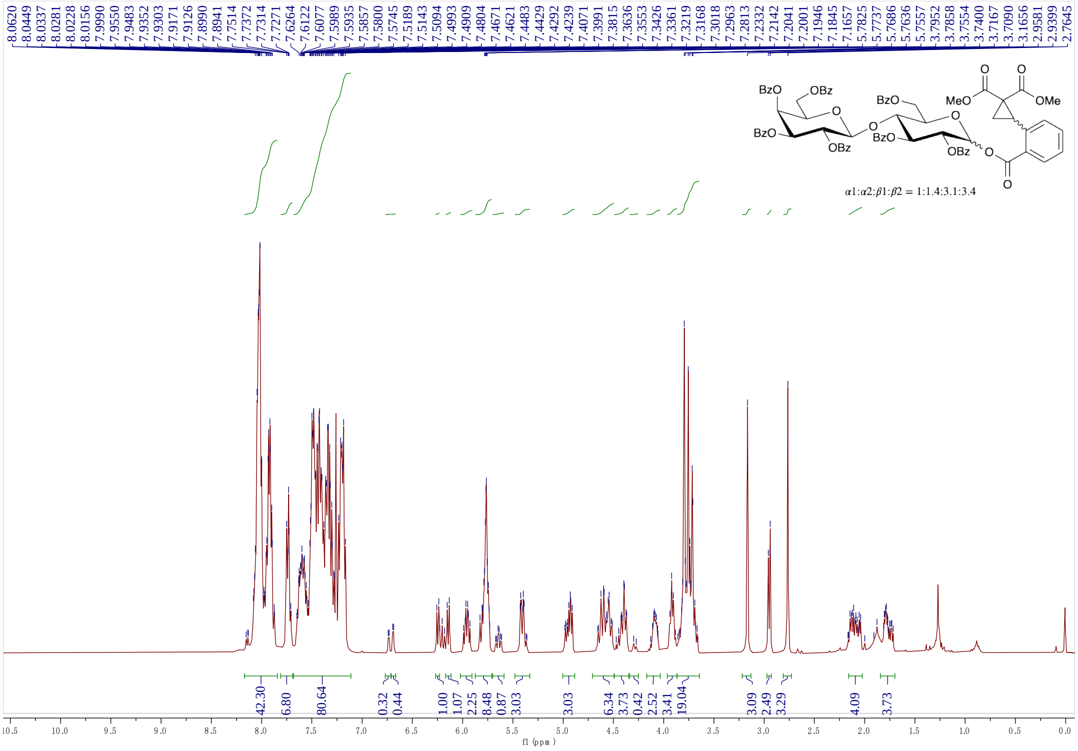

Supplementary Fig. 85 | ^{1}H NMR spectrum of 1h (400 MHz, 25 ^{o}C, $CDCl_3$).

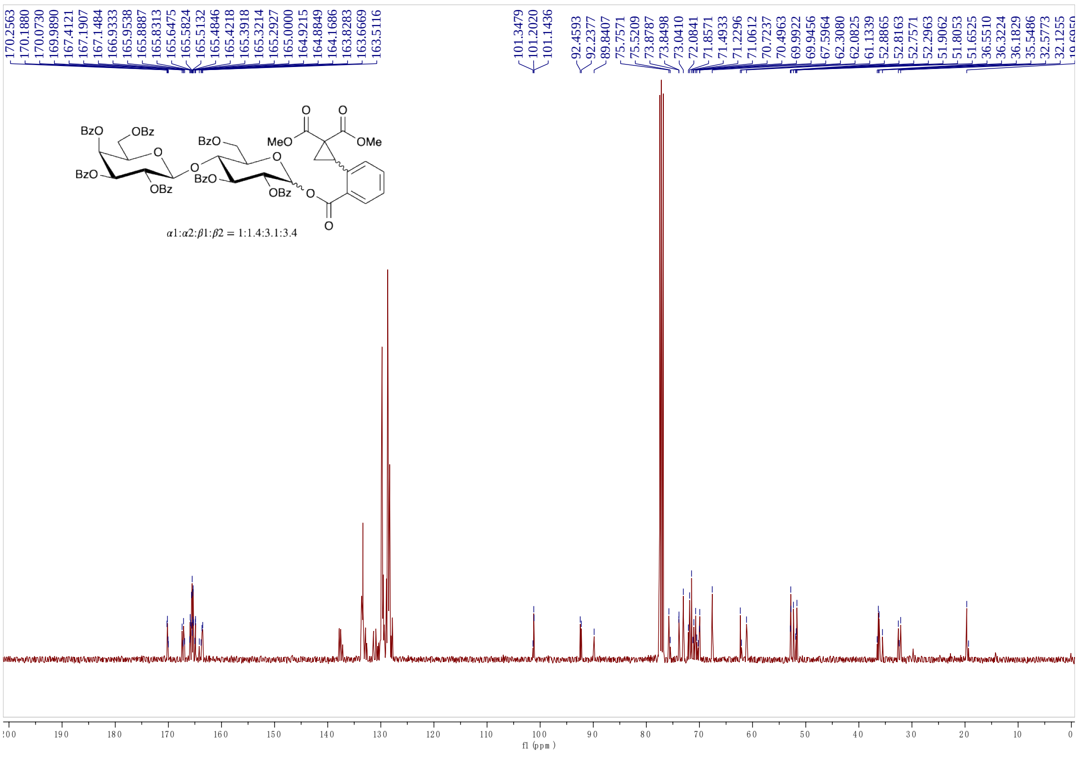

Supplementary Fig. 86 | ^{13}C NMR spectrum of 1h (100 MHz, 25 ^{o}C, $CDCl_3$).

^{1}H and ^{13}C spectra for **4**.

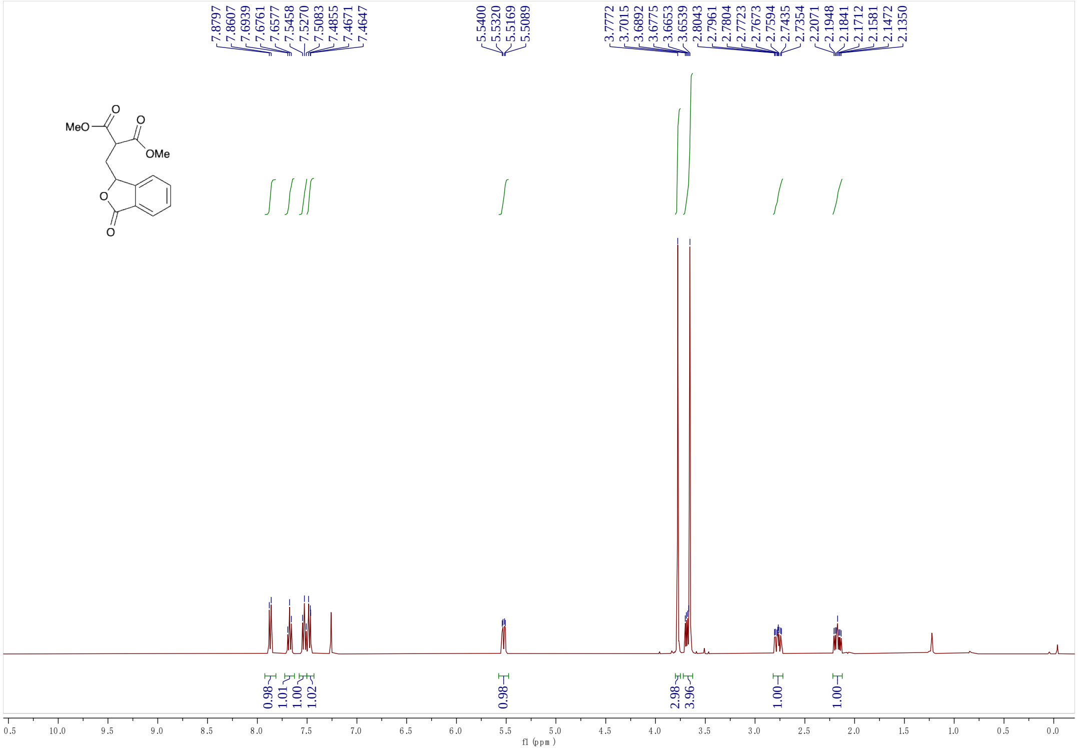

Supplementary Fig. 87 | ^{1}H NMR spectrum of 4 (400 MHz, 25 ºC, $CDCl_3$).

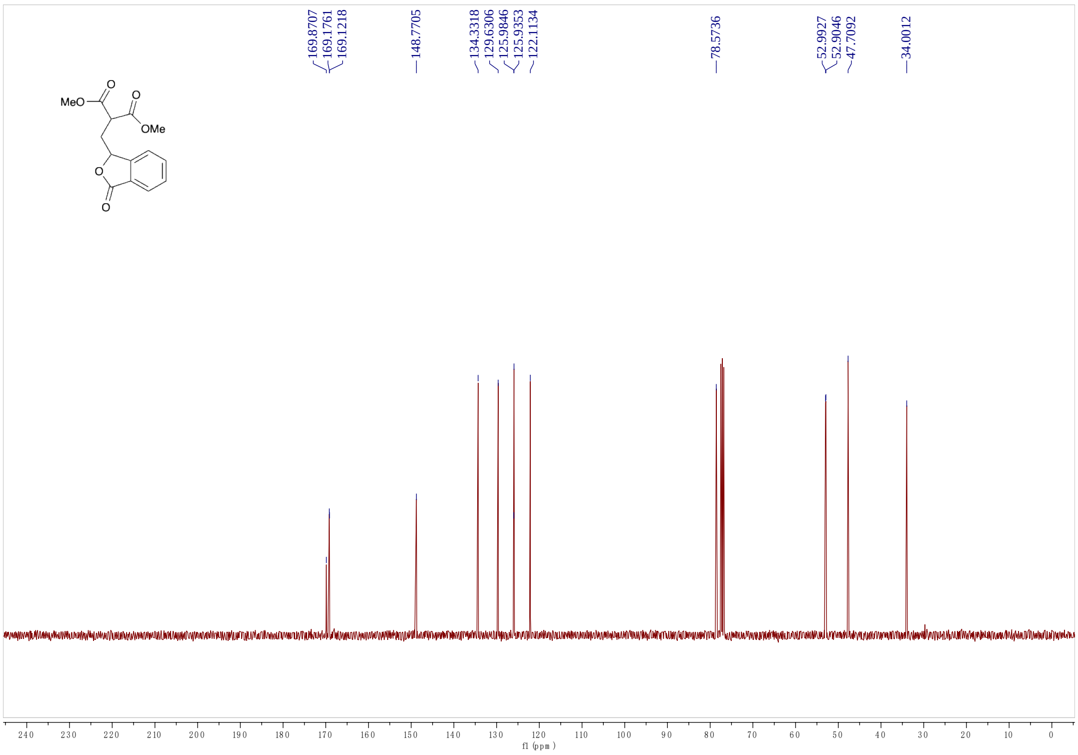

Supplementary Fig. 88 | ^{13}C NMR spectrum of 4 (100 MHz, 25 ºC, $CDCl_3$).

^{1}H spectrum for **3a**.

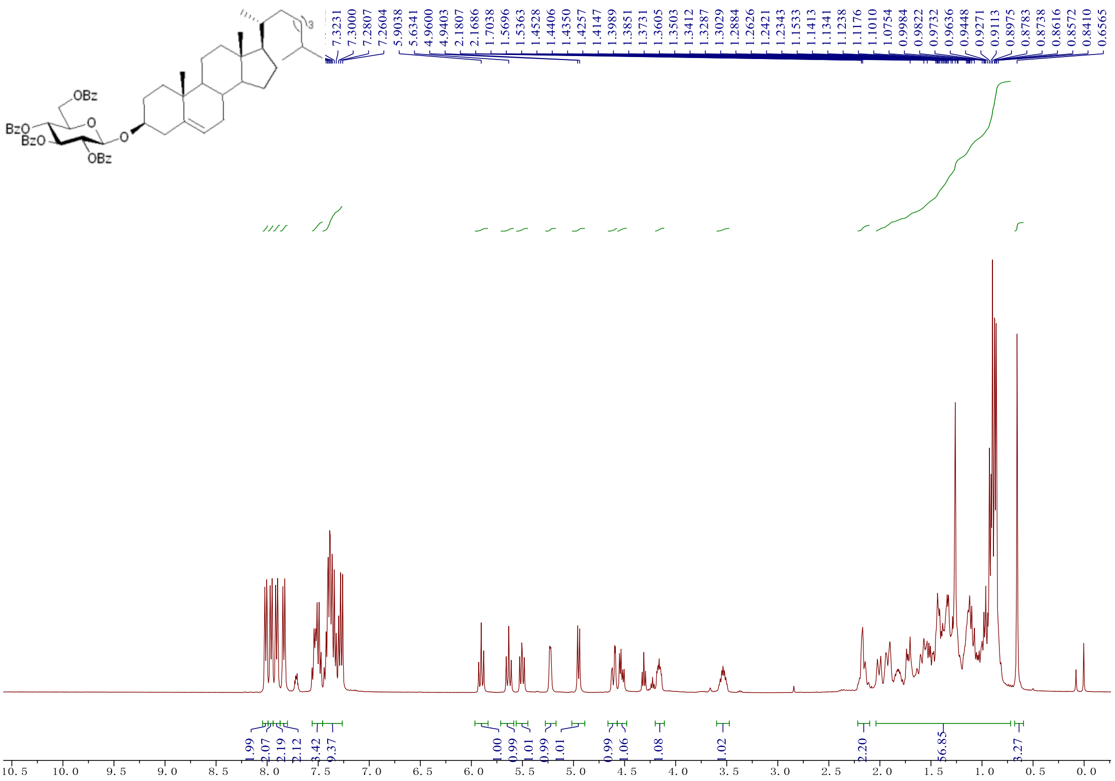

Supplementary Fig. 89 | ^{1}H NMR spectrum of 3a (400 MHz, 25 °C, $CDCl_3$).

^{1}H spectrum for **3b**.

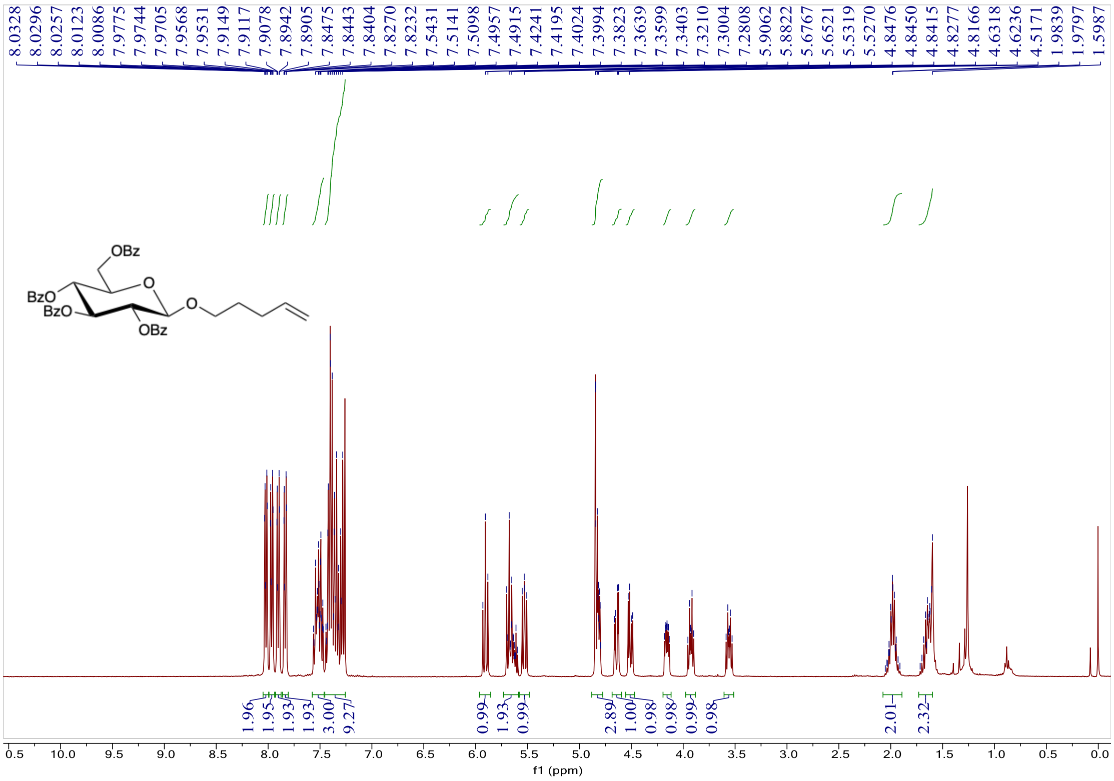

Supplementary Fig. 90 | ^{1}H NMR spectrum of 3b (400 MHz, 25 °C, $CDCl_3$).

^{1}H spectrum for **3c**.

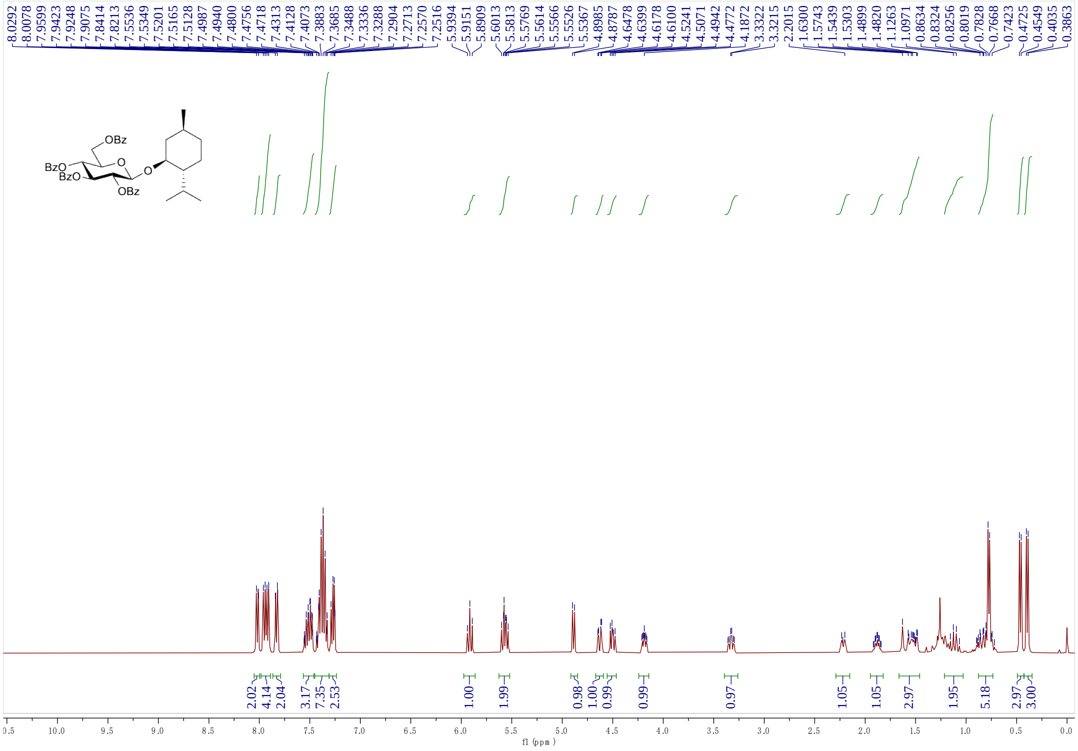

Supplementary Fig. 91 | ^{1}H NMR spectrum of 3c (400 MHz, 25 °C, $CDCl_3$).

^{1}H spectrum for **3d**.

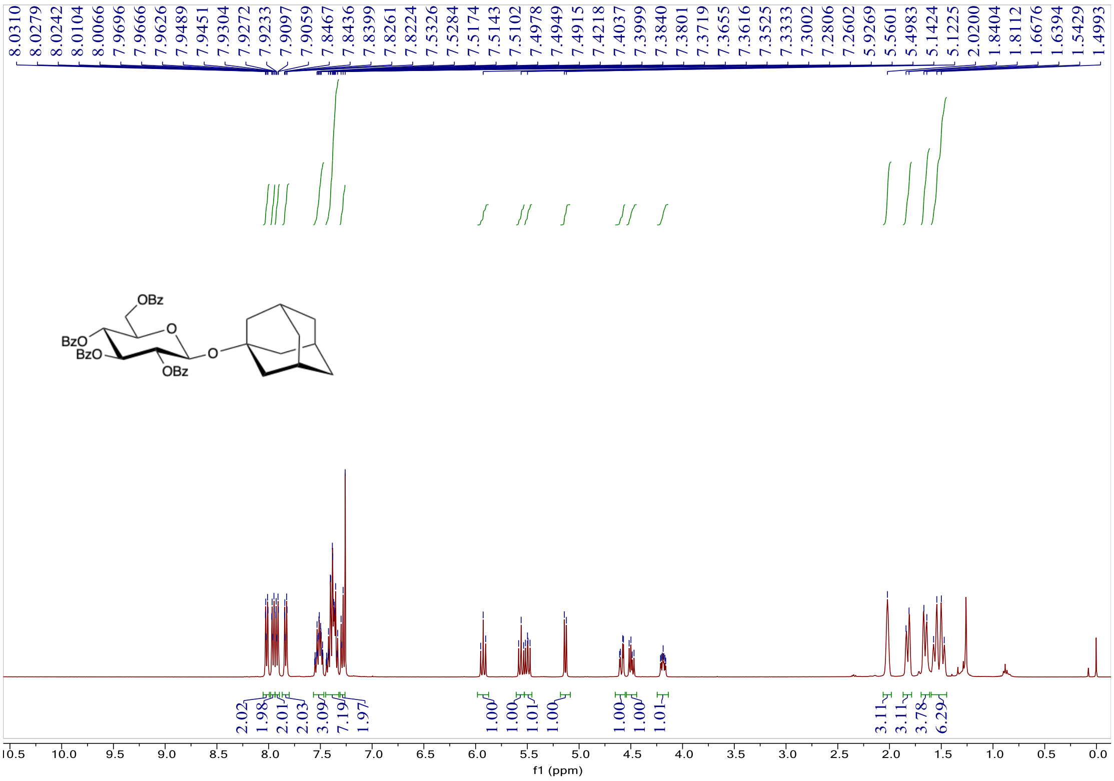

Supplementary Fig. 92 | ^{1}H NMR spectrum of 3d (400 MHz, 25 °C, $CDCl_3$).

^{1}H spectrum for **3e**.

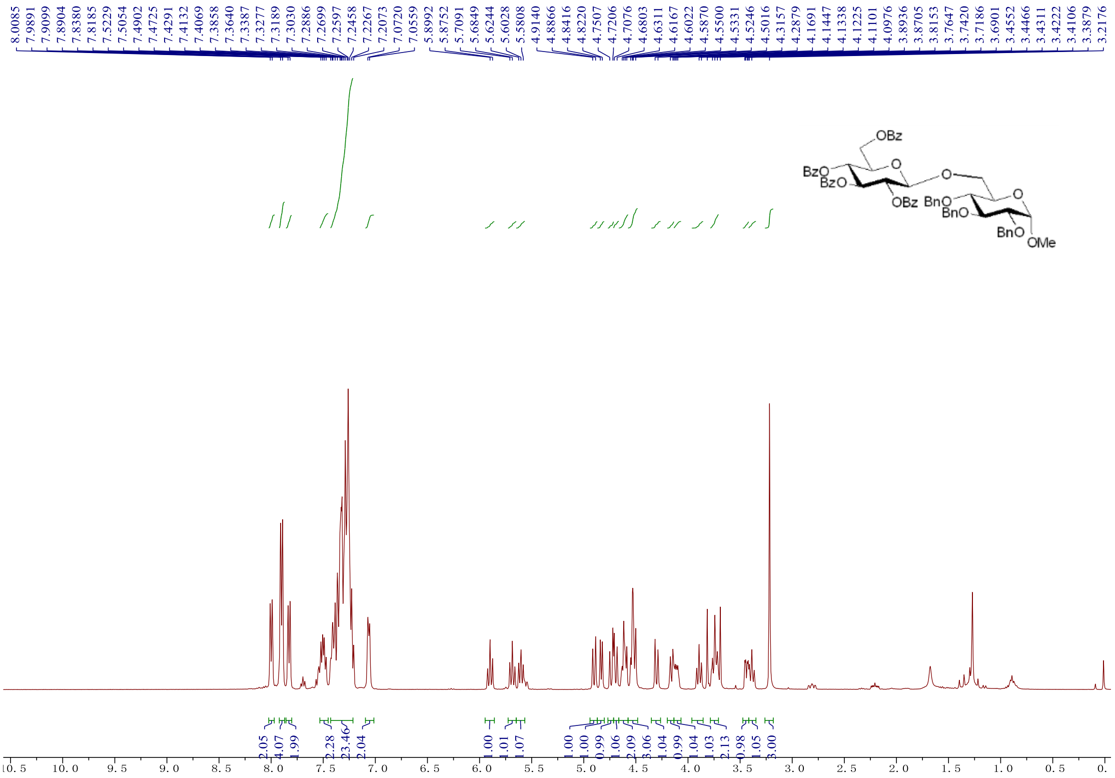

Supplementary Fig. 93 | ^{1}H NMR spectrum of 3e (400 MHz, 25 °C, $CDCl_3$).

^{1}H spectrum for **3f**.

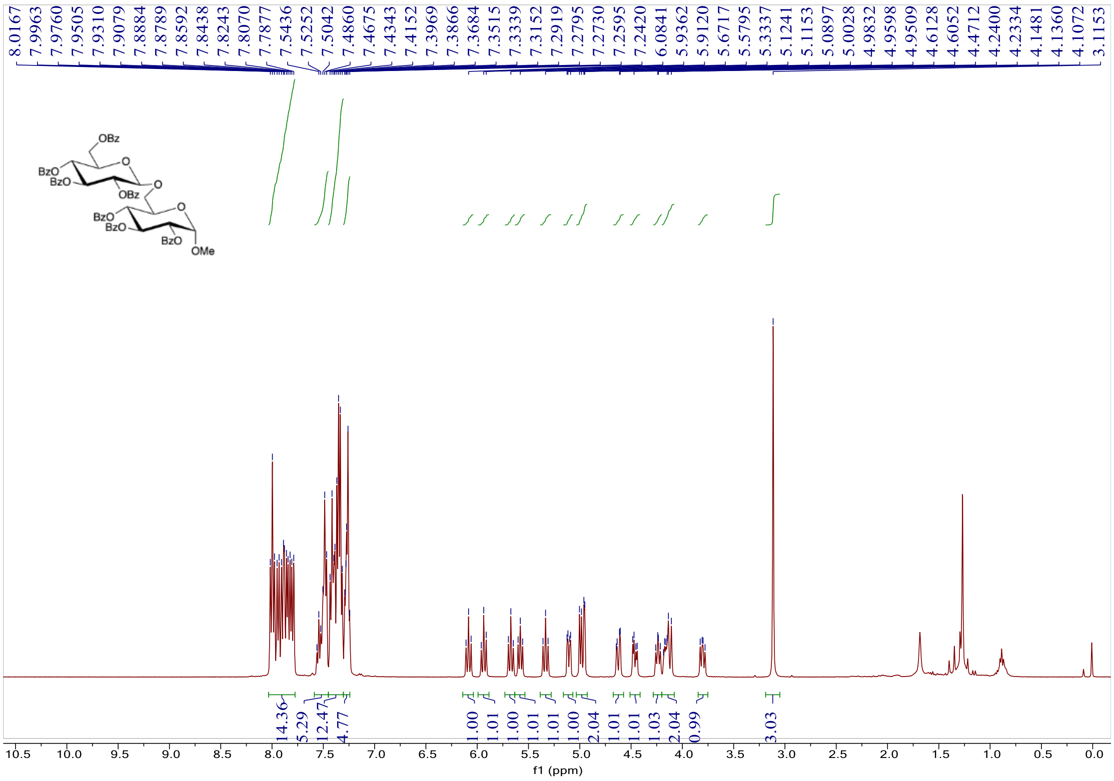

Supplementary Fig. 94 | ^{1}H NMR spectrum of 3f (400 MHz, 25 °C, $CDCl_3$).

^{1}H spectrum for **3g**.

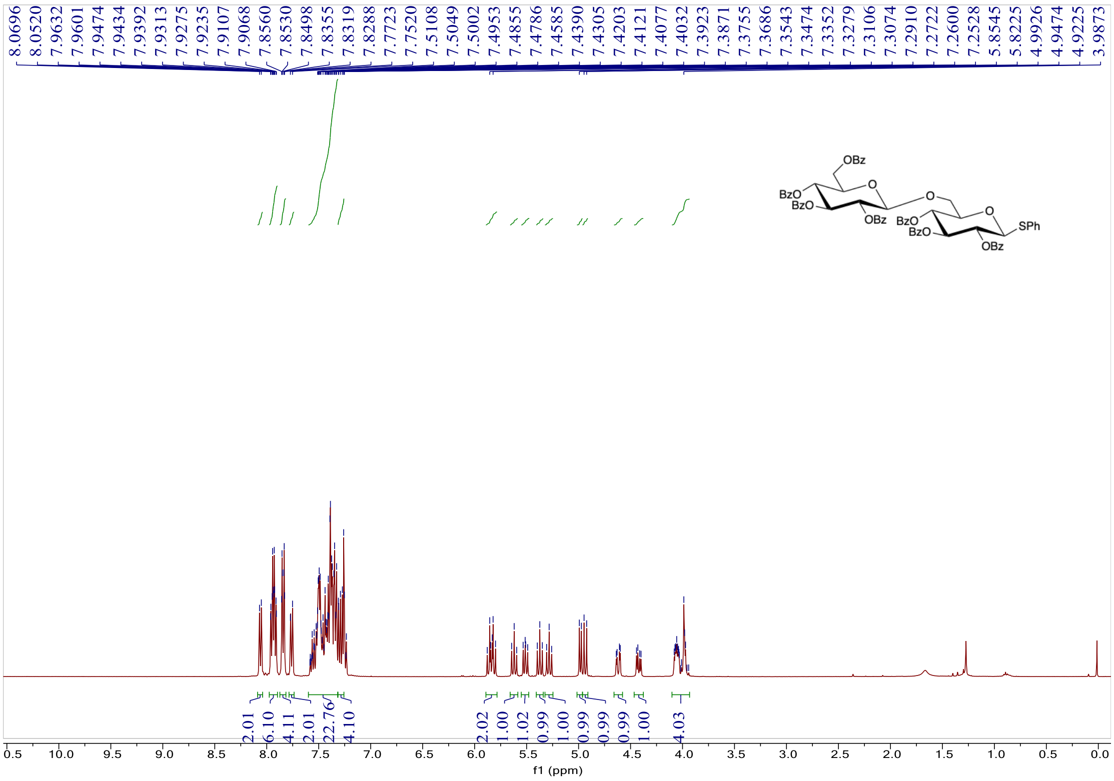

Supplementary Fig. 95 | ^{1}H NMR spectrum of 3g (400 MHz, 25 °C, $CDCl_3$).

^{1}H spectrum for **3h**.

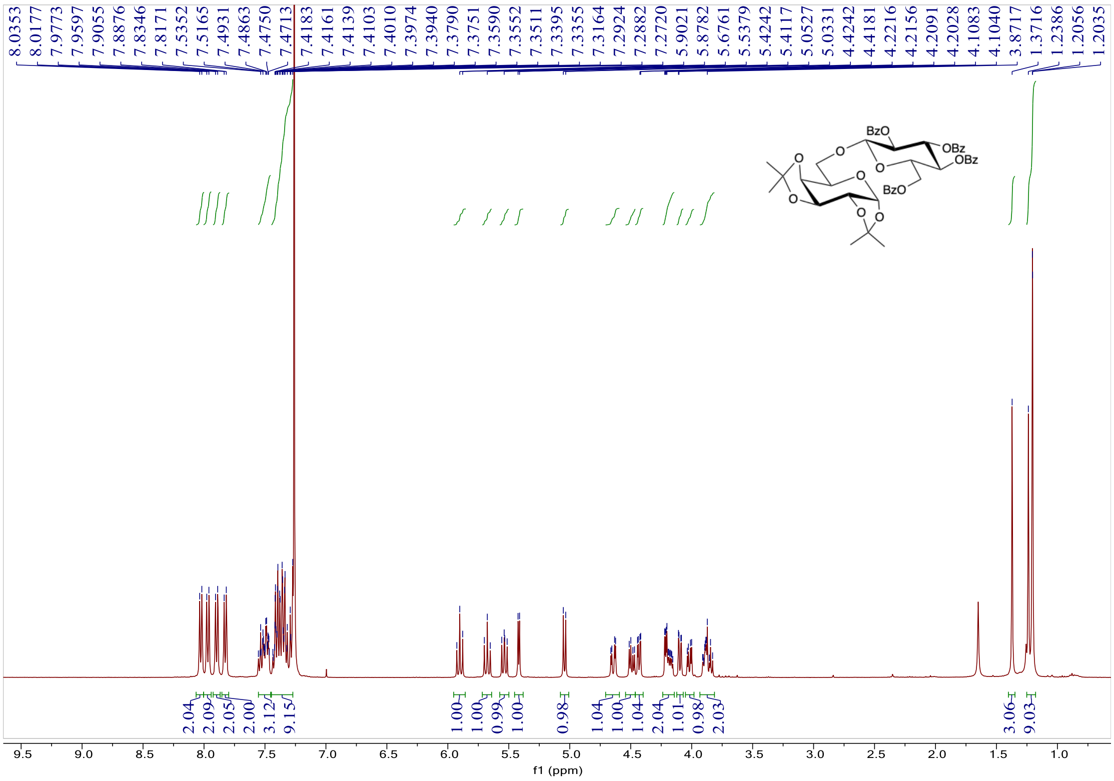

Supplementary Fig. 96 | ^{1}H NMR spectrum of 3h (400 MHz, 25 ^{o}C, $CDCl_3$).

^{1}H spectrum for **3i**.

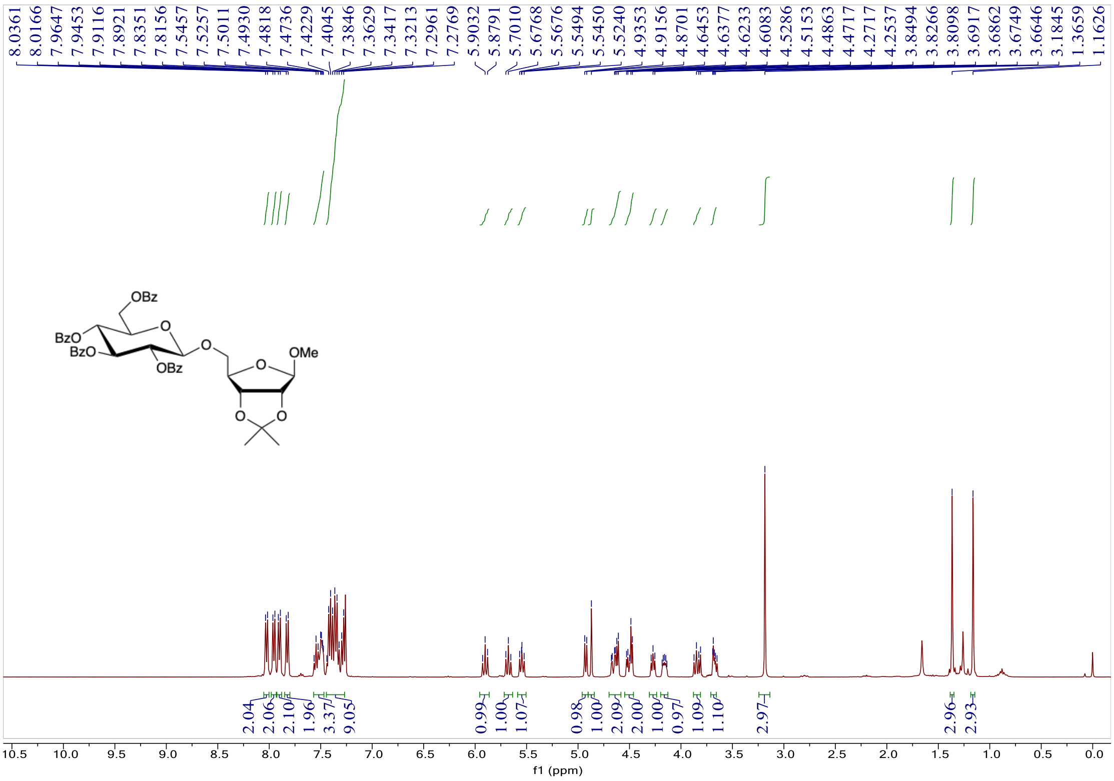

Supplementary Fig. 97 | ^{1}H NMR spectrum of 3i (400 MHz, 25 °C, $CDCl_3$).

^{1}H spectrum for **3j**.

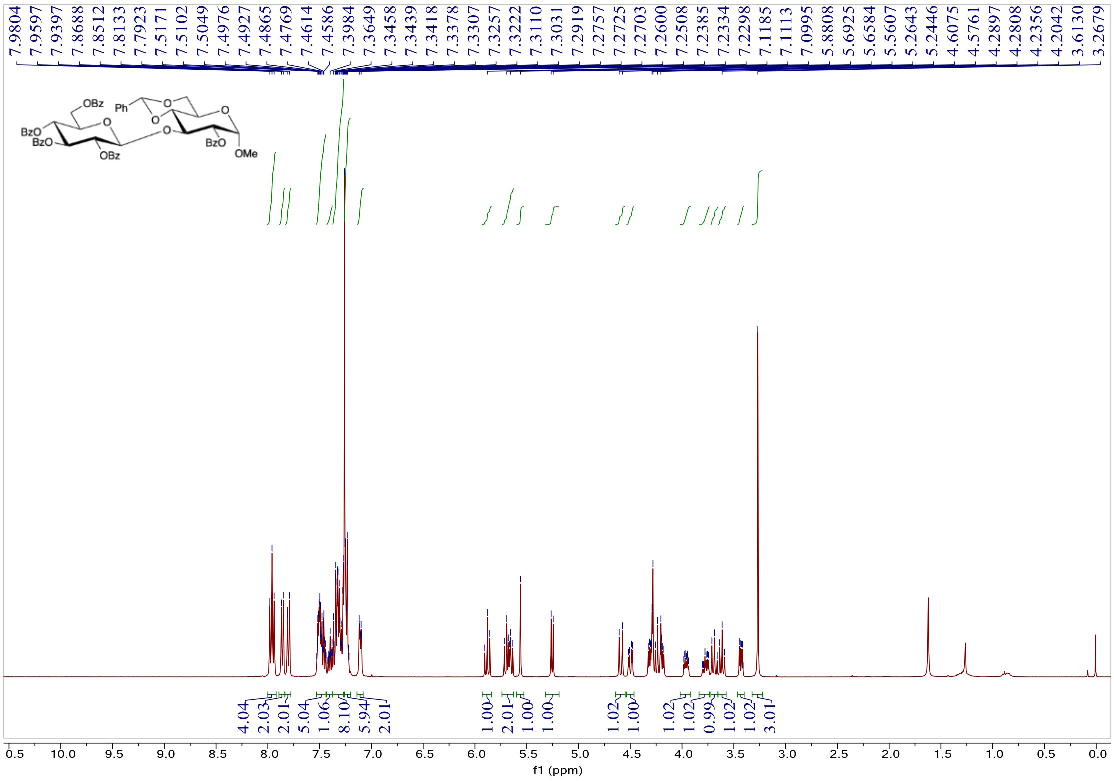

Supplementary Fig. 98 | ^{1}H NMR spectrum of 3j (400 MHz, 25 °C, $CDCl_3$).

^{1}H spectrum for **3k**.

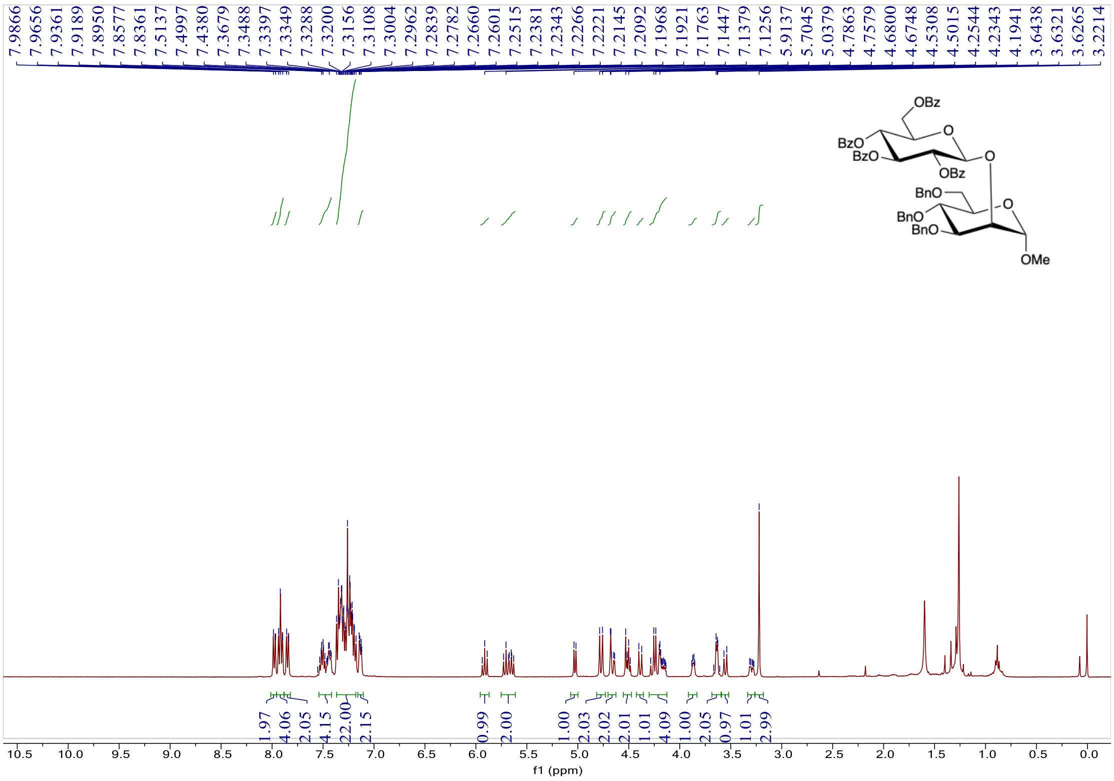

Supplementary Fig. 99 | ^{1}H NMR spectrum of 3k (400 MHz, 25 °C, $CDCl_3$).

^{1}H spectrum for **3l**.

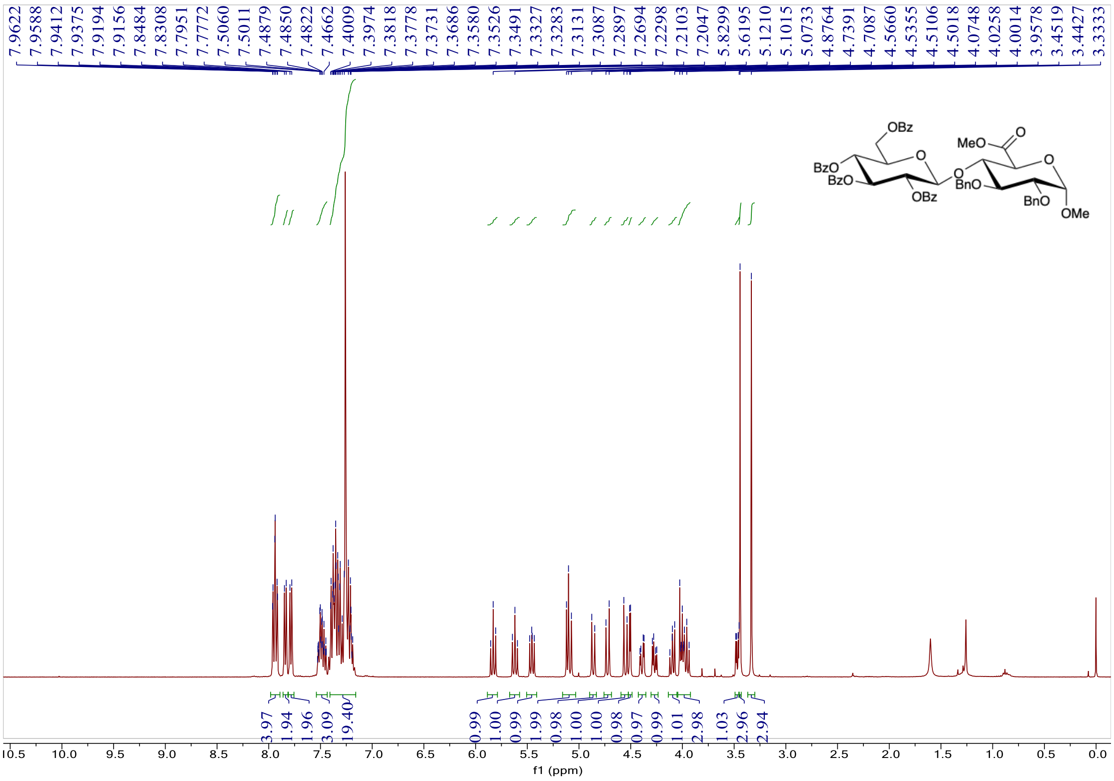

Supplementary Fig. 100 | ^{1}H NMR spectrum of 3l (400 MHz, 25 °C, $CDCl_3$).

^{1}H spectrum for **3m**.

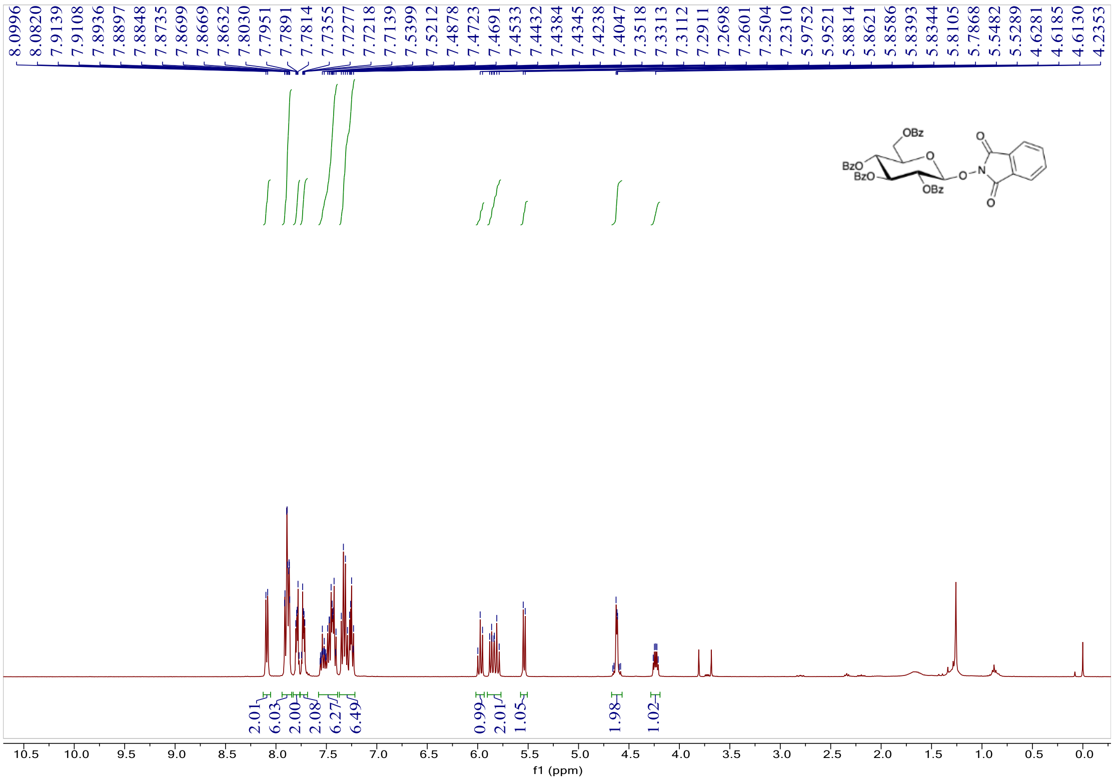

Supplementary Fig. 101 | ^{1}H NMR spectrum of 3m (400 MHz, 25 °C, $CDCl_3$).

^{1}H and ^{13}C spectra for **3n**.

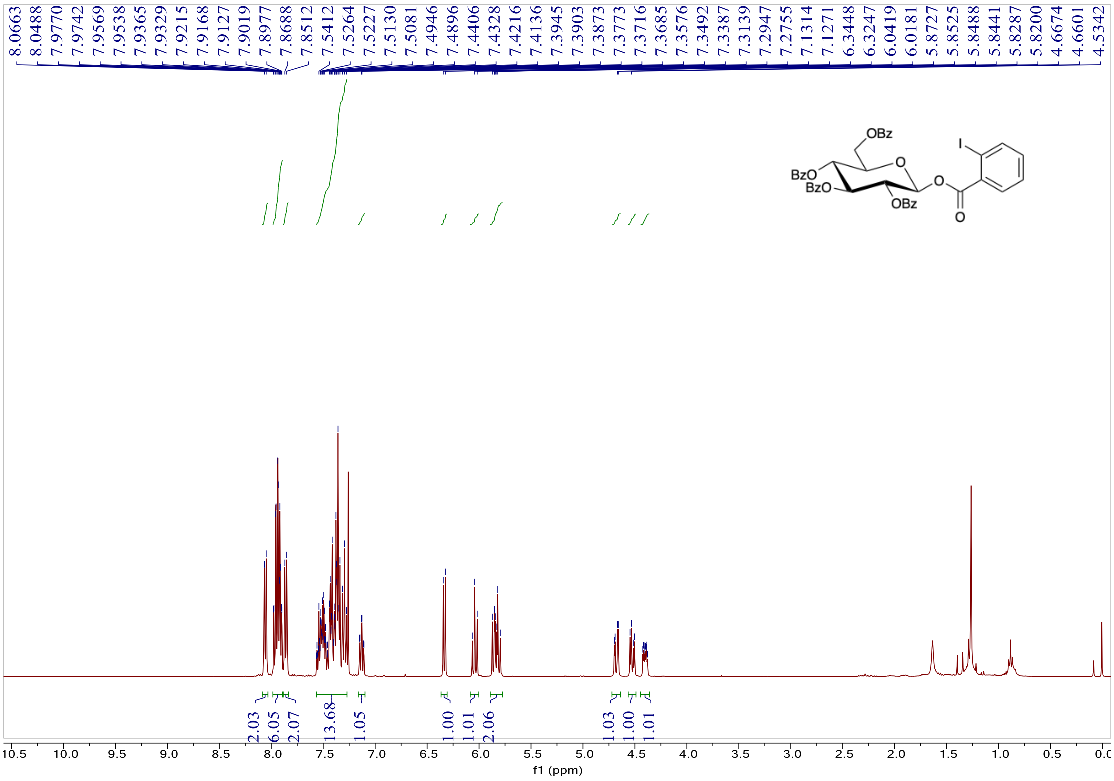

Supplementary Fig. 102 | ^{1}H NMR spectrum of 3n (400 MHz, 25 °C, $CDCl_3$).

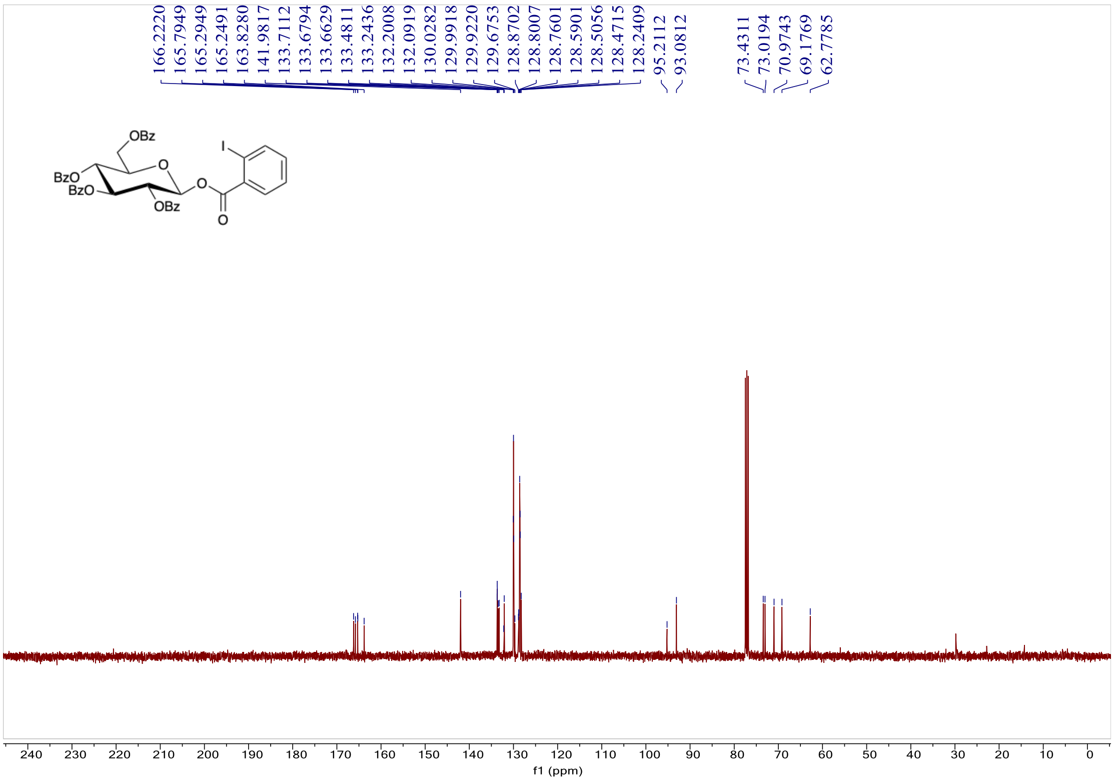

Supplementary Fig. 103 | ^{13}C NMR spectrum of 3n (100 MHz, 25 °C, $CDCl_3$).

^{1}H spectrum for **3o**.

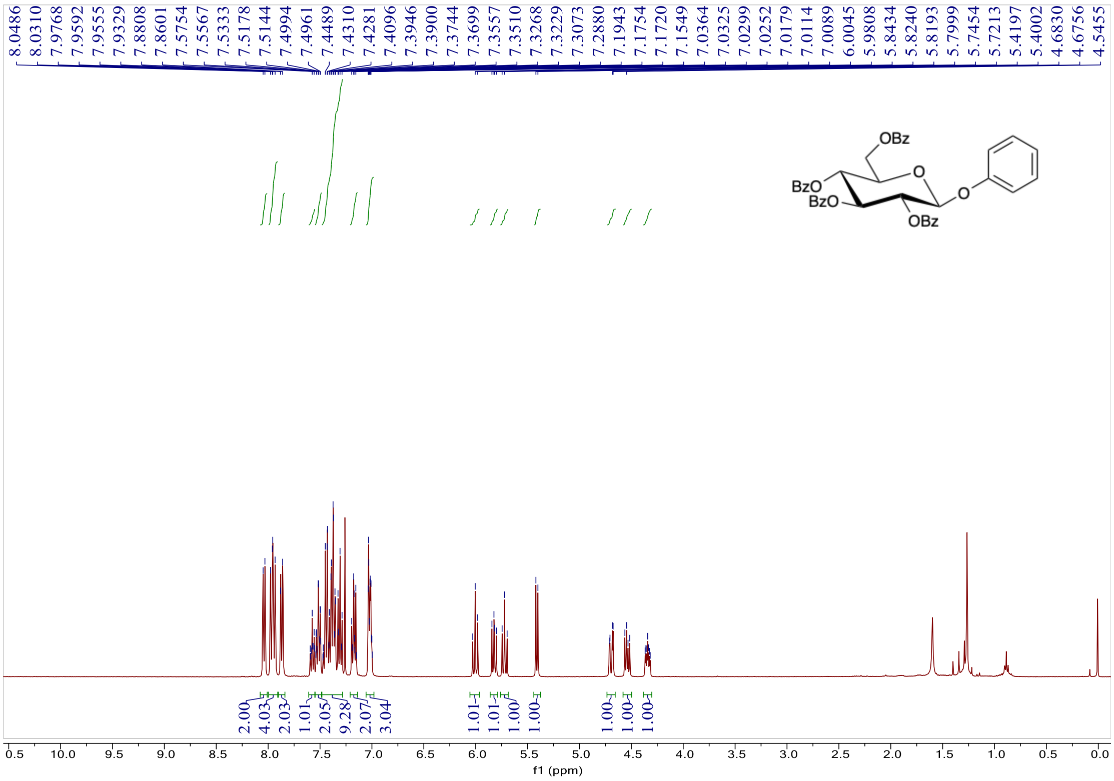

Supplementary Fig. 104 | ^{1}H NMR spectrum of 3o (400 MHz, 25 °C, $CDCl_3$).

^{1}H spectrum for **3p**.

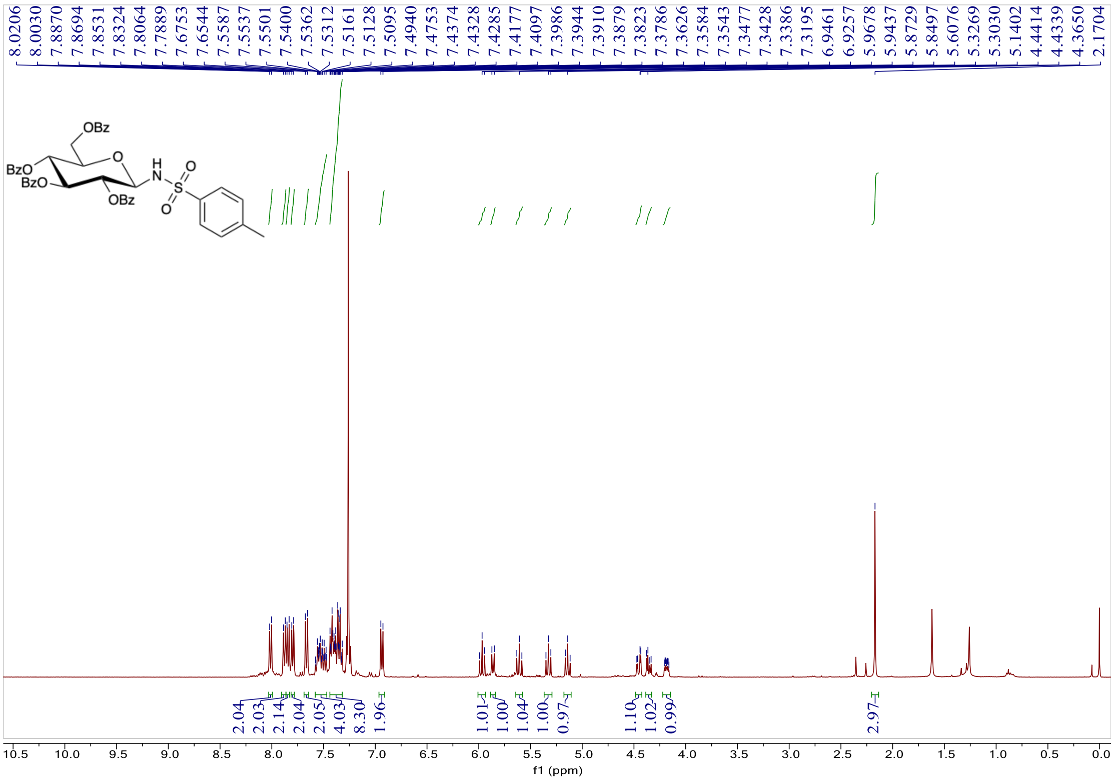

Supplementary Fig. 105 | ^{1}H NMR spectrum of 3p (400 MHz, 25 °C, $CDCl_3$).

^{1}H spectrum for **3q**.

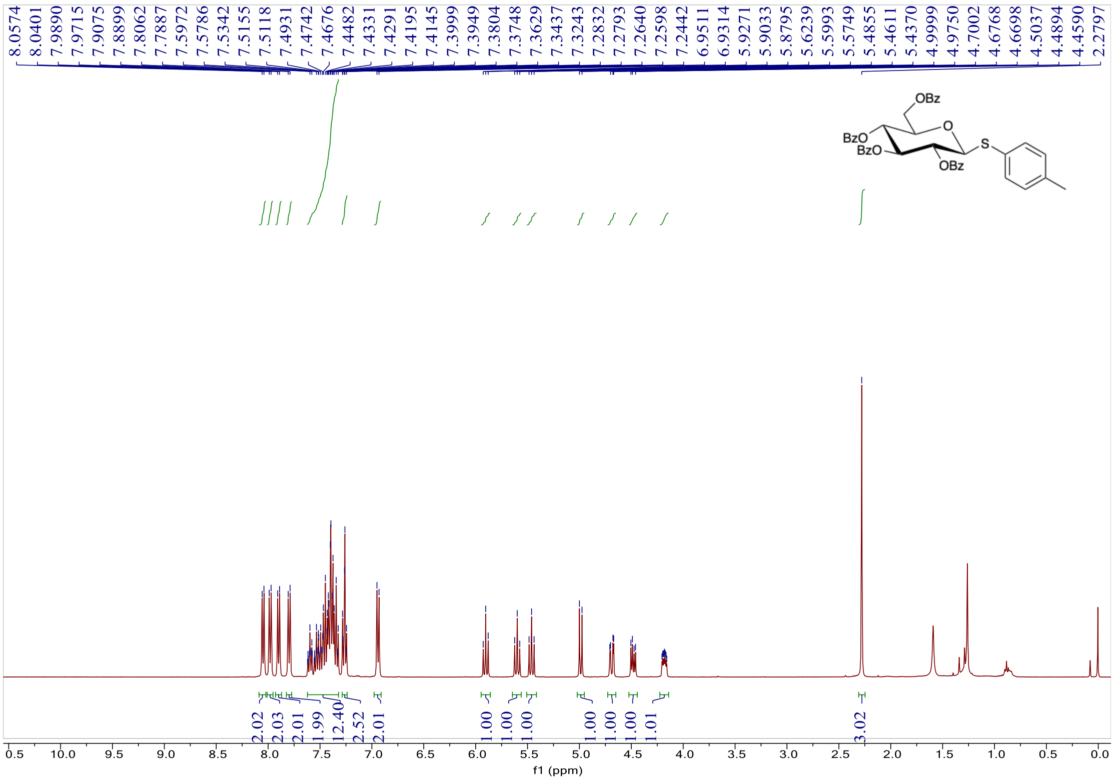

Supplementary Fig. 106 | ^{1}H NMR spectrum of 3q (400 MHz, 25 °C, $CDCl_3$).

^{1}H and ^{13}C spectra for **3r**.

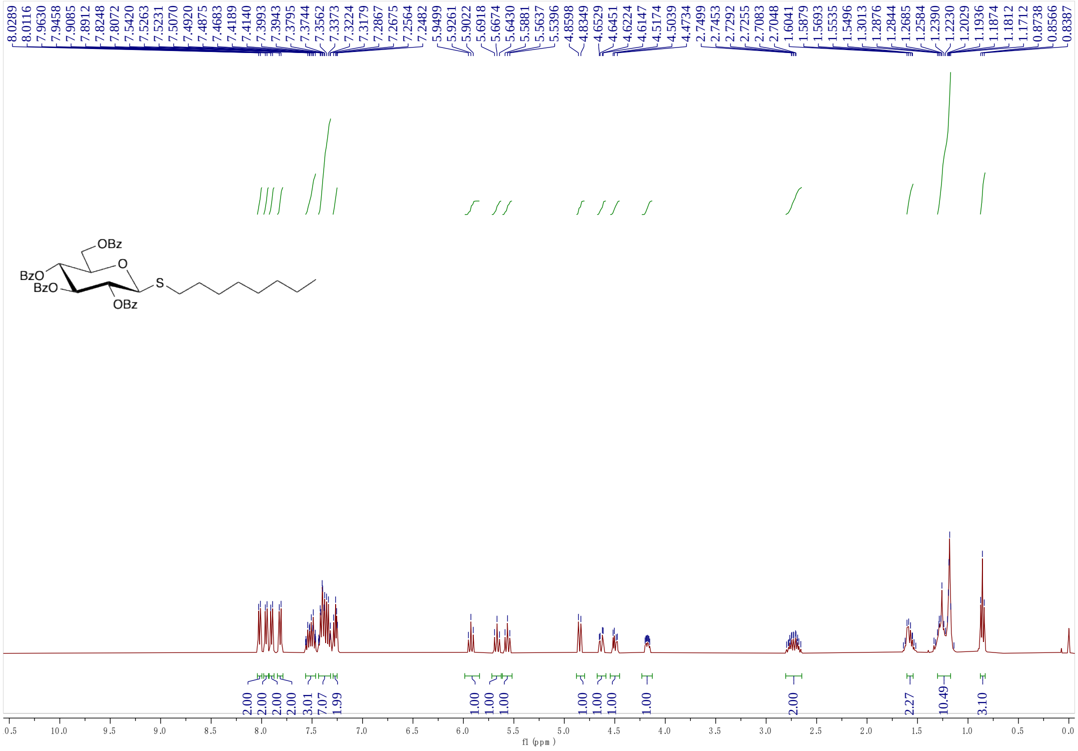

Supplementary Fig. 107 | ^{1}H NMR spectrum of 3r (400 MHz, 25 °C, $CDCl_3$).

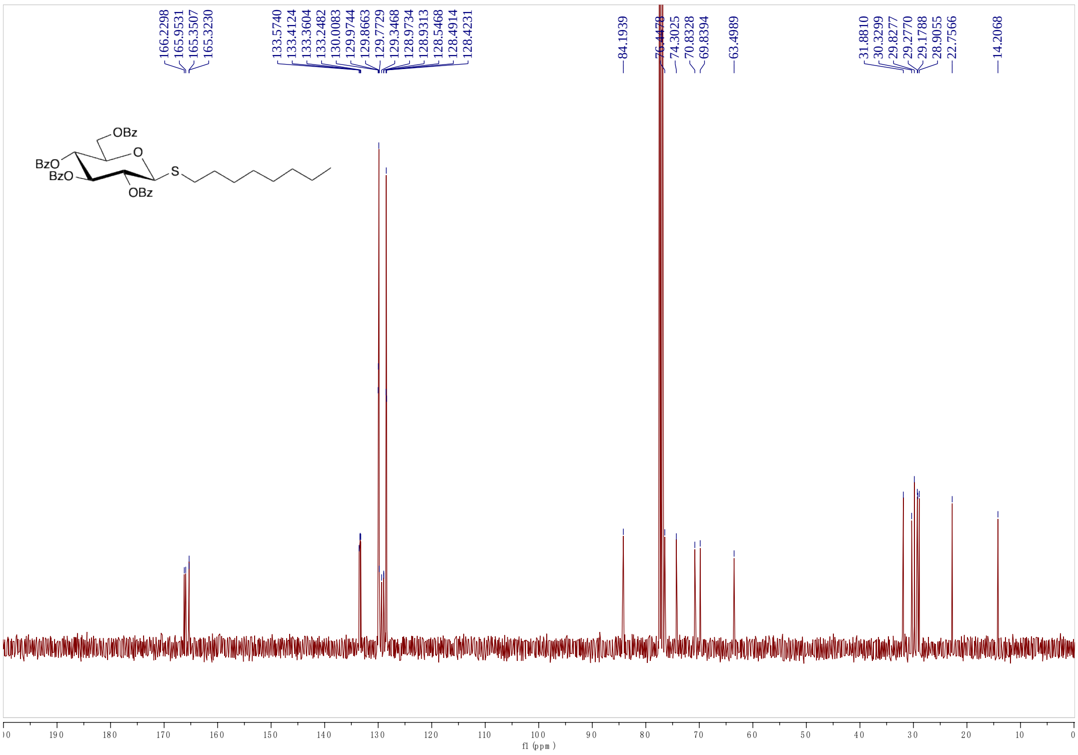

Supplementary Fig. 108 | ^{13}C NMR spectrum of 3r (100 MHz, 25 °C, $CDCl_3$).

^{1}H spectrum for **3ba**.

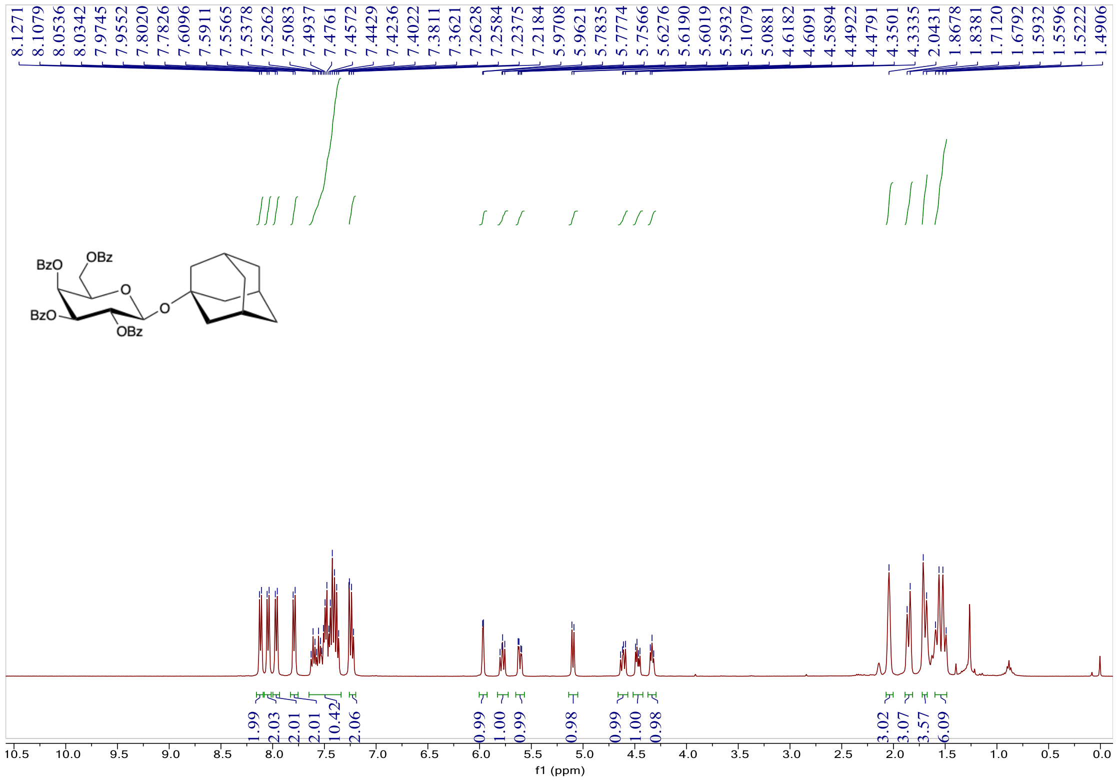

Supplementary Fig. 109 | ^{1}H NMR spectrum of 3ba (400 MHz, 25 ^{o}C, $CDCl_3$).

^{1}H spectrum for **3bb**.

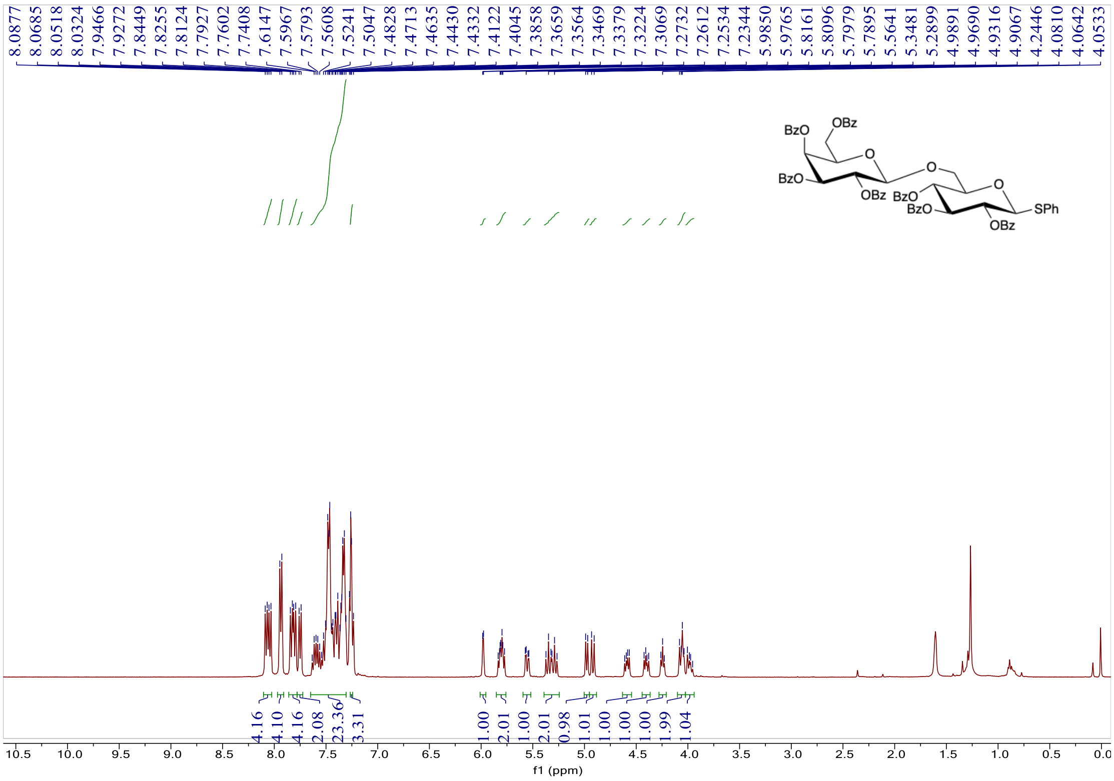

Supplementary Fig. 110 | ^{1}H NMR spectrum of 3bb (400 MHz, 25 °C, $CDCl_3$).

^{1}H and ^{13}C spectra for **3bc**.

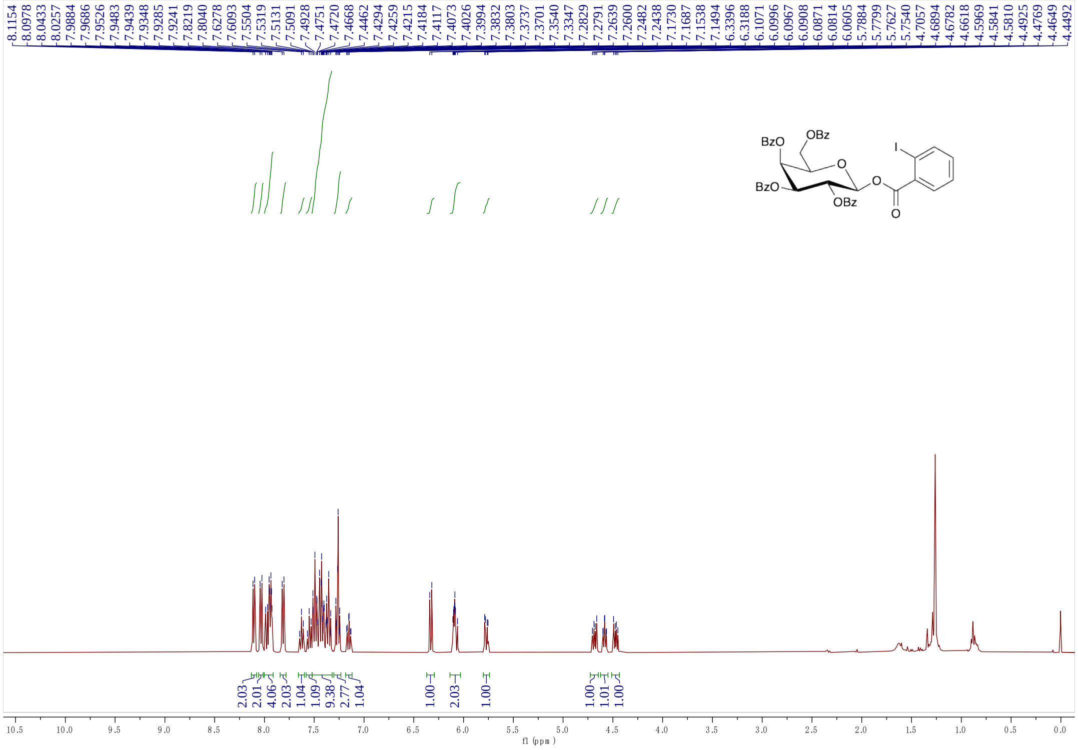

Supplementary Fig. 111 | ^{1}H NMR spectrum of 3bc (400 MHz, 25 °C, $CDCl_3$).

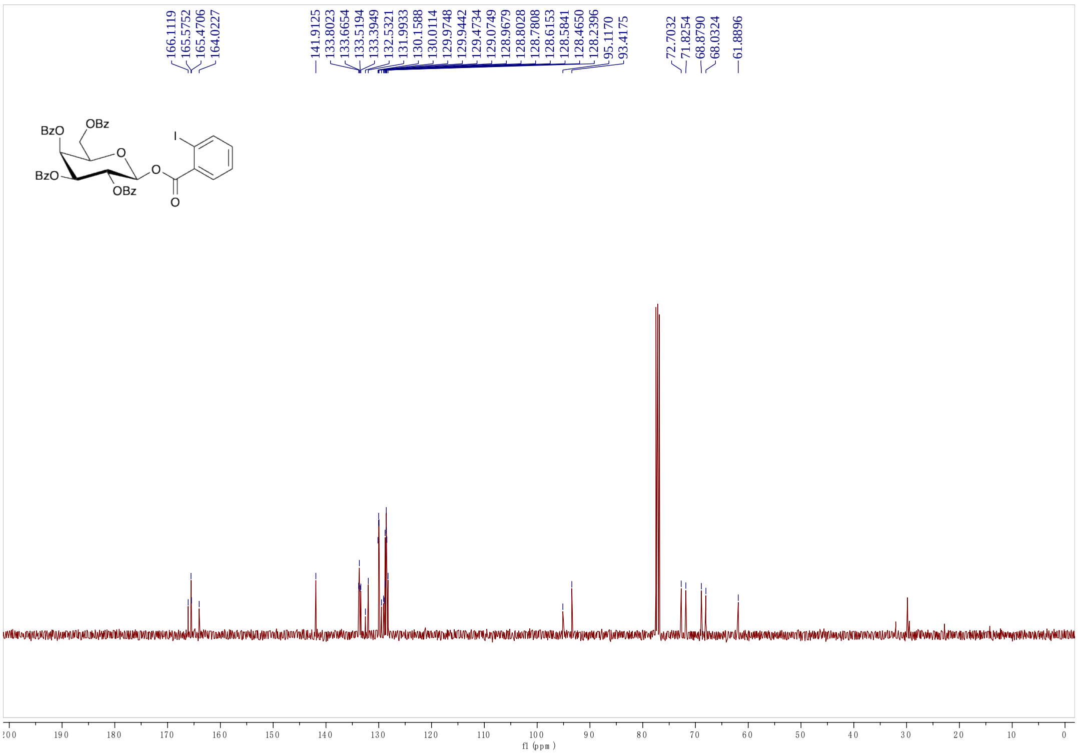

Supplementary Fig. 112 | ^{13}C NMR spectrum of 3bc (100 MHz, 25 °C, $CDCl_3$).

^{1}H spectra for **3ca**.

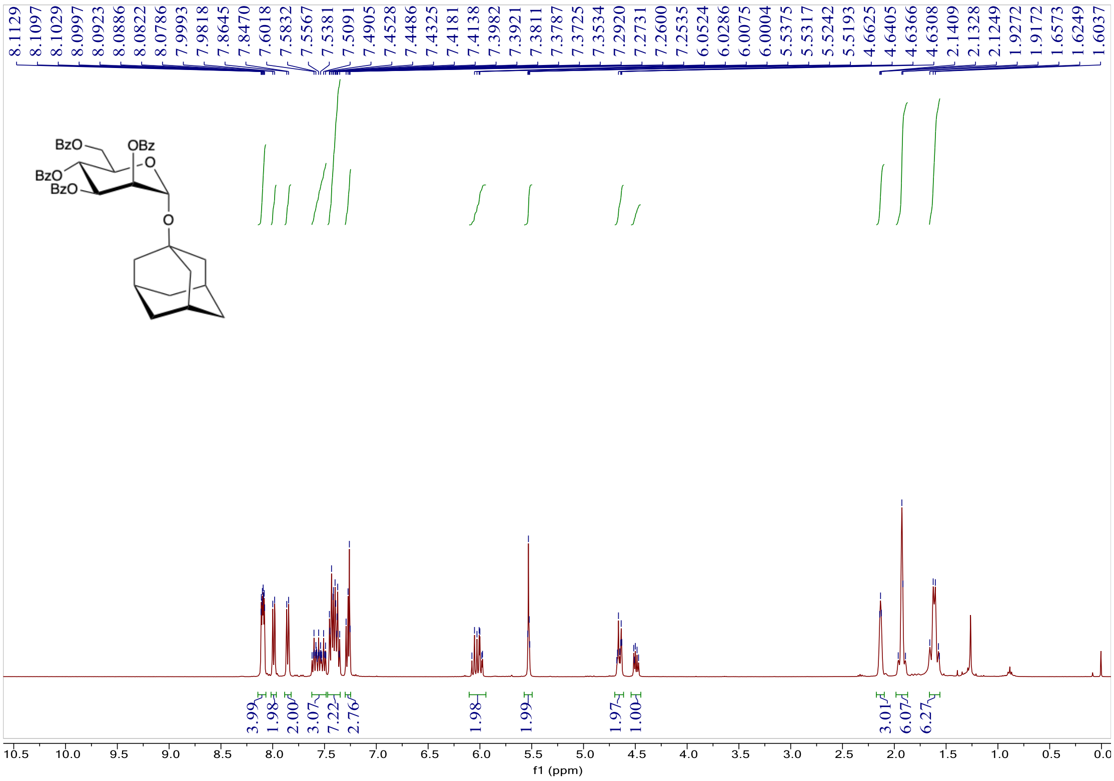

Supplementary Fig. 113 | ^{1}H NMR spectrum of 3ca (400 MHz, 25 ^{o}C, $CDCl_3$).

^{1}H and ^{13}C spectra for **3cb**.

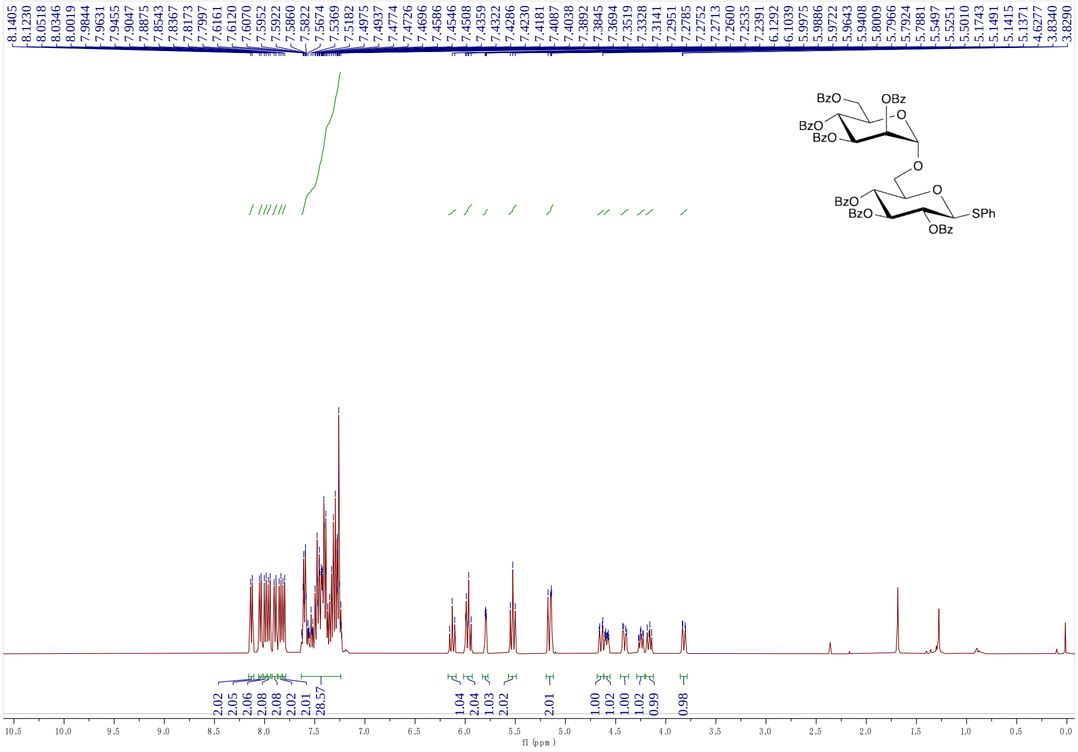

Supplementary Fig. 114 | ^{1}H NMR spectrum of 3cb (400 MHz, 25 °C, $CDCl_3$).

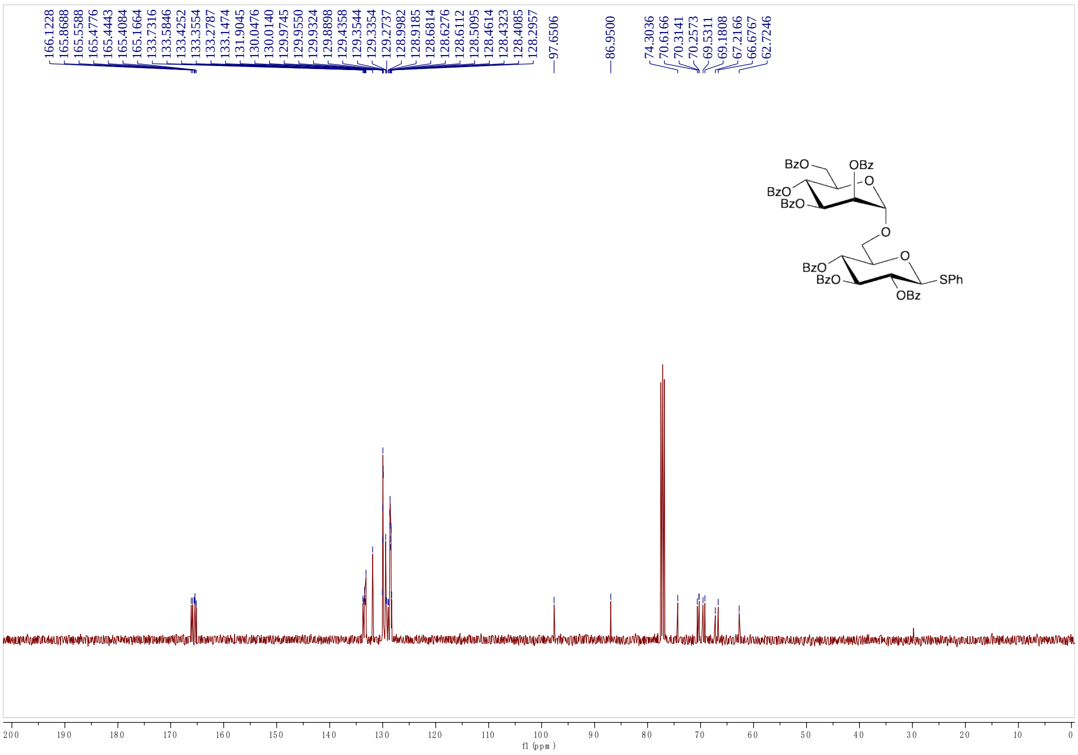

Supplementary Fig. 115 | ^{13}C NMR spectrum of 3cb (100 MHz, 25 °C, $CDCl_3$).

^{1}H and ^{13}C spectra for **3cc**.

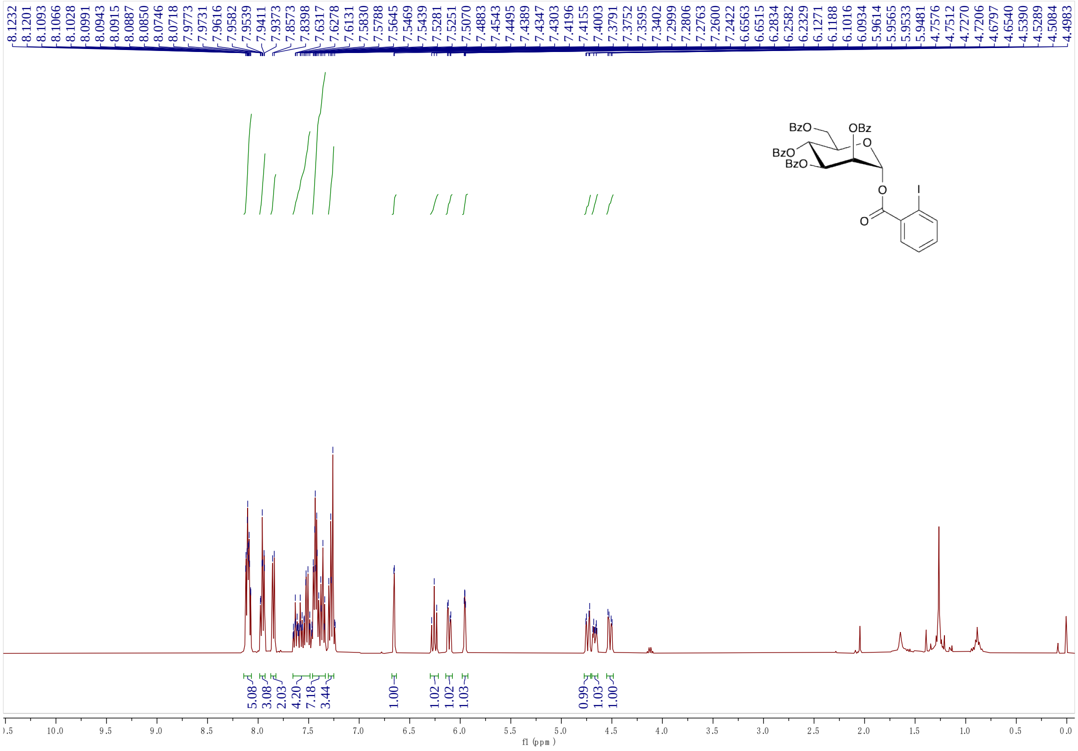

Supplementary Fig. 116 | ^{1}H NMR spectrum of 3cc (400 MHz, 25 °C, CDCl$_3$).

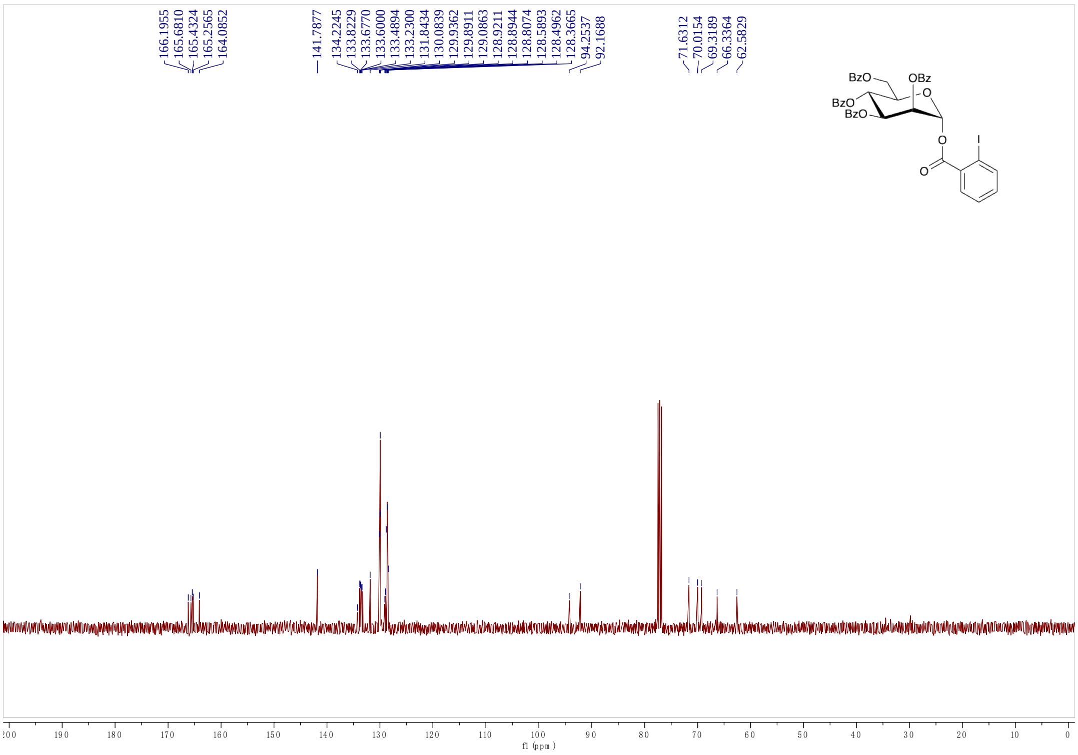

Supplementary Fig. 117 | ^{13}C NMR spectrum of 3cc (100 MHz, 25 °C, CDCl$_3$).

^{1}H spectrum for **3da**.

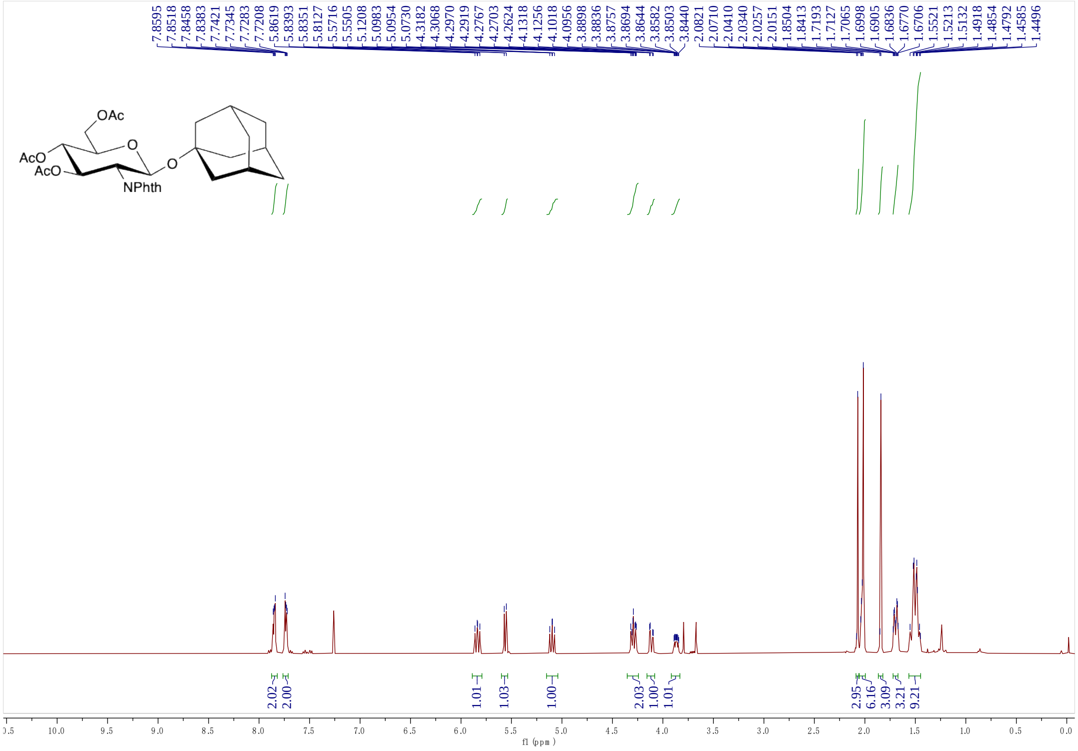

Supplementary Fig. 118 | ^{1}H NMR spectrum of 3da (400 MHz, 25 °C, $CDCl_3$).

^{1}H and ^{13}C spectra for **3db**.

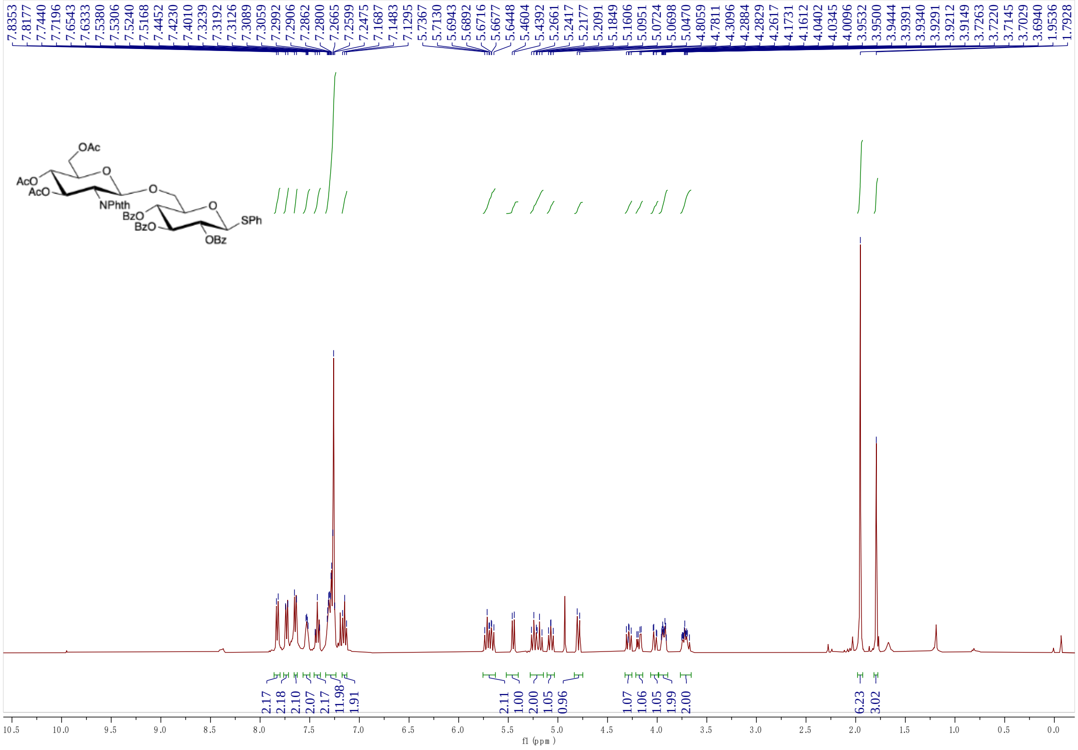

Supplementary Fig. 119 | ^{1}H NMR spectrum of 3db (400 MHz, 25 °C, $CDCl_3$).

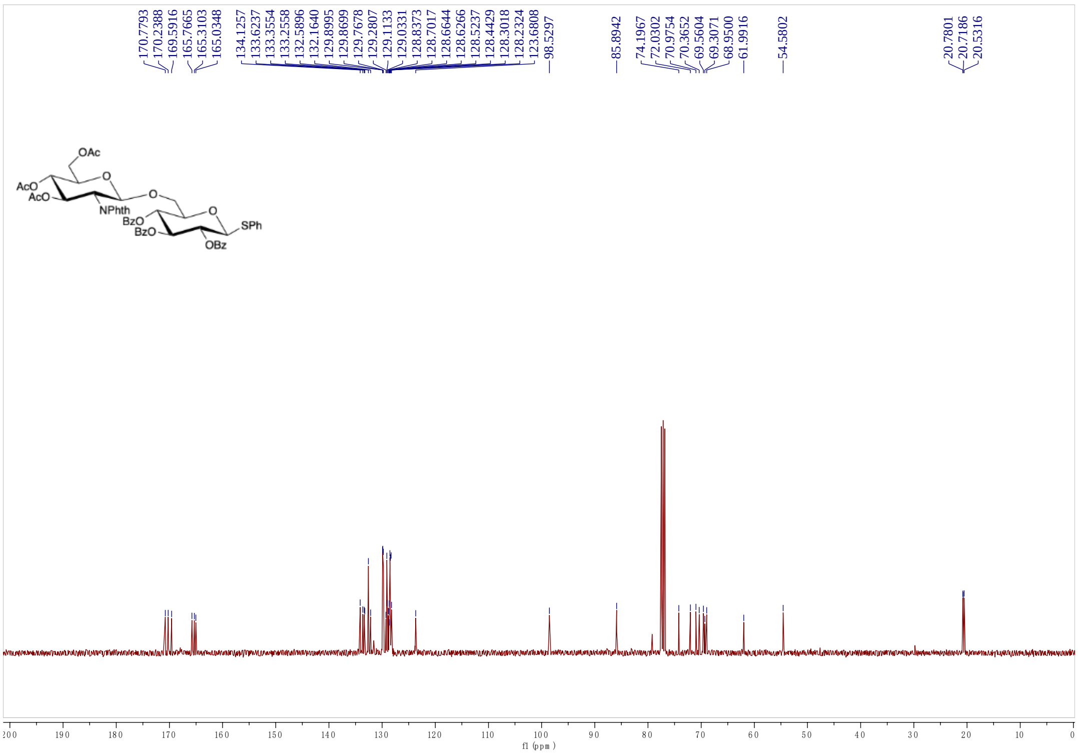

Supplementary Fig. 120 | ^{13}C NMR spectrum of 3db (100 MHz, 25 °C, $CDCl_3$).

^{1}H and ^{13}C spectra for **3dc**.

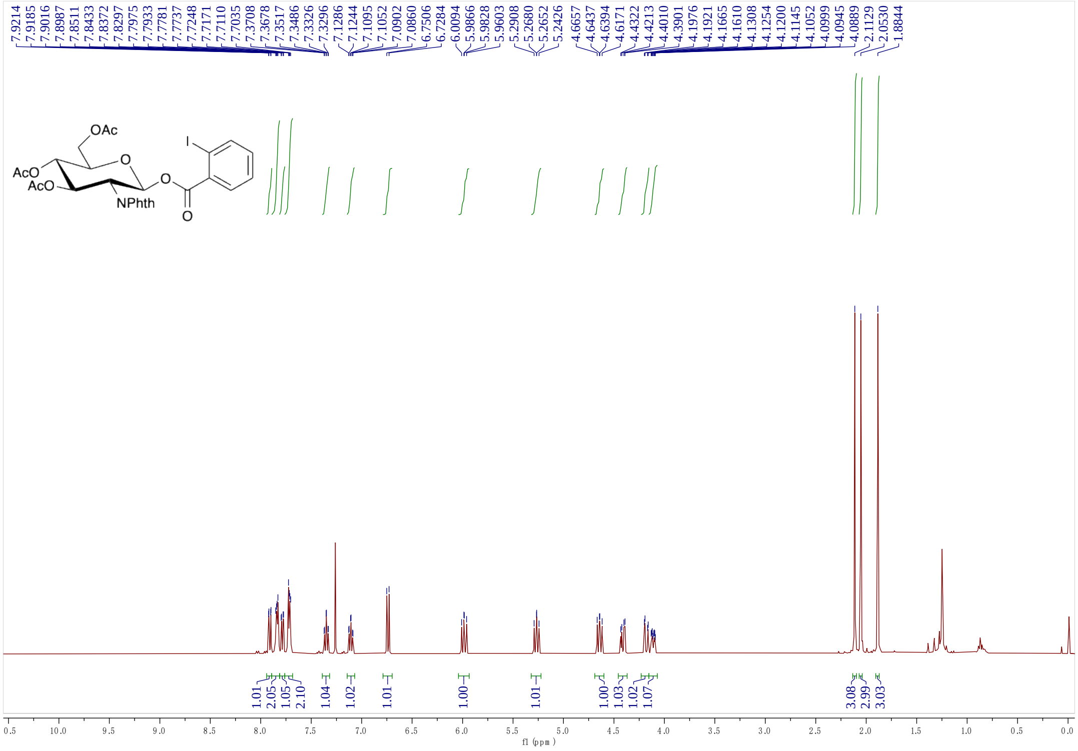

Supplementary Fig. 121 | ^{1}H NMR spectrum of 3dc (400 MHz, 25 °C, $CDCl_3$).

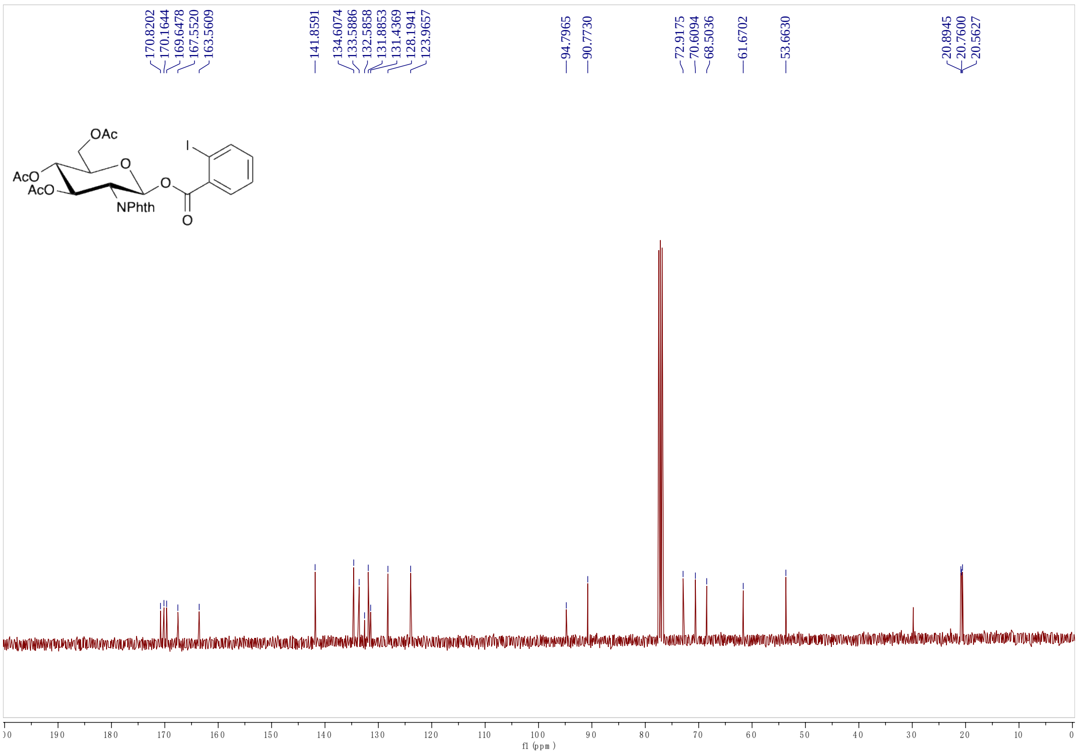

Supplementary Fig. 122 | ^{13}C NMR spectrum of 3dc (100 MHz, 25 °C, $CDCl_3$).

^{1}H and ^{13}C spectra for **3ea**.

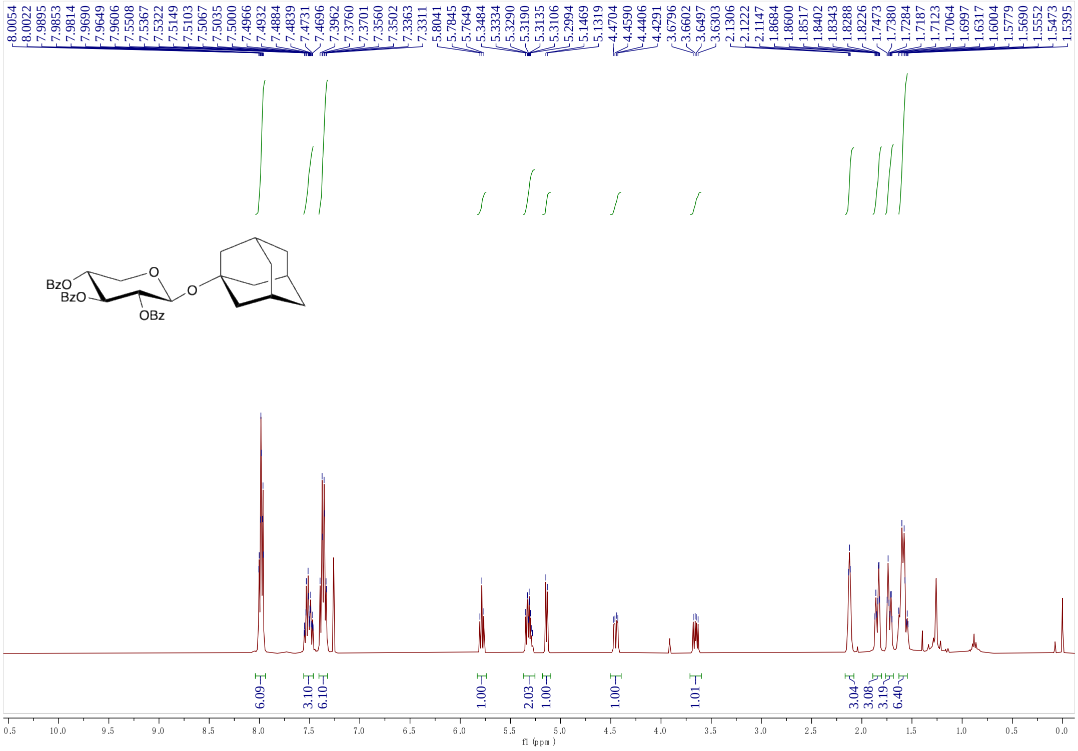

Supplementary Fig. 123 | ^{1}H NMR spectrum of 3ea (400 MHz, 25 °C, $CDCl_3$).

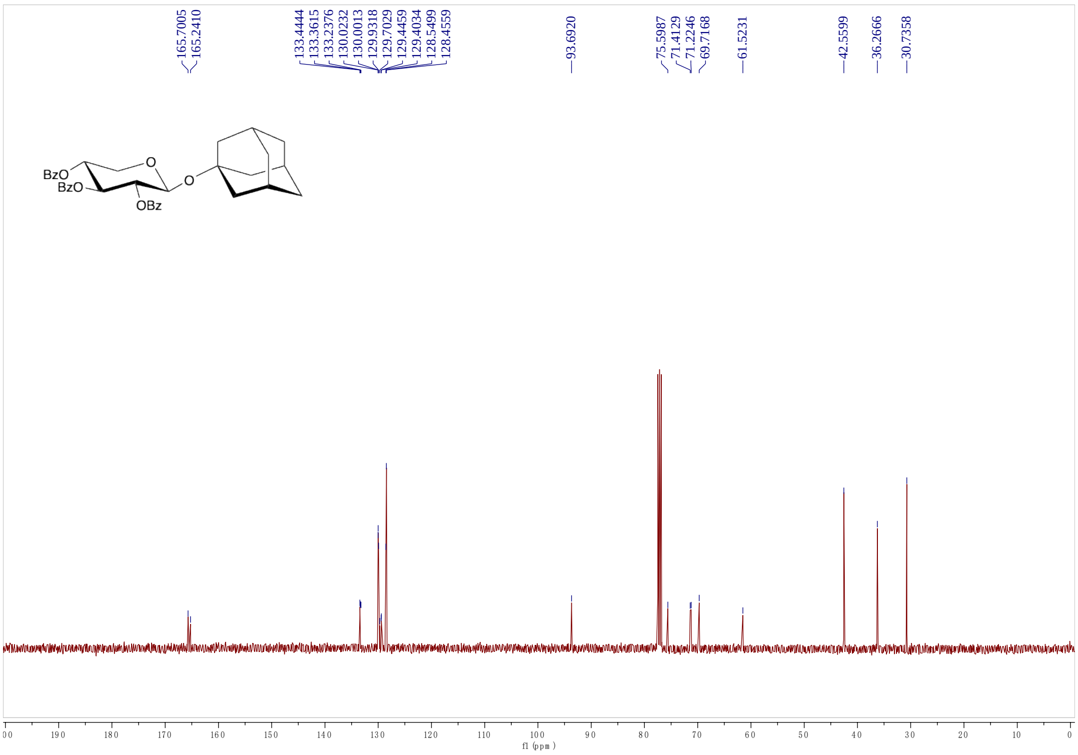

Supplementary Fig. 124 | ^{13}C NMR spectrum of 3ea (100 MHz, 25 °C, $CDCl_3$).

^{1}H and ^{13}C spectra for **3eb**.

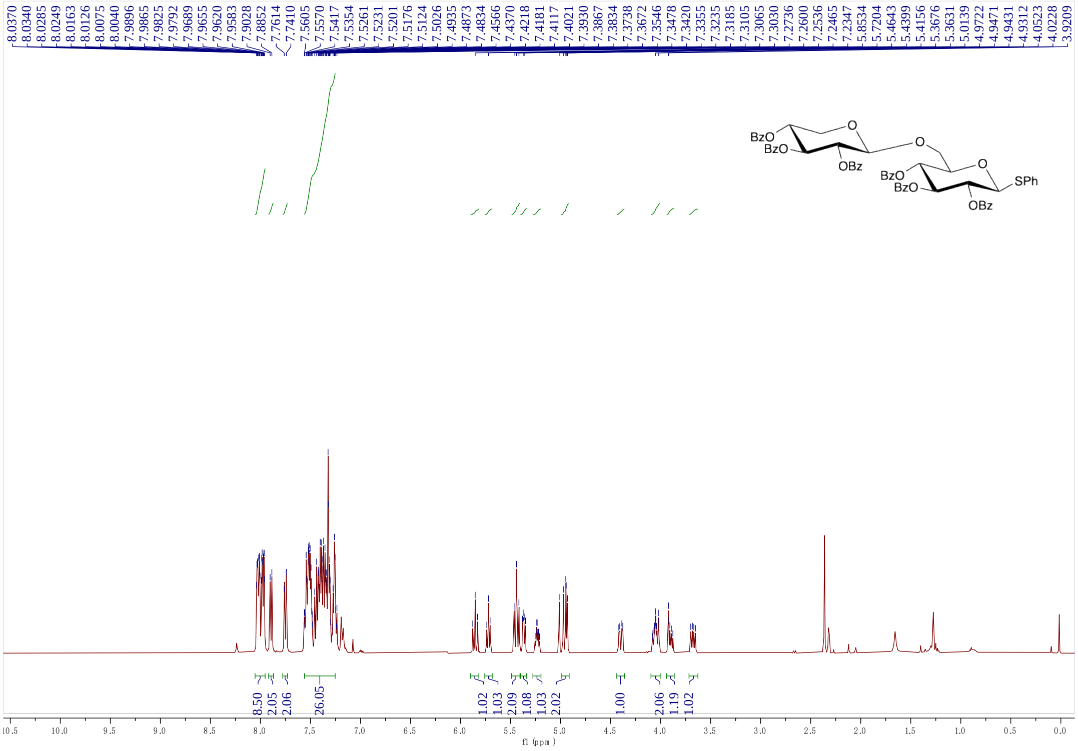

Supplementary Fig. 125 | ^{1}H NMR spectrum of 3eb (400 MHz, 25 °C, $CDCl_3$).

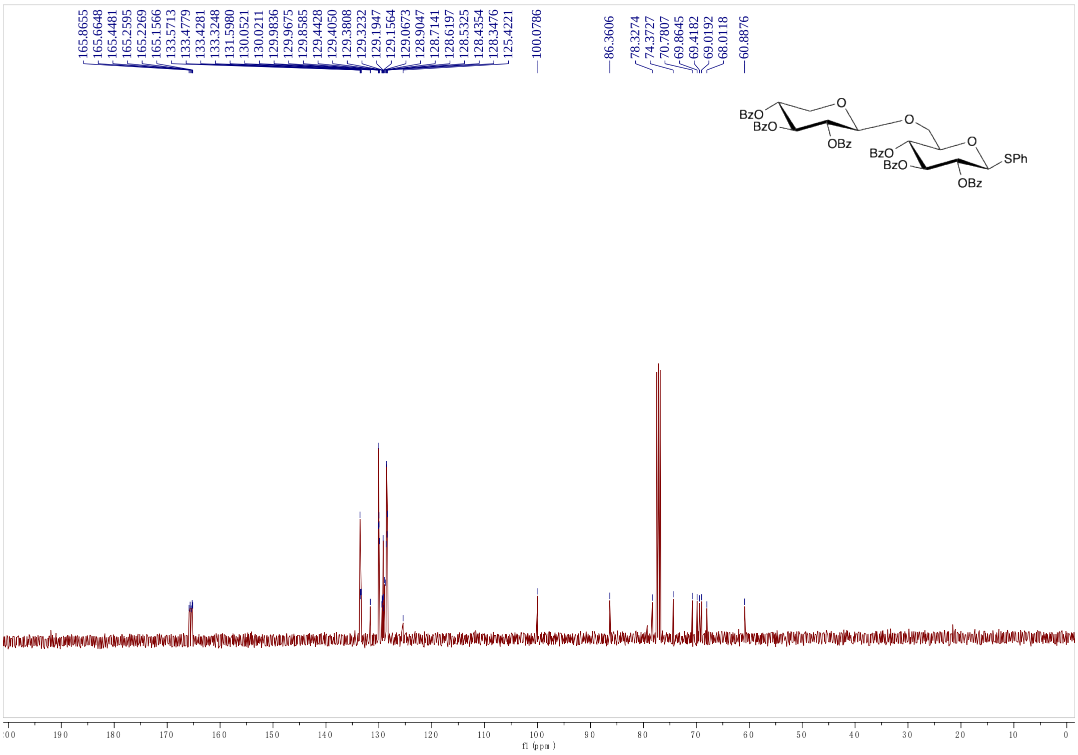

Supplementary Fig. 126 | ^{13}C NMR spectrum of 3eb (100 MHz, 25 °C, $CDCl_3$).

^{1}H and ^{13}C spectra for **3ec**.

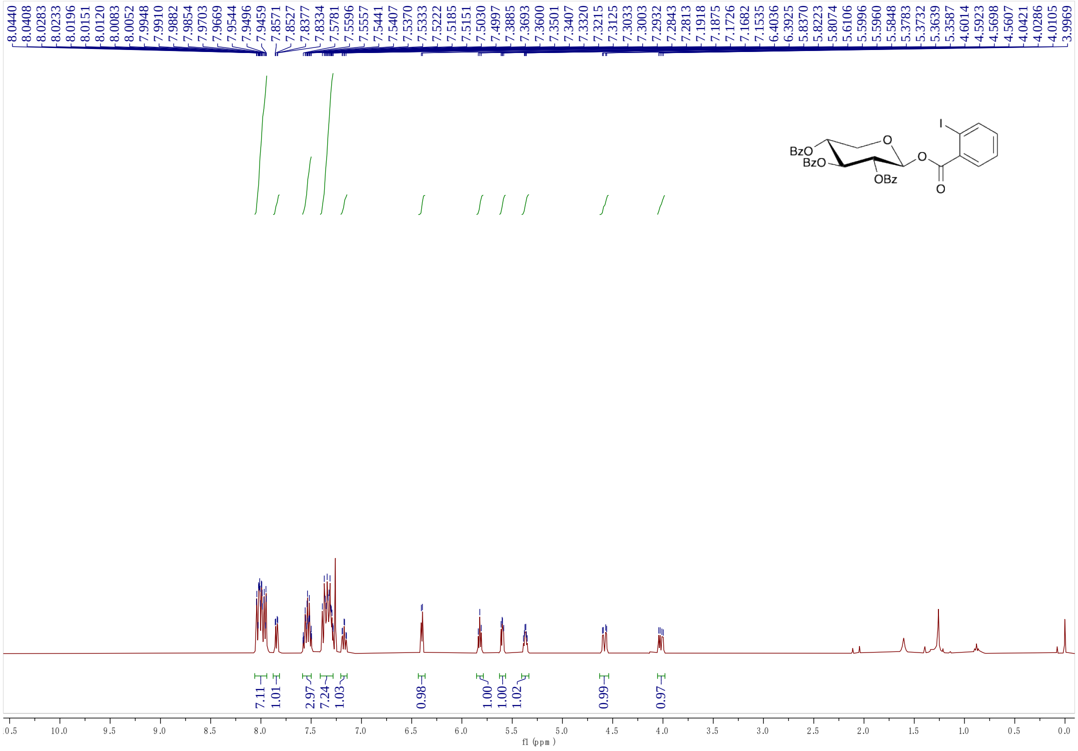

Supplementary Fig. 127 | ^{1}H NMR spectrum of 3ec (400 MHz, 25 °C, $CDCl_3$).

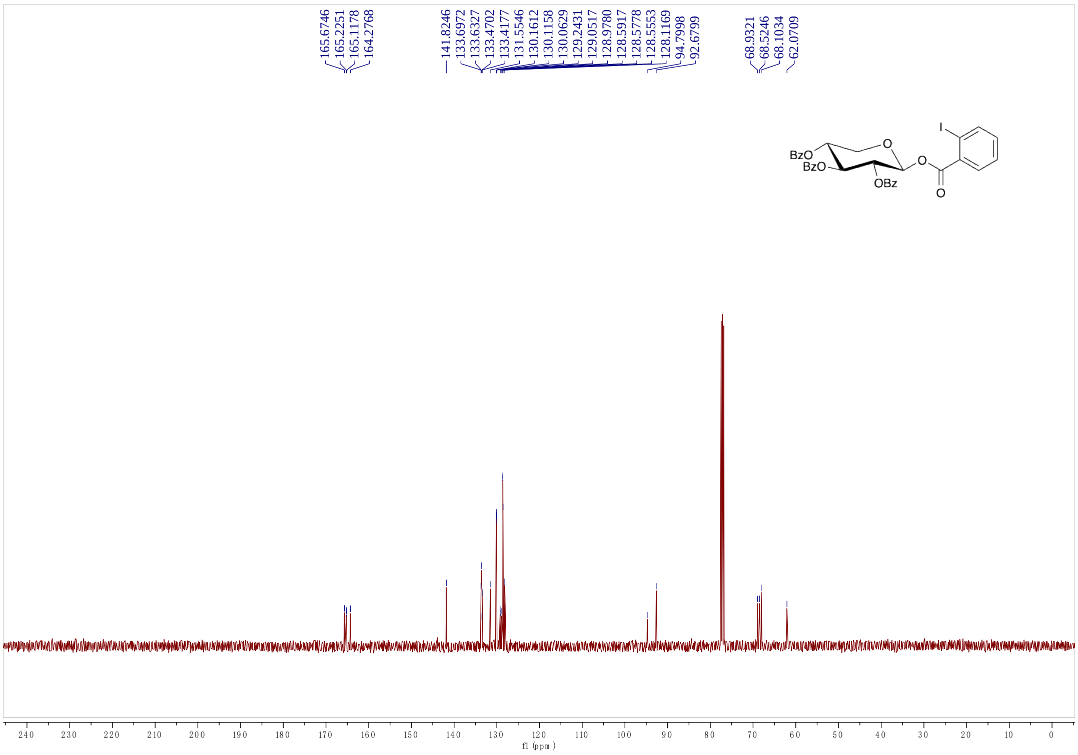

Supplementary Fig. 128 | ^{13}C NMR spectrum of 3ec (100 MHz, 25 °C, $CDCl_3$).

^{1}H spectrum for **3fa**.

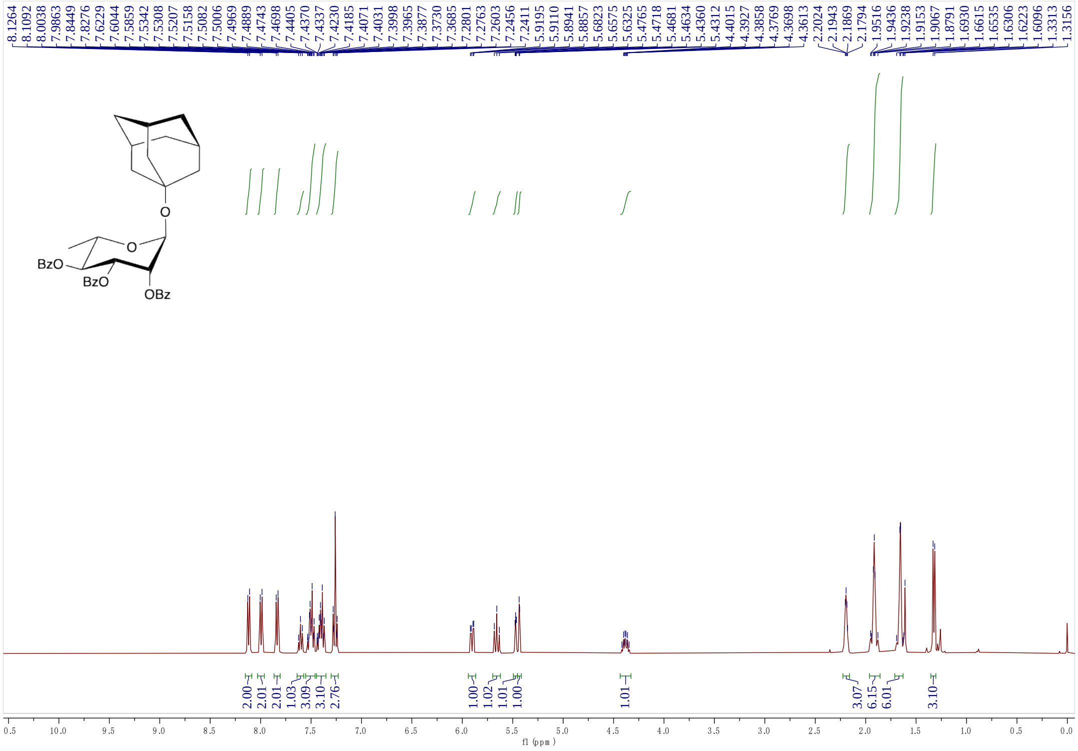

Supplementary Fig. 129 | ^{1}H NMR spectrum of 3fa (400 MHz, 25 ^{o}C, $CDCl_3$).

^{1}H and ^{13}C spectra for **3fb**.

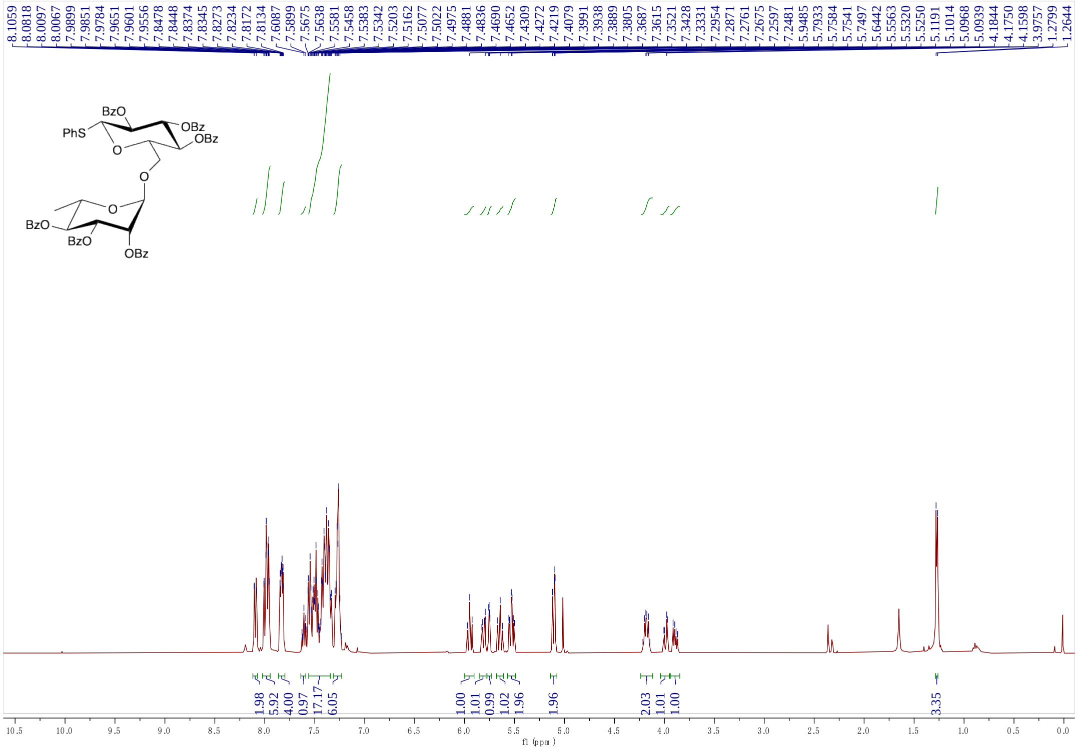

Supplementary Fig. 130 | ^{1}H NMR spectrum of 3fb (400 MHz, 25 °C, $CDCl_3$).

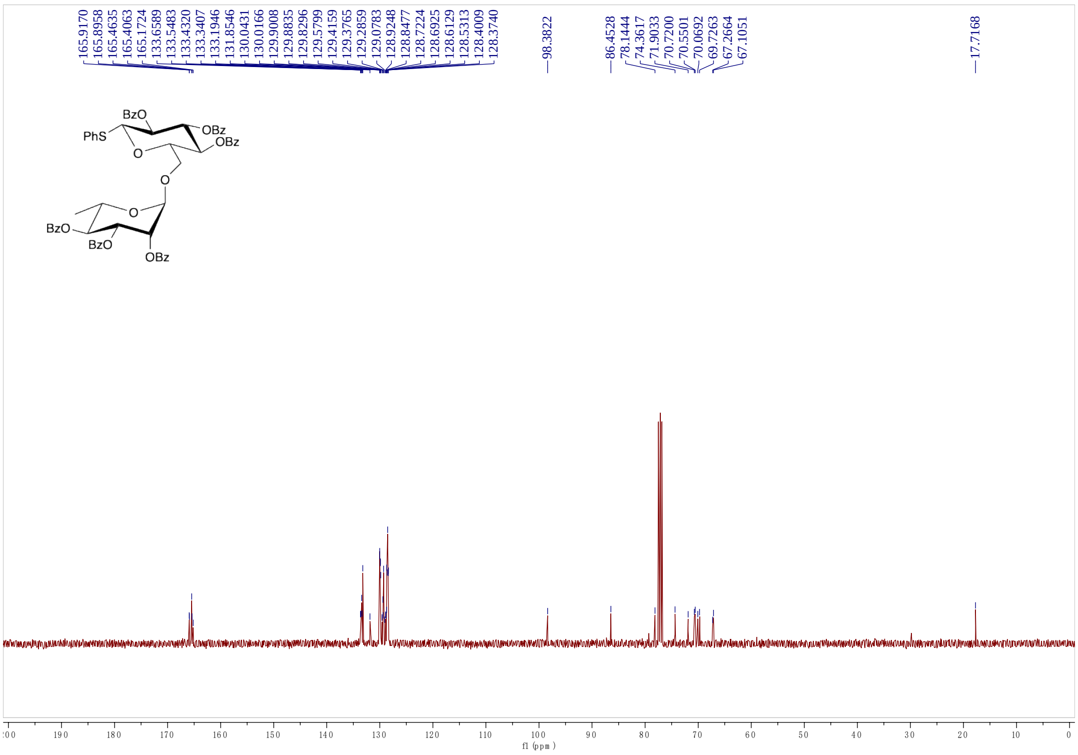

Supplementary Fig. 131 | ^{13}C NMR spectrum of 3fb (100 MHz, 25 °C, $CDCl_3$).

^{1}H and ^{13}C spectra for **3fc**.

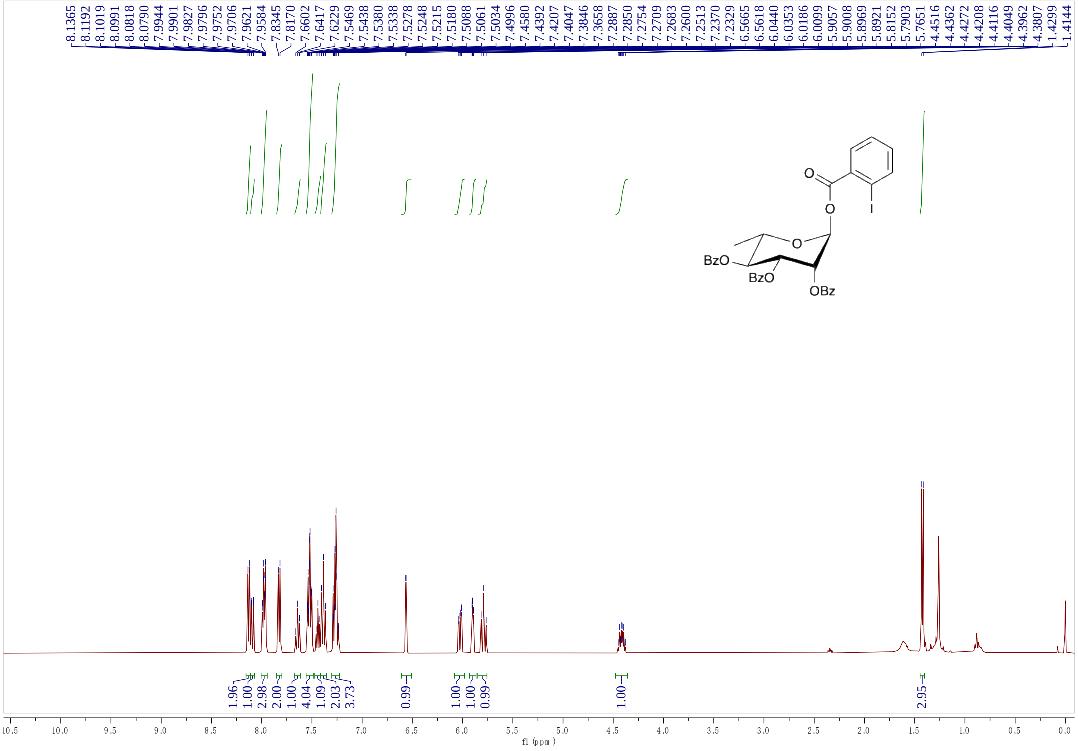

Supplementary Fig. 132 | ^{1}H NMR spectrum of 3fc (400 MHz, 25 °C, CDCl$_3$).

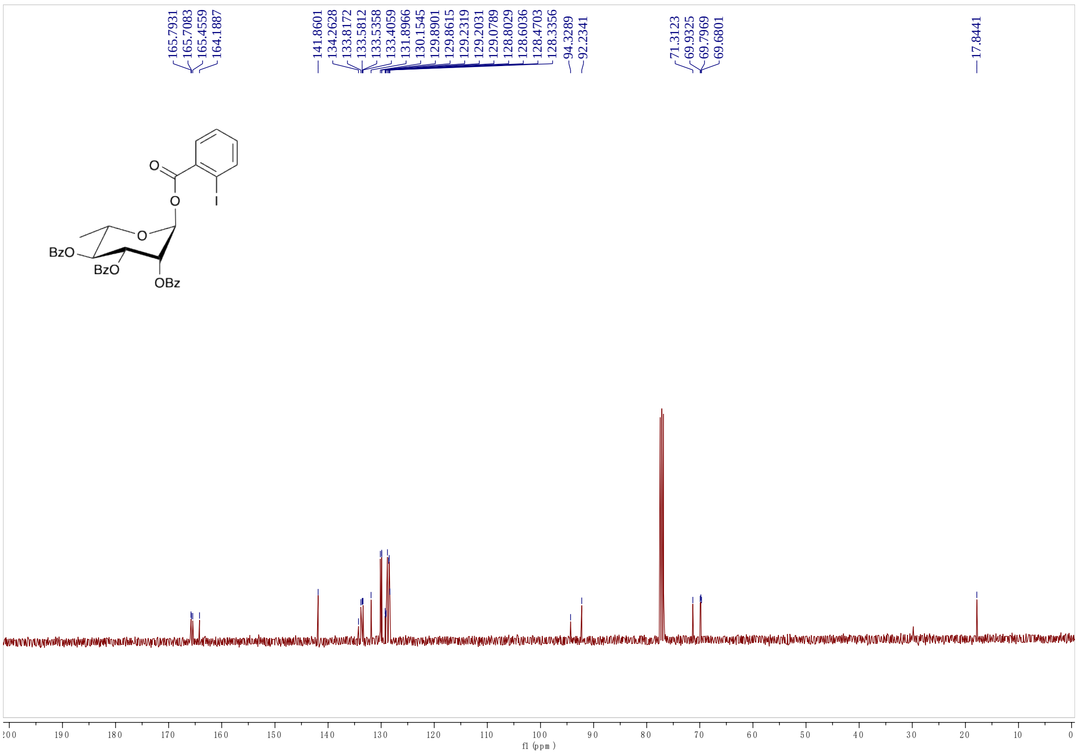

Supplementary Fig. 133 | ^{13}C NMR spectrum of 3fc (100 MHz, 25 °C, CDCl$_3$).

¹H spectrum for **3ga**.

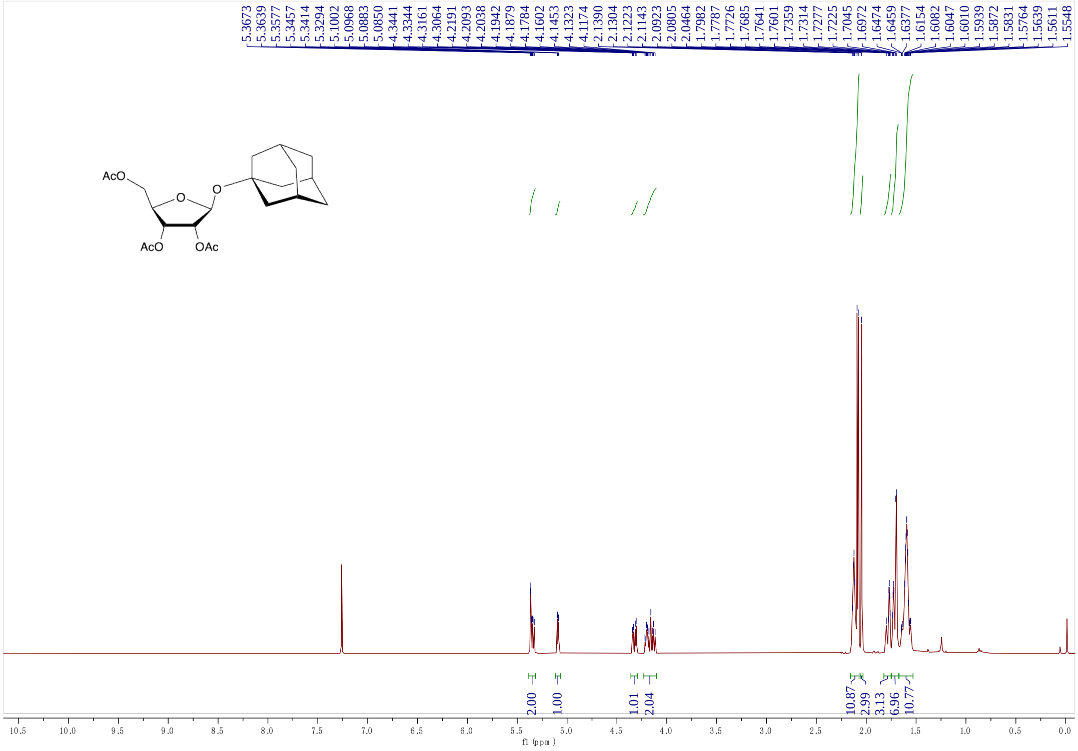

Supplementary Fig. 134 | ¹H NMR spectrum of 3ga (400 MHz, 25 °C, $CDCl_3$).

^1H and ^{13}C spectra for **3gb**.

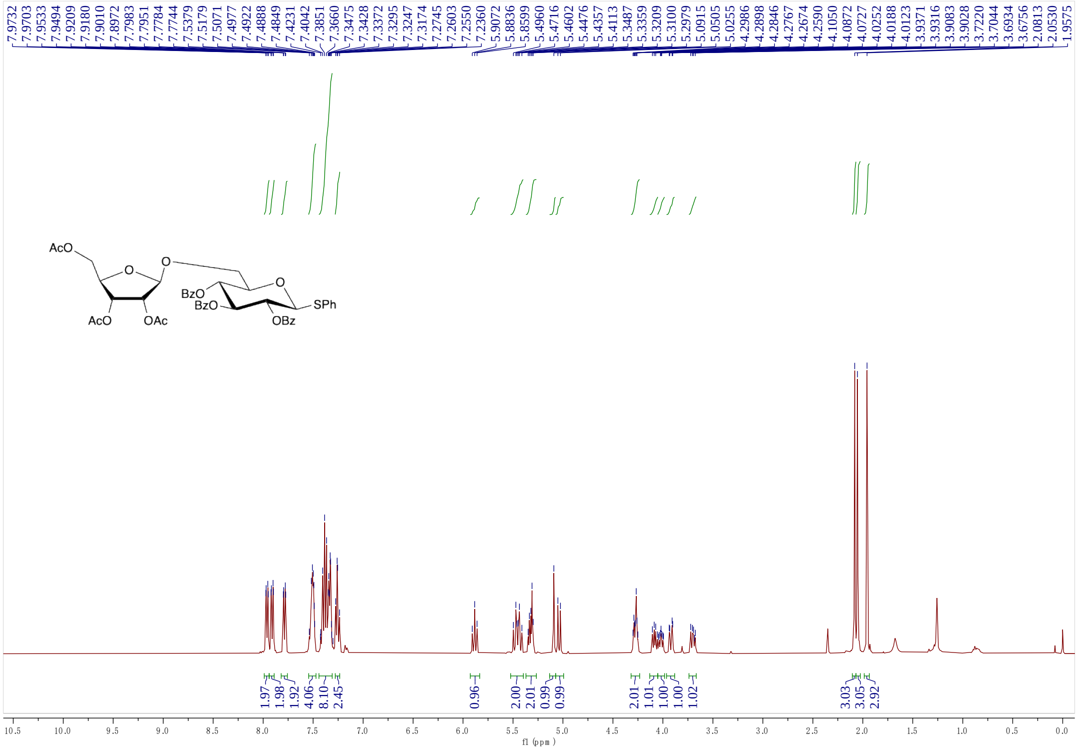

Supplementary Fig. 135 | ^1H NMR spectrum of 3gb (400 MHz, 25 °C, $CDCl_3$).

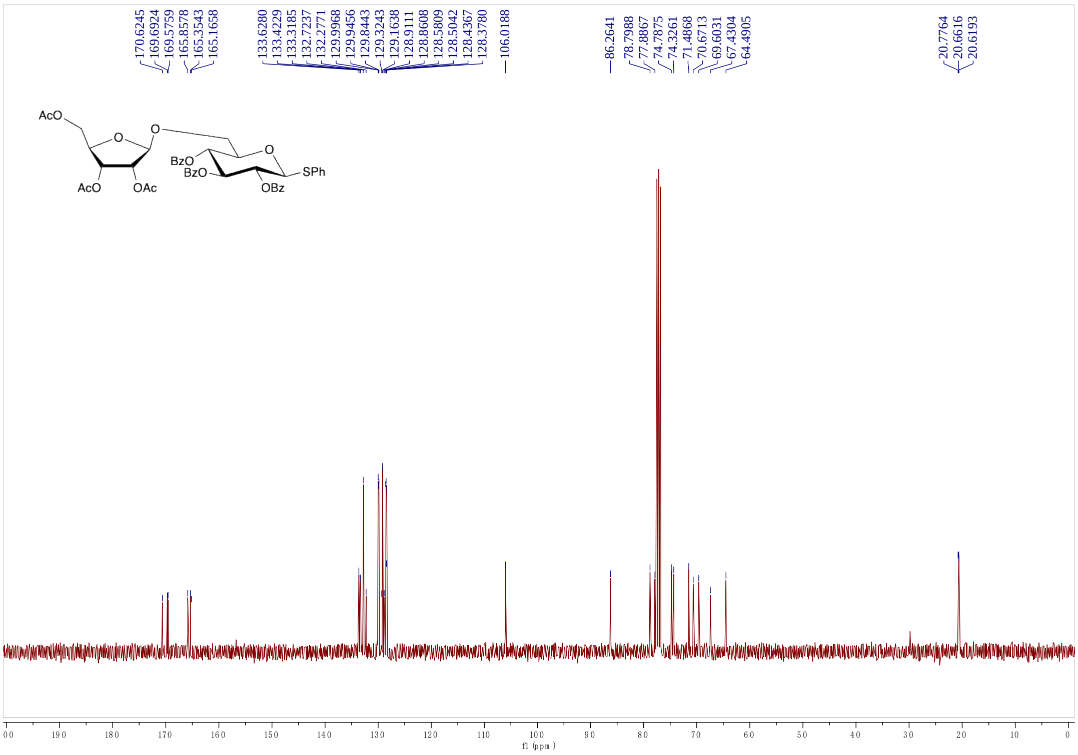

Supplementary Fig. 136 | ^{13}C NMR spectrum of 3gb (100 MHz, 25 °C, $CDCl_3$).

^1H and ^{13}C spectra for **3gc**.

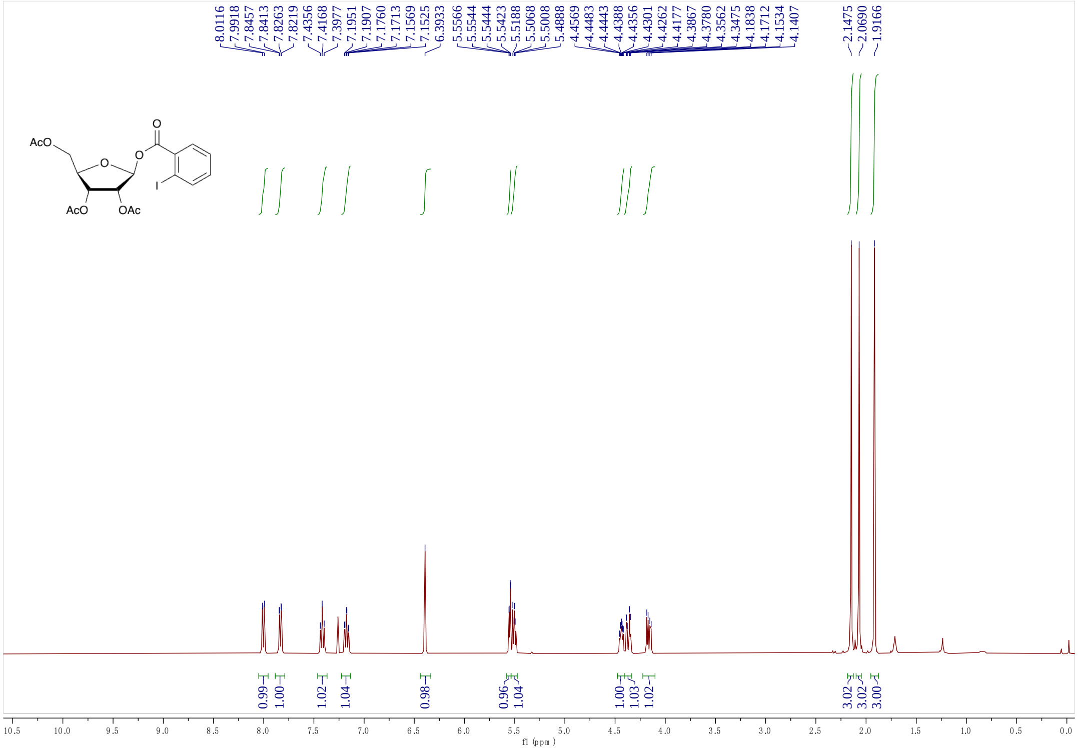

Supplementary Fig. 137 | ^1H NMR spectrum of 3gc (400 MHz, 25 °C, $CDCl_3$).

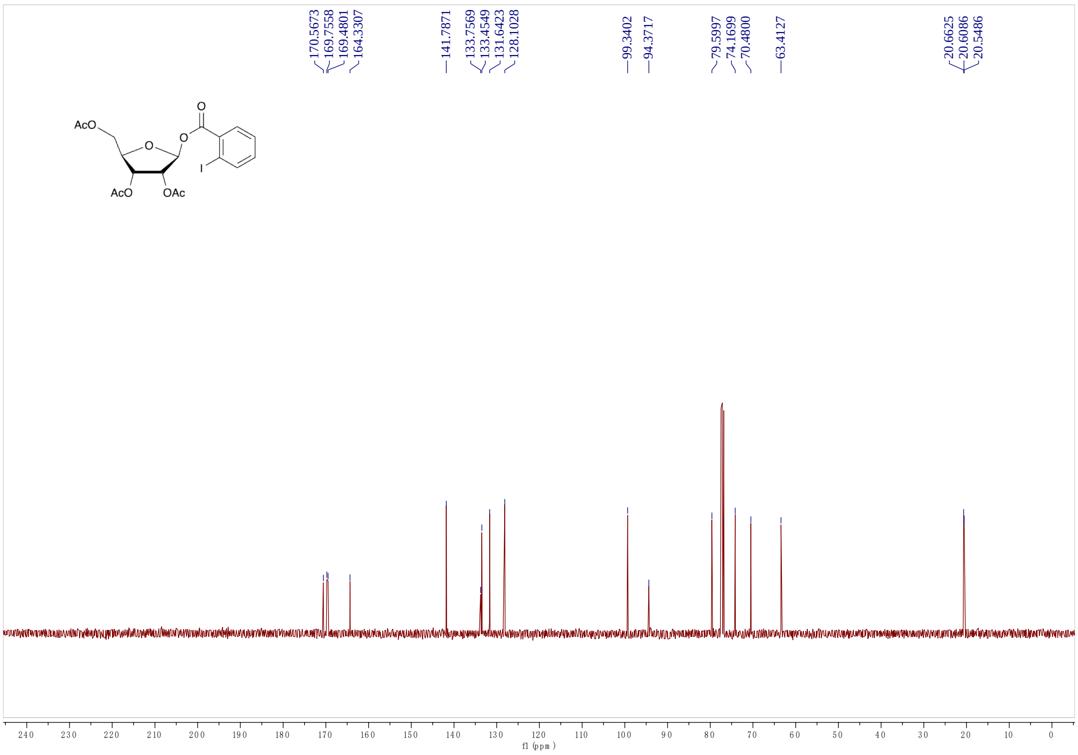

Supplementary Fig. 138 | ^{13}C NMR spectrum of 3gc (100 MHz, 25 °C, $CDCl_3$).

^{1}H and ^{13}C spectra for **3ha**.

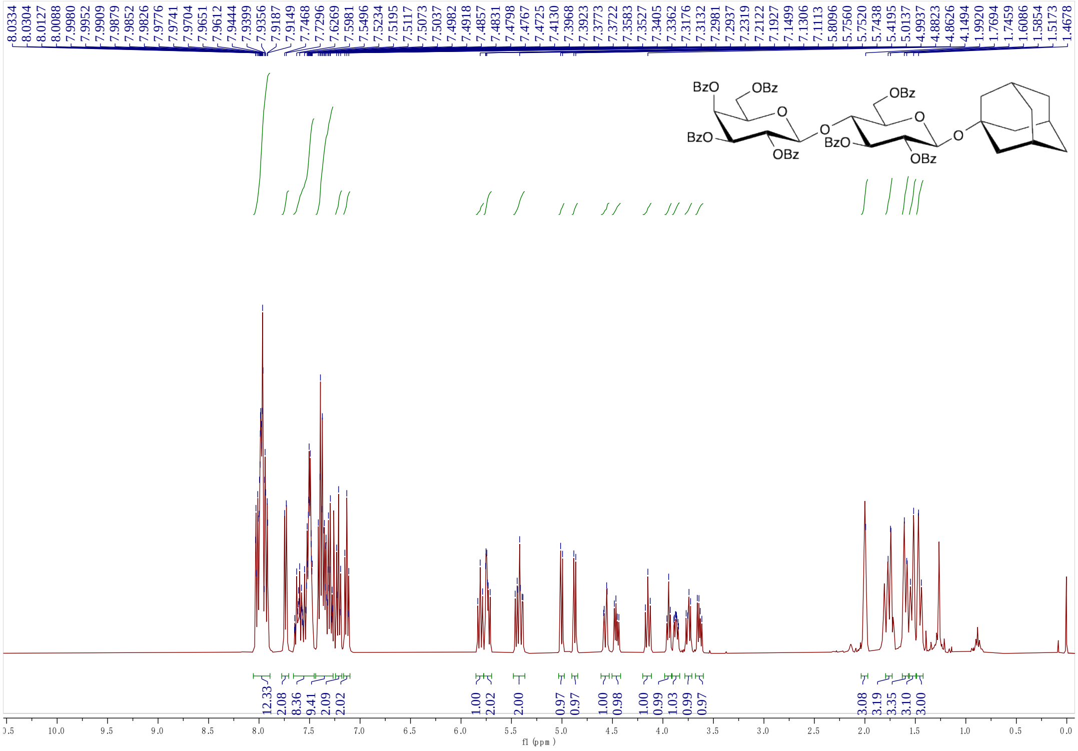

Supplementary Fig. 139 | ^{1}H NMR spectrum of 3ha (400 MHz, 25 °C, $CDCl_3$).

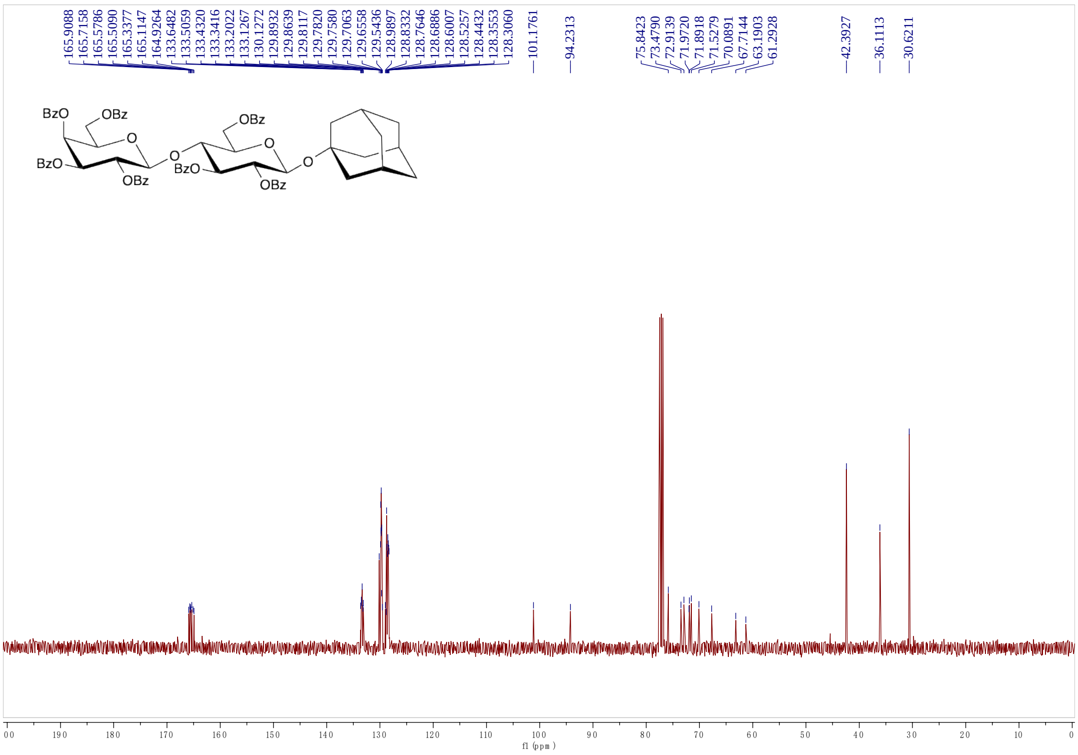

Supplementary Fig. 140 | ^{13}C NMR spectrum of 3ha (100 MHz, 25 °C, $CDCl_3$).

^{1}H and ^{13}C spectra for **3hb**.

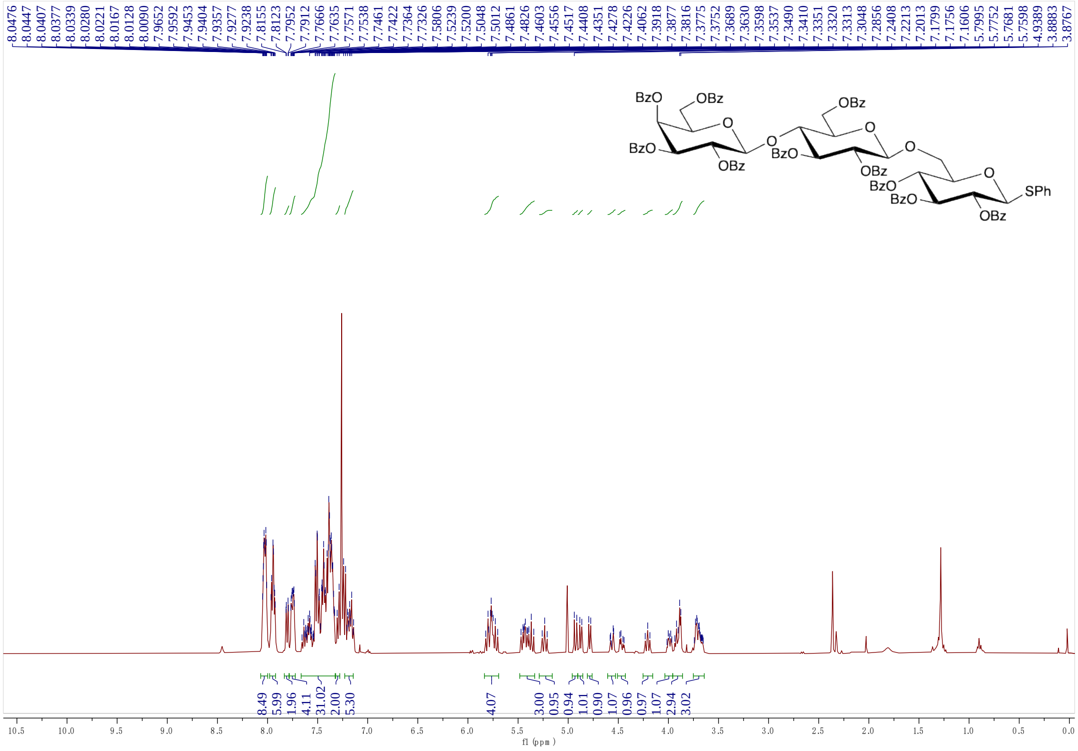

Supplementary Fig. 141 | ^{1}H NMR spectrum of 3hb (400 MHz, 25 °C, $CDCl_3$).

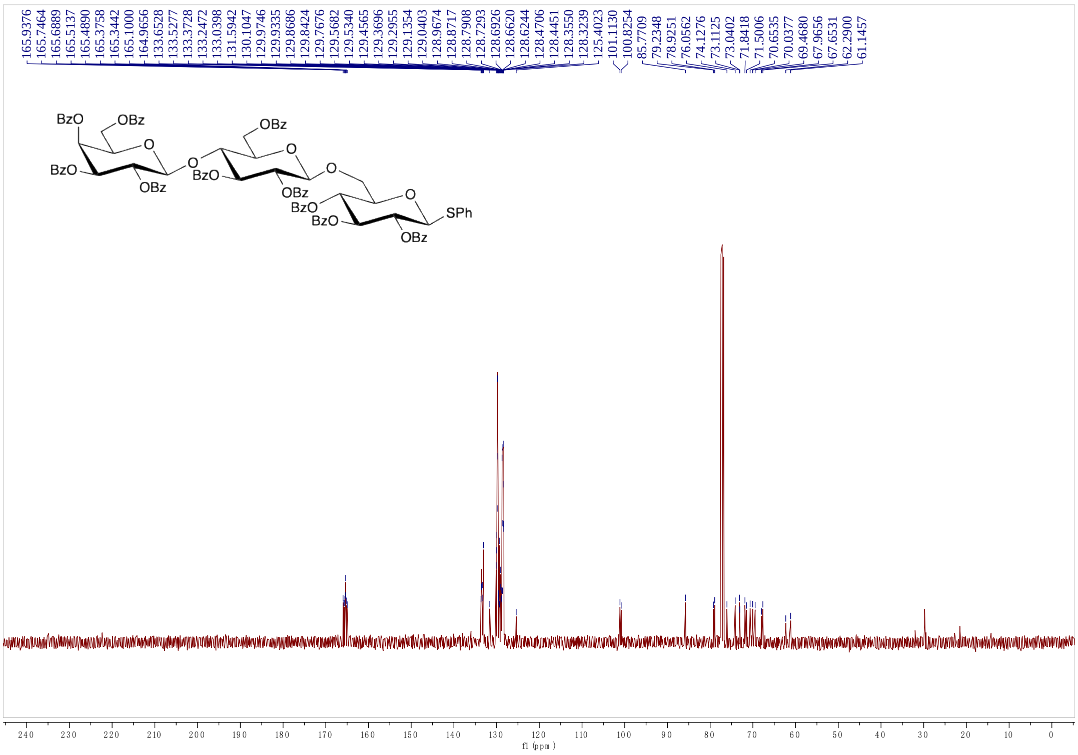

Supplementary Fig. 142 | ^{13}C NMR spectrum of 3hb (100 MHz, 25 °C, $CDCl_3$).

^{1}H and ^{13}C spectra for **3hc**.

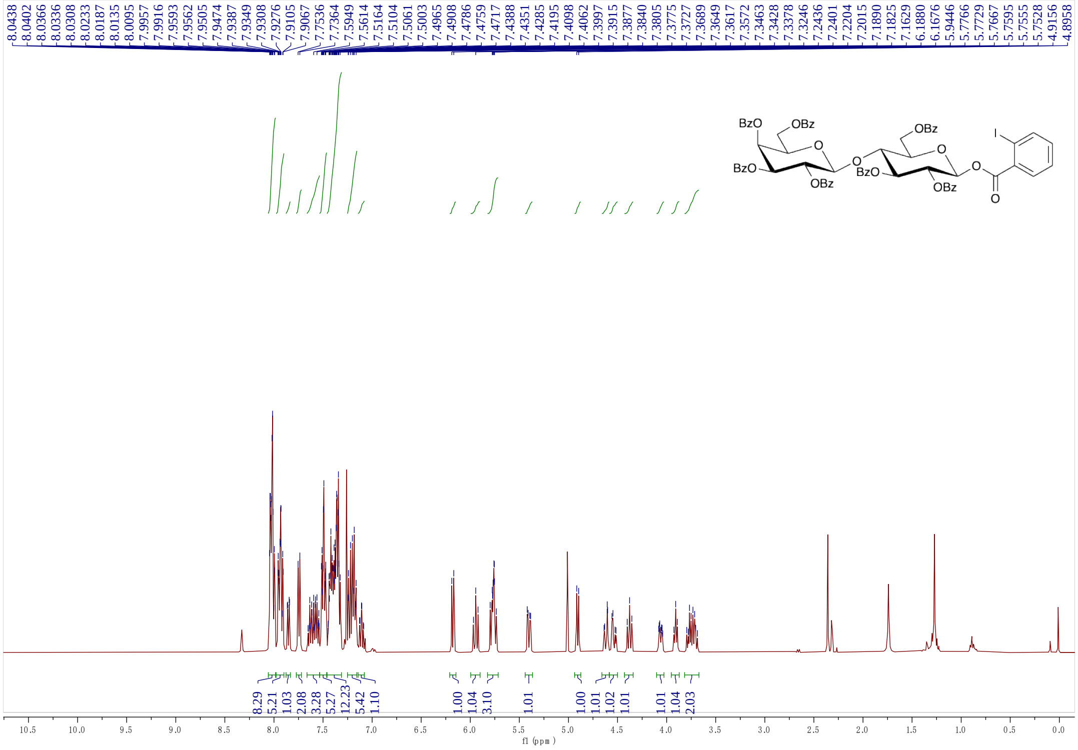

Supplementary Fig. 143 | ^{1}H NMR spectrum of 3hc (400 MHz, 25 °C, $CDCl_3$).

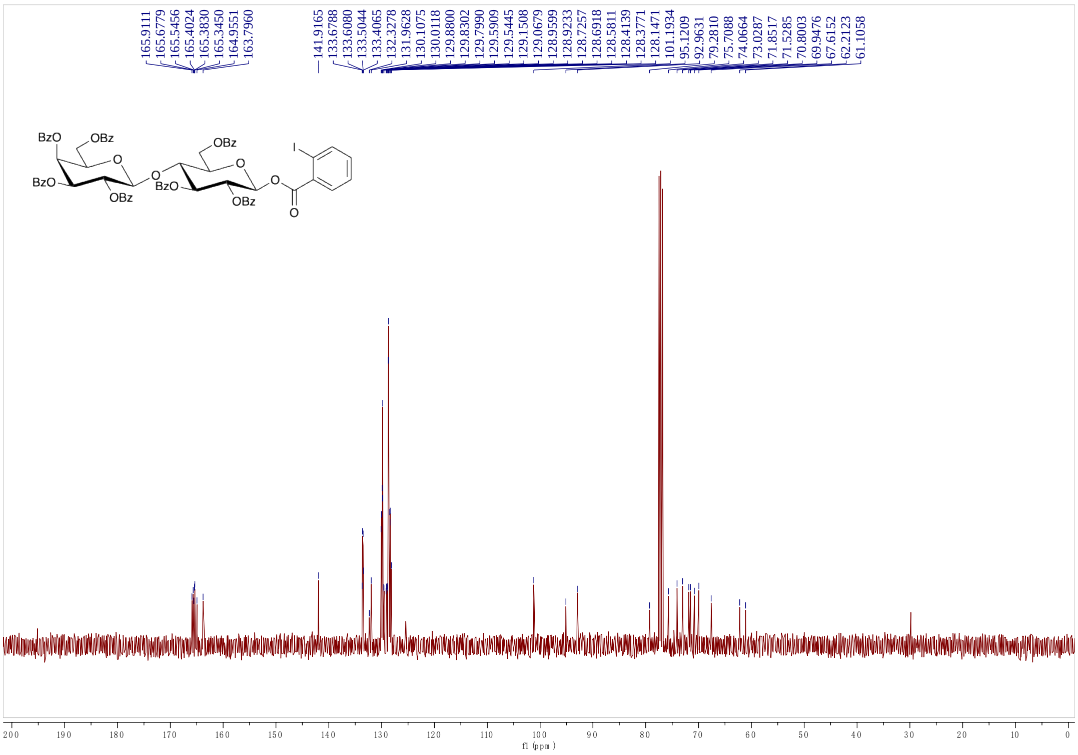

Supplementary Fig. 144 | ^{13}C NMR spectrum of 3hc (100 MHz, 25 °C, $CDCl_3$).

^{1}H and ^{13}C spectra for **7**.

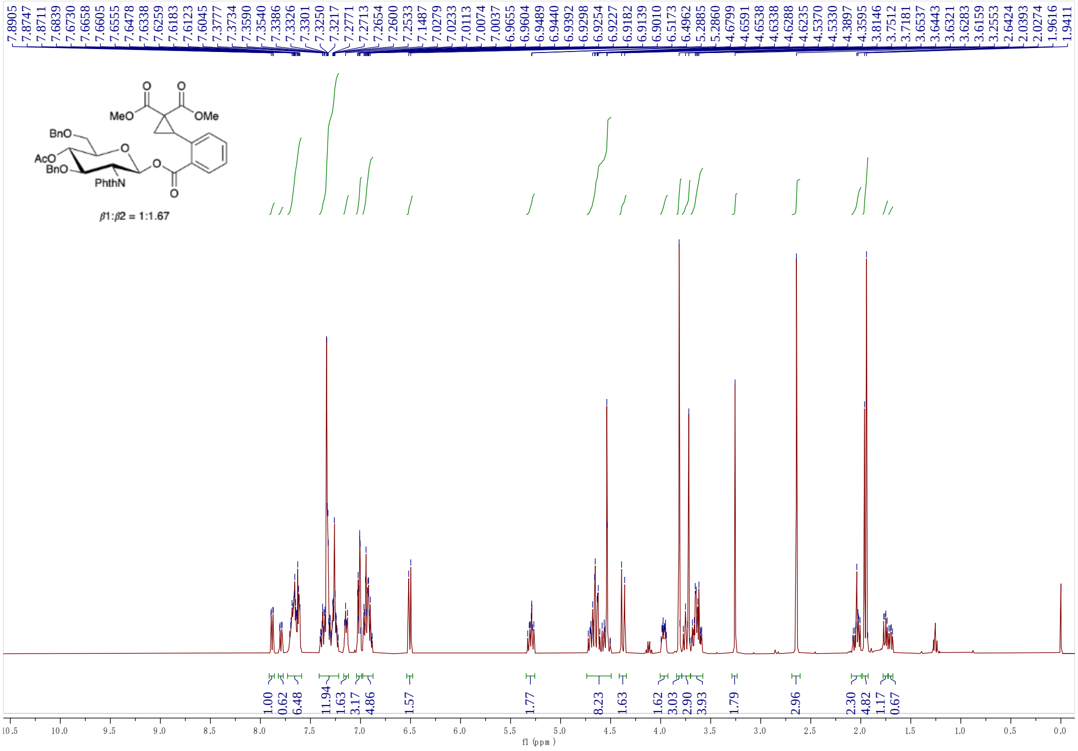

Supplementary Fig. 145 | ^{1}H NMR spectrum of 7 (400 MHz, 25 °C, $CDCl_3$).

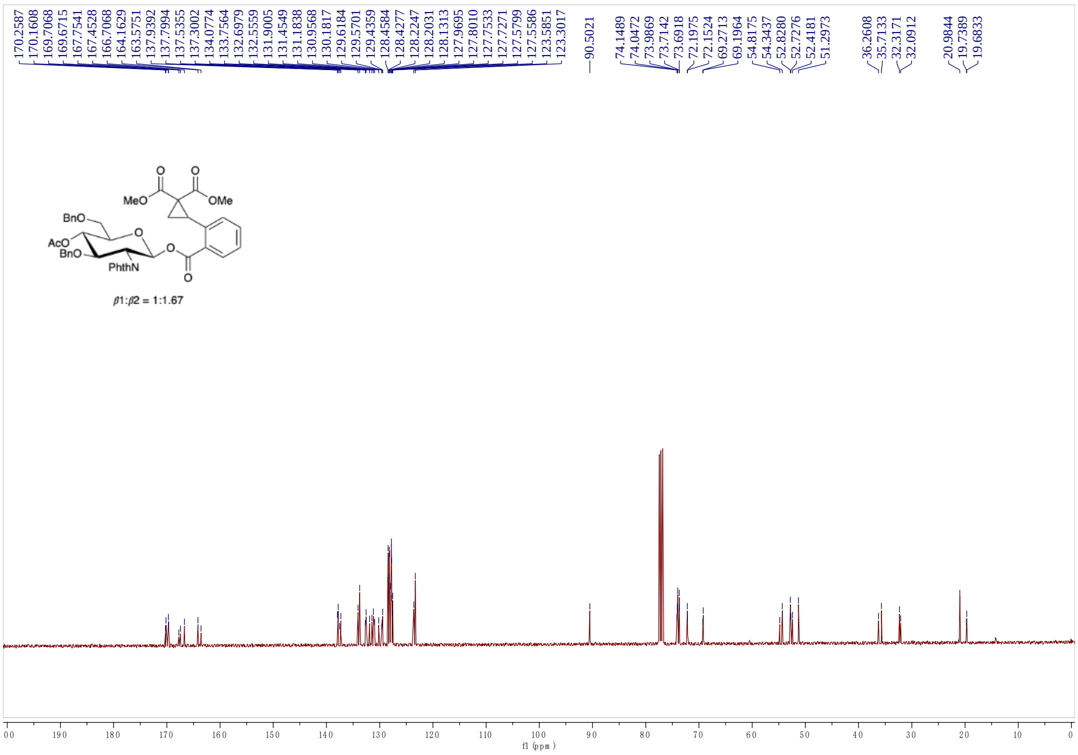

Supplementary Fig. 146 | ^{13}C NMR spectrum of 7 (100 MHz, 25 °C, $CDCl_3$).

^{1}H spectrum for **9**.

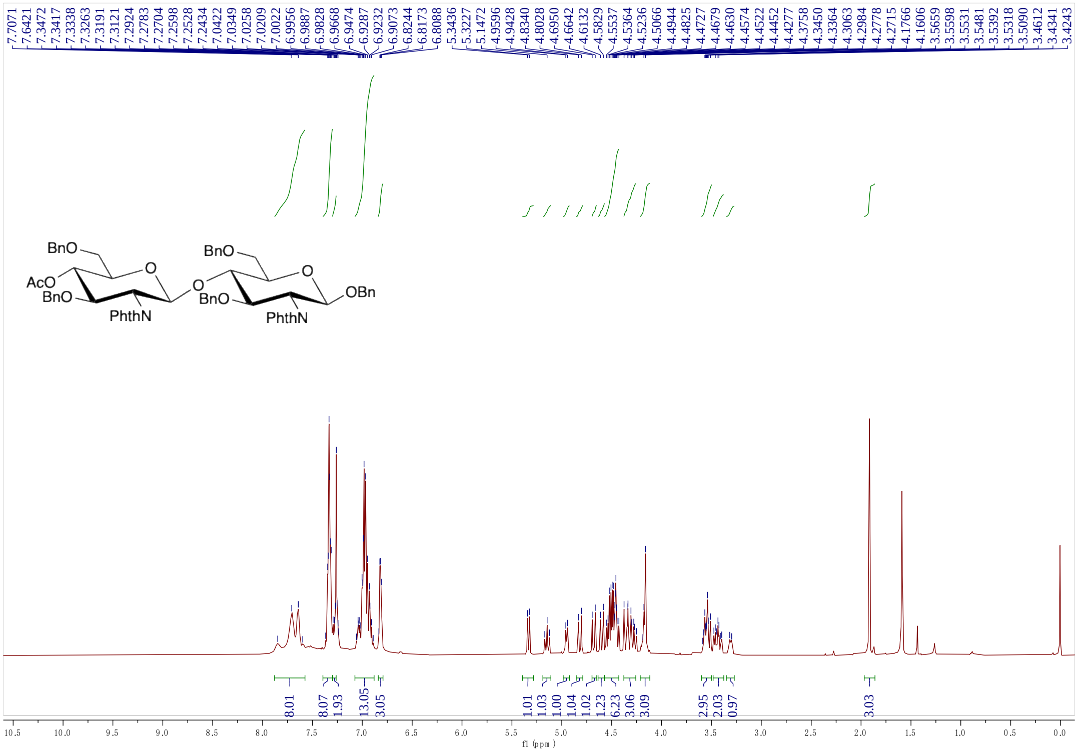

Supplementary Fig. 147 | ^{1}H NMR spectrum of 9 (400 MHz, 25 °C, $CDCl_3$).

^{1}H spectrum for **10**.

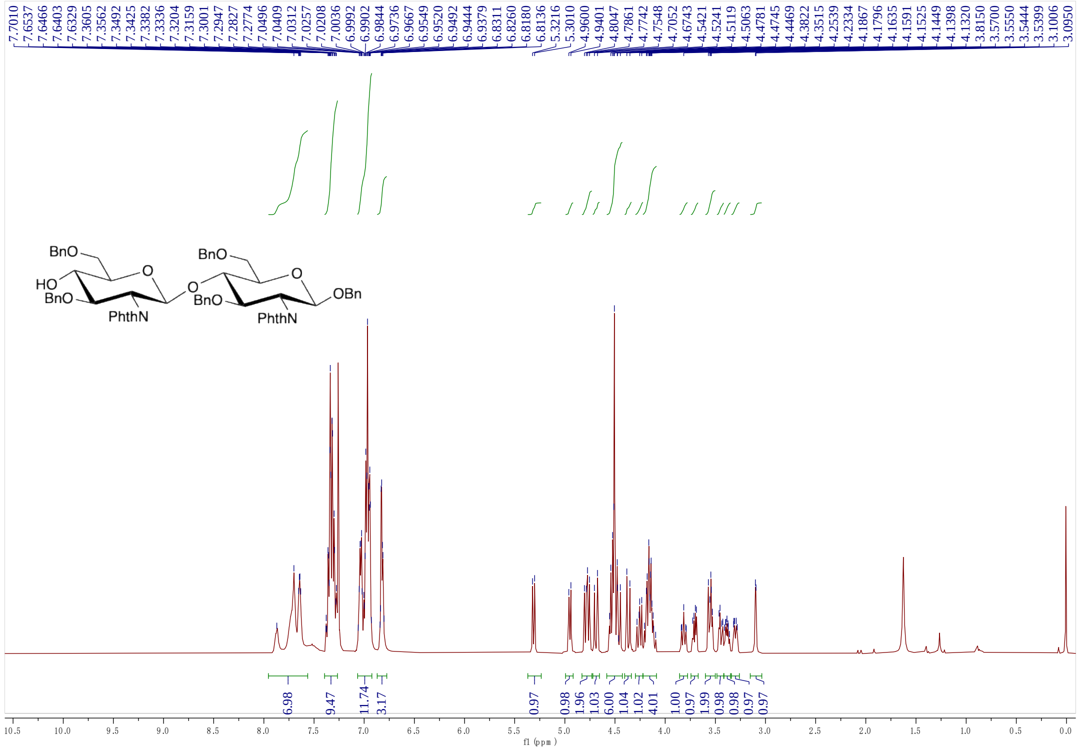

Supplementary Fig. 148 | ^{1}H NMR spectrum of 10 (400 MHz, 25 °C, $CDCl_3$).

^{1}H, ^{13}C, ^{1}H-^{1}H COSY and ^{1}H-^{13}C HSQC spectra for **11**.

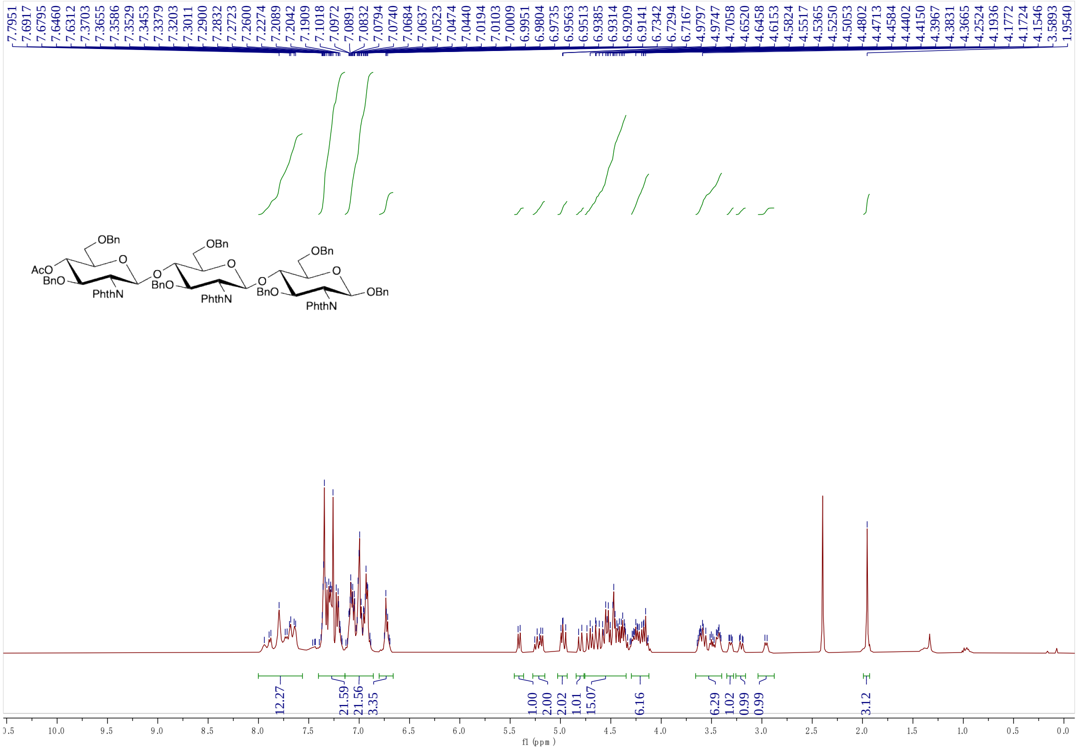

Supplementary Fig. 149 | ^{1}H NMR spectrum of 11 (400 MHz, 25 °C, $CDCl_3$).

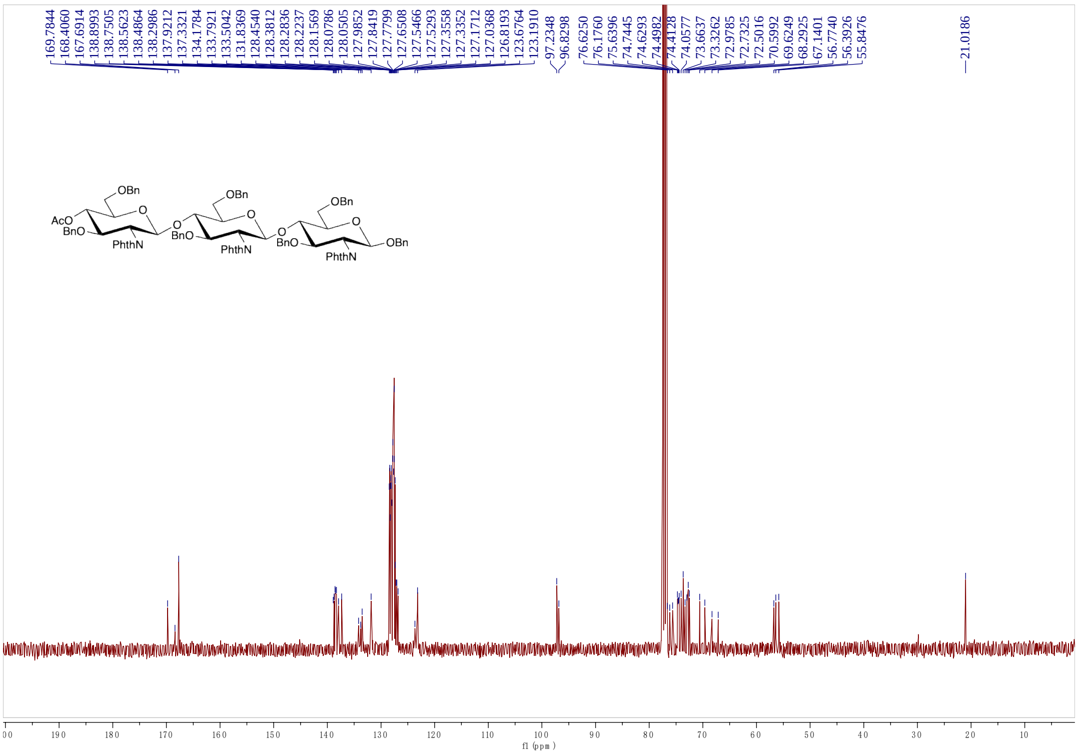

Supplementary Fig. 150 | ^{13}C NMR spectrum of 11 (100 MHz, 25 °C, $CDCl_3$).

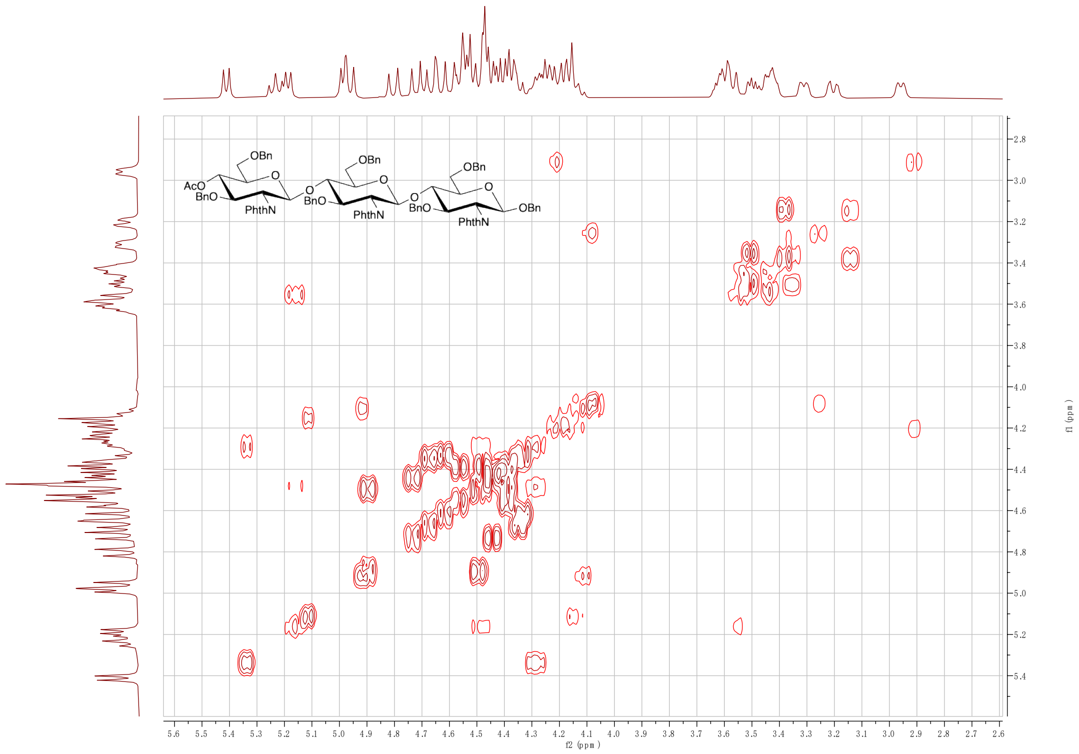

Supplementary Fig. 151 | ^{1}H-^{1}H COSY NMR spectrum of 11 (25 °C, $CDCl_3$).

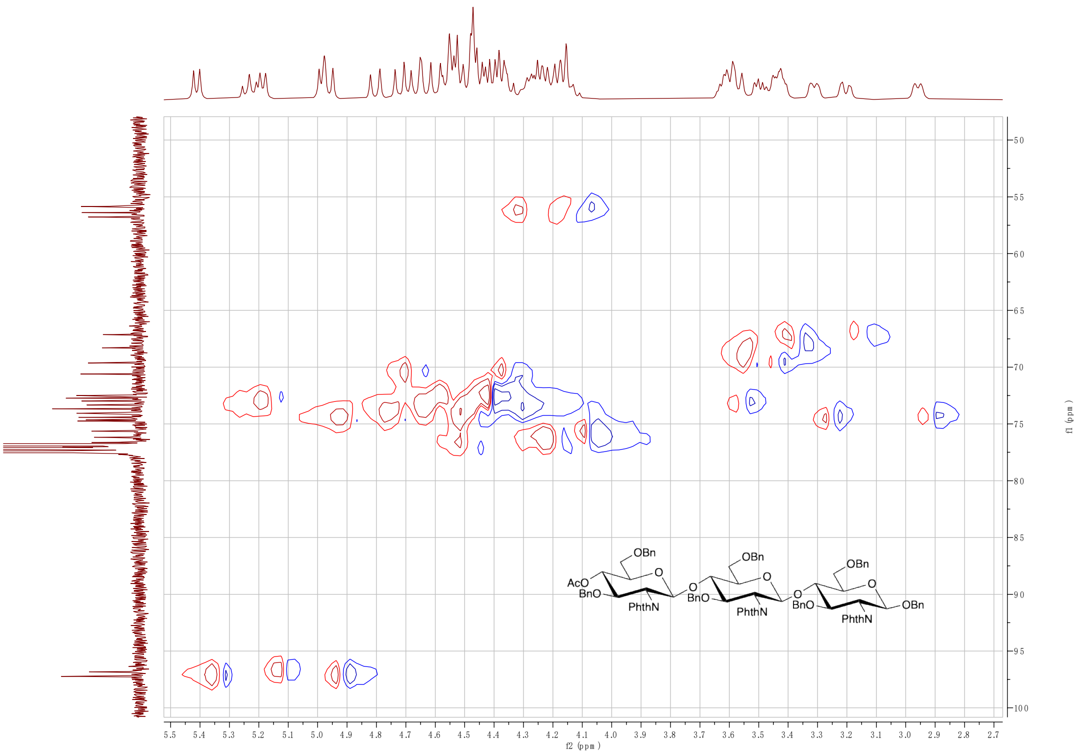

Supplementary Fig. 152 | ^{1}H-^{13}C HSQC NMR spectrum of 11 (25 °C, $CDCl_3$).

^1H, ^{13}C, ^1H-^1H COSY and ^1H-^{13}C HSQC spectra for **13**.

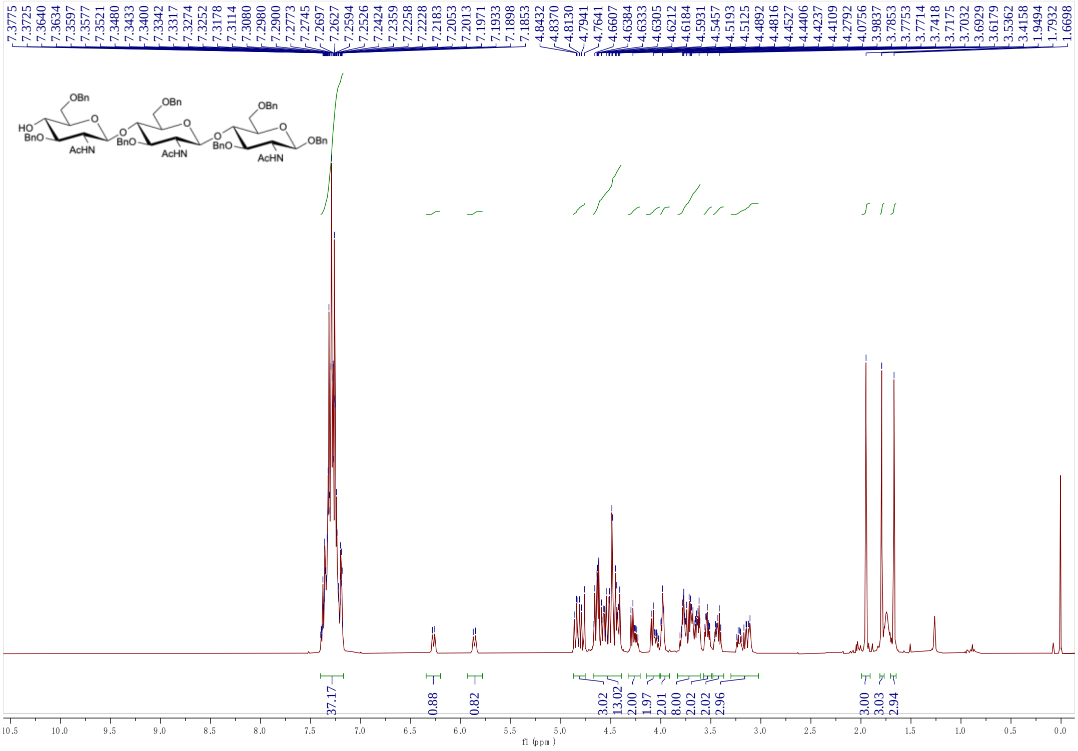

Supplementary Fig. 153 | ^1H NMR spectrum of 13 (400 MHz, 25 °C, $CDCl_3$).

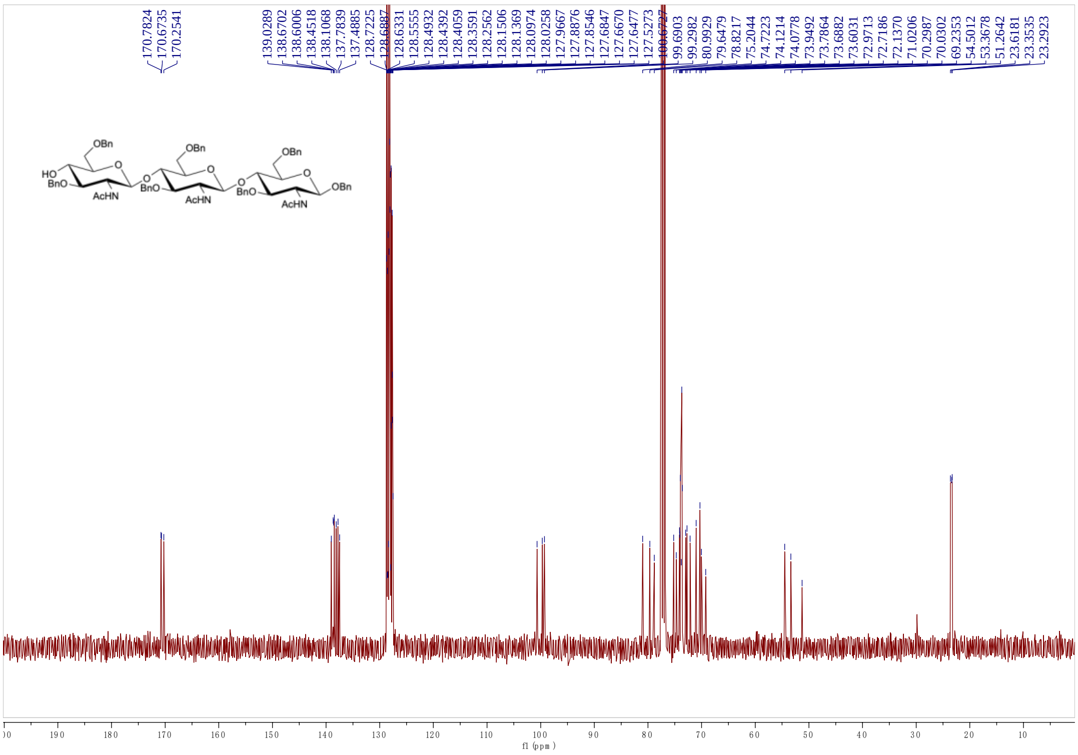

Supplementary Fig. 154 | ^{13}C NMR spectrum of 13 (100 MHz, 25 °C, $CDCl_3$).

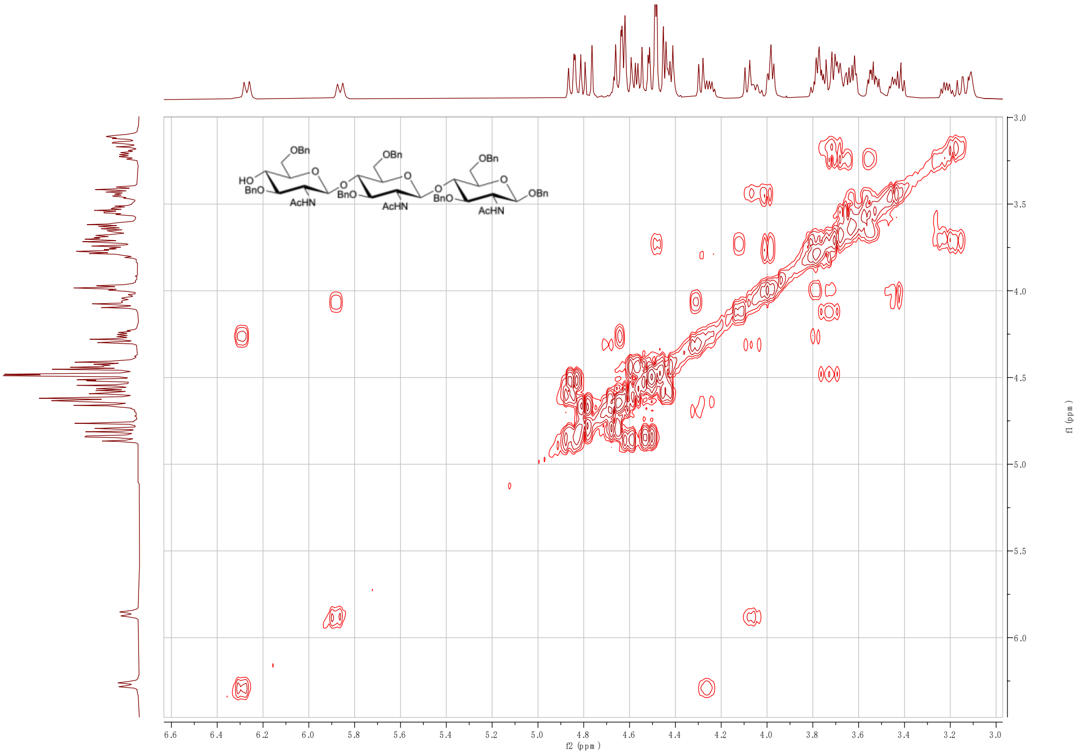

Supplementary Fig. 155 | ^{1}H-^{1}H COSY NMR spectrum of 13 (25 °C, $CDCl_3$).

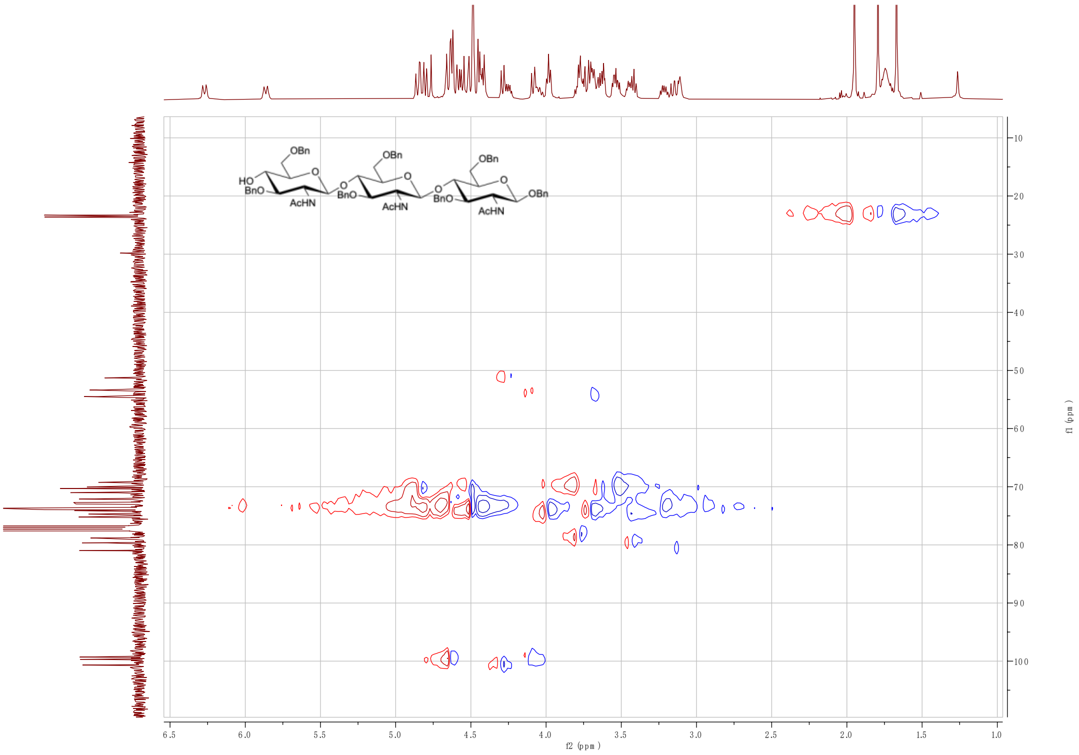

Supplementary Fig. 156 | ^{1}H-^{13}C HSQC NMR spectrum of 13 (25 °C, $CDCl_3$).

^{1}H and ^{13}C spectra for **14**.

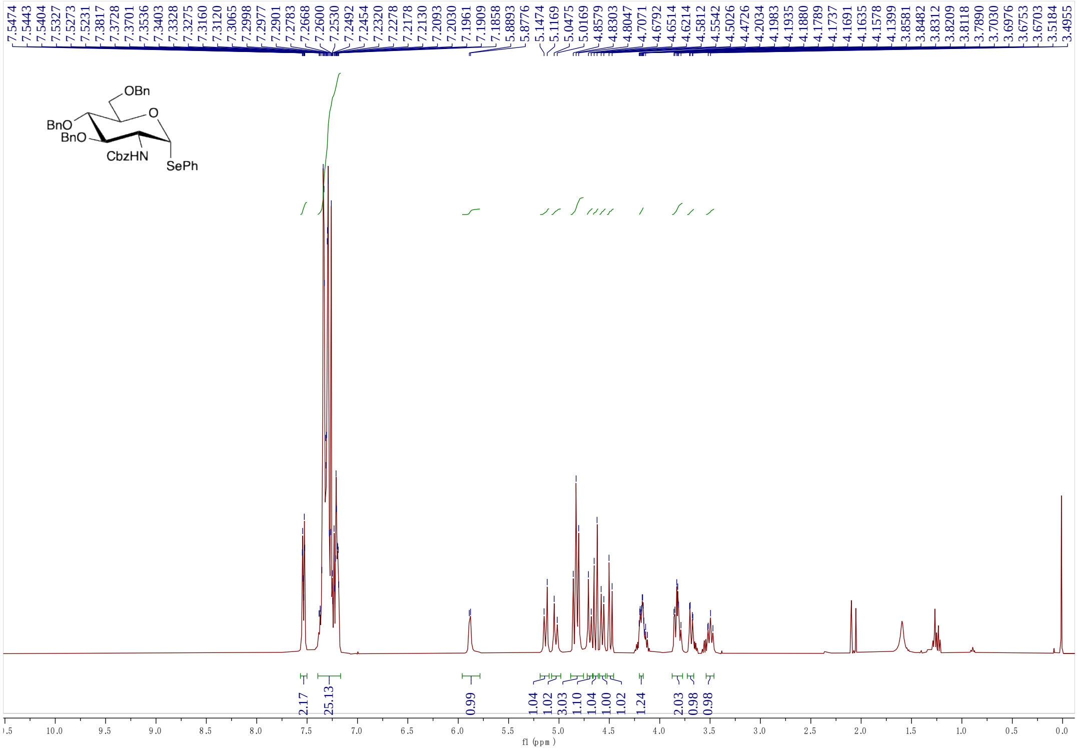

Supplementary Fig. 157 | ^{1}H NMR spectrum of 14 (400 MHz, 25 °C, $CDCl_3$).

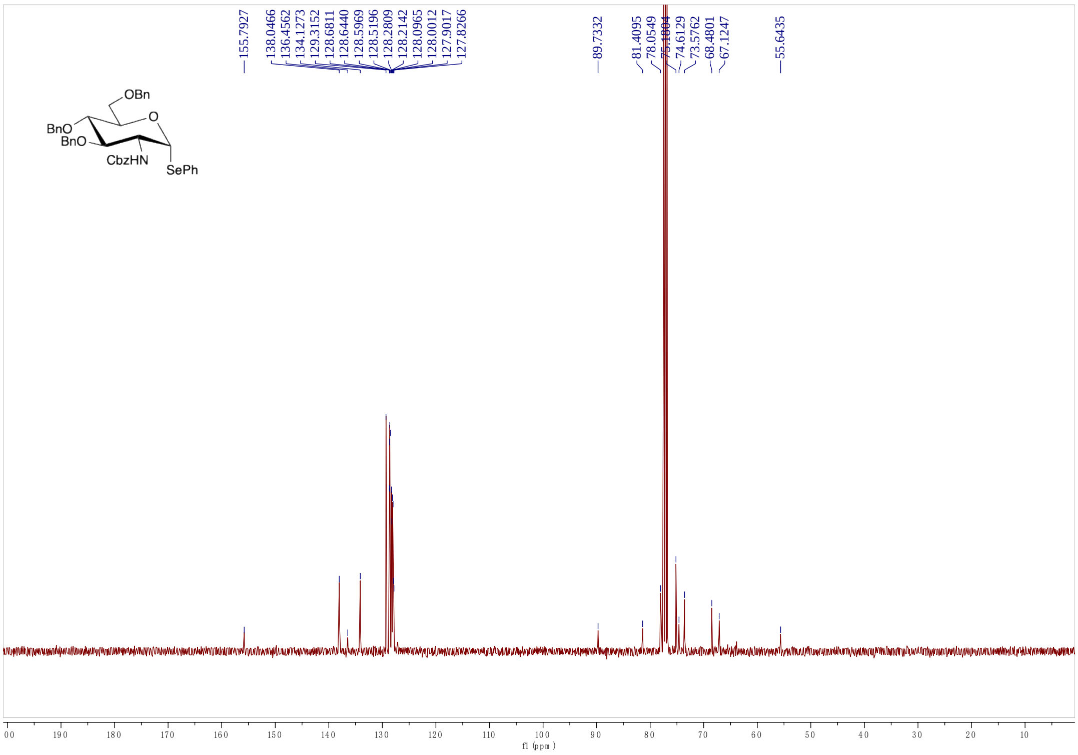

Supplementary Fig. 158 | ^{13}C NMR spectrum of 14 (100 MHz, 25 °C, $CDCl_3$).

^{1}H and ^{13}C spectra for **15**.

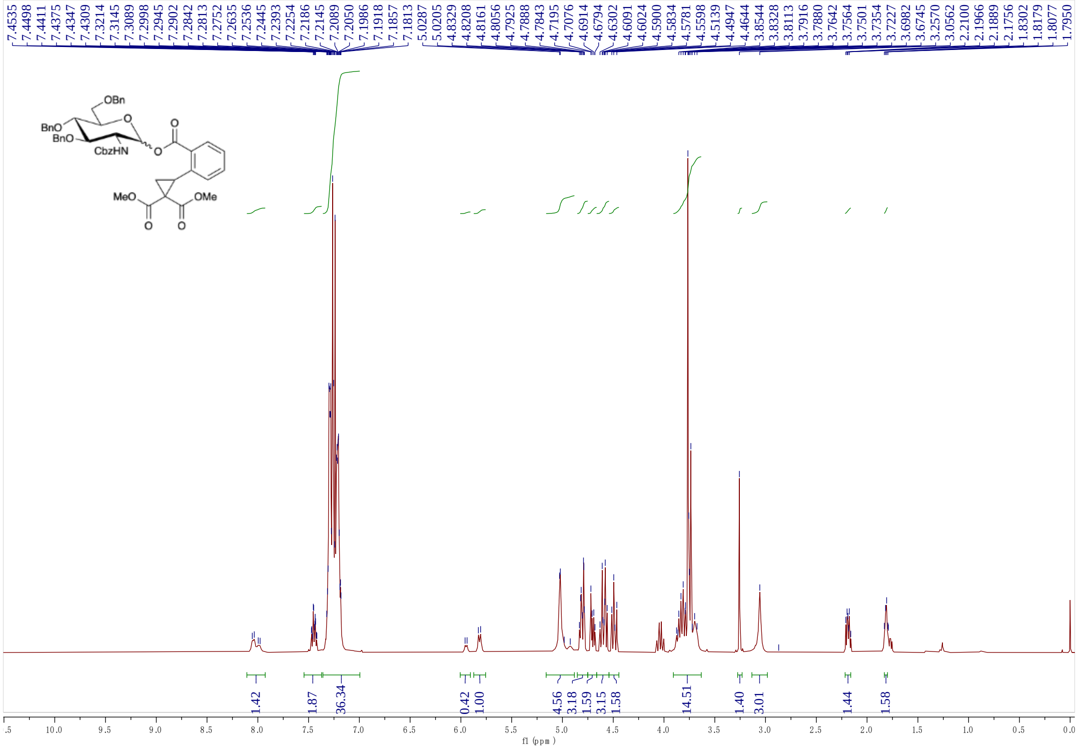

Supplementary Fig. 159 | ^{1}H NMR spectrum of 15 (400 MHz, 25 °C, $CDCl_3$).

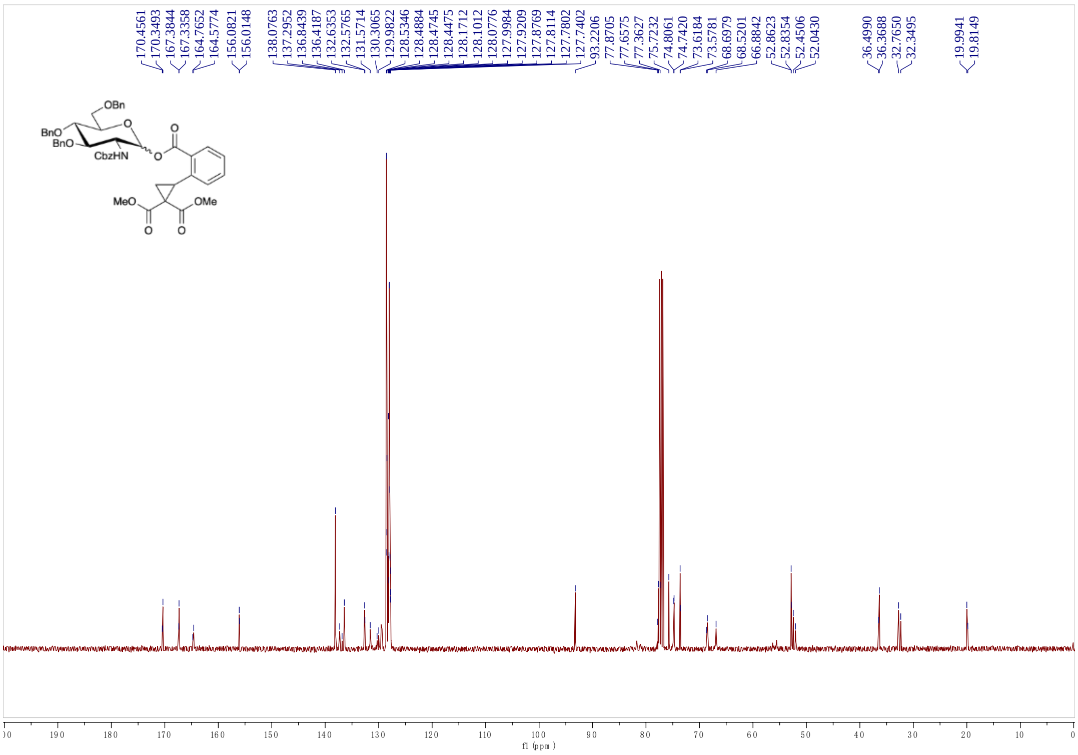

Supplementary Fig. 160 | ^{13}C NMR spectrum of 15 (100 MHz, 25 °C, $CDCl_3$).

^{1}H and ^{13}C spectra for **16**.

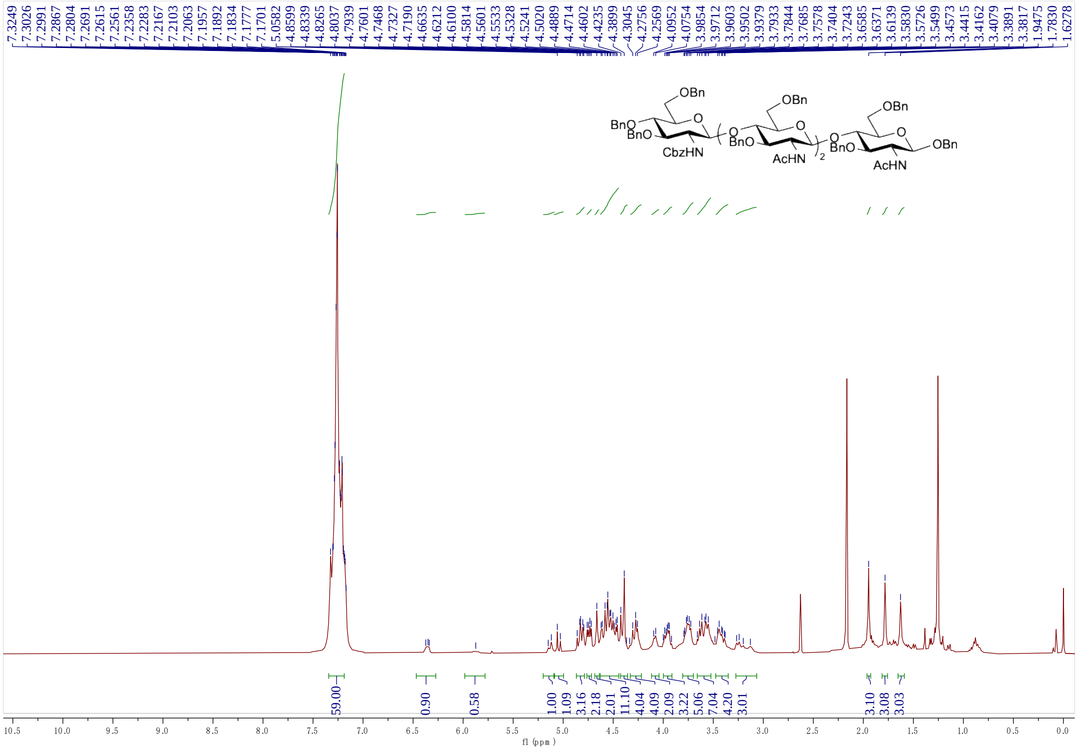

Supplementary Fig. 161 | ^{1}H NMR spectrum of 16 (400 MHz, 25 °C, $CDCl_3$).

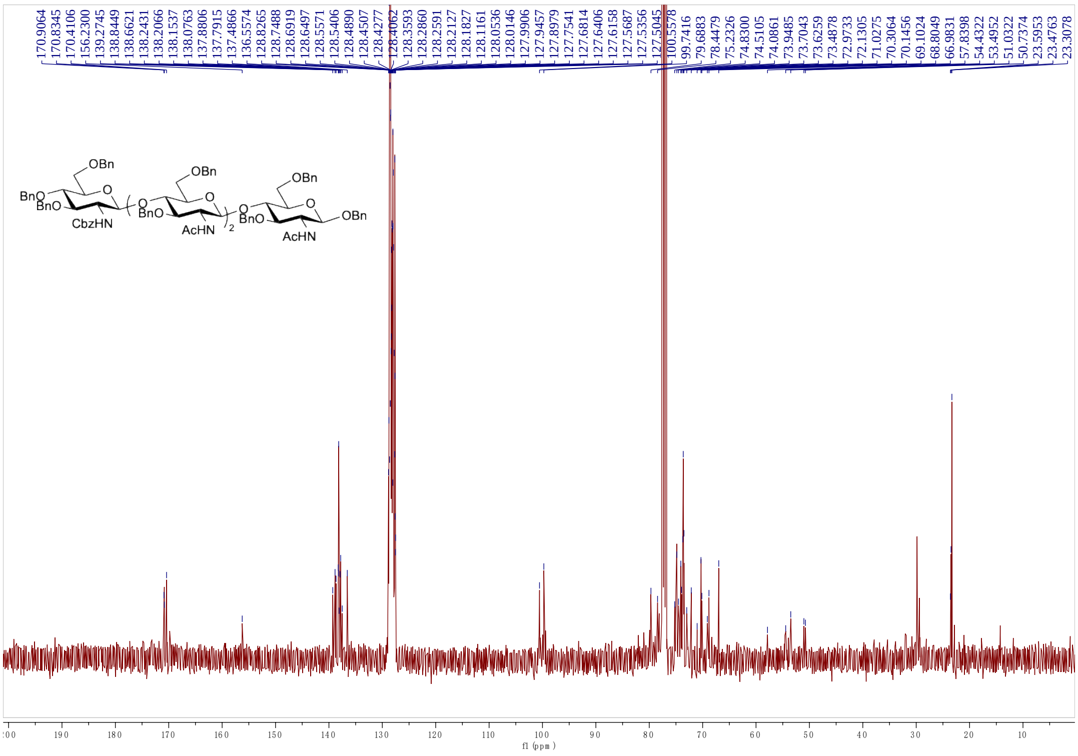

Supplementary Fig. 162 | ^{13}C NMR spectrum of 16 (100 MHz, 25 °C, $CDCl_3$).

^{1}H, ^{13}C, ^{1}H-^{1}H COSY and ^{1}H-^{13}C HSQC spectra for **17**.

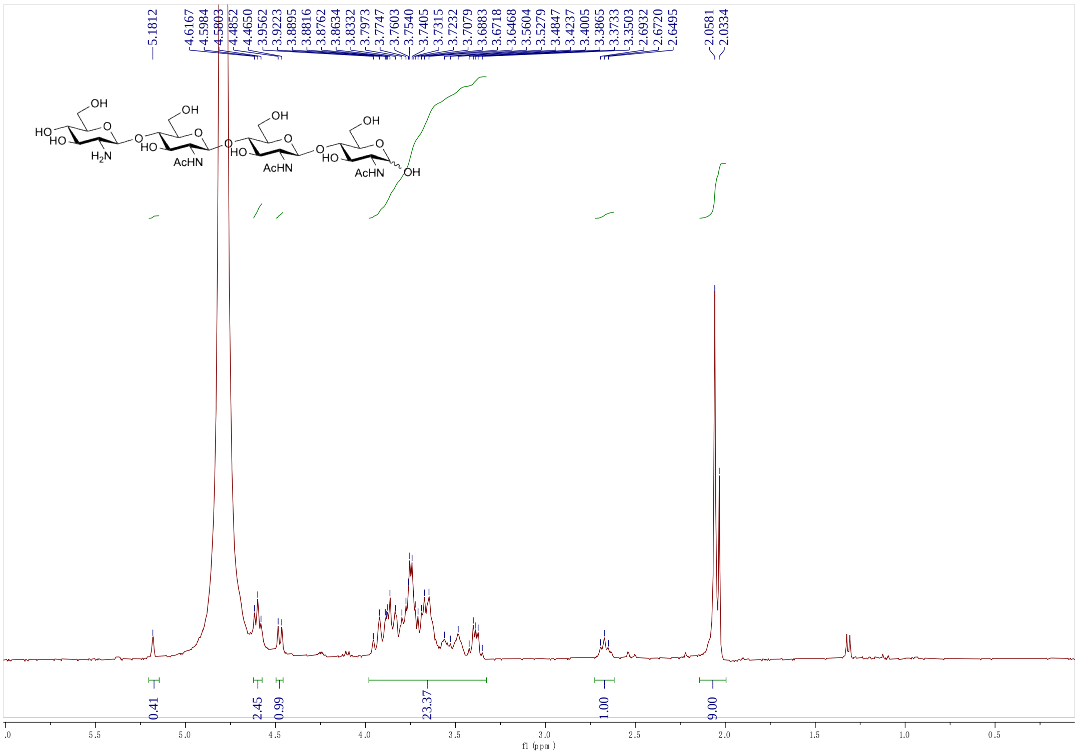

Supplementary Fig. 163 | ^{1}H NMR spectrum of 17 (400 MHz, 25 °C, D_2O).

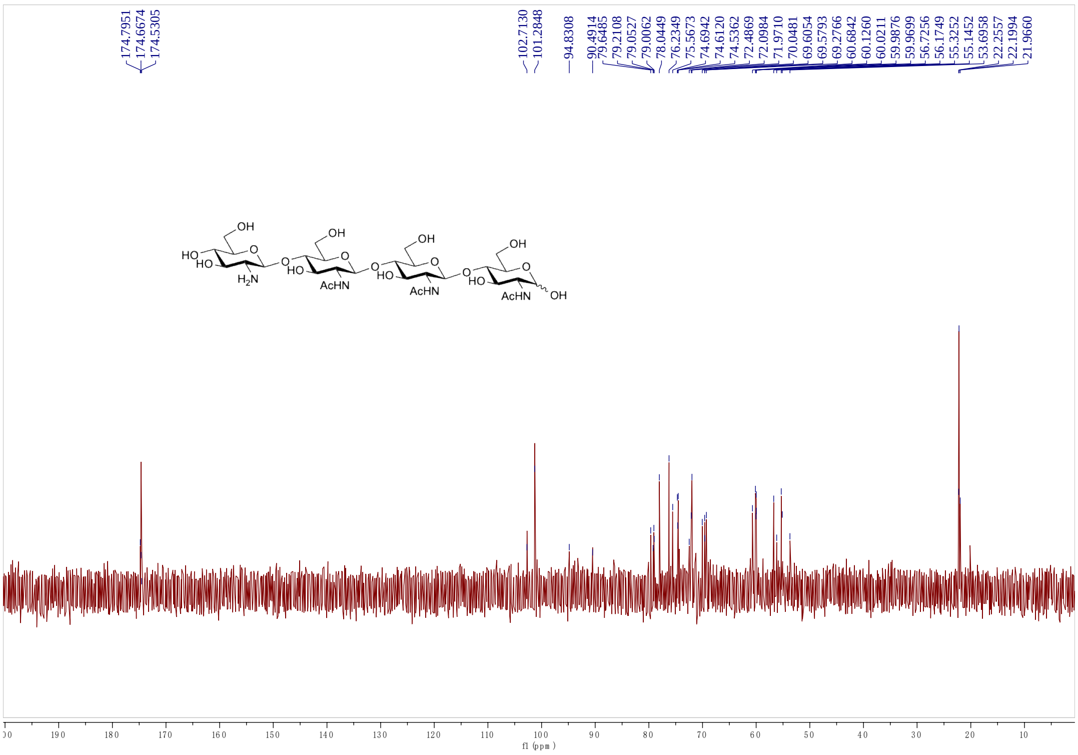

Supplementary Fig. 164 | ^{13}C NMR spectrum of 17 (100 MHz, 25 °C, D_2O).

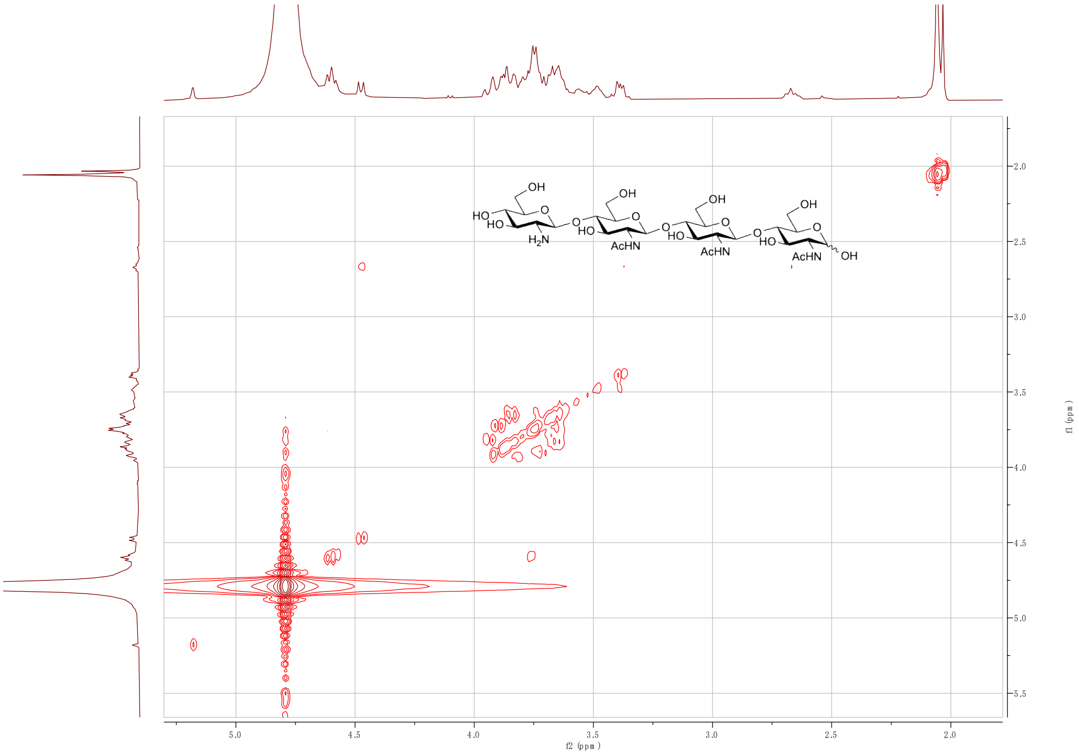

Supplementary Fig. 165 | ^{1}H-^{1}H COSY NMR spectrum of 17 (25 °C, D_2O).

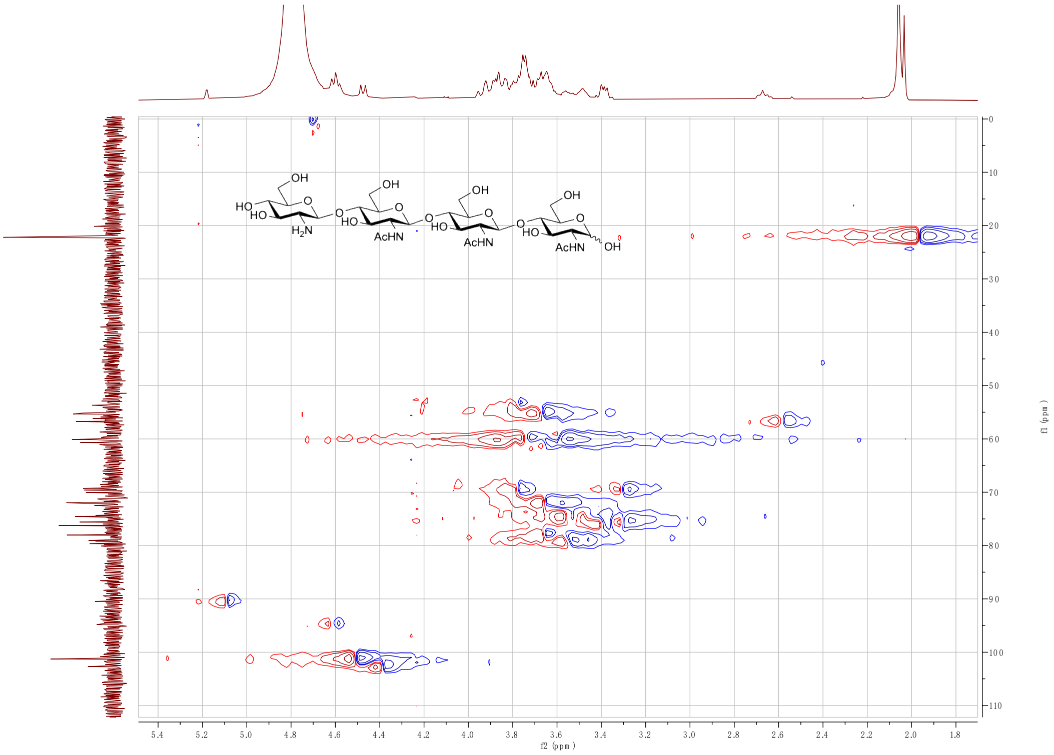

Supplementary Fig. 166 | ^{1}H-^{13}C HSQC NMR spectrum of 17 (25 °C, D_2O).

^{1}H and ^{13}C spectra for **19**.

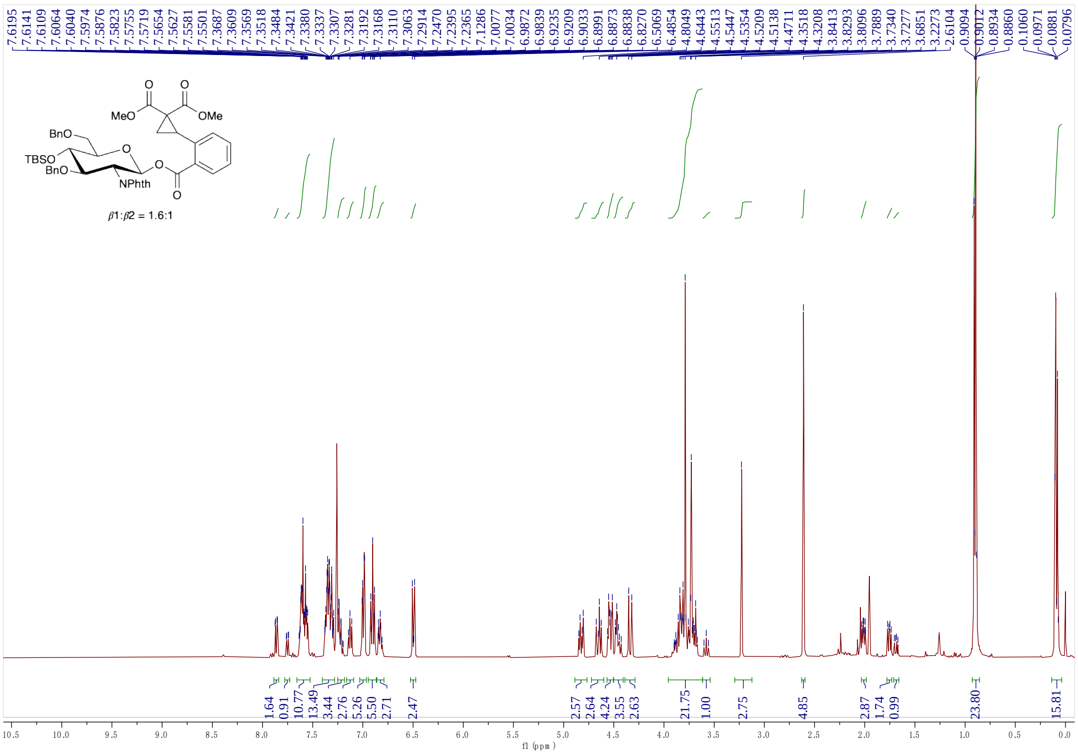

Supplementary Fig. 167 | ^{1}H NMR spectrum of 19 (400 MHz, 25 °C, $CDCl_3$).

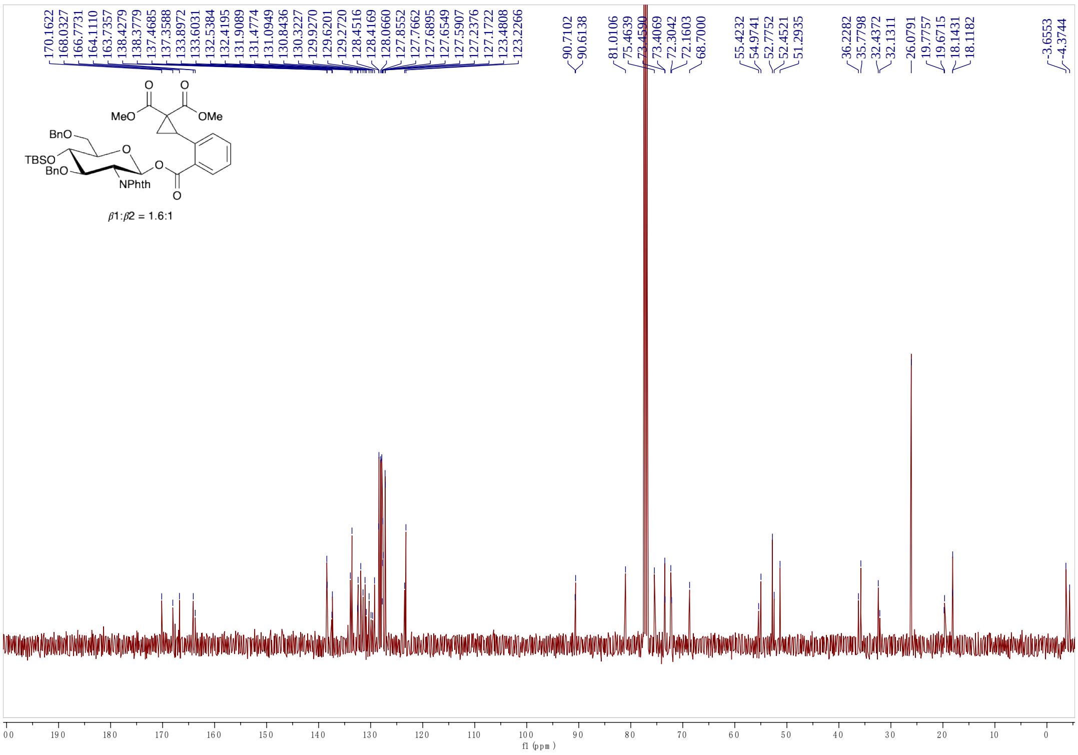

Supplementary Fig. 168 | ^{13}C NMR spectrum of 19 (100 MHz, 25 °C, $CDCl_3$).

^1H, ^{13}C, ^1H-^1H COSY and ^1H-^{13}C HSQC spectra for **21**.

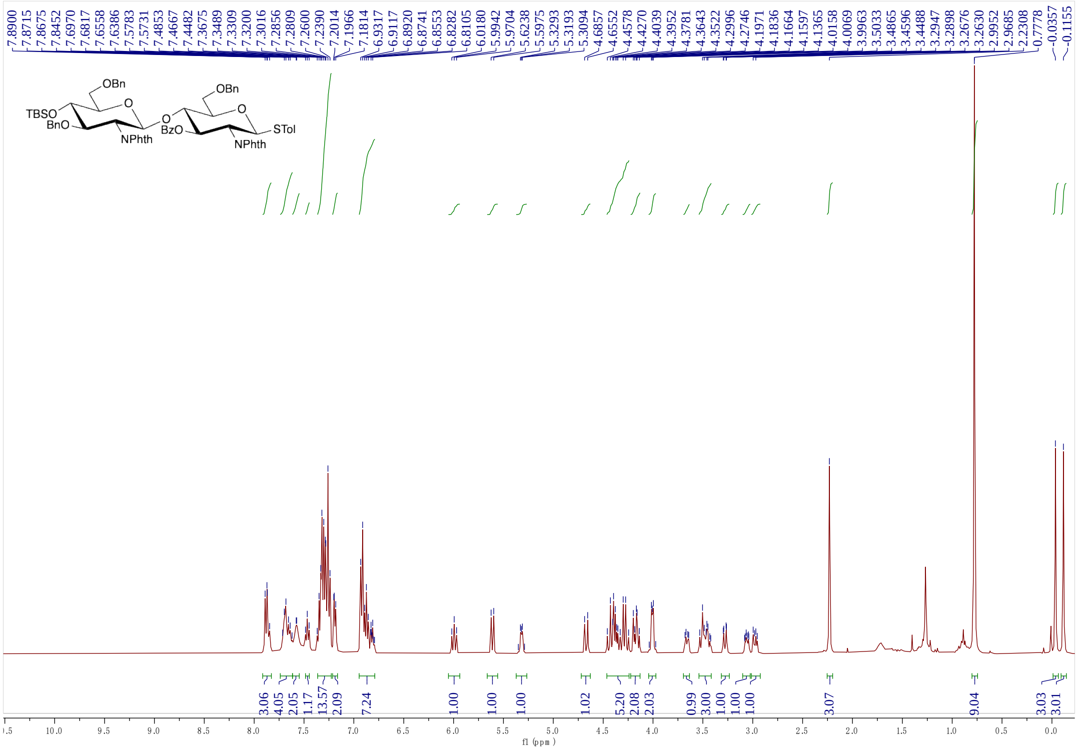

Supplementary Fig. 169 | ^1H NMR spectrum of 21 (400 MHz, 25 °C, $CDCl_3$).

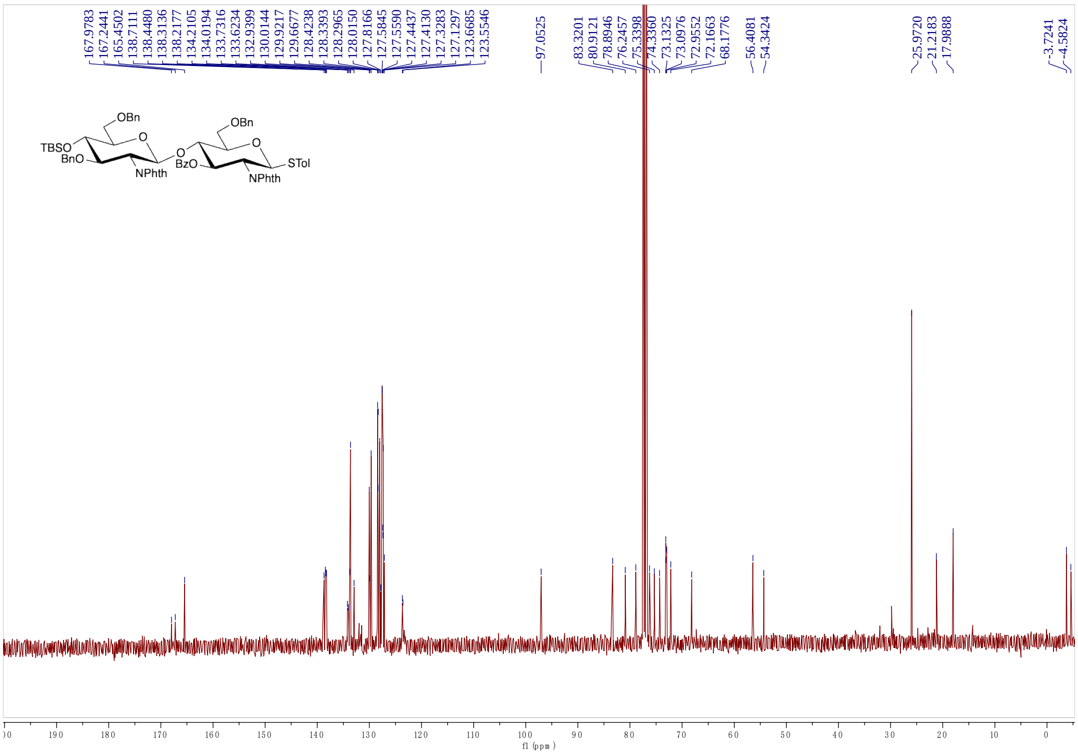

Supplementary Fig. 170 | ^{13}C NMR spectrum of 21 (100 MHz, 25 °C, $CDCl_3$).

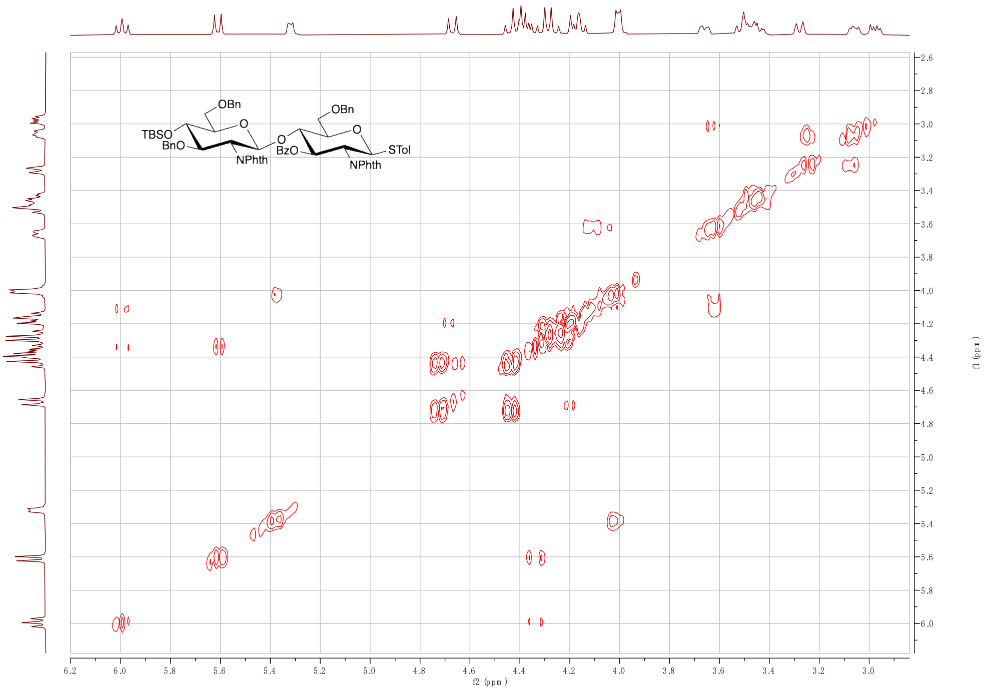

Supplementary Fig. 171 | ^{1}H-^{1}H COSY NMR spectrum of 21 (25 °C, $CDCl_3$).

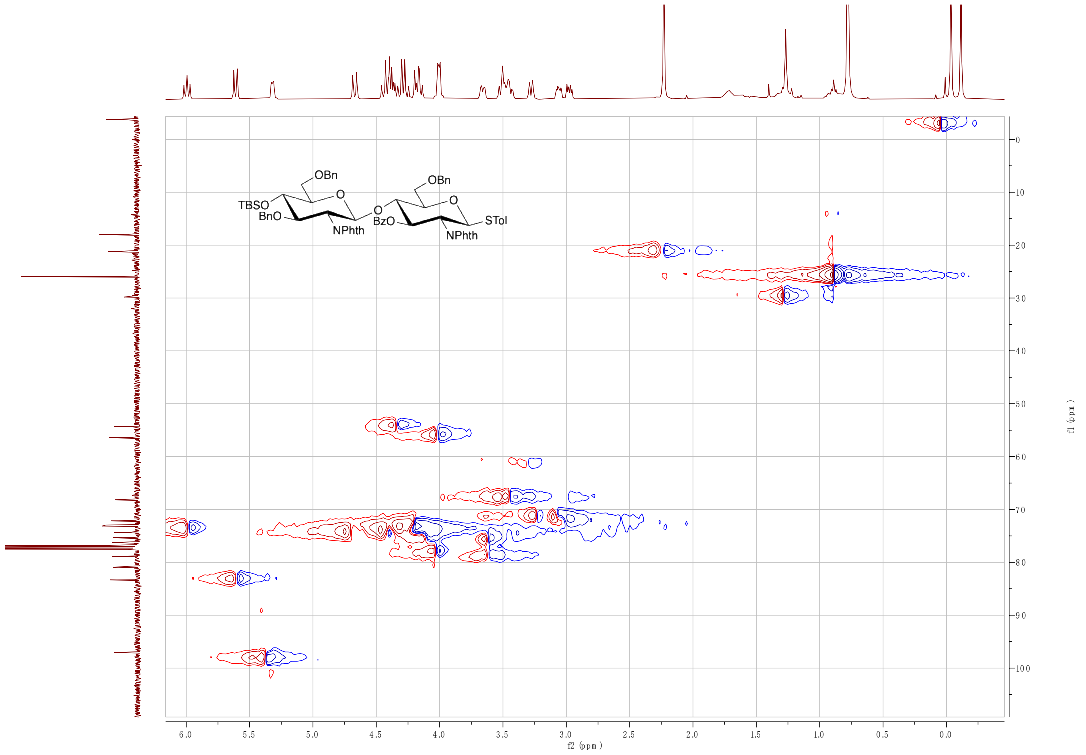

Supplementary Fig. 172 | ^{1}H-^{13}C HSQC NMR spectrum of 21 (25 °C, $CDCl_3$).

^1H, ^{13}C, ^1H-^1H COSY and ^1H-^{13}C HSQC spectra for **22**.

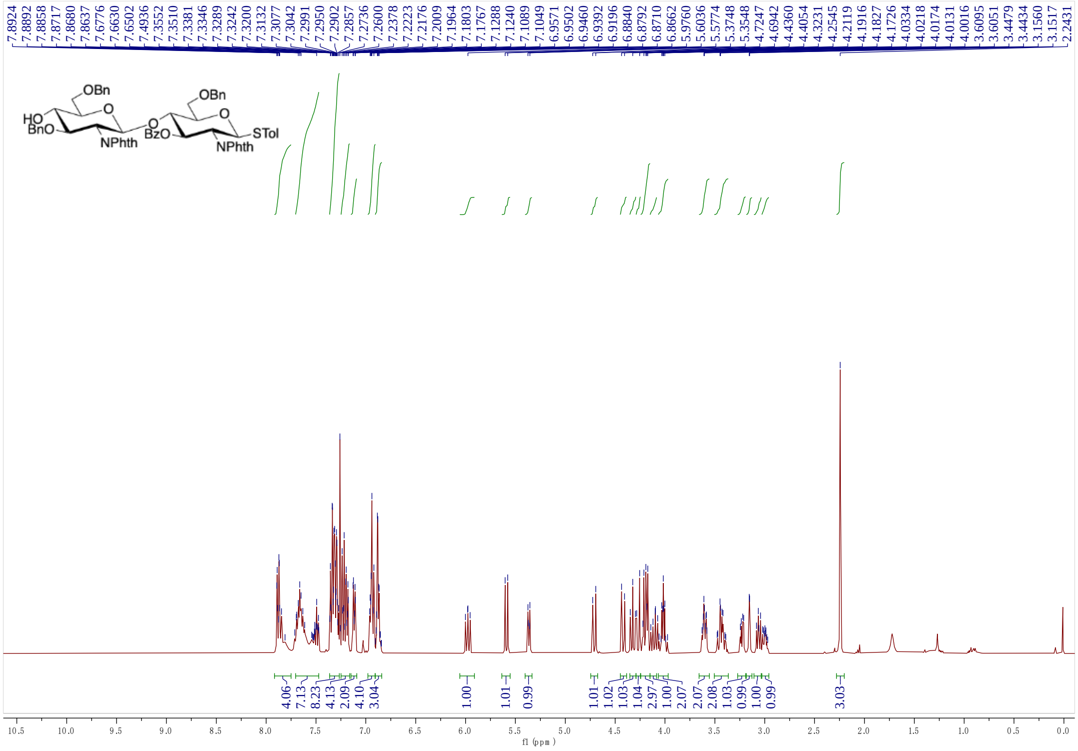

Supplementary Fig. 173 | ^1H NMR spectrum of 22 (400 MHz, 25 °C, $CDCl_3$).

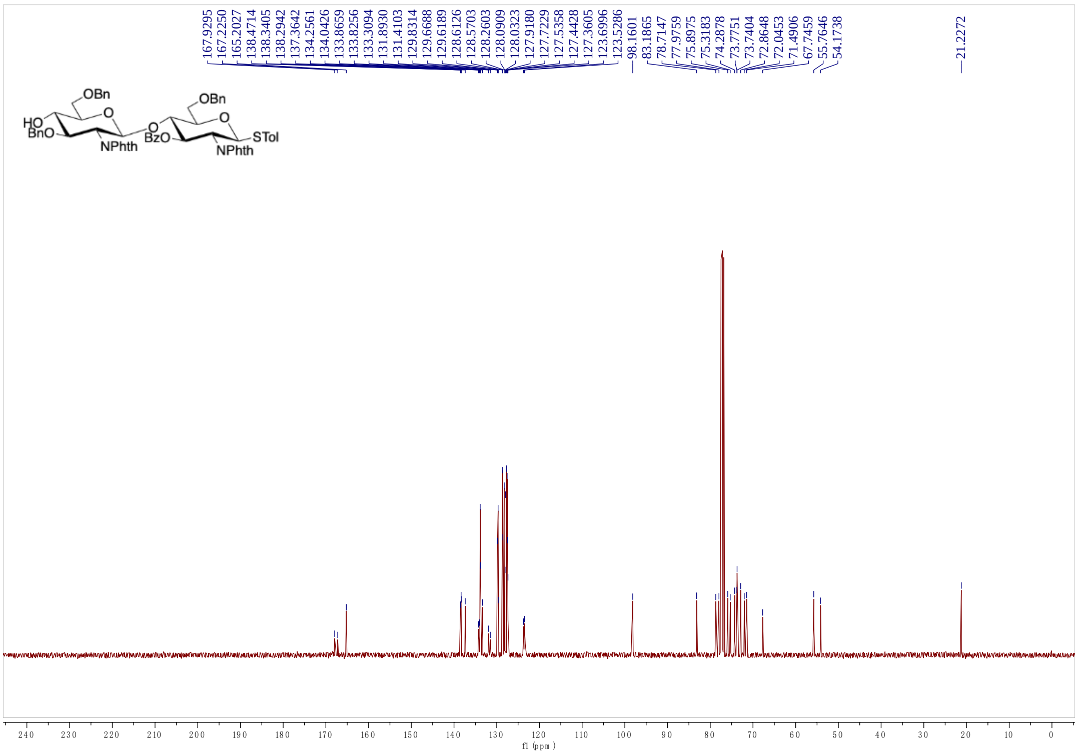

Supplementary Fig. 174 | ^{13}C NMR spectrum of 22 (100 MHz, 25 °C, $CDCl_3$).

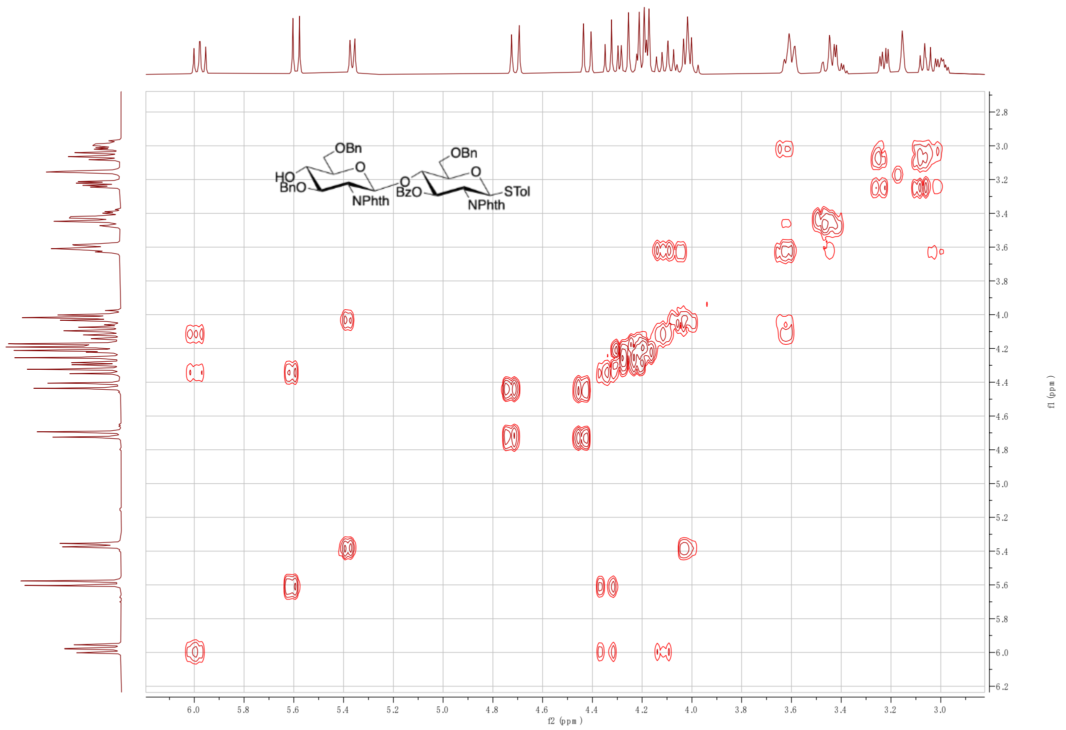

Supplementary Fig. 175 | ^{1}H-^{1}H COSY NMR spectrum of 22 (25 °C, $CDCl_3$).

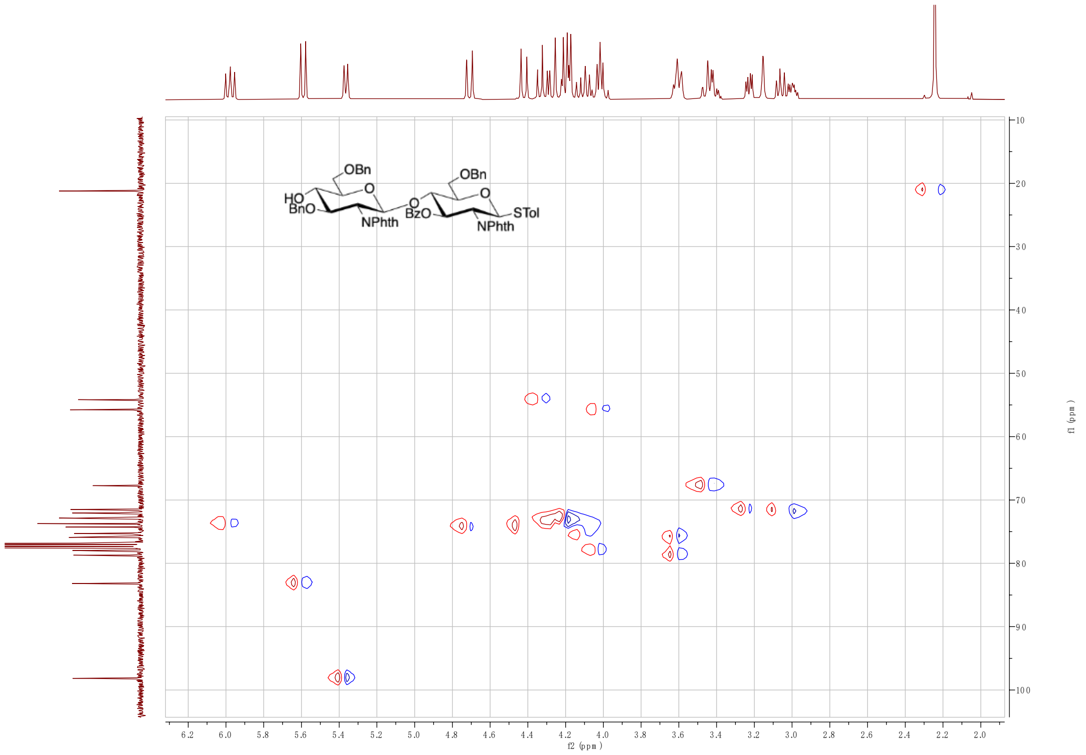

Supplementary Fig. 176 | ^{1}H-^{13}C HSQC NMR spectrum of 22 (25 °C, $CDCl_3$).

^{1}H and ^{13}C spectra for **23**.

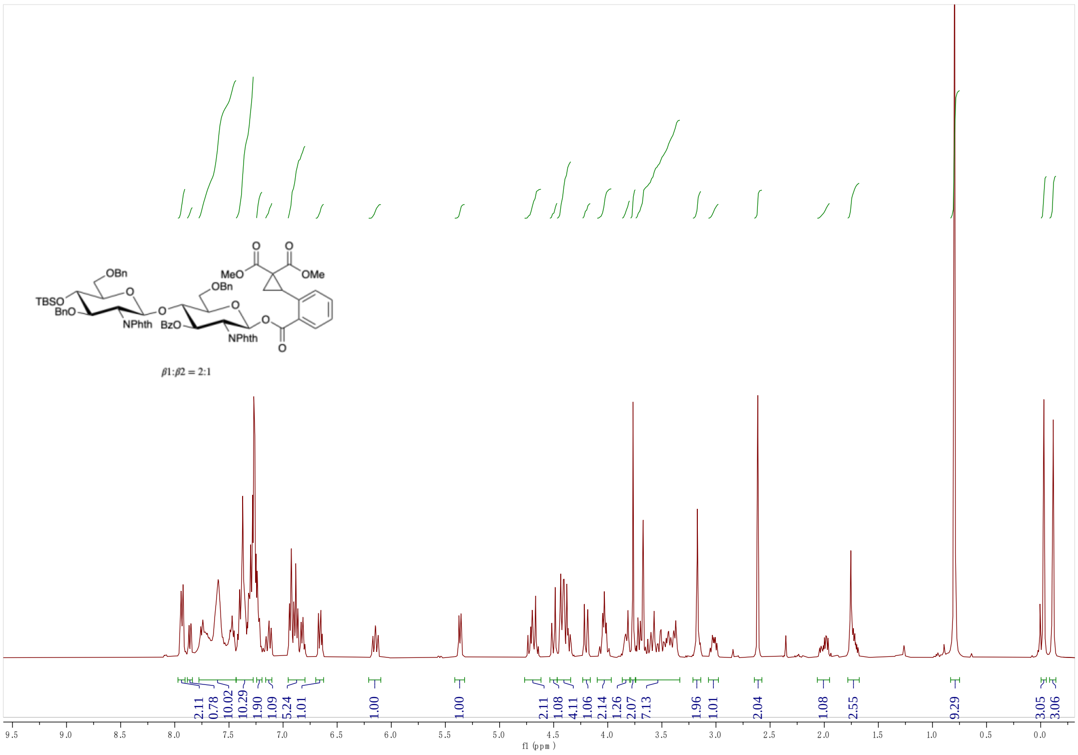

Supplementary Fig. 177 | ^{1}H NMR spectrum of 23 (400 MHz, 25 °C, $CDCl_3$).

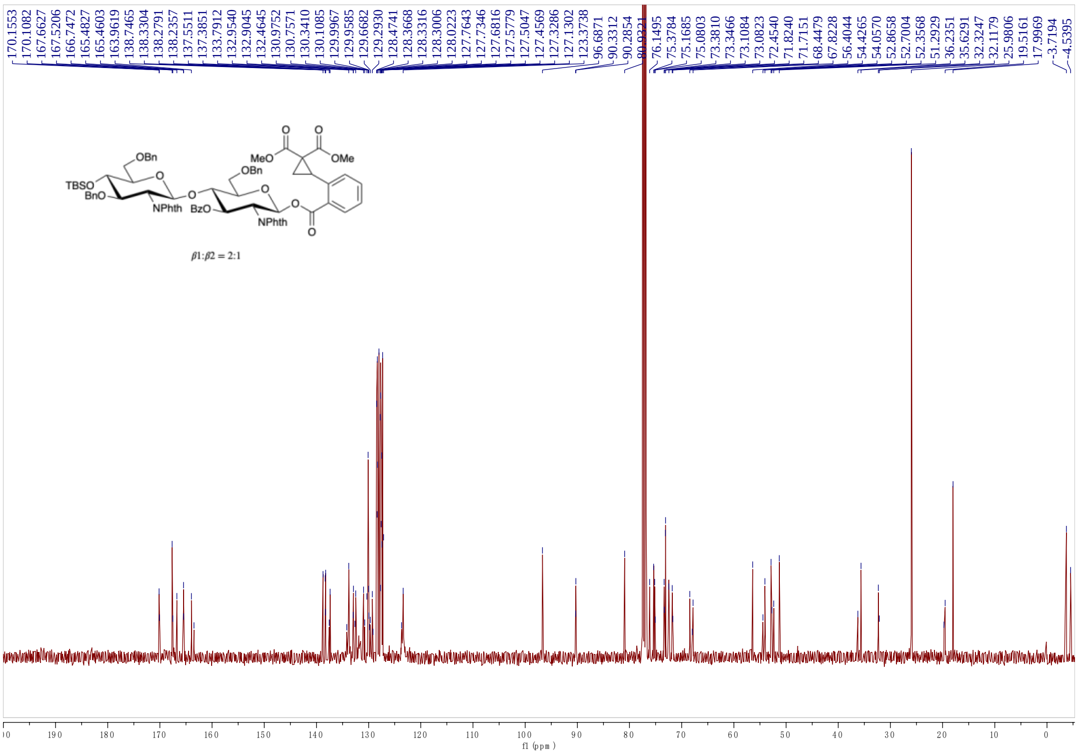

Supplementary Fig. 178 | ^{13}C NMR spectrum of 23 (100 MHz, 25 °C, $CDCl_3$).

^{1}H, ^{13}C, ^{1}H-^{1}H COSY and ^{1}H-^{13}C HSQC spectra for **24**.

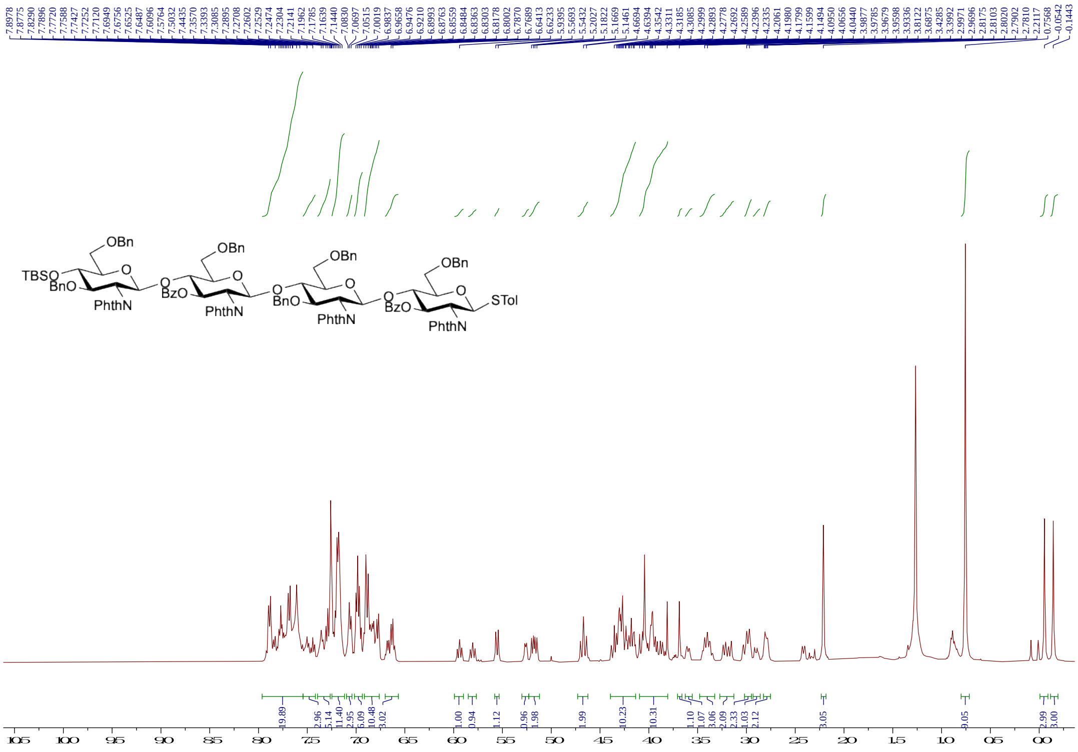

Supplementary Fig. 179 | ^{1}H NMR spectrum of 24 (400 MHz, 25 °C, $CDCl_3$).

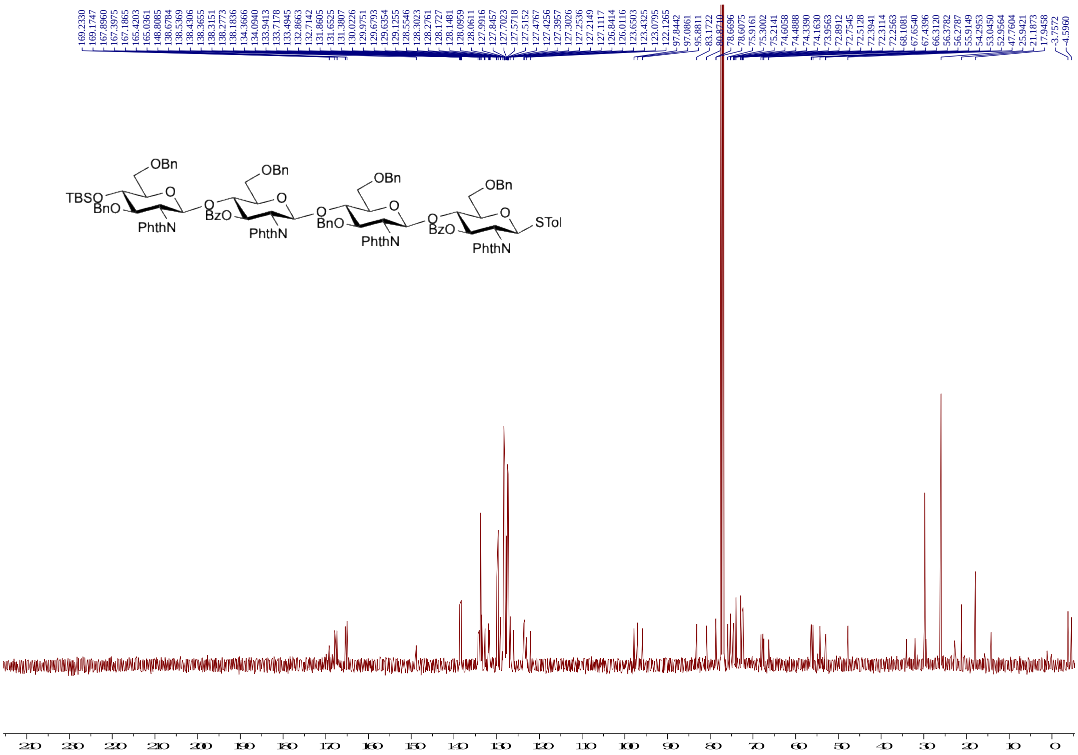

Supplementary Fig. 180 | ^{13}C NMR spectrum of 24 (100 MHz, 25 °C, $CDCl_3$).

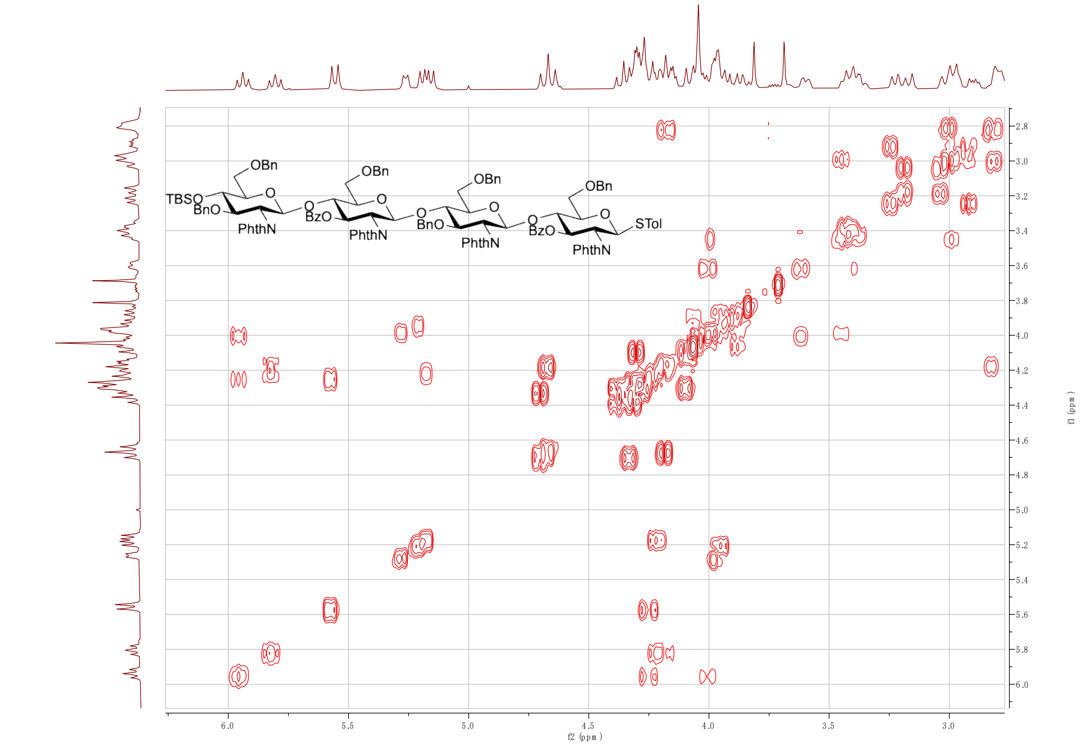

Supplementary Fig. 181 | ^{1}H-^{1}H COSY NMR spectrum of 24 (25 °C, $CDCl_3$).

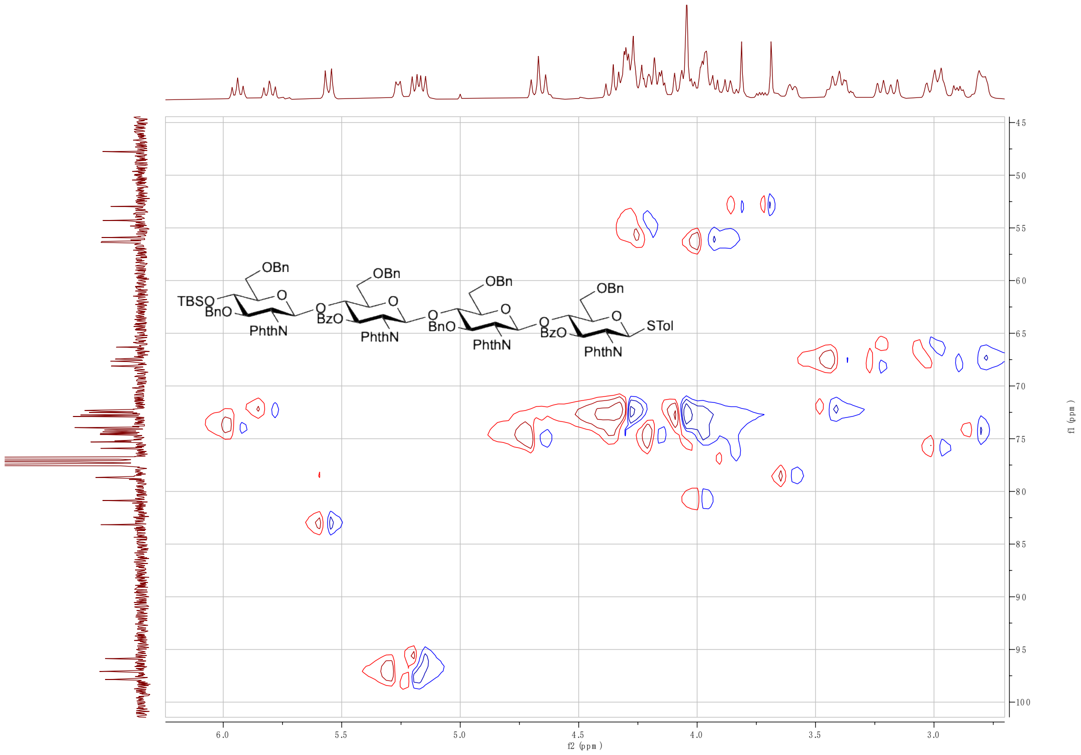

Supplementary Fig. 182 | ^{1}H-^{13}C HSQC NMR spectrum of 24 (25 °C, $CDCl_3$).

Section 7. Supplementary References.

1. Dias, D. A. & Kerr, M. A. Domino synthesis of bridged bicyclic tetrahydro-1,2-oxazines: Access to stereodefined 4-aminocyclohexanols. *Org. Lett.* **11**, 3694–3697 (2009).
2. Huo, G., Liu, C., Hui, Y., Chen, X. & Xiao, D. Synthesis and structure-activity relationship of oleanolic mono- or di-glycosides against Magnaporthe oryzae. *Genetics & Molecular Research*, **15**, gmr.15038998 (2016).
3. Lankalapalli, R. S., Baksa, A., Liliom, K. & Bittman, R. Synthesis and properties of a photoactivatable analogue of psychosine (β-galactosylsphingosine). *ChemMedChem* **5**, 682–686 (2010).
4. Hartmann, M., Betz, P., Sun, Y., Gorb, S. N., Lindhorst, T. K. & Krueger, A. Saccharide-modified nanodiamond conjugates for the efficient detection and removal of pathogenic bacteria. *Chem. Eur. J.* **18**, 6485–6492 (2012).
5. Mehta, S. & Pinto, B. M. Novel glycosidation methodology. The use of phenyl selenoglycosides as glycosyl donors and acceptors in oligosaccharide synthesis. *J. Org. Chem.* **58**, 3269–3276 (1993).
6. Doyle, L. M., O'Sullivan, S., Salvo, C. D., McKinney, M., McArdle, P. & Murphy. P. V. Stereoselective epimerizations of glycosyl thiols. *Org. Lett.* **19**, 5802–5805 (2017).
7. Wang, R., Chen, J.-Z., Zheng, X.-A., Kong, R., Gong, S.-S. & Sun, Q. Hafnium(IV) triflate as a potent catalyst for selective 1-*O*-deacetylation of peracetylated saccharides *Carbohydr. Res.* **455**, 114–118 (2018).
8. Qiao, Z., Wang, P., Ni, J., Li, D., Sun, Y., Li, T. & Li, M. Triflic imide-catalyzed glycosylation of disarmed glycosyl *ortho*-isopropenylphenylacetates and *ortho*-isopropenylbenzyl thioglycosides. *Eur. J. Org. Chem.* **2022**, e202101367 (2022).
9. Li, Y., Yang, X., Liu, Y., Zhu, C., Yang, Y. & Yu. B. Gold(I)-catalyzed glycosylation with glycosyl *ortho*-alkynylbenzoates as donors: General scope and application in the synthesis of a cyclic triterpene saponin. *Chem. Eur. J.* **16**, 1871–1882 (2010).

10. Ghosh, T., Mukherji, A., Srivastava, H. K. & Kancharla, P. K. Secondary amine salt catalyzed controlled activation of 2-deoxy sugar lactols towards alpha-selective dehydrative glycosylation. *Org. Biomol. Chem.* **16**, 2870–2875 (2018).
11. Balmond, E. I., Benito-Alifonso, D., Coe, D. M., Alder, R. W., McGarrigle, E. M. & Galan, C. A 3,4-*trans*-fused cyclic protecting group facilitates α-selective catalytic synthesis of 2-deoxyglycosides. *Angew. Chem. Int. Ed.* **53**, 8190–8194 (2014).
12. Ji, P., Zhang, Y., Wei, Y., Huang, H., Hu, W., Mariano, P. A. & Wang, W. Visible-light-mediated, chemo- and stereoselective radical process for the synthesis of *C*-glycoamino acids. *Org. Lett.* **21**, 3086–3092 (2019).
13. Lonnecker, A. T., Lim, Y. H., Felder, S. E., Besset, C. J. & Wooley, K. L. Four different regioisomeric polycarbonates derived from one natural product, D-glucose. *Macromolecules* **49**, 7857–7867 (2016).
14. Beignet, J., Tiernan, J., Woo, C. H., Kariuki, B. M. & Cox, L. R. Stereoselective synthesis of allyl-*C*-mannosyl compounds: Use of a temporary silicon connection in intramolecular allylation strategies with allylsilanes. *J. Org. Chem.* **69**, 6341–6356 (2004).
15. Ingle, A. B., Chao, C.-S., Hung, W.-C. & Mong, K.-K. T. Tuning reactivity of glycosyl imidinium intermediate for 2-azido-2-deoxyglycosyl donors in α-glycosidic bond formation. *Org. Lett.* **15**, 5290–5293 (2013).
16. Trujillo, M., Morales, E. Q. & Vazquez, J. T. Tetra-*O*-benzoylglucosylation: A new ^{1}H nuclear magnetic resonance method for determination of the absolute configuration of secondary alcohols. *J. Org. Chem.* **59**, 6637–6642 (1994).
17. Steber, H. B., Singh, Y. & Demchenko A. V. Bismuth(III) triflate as a novel and efficient activator for glycosyl halides. *Org. Biomol. Chem.* **19**, 3220–3233 (2021).
18. Kawa, K., Saitoh, T., Kaji, E. & Nishiyama, S. Development of glycosylation using the glucopyranose 1,2-orthobenzoate under electrochemical conditions. *Org. Lett.* **2013**, *15*, 5484–5487 (2013).

19. Xiao, X., Zhao, Y., Shu, P., Zhao, X., Liu, Y., Sun, J., Zhang, Q., Zeng, Q. & Wan, Q. Remote activation of disarmed thioglycosides in latent-active glycosylation *via* interrupted Pummerer reaction. *J. Am. Chem. Soc.* **138**, 13402–13407 (2016).
20. Liu, R., Hua, Q., Lou, Q., Wang, J., Li, X., Ma, Z. & Yang, Y. NIS/TMSOTf-promoted glycosidation of glycosyl *ortho*-hexynylbenzoates for versatile synthesis of *O*-glycosides and nucleosides. *J. Org. Chem.* **86**, 4763–4778 (2021).
21. Das, A. & Jayaraman, N. Carbon tetrachloride-free allylic halogenation-mediated glycosylations of allyl glycosides. *Org. Biomol. Chem.* **19**, 9318–9325 (2021).
22. Thadke, S. A., Neralkar, M. & Hotha, S. Facile synthesis of aminooxy glycosides by gold(III)-catalyzed glycosidation. *Carbohydr. Res.* **430**, 16–23 (2016).
23. Mishra, B., Neralkar, M. & Hotha, S. Stable alkynyl glycosyl carbonates: Catalytic anomeric activation and synthesis of a tridecasaccharide reminiscent of *Mycobacterium tuberculosis* cell wall lipoarabinomannan *Angew. Chem. Int. Ed.* **55**, 7786–7791 (2016).
24. Long, Q., Gao, J., Yan, N., Wang, P. & Li, M. $(C_6F_5)_3B{\cdot}(HF)_n$-catalyzed glycosylation of disarmed glycosyl fluorides and reverse glycosyl fluorides. *Org. Chem. Front.* **8**, 3332–3341 (2021).
25. Liu, M., Li, B.-H., Xiong, D.-C. & Ye, X.-S. *O*-Glycosylation enabled by *N*-(glycosyloxy)acetamides. *J. Org. Chem.* **83**, 8292–8303 (2018).
26. Carthy, C. M., Tacke, M. & Zhu, X. *N*-Trifluoromethylthiosaccharin/TMSOTf: A new mild promoter system for thioglycoside activation. *Eur. J. Org. Chem.* **16**, 2729–2734 (2019).
27. Singh, Y., Wang, T., Geringer, S. A., Stine, K. J. & Demchenko, A. V. Regenerative glycosylation. *J. Org. Chem.* **83**, 374–381 (2018).
28. Hu, Y., Yu, K., Shi, L.-L., Liu, L., Sui, J.-J., Liu, D.-Y., Xiong, B. & Sun, J.-S. *o*-(*p*-Methoxyphenylethynyl)phenyl glycosides: Versatile new glycosylation donors for the highly efficient construction of glycosidic linkages. *J. Am. Chem. Soc.* **139**, 12736–12744 (2017).

29. Serna, S., Kardak, B., Reichardt, N.-C. & Martin-Lomas, M. Synthesis of a core trisaccharide building block for the assembly of *N*-glycan neoconjugates. *Tetrahedron: Asymmetry* **20**, 851–856 (2009).

30. Holloran, N., Collins, D., Rathnayake, U., Zhang, B., Koh, M., Kang, C. H. & Garner, P. Site-specific synthesis of cysteine-bridged glycoproteins *via* expressed protein glycoligation. *Bioconjugate Chem.* **31**, 2362–2366 (2020).

31. Xiao, X., Ding, H.; Peng, L.-C., Fang, X.-Y., Qin, Y.-Y., Mu, Q.-Q. & Liu, X.-W. Sweet strain release: Donor-acceptor cyclopropane mediated glycosylation. *CCS Chem.* doi: 10.31635/ccschem.023.202202671 (2023).

32. He, H., Xu, L., Sun, R., Zhang, Y., Huang, Y., Chen, Z., Li, P., Yang, R. & Xiao, G. An orthogonal and reactivity-based one-pot glycosylation strategy for both glycan and nucleoside synthesis: Access to TMG-chitotriomycin, lipochitooligosaccharides and capuramycin. *Chem. Sci.* **12**, 5143–5151 (2021).

33. Shih, H.-W., Chen, K.-T., Cheng, T.-J. R., Wong, C.-H. & Cheng, W.-C. A new synthetic approach toward bacterial transglycosylase substrates, lipid II and lipid IV *Org. Lett.* **13**, 4600–4603 (2011).

34. Gong, J., Liu, H., Nicholls, J. M. & Li, X. Studies on the sialylation of galactoses with different C-5 modified sialyl donors. *Carbohydr. Res.* **361**, 91–99 (2012).
